# Biodiversity of carbapenem-resistant bacteria in clinical samples from the Southwest Amazon region (Rondônia/Brazil)

**DOI:** 10.1038/s41598-024-59733-w

**Published:** 2024-04-23

**Authors:** Levy Assis dos Santos, Rodrigo Cayô, Tiago Barcelos Valiatti, Ana Cristina Gales, Larissa Fatarelli Bento de Araújo, Fernando Marques Rodrigues, Tatiane Silva de Carvalho, Marcos André Braz Vaz, Marcela Campanharo

**Affiliations:** 1 Federal University of Rondônia Foundation (UNIR), Postgraduate Program in Conservation and Use of Natural Resources (PPGReN), Porto Velho, RO Brazil; 2 Central Public Health Laboratory of Rondônia (LACEN/RO), Medical Biology Center, Porto Velho, RO Brazil; 3 Laboratory ALERTA, Department of Medicine, Paulista School of Medicine (EPM), Federal University of São Paulo (UNIFESP), São Paulo, SP Brazil; 4 Laboratory of Bacteriology and Immunology (LIB), Department of Biological Sciences (DCB), Institute of Environmental, Chemical and Pharmaceutical Sciences (ICAQF), Federal University of São Paulo (UNIFESP), Diadema, SP Brazil; 5 Department of Informatics and Statistics, Federal University of Santa Catarina (UFSC), Florianópolis, SC Brazil; 6 Department of Agricultural and Biological Sciences (DCAB), Federal University of Espirito Santo, São Mateus, ES, Brazil

**Keywords:** Surveillance epidemiological, Antimicrobial resistance, Carbapenemases, Multi-drug resistant bacteria, Public health, Antimicrobial resistance, Clinical microbiology, Bacterial genes, Policy and public health in microbiology, Biodiversity

## Abstract

Brazil is recognized for its biodiversity and the genetic variability of its organisms. This genetic variability becomes even more valuable when it is properly documented and accessible. Understanding bacterial diversity through molecular characterization is necessary as it can improve patient treatment, reduce the length of hospital stays and the selection of resistant bacteria, and generate data for health and epidemiological surveillance. In this sense, in this study, we aimed to understand the biodiversity and molecular epidemiology of carbapenem-resistant bacteria in clinical samples recovered in the state of Rondônia, located in the Southwest Amazon region. Retrospective data from the Central Public Health Laboratories (LACEN/RO) between 2018 and 2021 were analysed using the Laboratory Environment Manager Platform (GAL). Seventy-two species with carbapenem resistance profiles were identified, of which 25 species carried at least one gene encoding carbapenemases of classes A (*bla*_KPC_-like), B (*bla*_NDM_-like, *bla*_SPM_-like or *bla*_VIM_-like) and D (*bla*_OXA-23_-like, *bla*_OXA-24_-like, *bla*_OXA-48_-like, *bla*_OXA-58_-like or *bla*_OXA-143_-like), among which we will highlight *Klebsiella pneumoniae*, *Pseudomonas aeruginosa*, *Acinetobacter baumannii*, *Serratia marcescens*, and *Providencia* spp. With these results, we hope to contribute to the field by providing epidemiological molecular data for state surveillance on bacterial resistance and assisting in public policy decision-making.

## Introduction

Brazil is recognized for its biodiversity. Often associated with the vast spectrum of macroorganisms, Brazil's biodiversity is equally profound among microorganisms, including bacteria, whose genetic diversity is a critical component of our ecosystem. This genetic variability gains more value when properly identified, classified and documented. Therefore, discovering the bacterial diversity, analysing its geographic distribution, understanding its role in the ecosystem, and monitoring isolated cases and outbreaks via epidemiological and health surveillance is important^1^.

Bacterial resistance to antimicrobials is a public health problem with global relevance. Bacteria previously susceptible to commonly used antibiotics no longer respond to these agents and are responsible for serious clinical and economic consequences related to increased morbidity and mortality. Over time, with each new generation of antimicrobials used in clinical practice, bacteria rapidly evolve and acquire resistance, thus becoming multidrug resistant (MDR)^2^.

The main factor responsible for the increase in antimicrobial resistance (AMR) rates is the uncontrolled use of antimicrobial agents for human health, livestock, and agriculture activities. In addition to causing serious illnesses in humans, MDR bacteria are also associated with infections/colonization in food-producing animals and pets, thereby substantially affecting public health worldwide. Therefore, this phenomenon leads to an increase in morbidity and mortality rates, prolonged hospital stays, and increased health care costs^3–5^. A survey conducted by the GBD 2019 Antimicrobial Resistance Collaborators estimated that 13.7 million deaths were related to infections, and there were 7.7 million deaths associated with 33 bacterial pathogens^6^. According to an estimate from the World Bank, low- and middle-income countries such as Brazil are at risk of losing up to 4.4% of their gross domestic product (GDP) by 2050 because of AMR, and such a phenomenon also impacts high-income countries^7^.

To combat AMR in the United States of America (USA), in 2016, the Center for Disease Control and Prevention (CDC) established an Antimicrobial Resistance Laboratory Network (AR Lab Network) aimed at the rapid detection of AMR pathogens and the prevention of intra- and interhospital spread of these pathogens^8^. Years later, in 2020, the French government adopted a One Health approach and requested a nationwide report about the current epidemiological status of AMR to recommend future actions to mitigate AMR in the environment^9^.

Similar to the USA, Brazil, starting in 2015, began implementing the Microbial Resistance Analytical Subnet in Health Services, and in 2018, based on the Global Antimicrobial Resistance Surveillance System (GLASS), Brazil began its own national antimicrobial resistance surveillance program (BR-GLASS); however, the first national results regarding the antimicrobial resistance profile, determined via a molecular epidemiology approach, was obtained in 2023 and contributed to the first epidemiological bulletin in 2024^2,10^.

Little is known about the biodiversity of resistant bacteria in Brazil. Thus, in this work, we aimed to map the biodiversity of carbapenem-resistant bacteria by specifically focusing on the molecular epidemiology and analysis of carbapenemase-encoding genes. Such insights are crucial for refining therapeutic and preventive strategies related to AMR and reducing the spread of MDR bacteria.

## Results

### Species biodiversity

During the period studied, the GAL recorded a total of 15,025 laboratory reports that included 22,946 antimicrobial sensitivity tests (ASTs). Based on the ASTs for meropenem, 9126 tests were performed; in other words, we estimated that there were 9126 bacterial isolates.

A total of 163 different bacterial species were identified over 4 years of research, among which 110/163 (67.48%) had AST profiles for ertapenem, meropenem and imipenem (Table 1). Among them, 72/110 (65.45%) species showed resistance to carbapenems (based on phenotypic tests). Among these resistant species, 25/72 species (34.72%) carried at least one gene encoding a carbapenemase (genotypic tests).

**Table 1:** Biodiversity of bacterial species in which carbapenems were tested.

Class	Family	Species	NCBI: taxid^(i)^
PHYLUM: *Proteobacteria*			
* Gammaproteobacteria*	*Enterobacteriaceae*	*Cedecea lapagei	158,823
		^#^*Citrobacter braakii*	57,706
		*Citrobacter farmeri*	67,824
		*Citrobacter freundii	546
		*Citrobacter koseri	545
		*Citrobacter spp*.*	544
		*Enterobacter aerogenes^(a)^	548
		^#^*Enterobacter asburiae*	61,645
		*Enterobacter cloacae	550
		^#^*Enterobacter sakazakii*^(b)^	28,141
		*Enterobacter spp*.*	547
		*Escherichia coli	562
		*Escherichia fergusonii*	564
		*Escherichia* spp*.*	561
		^#^*Klebsiella aerogenes*^(a)^	548
		^#^*Klebsiella oxytoca*	571
		*Klebsiella ozaenae^(c)^	574
		^#^*Klebsiella planticola*^(d)^	575
		*Klebsiella pneumoniae	573
		*Klebsiella pneumoniae subsp*. Pneumoniae*	72,407
		*Klebsiella spp*.*	570
		^#^*Kluyvera ascorbata*	51,288
		*Kluyvera* spp*.*	579
		^#^*Leclercia adecarboxylata*	83,655
		^#^*Pluralibacter gergoviae*	61,647
		*Raoultella ornithinolytica*	54,291
		^#^*Salmonella entérica*	28,901
		*Salmonella enterica* subsp*. enterica*	59,201
		^#^*Salmonella enteritidis*^(e)^	149,539
		*Salmonella* spp*.*	590
		*Shigella boydii*	621
		*Shigella flexneri*	623
		*Shigella sonnei*	624
		*Shigella* spp*.*	620
	*Moraxellaceae*	^#^*Acinetobacter anitratus*^(f)^	107,673
		*Acinetobacter baumannii	470
		*Acinetobacter calcoaceticus	471
		^#^*Acinetobacter haemolyticus*	29,430
		*Acinetobacter lwoffii	28,090
		*Acinetobacter spp*.*	469
	*Pseudomonadaceae*	*Pseudomonas aeruginosa	287
		*Pseudomonas fluorescens*	294
		*Pseudomonas luteola*	47,886
		*Pseudomonas mendocina*	300
		*Pseudomonas oryzihabitans*	47,885
		^#^*Pseudomonas pseudoalcaligenes*^(g)^	301
		^#^*Pseudomonas putida*	303
		^#^*Pseudomonas* spp*.*	286
		*Pseudomonas stutzeri*	316
	*Morganellaceae*	*Morganella morganii	582
		^#^*Morganella morganii* subsp*. morganii*	180,434
* Gammaproteobacteria*		*Morganella morganii* subsp*. Sibonii*	180,435
		^*#*^*Morganella* spp*.*	581
		^#^*Proteus mirabilis*	584
		*Proteus penneri*	102,862
		*Proteus* spp*.*	583
		^#^*Proteus vulgaris*	585
		^#^*Providencia alcalifaciens*	126,385
		*Providencia rettgeri	587
		^#^*Providencia rustigianii*	158,850
		*Providencia spp*.*	586
		*Providencia stuartii	588
	*Yersiniaceae*	^#^*Serratia ficaria*	61,651
		^#^*Serratia liquefaciens*	614
		*Serratia marcescens	615
		^#^*Serratia plymuthica*	82,996
		*Serratia rubidaea	61,652
		*Serratia spp.	613
		^#^*Yersinia intermedia*	631
	*Erwiniaceae*	*Pantoea agglomerans	549
		^#^*Pantoea* spp.	53,335
	*Aeromonadaceae*	^#^*Aeromonas caviae*	648
		^#^*Aeromonas hydrophila*	644
		*Aeromonas* spp.	642
	*Hafniaceae*	*Edwardsiella tarda*	636
		^#^*Hafnia alvei*	569
	*Shewanellaceae*	*Shewanella putrefaciens*	24
	*Xanthomonadaceae*	^#^*Stenotrophomonas maltophilia*	40,324
* Betaproteobacteria*	*Alcaligenaceae*	*Achromobacter denitrificans*	32,002
		*Achromobacter* spp.	134,375
		*Achromobacter xylosoxidans*	85,698
		^#^*Alcaligenes faecalis*	511
	*Burkholderiaceae*	^#^*Burkholderia cenocepacia*	95,486
		^#^*Burkholderia cepacia*	292
		^#^ *Burkholderia cepacia* Complex	87,882
		^#^*Burkholderia gladioli*	28,095
		^#^*Burkholderia* spp.	32,008
		*Ralstonia pickettii*	329
	*Chromobacteriaceae*	*Chromobacterium violaceum*	536
	*Comamonadaceae*	^#^*Delftia acidovorans*	80,866
	*Neisseriaceae*	^#^*Kingella denitrificans*	502
		*Neisseria meningitidis*	487
* Alphaproteobacteria*	*Brucellaceae*	*Ochrobactrum anthropi*^(h)^	529
	*Sphingomonadaceae*	^#^*Sphingomonas paucimobilis*	13,689
PHYLUM: *Bacteroidetes*			
* Flavobacteriia*	*Weeksellaceae*	^#^*Chryseobacterium indologenes*	253
		^#^*Elizabethkingia meningoseptica*	238
* Sphingobacteriia*	*Sphingobacteriaceae*	^#^*Sphingobacterium* spp.	28,453
		^#^*Sphingobacterium spiritivorum*	258
PHYLUM: *Bacillota* (*Firmicutes*)			
* Bacilli*	*Enterococcaceae*	*Enterococcus faecalis*	1351
		*Enterococcus faecium*	1352
		*Enterococcus* spp.	1350
PHYLUM: *Proteobacteria*			
	*Staphylococcaceae*	^#^*Staphylococcus aureus*	1280
		#*Staphylococcus cohnii* subsp.* cohnii*	74,704
		^#^*Staphylococcus epidermidis*	1282
		^#^*Staphylococcus haemolyticus*	1283
		^#^*Staphylococcus schleiferi*	1295
	*Streptococcaceae*	*Streptococcus agalactiae*	1311
		*Streptococcus mitis*	28,037
		*Streptococcus pneumoniae*	1313
		*Streptococcus pyogenes*	1314

^(a)^Current name: *Klebsiella aerogenes.*

^(b)^Current name: *Cronobacter sakazakii.*

^(c)^Current name: *Klebsiella pneumoniae* subsp.* ozaenae.*

^(d)^Current name: *Raoultella planticola.*

^(e)^Current name: *Salmonella enterica* subsp*. enterica* serovar Enteritidis.

^(f)^Current name: *Acinetobacter calcoaceticus* subsp*. anitratus.*

^(g)^Current name: *Pseudomonas oleovorans.*

^(h)^Current name: *Brucella anthropic.*

*Resistant to carbapenems—ARG detected.

^#^Resistant to carbapenems—Phenotypic test.

Fewer different bacterial species were identified after the start of the COVID-19 pandemic. In the first two years of this research, 2018 and 2019, 63 species were identified in each period. The number of identified species decreased to 50 in 2020 and 44 in 2021, representing a decrease of − 30.16%.

The five most frequent species belonged to the class Gammaproteobacteria and the Enterobacterales order, followed by the Moraxellaceae and Pseudomonadaceae families, which accounted for 79.99% of the ASTs (n = 18,354/22,946). Overall, the carbapenem resistance rate was 30.61%, with imipenem being slightly more active (34.99%) than meropenem and ertapenem. However, the percentage of isolates resistant to these agents increased from the beginning of the research period to the end of the study period; that of ertapenem increased by 165.39%, while that of meropenem and imipenem increased by 58.56% and 40.65%, respectively (Table 2).

**Table 2:** Number of different bacterial species distributed according to susceptibility to carbapenems between 2018 and 2021 in the state of Rondônia.

	Nº species	Species	Average rate of resistance	Percentage increase in resistance
Total	163	100.0%	41.53%^(a)^	5.28%
Tested for carbapenems	110	67.48%	30.61%^(b)^	69.69%
Ertapenem	77	–	27.44%^(c)^	165.39%
Imipenem	94	–	34.99%^(d)^	40.65%
Meropenem	103	–	31.28%^(e)^	58.56%
Showed resistance	72	65.45%	–	

^(a)^Overall percentage of isolates with a profile of resistance to all classes of antimicrobials at LACEN/RO (73,069 resistant AST/175,936 total AST).

^(b)^Overall percentage of isolates with a profile of resistance to carbapenems at LACEN/RO (7251 resistant ASTs/22,946 total ASTs).

^(c)^Overall percentage of isolates with a profile of resistance to ertapenem at LACEN/RO (1599 resistant ASTs/5827 total ASTs).

^(d)^Overall percentage of isolates with a profile of resistance to imipenem at LACEN/RO (2797 resistant ASTs/7993 total ASTs).

^(e)^Overall percentage of isolates with with a meropenem resistance profile at LACEN/RO (2855 resistant ASTs/9126 total ASTs).

The species of gram-negative bacilli (GNB) that showed the highest rates of resistance to carbapenems were *Acinetobacter baumannii* (1581 bacterial isolates; 95% CI 70.46–73.58%), followed by *Klebsiella pneumoniae* (2104 bacterial isolates; 95% CI 37.38–39.82%), *Pseudomonas aeruginosa* (1065 bacterial isolates; 95% CI 31.68–35.72%) and *Enterobacter cloacae* (330 bacterial isolates; 95% CI 18.32–23.50%).

### Molecular epidemiology

Genes encoding carbapenemases were detected in 25 carbapenem-resistant species, with *A. baumannii* having the highest number of detected genes (n = 625), followed by *K. pneumoniae* (n = 281), *P. aeruginosa* (n = 28) and *E. coli* (n = 23) (Table 3).

**Table 3:** Description of the 25 species for which genes encoding carbapenemases were detected according to molecular class (A–D).

Bacteria		Acquired Carbapenemase-encoding genes		
Genus	Species	Molecular Class A	Molecular Class B	Molecular Class D
*Acinetobacter* (n = 1677)				
	*A. baumannii* (n = 1558)	*bla*_KPC_-like (n = 1)	*bla*_NDM_-like (n = 4)	*bla*_OXA-23_-like (n = 470)
				*bla*_OXA-24/40_-like (n = 2)
				*bla*_OXA-48_-like (n = 3)
				*bla*_OXA-58_-like (n = 119)
				*bla*_OXA-143_-like (n = 26)
	*A. calcoaceticus* (n = 4)	–	–	*bla*_OXA-58_-like (n = 1)
	*A. lwoffii*^*a*^ (n = 6)	–	*bla*_NDM_-like (n = 1)	*bla*_OXA-58_-like (n = 1)
	*Acinetobacter* spp. (n = 109)	–	–	*bla*_OXA-23_-like (n = 20)
				*bla*_OXA-58_-like (n = 5)
				*bla*_OXA-143_-like (n = 1)
*Cedecea* (n = 1)				
	*C. lapagei* (n = 1)	–	*bla*_NDM_-like (n = 1)	–
*Citrobacter* (n = 78)				
	*C. freundii* (n = 38)	*bla*_KPC_-like (n = 2)	–	–
	*C. koseri* (n = 35)	*bla*_KPC_-like (n = 1)	–	–
	*Citrobacter* spp.^a^ (n = 5)	*bla*_KPC_-like (n = 5)	–	–
*Enterobacter* (n = 385)				
	*E. aerogenes*^*b*^ (n = 59)	*bla*_KPC_-like (n = 1)	–	–
	*E. cloacae* (n = 316)	*bla*_KPC_-like (n = 7)	*bla*_NDM_-like (n = 1)	–
	*Enterobacter* spp.^a^ (n = 10)	*bla*_KPC_-like (n = 8)	*bla*_NDM_-like (n = 1)	–
			*bla*_*VIM*_-like (n = 1)	
*Escherichia* (n = 2000)				
	*E. coli* (n = 2000)	*bla*_KPC_-like (n = 21)	*bla*_NDM_-like (n = 1)	*bla*_OXA-48_-like (n = 1)
*Klebsiella* (n = 2231)				
	*K. pneumoniae* (n = 2042)	*bla*_KPC_-like (n = 269)	*bla*_NDM_-like (n = 2)	*bla*_OXA-48_-like (n = 9)
			*bla*_IMP_-like (n = 1)	
	*K. ozaenae*^*c*^ (n = 21)	*bla*_KPC_-like (n = 2)	–	–
	*K. pneumoniae* subsp. *pneumoniae* (n = 67)	*bla*_KPC_-like (n = 3)	–	–
*Klebsiella* spp. (n = 101)	*bla*_KPC_-like (n = 10)	–	–	
*Morganella* (n = 77)				
	*M. morganii* (n = 77)	*bla*_KPC_-like (n = 2)	–	*bla*_OXA-48_-like (n = 2)
*Pantoea* (n = 15)				
	*P. agglomerans* (n = 15)	–	*bla*_NDM_-like (n = 1)	–
*Providencia* (n = 79)				
	*P. rettgeri* (n = 9)	–	–	*bla*_OXA-48_-like (n = 1)
	*P. stuartii* (n = 67)	*bla*_KPC_-like (n = 1)	*bla*_NDM_-like (n = 5)	–
	*Providencia* spp.^a^ (n = 3)	–	*bla*_NDM_-like (n = 1)	*bla*_OXA-48_-like (n = 1)
			*bla*_*VIM*_-like (n = 1)	
*Pseudomonas* (n = 1038)				
	*P. aeruginosa* (n = 1038)	*bla*_KPC_-like (n = 2)	*bla*_SPM_-like (n = 20)	*bla*_OXA-48_-like (n = 4)
			*bla*_VIM_-like (n = 2)	
			*bla*_VIM_-like (n = 3)	
*Serratia* (n = 294)				
	*S. marcescens* (n = 241)	*bla*_KPC_-like (n = 7)	–	–
	*S. rubidaea* (n = 4)	*bla*_KPC_-like (n = 1)	–	–
	*Serratia* spp. (n = 49)	*bla*_KPC_-like (n = 1)	–	–

The (n) values of the species are averages that were calculated based on the ASTs with carbapenem resistance profiles.

^a^Data taken from internal laboratory records.

^b^Species registered in laboratory reports as *E. aerogenes*, but the current name is *K. aerogenes.*

^c^Species registered in laboratory reports as *K. ozaenae*, but the current name is *K. pneumoniae* subsp. *ozaenae.*

**Figure 1:**
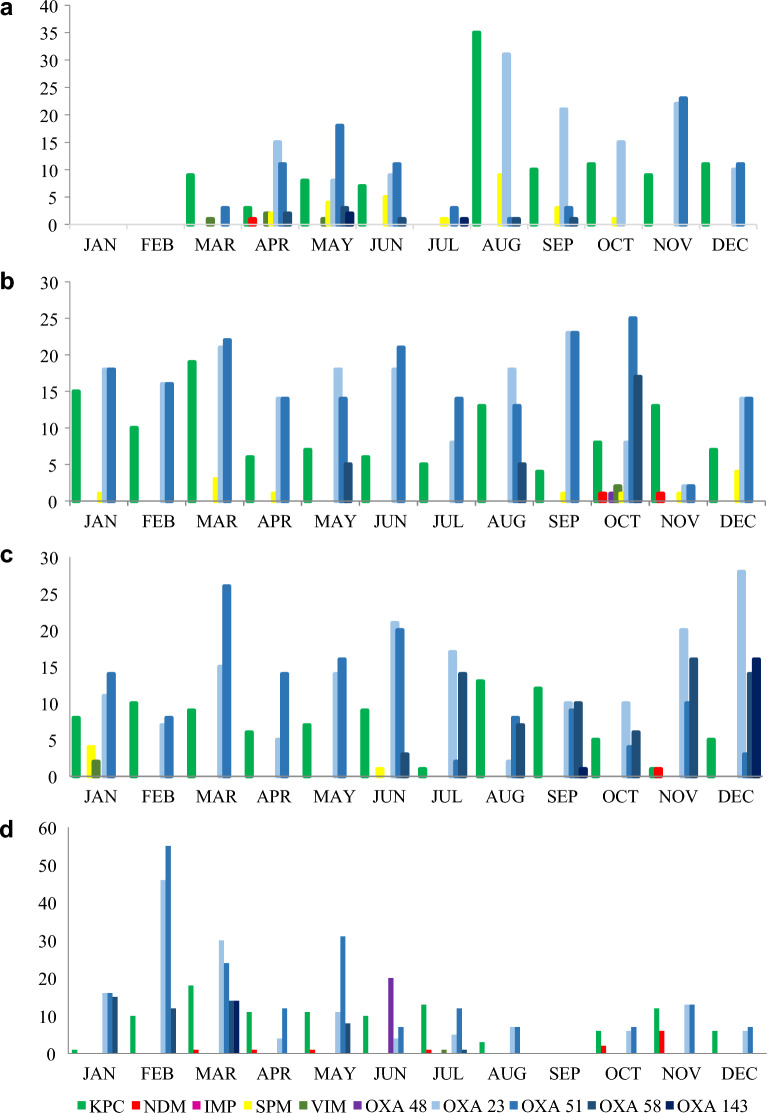
Temporal distribution of genes encoding detected carbapenemases in (**a**) 2018, (**b**) 2019, (**c**) 2020, and (**d**) 2021. The ARGs *bla*KPC and *bla*OXA-23 were endemic during the analysed period; in 2020, the level of *bla*SPM regressed, and in 2021, the presence of *bla*NDM in the region was revealed. Greater ARG diversity was observed in the last quarter of each year from 2019 onwards, and a possible *bla*OXA48 outbreak occurred in June 2021.

Among the *A. baumannii* isolates, the bla_OXA-23_-like gene was the most common (n = 470), followed by bla_OXA-58_-like (n = 119) and bla_OXA-143_-like (n = 26). Although a single isolate carrying the bla_OXA-143_-like type was detected in 2018, a subsequent increase in this ARG (24%) was observed in 2021. Similarly, the bla_OXA-58_-like gene exhibited a 25% increase in prevalence in the same year (Fig. 1). Furthermore, bla_KPC_-like was detected in a single isolate recovered in 2020. Screening for bla_NDM_-like among isolates of the genus Acinetobacter began only in 2021, when 4 bla_OXA-23_-like genes were detected.

In *K. pneumoniae*, the bla_KPC_-like gene encoded most carbapenemases (n = 269). Sporadic cases of bla_NDM_-like were observed in 2019 and 2021, as well as bla_IMP_-like, which was detected in one isolate in 2021, and 4 bla_OXA-48_-like genes were detected in the same year (Fig. 1).

bla_SPM_-like was the most frequent gene encoding carbapenemases that was detected in *P. aeruginosa*, with a decrease to a prevalence of 86% observed over the years. As observed for *K. pneumoniae* and *A. baumannii*, bla_OXA-48_-like was detected among *P. aeruginosa* isolates mainly in 2021. bla_VIM_-like isolates were detected in 2018 and 2020, while bla_KPC_-like isolates were detected in 2020 and 2021 (Fig. 1).

For *E. coli*, the predominant ARG was the bla_KPC_-like type, with the highest frequency occurring in 2019 (n = 13). In 2021, bla_NDM_-like and bla_OXA-48_-like ARGs were detected.

ARGs were detected at lower frequencies in other species (n < 10), such as bla_KPC_-like genes detected in *Citrobacter freundii*, *Citrobacter koseri*, *Klebsiella aerogenes*, *Enterobacter cloacae*, *Klebsiella ozaenae*, *Klebsiella* spp., *Morganella morganii*, *Providencia stuartii*, *Serratia marcescens*, *Serratia rubidaea* and *Serratia* spp. Similarly, bla_NDM_-like ARGs were detected in *Cedecea lapagei*, *E. cloacae*, *Pantoea agglomerans* and *P. stuartii*. Furthermore, bla_OXA-48_-like ARGs were detected in *M. morganii* and *Providencia rettgeri* (Table 3).

An increase in the number of ARGs detected over time was observed based on laboratory records: 2018 (n = 186), 2019 (n = 229), 2020 (n = 180) and 2021 (n = 448). Therefore, the percent of isolates with ARGs increased by 140.86% from the beginning of the research period to the end date. Genetic variation among these ARGs was detected mainly at the end of 2019 and reappeared throughout 2021, with a distribution of bla_NDM_-like throughout the months and an evident peak of bla_OXA-48_-like in June of 2021 (Fig. 1).

## Discussion

This study revealed a significant increase in carbapenem resistance (69.69%) and ARG detection (140.86%) among bacterial species in Rondônia, highlighting the growing challenge of AMR in Brazil. Importantly, this research also revealed a reduction (− 30.16%) in bacterial biodiversity in the state. Notably, this is the first time that data on carbapenem-resistant bacterial biodiversity in clinical samples have been revealed in the state of Rondônia.

Extraordinarily, our study demonstrated a 407.39% increase in resistance to meropenem in *E. coli* and a 108.73% increase in *Klebsiella pneumoniae,* a smaller increase of 5.53% with a tendency to plateau in *A. baumannii*, and a − 27.93% decrease in *P. aeruginosa* during the analysed period.

Although the increase in resistance in *A. baumannii* was not as high as that in *E. coli* and *K. pneumoniae*, this bacterium already has intrinsic resistance to ertapenem and a high rate of resistance to meropenem and imipenem, as demonstrated by studies in Rondônia where the resistance to carbapenem was 76.2%^11^, 4.91% higher than what we found in our study; IC1 was the most frequent clonal group, followed by IC5 and IC4^12^. Furthermore, the greatest ARG plasticity was detected in *A. baumannii*, which presented with *bla*_KPC_-like or *bla*_NDM_-like together with *bla*_OXA23_-like and *bla*_OXA58_-like or even *bla*_OXA23_-like together with *bla*_OXA143_-like.

Notably, unlike *A. baumannii*, *P. aeruginosa* lost ARG diversity over the years, which may explain the reduction in resistance to carbapenems; however, importantly, other resistance mechanisms that were not tested in the laboratory may be active; for example, the production of *AmpC*^13–17^. Like other phyla and classes (Table 1), *P. aeruginosa* had a carbapenem resistance phenotype, but these isolates did not express the genes investigated in this study. These isolates could have other resistance mechanisms that were not addressed in this study.

The five species with the highest number of isolates analysed in the present study are classified as priority pathogens by the WHO^18^. At least nine species resistant to carbapenems and carrying ARGs in the present study were not reported in epidemiological bulletin/health reports by Brazilian public health bodies, such as the National Health Surveillance Agency (ANVISA) and the Oswaldo Cruz Foundation (FIOCRUZ). There were also no reports from international bodies such as the Global Antimicrobial Use and Resistance Surveillance System (GLASS)^19^, the Latin American Antimicrobial Resistance Surveillance Network (ReLAVRA) or the Health Information Platform for the Americas (PLISA).

Until the end of the study period (2021), Brazil did not have compiled data on bacterial biodiversity and gene-mediated antimicrobial resistance, which makes it difficult to compare state results at the national level. ANVISA publishes the Bulletin on Patient Safety and Quality in Health Services and Microbial Resistance; however, data on bacterial diversity and microbial resistance are limited and fragmented by the type of intensive care unit (ICU) and by the type of infection with national indicators of health care-associated infections (HAIs)^10^. Although the Bulletin mentions microbial resistance, the data are not presented in a nationally compiled form, nor is the genetic variability of resistance. For example, nationwide, in the adult ICU for primary bloodstream infection (IPCSL), only 8 g-negative species were identified. When observing other Brazilian states, including Rondônia, the results are the same in terms of the number of species, only varying in the percentage of resistance. Only in 2018 did Brazil present the PAN-BR (2018–2022), which is aligned with the five strategic objectives of the Global Action Plan, which is now part of the One Health approach that includes several government bodies; however, there is no national data on microbial resistance, and the inefficiency or lack of infection prevention and control programs was mentioned in the presentation^20^.

With the implementation of the Brazilian Antimicrobial Resistance Surveillance System (BR-GLASS)^10^, we obtained the first results in 2021, but we were limited to 3 hospitals in the state of Paraná; the data were analysed for 13 months (December 2017–December 2018). Notably, in this study, overall meropenem resistance was detected (17.1–21.7% resistance in all patients vs. hospitalized patients) in 11,347 isolates. In our study, we observed an average rate of resistance to this antimicrobial of 31.28%, with a considerable increase to 58.56% in 9,126 isolates over the course of the study. With respect to *E. coli* resistance to meropenem, a resistance rate of 3.2% was observed, and in our study, an average resistance rate of 9.99% and a notable percentage of 407.39% were detected. A low percentage of resistance to meropenem was reported for *Klebsiella pneumoniae* (38.5%), and we observed that this species had an average resistance rate of 37.55% for meropenem, with a significant increase of 108.73% (in the period analysed). *Acinetobacter baumannii* was revealed to have a high meropenem resistance rate of 81.4% by BR-GLASS, and our study revealed a resistance rate of 71.41%, with a low increase of 5.53% during the research period. A 29.3% resistance rate for *Pseudomonas aeruginosa* was detected, and a 29.95% rate of resistance to meropenem was detected in this study; however, the rate of resistance decreased by 27.93% over the analysed period.

After 5 years of BR-GLASS implementation, data related to national ARG detection rates were revealed for the first time^2^ to the scientific community; one year later, this data contributed to the preparation of the first national epidemiological bulletin addressing the molecular epidemiology of microorganisms resistant to carbapenems. In the BR-GLASS study, the researchers reported a − 4% reduction in *bla*_KPC_ and an increase of 41.1% in *bla*_NDM_ among Enterobacterales^2^; in our study, we observed a 56.34% increase in *bla*_KPC_ detection among species of this order, and *bla*_NDM_ was detected once in 2019 and 2020, with an increase to 11 cases in 2021. Both studies confirmed the reduction in *bla*_SPM_ in *P. aeruginosa* over this period. In relation to the ARGs *bla*_OXA23_ and *bla*_OXA58_ in *A. baumannii* at the national level, the level of the first remained stationary, and the level of the second increased; in the state of Rondônia, the level of both increased. As the study parameters are different, distinguishing which Enterobacterales specimens had *bla*_KPC_ and *bla*_NDM_ is not possible. In our study, there were few isolates when compared to that at the national level. However, the relevance of cataloguing species when mobile resistance genes are detected, even at low numbers or in few isolates, is important. This reporting is fundamental for epidemiological and ecological studies since each species may present with different virulence and pathogenicity characteristics. *Pantoea agglomerans, Morganella morganii,* and *Providencia stuartii* and specimens of *Citrobacter* spp., *Enterobacter* spp. and *Serratia* spp. were found to carry ARGs in our study in Rondônia, but these details were not described by the BR-GLASS study.

The increase in ARG detection in the last year analysed (2021) is due to the increase in the number of hospitalizations as a result of the COVID-19 pandemic, which increased the demand for antimicrobial agents and laboratory tests. Importantly, in the case of critical GNB pathogens that carry a variety of genes encoding carbapenemases, there is concern that these resistance mechanisms are shared with other bacterial species. Plasmids mediate the horizontal transmission of ARGs between bacteria, thus facilitating their adaptation to multiple environmental conditions and contributing to the global spread of AMR^21,22^. Some of these ARGs, such as those encoding carbapenemases, are carried on conjugative plasmids that spread through high-risk epidemic clones^23^.

The reduction in biodiversity may be related to the COVID-19 pandemic since all services were focused on hospitalizations due to the pandemic. Therefore, the most common species at the end of the period were identified and reported by health services. Another explanation is the selective pressure of these ARGs on bacterial communities and the probability of plasmid persistence increasing the number of bacterial strains in the community, as the strains become less dependent on a high conjugation rate^23^. According to multilevel ecological dynamics, fitness differences are limited not only to plasmid-free and plasmid-bearing cells within single-strain populations but also different strains. Therefore, the loss of a plasmid can occur due to the low intrinsic fitness of its hosts or even because of ecological drift, and the benefits of plasmid fitness at the phylotype level significantly facilitate persistence at the community level^24^.

Notably, hospital-associated bacteria, even before being popularized, were already part of a natural and balanced ecosystem^25^. However, human action contributes to the import/export of environmental and nosocomial/resistant bacterial species in different habitats, including polar ecosystems^26,27^. Similar to the previous example, the same import/export occurs in the Amazon region, when areas are opened and explored. Bacteria previously commonly found in soils, rivers and wildlife have a high capacity to acquire mechanisms of AMR^3,5,28,29^. Recent studies with mosquitoes in the Amazon, which are normally associated with arboviruses, malaria, and filarial worms, revealed diverse bacteria of clinical interest in their faeces and salivary glands^30–32^. Another important point is the climate of the Amazon, where different forest/soil extracts suffer floods and droughts due to the climate, which alters the diversity of bacterial communities, as demonstrated in a study in Rondônia^33^.

Due to Brazil's vast biodiversity, antimicrobial-resistant bacteria can circulate between humans and animals through different routes, including through food, water and the environment. The transmission of these bacteria is influenced by globalization, that is, by trade, travel and human and animal migration. A multisectoral approach to combating AMR may be more effective than actions focused exclusively on human health. For example, monitoring bacterial biodiversity and antimicrobial-resistant species in the context of One Health has become a priority for identifying new species utilizing this strategy^9,34^.

This study had several limitations. The data used in this study came from laboratory test results/reports, with no possibility of forwarding the strains for sequencing at national reference centres for further analysis. Furthermore, LACEN/RO, during the research period, did not have a collection of microorganisms that was stored for future postdiagnosis analyses. For the validation of future strains in similar research, the strains should be sent to a national resistance reference centre for sequencing, the identification of rare bacteria, or loco sequencing. Another limitation was the scarcity of resources for the molecular identification of ARGs during the public health crisis caused by *SARS-CoV-2* infection, where attention was focused exclusively on diagnosing the virus.

Our study revealed that the bacterial biodiversity is broader than that reported for Epidemiological Bulletins and that genes encoding proteins related to carbapenem resistance are present and shared among different taxonomic classes. This carbapenem-resistant biodiversity represents a growing public health challenge. State epidemiological surveillance should include ARG data in reports, as well as in epidemiological bulletins. Monitoring the spread of these bacteria and developing control strategies is essential. New research is needed to better understand resistance mechanisms and develop new therapeutic options. The One Health perspective is important because of the contamination of water and food by hospital and residential sewage. With these results, we hope to provide molecular epidemiological data for the state surveillance of bacterial resistance in Rondônia and assist in making public policy decisions.

## Methods

### Bacterial isolates

The methodology used in this work consisted of compiling the results of clinical microbiological laboratory tests and molecular tests to search for resistance genes; this study was performed at the Rondônia Central Public Health Laboratory (LACEN/RO) between 2018 and 2021.

For information, LACEN/RO is a state reference laboratory of medium to high complexity in diagnostics that is part of the National System of Public Health Laboratories in Brazil (Sislab).

The identification of bacterial species was carried out by the Clinical Microbiology Laboratory at LACEN/RO through cultures of several clinical samples and surveillance swabs. In addition to culture media, until 2019, bacteria were identified and tested for sensitivity to antimicrobials by automation using Vitek^®^ 2 Biomerieux; BD PhoenixTM automation is currently used. Biochemical tests, diffusion disk assays, antibiotic concentration gradient strips (Etest type), and broth microdilution assays, among other tests, are routinely used in the laboratory according to bacterial species and in accordance with the Clinical and Laboratory Standards Institute (CLSI) and from 2022 according to the Brazilian Antimicrobial Susceptibility Testing Committee (BRCAST). The tests that were performed, in addition to automation, vary according to economic factors related to the purchasing power of the unit's inputs.

The following descriptions were used to identify carbapenem-resistant species in the GAL system using the following route: Medical Biology/Reports/Specifics/Resistance/Microbial Resistance. During the period studied, the GAL recorded a total of 15,025 laboratory reports. As a criterion for our search, only AST profiles that presented resistance to ertapenem, meropenem and imipenem were included. The data obtained were plotted in electronic spreadsheets organized by phylum, class, family and species.

The data were classified in order of the highest number of ASTs to determine which species were most frequently resistant to carbapenems in laboratory tests. The 95% confidence intervals (95% CIs) were created based on the standard deviation of the proportion of resistant isolates belonging to each of the five most common species.

### Molecular characterization of carbapenemase-encoding genes

Molecular characterization was carried out by the Molecular Microbiology Laboratory at LACEN/RO, which receives all bacterial isolates with phenotypic characteristics of resistance to carbapenems.

The extraction of genetic material was carried out using thermal lysis or commercial kits. Amplification was performed using conventional PCR followed by electrophoresis. The identification of genes encoding carbapenemases was performed based on the bacterial isolate.

The results of laboratory tests to search for resistance genes were obtained using the GAL database and laboratory records. In GAL analysis, the following electronic route was chosen for the ARG search period: Medical Biology/Reports/Specifics/Resistance/Report.

We did not to use the epidemiological bulletin Disease/Clinical Data because it was limited to a 90-day research period. For this reason, we also analysed laboratory reports to determine when a bacterial isolate had more than one ARG in the same strain (Supplementary information).

A total of 8296 molecular tests from the GAL database were evaluated. The PCR protocols used were in accordance with the ARG investigated. The genes encoding carbapenemases selected were: *bla*_KPC_-like (class A); *bla*_NDM_-like, *bla*_IMP_-like, *bla*_VIM_-like, *bla*_SPM_-like (class B); and *bla*_OXA-23_-like, *bla*_OXA-48_-like, *bla*_OXA-51_-like, *bla*_OXA-58_-like, and *bla*_OXA-143_-like (class D)^35–38^.

### Ethical approval

The study was approved by the Research Ethics Committees of NUSAU/UNIR and by the National Ethics Committee in Research (CONEP) under registration number 5.316.814. The study was also registered in the National System for the Management of Genetic Heritage and Associated Knowledge (SisGen) under the registration number AE18E21.

### Transparency declarations

A.C.G. has recently received research funding and/or consultation fees from bioMérieux Eurofarma, MSD, Pfizer, Sandoz, United Medical, and Zambon. The other authors have nothing to declare. This study was not financially supported by any diagnostic/pharmaceutical company.

### Supplementary Information

Supplementary Information 1.

Supplementary Information 2.

Supplementary Information 3.

Supplementary Information 4.

Supplementary Information 5.

Supplementary Information 6.

Supplementary Information 7.

## Data Availability

The datasets generated and/or analysed during the current study are not publicly available due to restricted access to public health laboratory data and information security policies but are available from the corresponding author upon reasonable request.
