# Supplementary material for: Biodiversity of carbapenem-resistant bacteria in clinical samples from the Southwest Amazon region (Rondônia/Brazil)
Source: Sci Rep. 2024 Apr 23;14:9383. doi: 10.1038/s41598-024-59733-w (PMC11039742; doi:10.1038/s41598-024-59733-w)
Supplement: Supplementary file 1 — Supplementary Information 1. [file 41598_2024_59733_MOESM1_ESM.pdf]

Relatório Pesquisa de Genes de Resistência

Data Início: 01/01/2018 **Data Fim:** 31/12/2018

Qtd. de Exame/Metodologia: 275 **Qtd. de Microrganismo/Gene Pesq.:** 57

Todos os Laboratórios

Microrganismo /Gene Pesquisado	Jan/2018	Fev/2018	Mar/2018	Abr/2018	Maio/2018	Jun/2018	Jul/2018	Ago/2018	Set/2018	Out/2018	Nov/2018	Dez/2018	Total
**Acinetobacter baumannii/bla IMP**													
Não Detectável	0	0	0	5	0	0	0	0	0	0	0	0	5
**Subtotal**	**0**	**0**	**0**	**5**	**0**	**0**	**0**	**0**	**0**	**0**	**0**	**0**	**5**
**Acinetobacter baumannii/bla KPC**													
Não Detectável	0	0	0	5	0	0	0	0	0	0	0	0	5
**Subtotal**	**0**	**0**	**0**	**5**	**0**	**0**	**0**	**0**	**0**	**0**	**0**	**0**	**10**
**Acinetobacter baumannii/bla OXA-143**													
Detectável	0	0	0	0	1	0	0	0	0	0	0	0	1
Não Detectável	0	0	0	5	0	0	0	0	0	0	0	0	5
**Subtotal**	**0**	**0**	**0**	**5**	**1**	**0**	**0**	**0**	**0**	**0**	**0**	**0**	**16**
**Acinetobacter baumannii/bla OXA-23**													
Detectável	0	0	0	5	2	12	0	17	19	8	0	29	92
Não Detectável	0	0	0	0	0	1	0	8	5	3	0	5	22
**Subtotal**	**0**	**0**	**0**	**5**	**2**	**13**	**0**	**25**	**24**	**11**	**0**	**34**	**130**
**Acinetobacter baumannii/bla OXA-24**													
Não Detectável	0	0	0	5	0	0	0	0	0	0	0	0	5
**Subtotal**	**0**	**0**	**0**	**5**	**0**	**0**	**0**	**0**	**0**	**0**	**0**	**0**	**135**
**Acinetobacter baumannii/bla OXA-48**													
Não Detectável	0	0	0	5	0	0	0	4	0	0	0	0	9
**Subtotal**	**0**	**0**	**0**	**5**	**0**	**0**	**0**	**4**	**0**	**0**	**0**	**0**	**144**
**Acinetobacter baumannii/bla OXA-51**													
Detectável	0	0	0	5	0	14	0	5	2	0	0	20	46
Não Detectável	0	0	0	0	0	1	0	20	21	11	0	5	58
**Subtotal**	**0**	**0**	**0**	**5**	**0**	**15**	**0**	**25**	**23**	**11**	**0**	**25**	**248**
**Acinetobacter baumannii/bla OXA-58**													
Detectável	0	0	0	0	0	1	0	1	0	0	0	1	3

Relatório Pesquisa de Genes de Resistência

Microrganismo /Gene Pesquisado	Jan/2018	Fev/2018	Mar/2018	Abr/2018	Maio/2018	Jun/2018	Jul/2018	Ago/2018	Set/2018	Out/2018	Nov/2018	Dez/2018	Total
Não Detectável	0	0	0	5	0	1	0	10	0	0	0	0	16
**Subtotal**	**0**	**0**	**0**	**5**	**0**	**2**	**0**	**11**	**0**	**0**	**0**	**1**	**267**
**Acinetobacter baumannii/bla SPM**													
Não Detectável	0	0	0	5	0	0	0	0	0	0	0	0	5
**Subtotal**	**0**	**0**	**0**	**5**	**0**	**0**	**0**	**0**	**0**	**0**	**0**	**0**	**272**
**Acinetobacter baumannii/SPM**													
Não Detectável	0	0	0	0	0	0	0	0	0	0	0	1	1
**Subtotal**	**0**	**0**	**0**	**0**	**0**	**0**	**0**	**0**	**0**	**0**	**0**	**1**	**273**
**Acinetobacter sp./bla OXA-23**													
Detectável	0	0	0	0	0	0	0	0	1	3	0	0	4
Não Detectável	0	0	0	0	0	0	0	0	0	0	0	1	1
**Subtotal**	**0**	**0**	**0**	**0**	**0**	**0**	**0**	**0**	**1**	**3**	**0**	**1**	**278**
**Acinetobacter sp./bla OXA-51**													
Não Detectável	0	0	0	0	0	0	0	0	0	3	0	1	4
**Subtotal**	**0**	**0**	**0**	**0**	**0**	**0**	**0**	**0**	**0**	**3**	**0**	**1**	**282**
**Citrobacter freundii/bla IMP**													
Não Detectável	0	0	0	0	0	0	0	0	0	0	0	1	1
**Subtotal**	**0**	**0**	**0**	**0**	**0**	**0**	**0**	**0**	**0**	**0**	**0**	**1**	**283**
**Citrobacter freundii/bla KPC**													
Detectável	0	0	0	0	0	0	0	0	1	0	0	1	2
**Subtotal**	**0**	**0**	**0**	**0**	**0**	**0**	**0**	**0**	**1**	**0**	**0**	**1**	**285**
**Citrobacter freundii/bla NDM**													
Não Detectável	0	0	0	0	0	0	0	0	0	0	0	1	1
**Subtotal**	**0**	**0**	**0**	**0**	**0**	**0**	**0**	**0**	**0**	**0**	**0**	**1**	**286**
**Enterobacter aerogenes/bla KPC**													
Detectável	0	0	0	0	0	0	0	0	1	0	0	0	1
**Subtotal**	**0**	**0**	**0**	**0**	**0**	**0**	**0**	**0**	**1**	**0**	**0**	**0**	**287**
**Enterobacter cloacae/bla KPC**													
Detectável	0	0	0	0	0	0	0	1	0	0	0	0	1
Não Detectável	0	0	0	0	0	0	0	1	1	0	0	0	2
**Subtotal**	**0**	**0**	**0**	**0**	**0**	**0**	**0**	**2**	**1**	**0**	**0**	**0**	**290**
**Enterobacter cloacae/bla NDM**													
Não Detectável	0	0	0	0	0	0	0	1	0	0	0	0	1
**Subtotal**	**0**	**0**	**0**	**0**	**0**	**0**	**0**	**1**	**0**	**0**	**0**	**0**	**291**
**Escherichia coli/bla IMP**													
Não Detectável	0	0	0	0	0	0	0	0	0	0	0	2	2

Relatório Pesquisa de Genes de Resistência

Microrganismo /Gene Pesquisado	Jan/2018	Fev/2018	Mar/2018	Abr/2018	Maio/2018	Jun/2018	Jul/2018	Ago/2018	Set/2018	Out/2018	Nov/2018	Dez/2018	Total
**Subtotal**	**0**	**0**	**0**	**0**	**0**	**0**	**0**	**0**	**0**	**0**	**0**	**2**	**293**
**Escherichia coli/bla KPC**													
Detectável	0	0	0	0	1	1	0	0	1	0	0	2	5
Não Detectável	0	0	0	0	0	0	0	0	0	2	0	0	2
**Subtotal**	**0**	**0**	**0**	**0**	**1**	**1**	**0**	**0**	**1**	**2**	**0**	**2**	**300**
**Escherichia coli/bla NDM**													
Não Detectável	0	0	0	0	0	0	0	0	0	2	0	2	4
**Subtotal**	**0**	**0**	**0**	**0**	**0**	**0**	**0**	**0**	**0**	**2**	**0**	**2**	**304**
**Klebsiella oxytoca/bla IMP**													
Não Detectável	0	0	0	0	0	0	0	0	0	0	0	1	1
**Subtotal**	**0**	**0**	**0**	**0**	**0**	**0**	**0**	**0**	**0**	**0**	**0**	**1**	**305**
**Klebsiella oxytoca/bla KPC**													
Não Detectável	0	0	0	0	0	0	0	0	0	1	0	1	2
**Subtotal**	**0**	**0**	**0**	**0**	**0**	**0**	**0**	**0**	**0**	**1**	**0**	**1**	**307**
**Klebsiella oxytoca/bla NDM**													
Não Detectável	0	0	0	0	0	0	0	0	0	1	0	1	2
**Subtotal**	**0**	**0**	**0**	**0**	**0**	**0**	**0**	**0**	**0**	**1**	**0**	**1**	**309**
**Klebsiella pneumoniae/bla IMP**													
Não Detectável	0	0	0	1	0	0	0	0	0	0	0	5	6
**Subtotal**	**0**	**0**	**0**	**1**	**0**	**0**	**0**	**0**	**0**	**0**	**0**	**5**	**315**
**Klebsiella pneumoniae/bla KPC**													
Detectável	0	0	2	1	1	2	0	18	9	3	2	7	45
Não Detectável	0	0	0	0	0	4	0	3	12	0	0	7	26
**Subtotal**	**0**	**0**	**2**	**1**	**1**	**6**	**0**	**21**	**21**	**3**	**2**	**14**	**386**
**Klebsiella pneumoniae/bla NDM**													
Não Detectável	0	0	1	1	0	1	0	0	1	0	0	11	15
**Subtotal**	**0**	**0**	**1**	**1**	**0**	**1**	**0**	**0**	**1**	**0**	**0**	**11**	**401**
**Klebsiella pneumoniae/bla OXA-48**													
Não Detectável	0	0	0	0	0	1	0	0	0	0	0	6	7
**Subtotal**	**0**	**0**	**0**	**0**	**0**	**1**	**0**	**0**	**0**	**0**	**0**	**6**	**408**
**Klebsiella pneumoniae/blaVIM**													
Não Detectável	0	0	1	0	0	0	0	0	0	0	0	0	1
**Subtotal**	**0**	**0**	**1**	**0**	**0**	**0**	**0**	**0**	**0**	**0**	**0**	**0**	**409**
**Klebsiella pneumoniae/KPC**													
Detectável	0	0	0	0	0	0	0	7	0	0	0	0	7
Não Detectável	0	0	0	0	0	0	0	1	0	0	0	0	1

Relatório Pesquisa de Genes de Resistência

Microrganismo /Gene Pesquisado	Jan/2018	Fev/2018	Mar/2018	Abr/2018	Maio/2018	Jun/2018	Jul/2018	Ago/2018	Set/2018	Out/2018	Nov/2018	Dez/2018	Total
**Subtotal**	**0**	**0**	**0**	**0**	**0**	**0**	**0**	**8**	**0**	**0**	**0**	**0**	**417**
**Klebsiella pneumoniae/NDM**													
Não Detectável	0	0	0	0	0	0	0	1	0	0	0	0	1
**Subtotal**	**0**	**0**	**0**	**0**	**0**	**0**	**0**	**1**	**0**	**0**	**0**	**0**	**418**
**Klebsiella pneumoniae subsp. pneumoniae/bla KPC**													
Detectável	0	0	0	0	0	0	0	0	0	2	0	0	2
**Subtotal**	**0**	**0**	**0**	**0**	**0**	**0**	**0**	**0**	**0**	**2**	**0**	**0**	**420**
**Klebsiella sp./bla KPC**													
Não Detectável	0	0	0	0	0	0	0	0	0	1	0	0	1
**Subtotal**	**0**	**0**	**0**	**0**	**0**	**0**	**0**	**0**	**0**	**1**	**0**	**0**	**421**
**Klebsiella sp./bla NDM**													
Não Detectável	0	0	0	0	0	0	0	0	0	1	0	0	1
**Subtotal**	**0**	**0**	**0**	**0**	**0**	**0**	**0**	**0**	**0**	**1**	**0**	**0**	**422**
**Klebsiella sp./KPC**													
Detectável	0	0	0	0	0	0	0	0	0	1	0	0	1
**Subtotal**	**0**	**0**	**0**	**0**	**0**	**0**	**0**	**0**	**0**	**1**	**0**	**0**	**423**
**Morganella morganii/KPC**													
Detectável	0	0	0	0	0	0	0	0	0	1	0	0	1
**Subtotal**	**0**	**0**	**0**	**0**	**0**	**0**	**0**	**0**	**0**	**1**	**0**	**0**	**424**
**Proteus mirabilis/bla IMP**													
Não Detectável	0	0	0	0	0	1	0	0	0	0	0	1	2
**Subtotal**	**0**	**0**	**0**	**0**	**0**	**1**	**0**	**0**	**0**	**0**	**0**	**1**	**426**
**Proteus mirabilis/bla KPC**													
Não Detectável	0	0	0	0	0	1	0	0	0	0	0	1	2
**Subtotal**	**0**	**0**	**0**	**0**	**0**	**1**	**0**	**0**	**0**	**0**	**0**	**1**	**428**
**Proteus mirabilis/bla NDM**													
Não Detectável	0	0	0	0	0	0	0	0	1	0	0	1	2
**Subtotal**	**0**	**0**	**0**	**0**	**0**	**0**	**0**	**0**	**1**	**0**	**0**	**1**	**430**
**Proteus mirabilis/NDM**													
Não Detectável	0	0	0	0	0	1	0	0	0	0	0	0	1
**Subtotal**	**0**	**0**	**0**	**0**	**0**	**1**	**0**	**0**	**0**	**0**	**0**	**0**	**431**
**Proteus sp./bla NDM**													
Não Detectável	0	0	0	0	0	0	0	0	1	0	0	0	1
**Subtotal**	**0**	**0**	**0**	**0**	**0**	**0**	**0**	**0**	**1**	**0**	**0**	**0**	**432**
**Pseudomonas aeruginosa/bla KPC**													
Não Detectável	0	0	0	0	0	0	0	0	0	0	0	1	1

Relatório Pesquisa de Genes de Resistência

Microrganismo /Gene Pesquisado	Jan/2018	Fev/2018	Mar/2018	Abr/2018	Maio/2018	Jun/2018	Jul/2018	Ago/2018	Set/2018	Out/2018	Nov/2018	Dez/2018	Total
**Subtotal**	**0**	**0**	**0**	**0**	**0**	**0**	**0**	**0**	**0**	**0**	**0**	**1**	**433**
**Pseudomonas aeruginosa/bla NDM**													
Não Detectável	0	0	0	0	0	0	0	0	0	1	0	0	1
**Subtotal**	**0**	**0**	**0**	**0**	**0**	**0**	**0**	**0**	**0**	**1**	**0**	**0**	**434**
**Pseudomonas aeruginosa/bla OXA-23**													
Não Detectável	0	0	0	0	0	0	0	0	0	0	0	1	1
**Subtotal**	**0**	**0**	**0**	**0**	**0**	**0**	**0**	**0**	**0**	**0**	**0**	**1**	**435**
**Pseudomonas aeruginosa/bla OXA-48**													
Não Detectável	0	0	0	0	0	0	0	0	0	0	0	1	1
**Subtotal**	**0**	**0**	**0**	**0**	**0**	**0**	**0**	**0**	**0**	**0**	**0**	**1**	**436**
**Pseudomonas aeruginosa/bla OXA-51**													
Não Detectável	0	0	0	0	0	0	0	0	0	0	0	1	1
**Subtotal**	**0**	**0**	**0**	**0**	**0**	**0**	**0**	**0**	**0**	**0**	**0**	**1**	**437**
**Pseudomonas aeruginosa/bla SPM**													
Detectável	0	0	0	0	0	0	0	6	1	0	0	1	8
Não Detectável	0	0	0	0	0	0	0	3	7	4	0	4	18
**Subtotal**	**0**	**0**	**0**	**0**	**0**	**0**	**0**	**9**	**8**	**4**	**0**	**5**	**463**
**Pseudomonas aeruginosa/blaVIM**													
Detectável	0	0	0	0	0	0	0	0	0	0	0	1	1
Não Detectável	0	0	0	0	0	0	0	7	8	5	0	0	20
**Subtotal**	**0**	**0**	**0**	**0**	**0**	**0**	**0**	**7**	**8**	**5**	**0**	**1**	**484**
**Pseudomonas aeruginosa/SPM**													
Detectável	0	0	0	0	0	0	0	5	1	0	0	0	6
Não Detectável	0	0	0	0	0	1	0	2	0	0	0	0	3
**Subtotal**	**0**	**0**	**0**	**0**	**0**	**1**	**0**	**7**	**1**	**0**	**0**	**0**	**493**
**Pseudomonas aeruginosa/VIM**													
Não Detectável	0	0	0	0	0	0	0	6	1	0	0	0	7
**Subtotal**	**0**	**0**	**0**	**0**	**0**	**0**	**0**	**6**	**1**	**0**	**0**	**0**	**500**
**Serratia marcescens/bla IMP**													
Não Detectável	0	0	0	0	0	0	0	0	0	0	0	1	1
**Subtotal**	**0**	**0**	**0**	**0**	**0**	**0**	**0**	**0**	**0**	**0**	**0**	**1**	**501**
**Serratia marcescens/bla KPC**													
Detectável	0	0	0	0	2	0	0	0	1	0	0	1	4
Não Detectável	0	0	0	0	0	0	0	0	0	1	0	0	1
**Subtotal**	**0**	**0**	**0**	**0**	**2**	**0**	**0**	**0**	**1**	**1**	**0**	**1**	**506**
**Serratia marcescens/bla NDM**													

Relatório Pesquisa de Genes de Resistência

Microrganismo /Gene Pesquisado	Jan/2018	Fev/2018	Mar/2018	Abr/2018	Maio/2018	Jun/2018	Jul/2018	Ago/2018	Set/2018	Out/2018	Nov/2018	Dez/2018	Total
Não Detectável	0	0	0	0	0	0	0	3	2	1	0	1	7
**Subtotal**	**0**	**0**	**0**	**0**	**0**	**0**	**0**	**3**	**2**	**1**	**0**	**1**	**513**
**Serratia rubidaea/bla KPC**													
Detectável	0	0	0	0	0	0	0	0	0	1	0	0	1
**Subtotal**	**0**	**0**	**0**	**0**	**0**	**0**	**0**	**0**	**0**	**1**	**0**	**0**	**514**
**Serratia sp./bla KPC**													
Detectável	0	0	0	0	0	0	0	0	1	0	0	0	1
Não Detectável	0	0	0	0	0	0	0	0	1	2	0	0	3
**Subtotal**	**0**	**0**	**0**	**0**	**0**	**0**	**0**	**0**	**2**	**2**	**0**	**0**	**518**
**Serratia sp./bla NDM**													
Não Detectável	0	0	0	0	0	0	0	0	2	2	0	0	4
**Subtotal**	**0**	**0**	**0**	**0**	**0**	**0**	**0**	**0**	**2**	**2**	**0**	**0**	**522**
	0	0	0	1	0	0	0	1	0	0	0	4	6
**Subtotal**	**0**	**0**	**0**	**1**	**0**	**0**	**0**	**1**	**0**	**0**	**0**	**4**	**528**
