# Supplementary material for: Biodiversity of carbapenem-resistant bacteria in clinical samples from the Southwest Amazon region (Rondônia/Brazil)
Source: Sci Rep. 2024 Apr 23;14:9383. doi: 10.1038/s41598-024-59733-w (PMC11039742; doi:10.1038/s41598-024-59733-w)
Supplement: Supplementary file 2 — Supplementary Information 2. [file 41598_2024_59733_MOESM2_ESM.pdf]

Relatório Pesquisa de Genes de Resistência

Data Início: 01/01/2020

Data Fim: 31/12/2020

Qtd. de Exame/Metodologia: 378

Qtd. de Microrganismo/Gene Pesq.: 86

Todos os Laboratórios

Microrganismo /Gene Pesquisado	Jan/2020	Fev/2020	Mar/2020	Abr/2020	Maio/2020	Jun/2020	Jul/2020	Ago/2020	Set/2020	Out/2020	Nov/2020	Dez/2020	Total
**Acinetobacter baumannii/bla KPC**													
Detectável	0	0	0	0	0	1	0	0	0	0	0	0	1
Não Detectável	0	0	0	3	0	0	0	0	0	0	0	0	3
**Subtotal**	**0**	**0**	**0**	**3**	**0**	**1**	**0**	**0**	**0**	**0**	**0**	**0**	**4**
**Acinetobacter baumannii/bla NDM**													
Não Detectável	0	0	0	3	0	0	0	0	0	0	0	0	3
**Subtotal**	**0**	**0**	**0**	**3**	**0**	**0**	**0**	**0**	**0**	**0**	**0**	**0**	**7**
**Acinetobacter baumannii/bla OXA-143**													
Inconclusivo	0	0	0	0	1	0	0	0	0	0	0	0	1
**Subtotal**	**0**	**0**	**0**	**0**	**1**	**0**	**0**	**0**	**0**	**0**	**0**	**0**	**8**
**Acinetobacter baumannii/bla OXA-23**													
Detectável	9	7	7	12	7	14	19	5	0	0	0	0	80
Inconclusivo	0	0	0	0	2	0	0	0	0	0	0	0	2
Não Detectável	2	0	1	4	1	0	5	6	1	0	0	0	20
**Subtotal**	**11**	**7**	**8**	**16**	**10**	**14**	**24**	**11**	**1**	**0**	**0**	**0**	**110**
**Acinetobacter baumannii/bla OXA-48**													
Não Detectável	8	0	0	3	0	0	1	0	0	0	0	0	12
**Subtotal**	**8**	**0**	**0**	**3**	**0**	**0**	**1**	**0**	**0**	**0**	**0**	**0**	**122**
**Acinetobacter baumannii/bla OXA-51**													
Detectável	9	8	7	24	7	14	12	7	0	0	0	0	88
Inconclusivo	0	0	0	0	2	0	0	0	0	0	0	0	2
Não Detectável	2	0	1	4	1	0	6	7	1	0	0	0	22
**Subtotal**	**11**	**8**	**8**	**28**	**10**	**14**	**18**	**14**	**1**	**0**	**0**	**0**	**234**
**Acinetobacter baumannii/bla OXA-58**													
Detectável	0	0	0	0	0	0	13	5	0	0	0	0	18

Relatório Pesquisa de Genes de Resistência

Microrganismo /Gene Pesquisado	Jan/2020	Fev/2020	Mar/2020	Abr/2020	Maio/2020	Jun/2020	Jul/2020	Ago/2020	Set/2020	Out/2020	Nov/2020	Dez/2020	Total
Não Detectável	1	0	0	0	0	0	2	8	1	0	0	0	12
**Subtotal**	**1**	**0**	**0**	**0**	**0**	**0**	**15**	**13**	**1**	**0**	**0**	**0**	**264**
**Acinetobacter sp./bla OXA-23**													
Detectável	2	0	0	0	0	0	0	0	0	0	0	0	2
**Subtotal**	**2**	**0**	**0**	**0**	**0**	**0**	**0**	**0**	**0**	**0**	**0**	**0**	**266**
**Acinetobacter sp./bla OXA-48**													
Não Detectável	1	0	0	0	0	0	0	0	0	0	0	0	1
**Subtotal**	**1**	**0**	**0**	**0**	**0**	**0**	**0**	**0**	**0**	**0**	**0**	**0**	**267**
**Acinetobacter sp./bla OXA-51**													
Detectável	2	0	0	0	0	0	0	0	0	0	0	0	2
**Subtotal**	**2**	**0**	**0**	**0**	**0**	**0**	**0**	**0**	**0**	**0**	**0**	**0**	**269**
**Acinetobacter sp./bla OXA-58**													
Não Detectável	1	0	0	0	0	0	0	0	0	0	0	0	1
**Subtotal**	**1**	**0**	**0**	**0**	**0**	**0**	**0**	**0**	**0**	**0**	**0**	**0**	**270**
**Burkholderia cepacia/bla OXA-143**													
Detectável	0	0	0	0	1	0	0	0	0	0	0	0	1
**Subtotal**	**0**	**0**	**0**	**0**	**1**	**0**	**0**	**0**	**0**	**0**	**0**	**0**	**271**
**Burkholderia cepacia/bla OXA-23**													
Detectável	0	0	0	0	1	0	0	0	0	0	0	0	1
Não Detectável	0	0	0	0	0	0	1	0	0	0	0	0	1
**Subtotal**	**0**	**0**	**0**	**0**	**1**	**0**	**1**	**0**	**0**	**0**	**0**	**0**	**273**
**Burkholderia cepacia/bla OXA-48**													
Não Detectável	0	0	0	0	0	0	1	0	0	0	0	0	1
**Subtotal**	**0**	**0**	**0**	**0**	**0**	**0**	**1**	**0**	**0**	**0**	**0**	**0**	**274**
**Burkholderia cepacia/bla OXA-51**													
Detectável	0	0	0	0	2	0	0	0	0	0	0	0	2
Não Detectável	0	0	0	0	0	0	1	0	0	0	0	0	1
**Subtotal**	**0**	**0**	**0**	**0**	**2**	**0**	**1**	**0**	**0**	**0**	**0**	**0**	**277**
**Citrobacter freundii/bla KPC**													
Não Detectável	0	0	0	0	0	0	0	0	1	0	0	0	1
**Subtotal**	**0**	**0**	**0**	**0**	**0**	**0**	**0**	**0**	**1**	**0**	**0**	**0**	**278**
**Citrobacter koseri/bla KPC**													
Detectável	0	1	0	0	0	0	0	0	0	0	0	0	1
Não Detectável	0	0	0	2	0	0	0	0	0	0	0	0	2
**Subtotal**	**0**	**1**	**0**	**2**	**0**	**0**	**0**	**0**	**0**	**0**	**0**	**0**	**281**
**Citrobacter koseri/bla NDM**													

Relatório Pesquisa de Genes de Resistência

Microrganismo /Gene Pesquisado	Jan/2020	Fev/2020	Mar/2020	Abr/2020	Maio/2020	Jun/2020	Jul/2020	Ago/2020	Set/2020	Out/2020	Nov/2020	Dez/2020	Total
Não Detectável	0	0	0	1	0	0	0	0	0	0	0	0	1
**Subtotal**	**0**	**0**	**0**	**1**	**0**	**0**	**0**	**0**	**0**	**0**	**0**	**0**	**282**
**Citrobacter koseri/bla OXA-48**													
Não Detectável	0	0	0	1	0	0	0	0	0	0	0	0	1
**Subtotal**	**0**	**0**	**0**	**1**	**0**	**0**	**0**	**0**	**0**	**0**	**0**	**0**	**283**
**Citrobacter sp./bla KPC**													
Não Detectável	0	0	0	0	2	0	0	0	0	0	0	0	2
**Subtotal**	**0**	**0**	**0**	**0**	**2**	**0**	**0**	**0**	**0**	**0**	**0**	**0**	**285**
**Enterobacter cloacae/bla KPC**													
Detectável	1	0	1	0	0	0	0	0	0	0	0	0	2
Não Detectável	0	0	1	0	0	1	3	0	0	0	0	0	5
**Subtotal**	**1**	**0**	**2**	**0**	**0**	**1**	**3**	**0**	**0**	**0**	**0**	**0**	**292**
**Enterobacter cloacae/bla NDM**													
Detectável	0	0	1	0	0	0	0	0	0	0	0	0	1
Não Detectável	0	0	1	0	0	1	3	0	0	0	0	0	5
**Subtotal**	**0**	**0**	**2**	**0**	**0**	**1**	**3**	**0**	**0**	**0**	**0**	**0**	**298**
**Enterobacter cloacae/bla OXA-48**													
Não Detectável	0	0	0	0	0	0	3	0	0	0	0	0	3
**Subtotal**	**0**	**0**	**0**	**0**	**0**	**0**	**3**	**0**	**0**	**0**	**0**	**0**	**301**
**Escherichia coli/bla IMP**													
Não Detectável	0	0	0	1	0	0	0	0	0	0	0	0	1
**Subtotal**	**0**	**0**	**0**	**1**	**0**	**0**	**0**	**0**	**0**	**0**	**0**	**0**	**302**
**Escherichia coli/bla KPC**													
Detectável	0	1	0	1	0	0	0	0	0	0	0	0	2
Não Detectável	2	1	1	8	1	0	0	1	0	0	0	0	14
**Subtotal**	**2**	**2**	**1**	**9**	**1**	**0**	**0**	**1**	**0**	**0**	**0**	**0**	**318**
**Escherichia coli/bla NDM**													
Não Detectável	2	0	1	5	0	0	0	1	0	0	0	0	9
**Subtotal**	**2**	**0**	**1**	**5**	**0**	**0**	**0**	**1**	**0**	**0**	**0**	**0**	**327**
**Escherichia coli/bla OXA-48**													
Não Detectável	0	0	0	5	0	0	0	1	0	0	0	0	6
**Subtotal**	**0**	**0**	**0**	**5**	**0**	**0**	**0**	**1**	**0**	**0**	**0**	**0**	**333**
**Escherichia coli/bla SPM**													
Não Detectável	0	0	0	1	0	0	0	0	0	0	0	0	1
**Subtotal**	**0**	**0**	**0**	**1**	**0**	**0**	**0**	**0**	**0**	**0**	**0**	**0**	**334**
**Escherichia coli/blaVIM**													

Relatório Pesquisa de Genes de Resistência

Microrganismo /Gene Pesquisado	Jan/2020	Fev/2020	Mar/2020	Abr/2020	Maio/2020	Jun/2020	Jul/2020	Ago/2020	Set/2020	Out/2020	Nov/2020	Dez/2020	Total
Não Detectável	0	0	0	1	0	0	0	0	0	0	0	0	1
**Subtotal**	**0**	**0**	**0**	**1**	**0**	**0**	**0**	**0**	**0**	**0**	**0**	**0**	**335**
**Escherichia coli enteroinvasora/outros**													
Detectável	0	0	0	0	0	0	1	0	0	0	0	0	1
**Subtotal**	**0**	**0**	**0**	**0**	**0**	**0**	**1**	**0**	**0**	**0**	**0**	**0**	**336**
**Escherichia coli enterotoxigênica/outros**													
Detectável	0	0	0	0	0	0	1	0	0	0	0	0	1
**Subtotal**	**0**	**0**	**0**	**0**	**0**	**0**	**1**	**0**	**0**	**0**	**0**	**0**	**337**
**Escherichia coli/KPC**													
Não Detectável	0	0	0	1	0	0	0	0	0	0	0	0	1
**Subtotal**	**0**	**0**	**0**	**1**	**0**	**0**	**0**	**0**	**0**	**0**	**0**	**0**	**338**
**Escherichia coli/outros**													
Detectável	0	0	0	0	1	0	0	0	0	0	0	0	1
Não Detectável	0	0	0	0	1	0	0	0	0	0	0	0	1
**Subtotal**	**0**	**0**	**0**	**0**	**2**	**0**	**0**	**0**	**0**	**0**	**0**	**0**	**340**
**Klebsiella aerogenes/bla KPC**													
Não Detectável	0	0	1	0	0	0	0	0	0	0	0	0	1
**Subtotal**	**0**	**0**	**1**	**0**	**0**	**0**	**0**	**0**	**0**	**0**	**0**	**0**	**341**
**Klebsiella aerogenes/bla NDM**													
Não Detectável	0	0	1	0	0	0	0	0	0	0	0	0	1
**Subtotal**	**0**	**0**	**1**	**0**	**0**	**0**	**0**	**0**	**0**	**0**	**0**	**0**	**342**
**Klebsiella ozaenae/bla KPC**													
Não Detectável	0	1	0	0	0	0	0	0	0	0	0	0	1
**Subtotal**	**0**	**1**	**0**	**0**	**0**	**0**	**0**	**0**	**0**	**0**	**0**	**0**	**343**
**Klebsiella pneumoniae/bla IMP**													
Não Detectável	0	0	0	1	0	0	2	0	0	0	0	0	3
**Subtotal**	**0**	**0**	**0**	**1**	**0**	**0**	**2**	**0**	**0**	**0**	**0**	**0**	**346**
**Klebsiella pneumoniae/bla KPC**													
Detectável	6	9	6	9	9	5	5	7	3	0	0	0	59
Inconclusivo	0	0	0	0	3	0	0	0	0	0	0	0	3
Não Detectável	7	5	7	15	1	3	20	4	6	0	0	0	68
**Subtotal**	**13**	**14**	**13**	**24**	**13**	**8**	**25**	**11**	**9**	**0**	**0**	**0**	**476**
**Klebsiella pneumoniae/bla NDM**													
Não Detectável	1	0	5	10	1	1	20	0	5	0	0	0	43
**Subtotal**	**1**	**0**	**5**	**10**	**1**	**1**	**20**	**0**	**5**	**0**	**0**	**0**	**519**
**Klebsiella pneumoniae/bla OXA-143**													

Relatório Pesquisa de Genes de Resistência

Microrganismo /Gene Pesquisado	Jan/2020	Fev/2020	Mar/2020	Abr/2020	Maio/2020	Jun/2020	Jul/2020	Ago/2020	Set/2020	Out/2020	Nov/2020	Dez/2020	Total
Não Detectável	0	0	0	1	0	0	0	0	0	0	0	0	1
**Subtotal**	**0**	**0**	**0**	**1**	**0**	**0**	**0**	**0**	**0**	**0**	**0**	**0**	**520**
**Klebsiella pneumoniae/bla OXA-23**													
Detectável	0	0	0	0	0	0	1	0	0	0	0	0	1
**Subtotal**	**0**	**0**	**0**	**0**	**0**	**0**	**1**	**0**	**0**	**0**	**0**	**0**	**521**
**Klebsiella pneumoniae/bla OXA-48**													
Não Detectável	0	0	0	9	0	0	20	0	5	0	0	0	34
**Subtotal**	**0**	**0**	**0**	**9**	**0**	**0**	**20**	**0**	**5**	**0**	**0**	**0**	**555**
**Klebsiella pneumoniae/bla OXA-58**													
Detectável	0	0	0	0	0	0	1	0	0	0	0	0	1
**Subtotal**	**0**	**0**	**0**	**0**	**0**	**0**	**1**	**0**	**0**	**0**	**0**	**0**	**556**
**Klebsiella pneumoniae/bla SPM**													
Detectável	1	1	0	0	0	0	0	0	0	0	0	0	2
Não Detectável	0	1	1	1	0	1	0	0	0	0	0	0	4
**Subtotal**	**1**	**2**	**1**	**1**	**0**	**1**	**0**	**0**	**0**	**0**	**0**	**0**	**562**
**Klebsiella pneumoniae/blaVIM**													
Não Detectável	1	1	1	1	0	0	2	0	0	0	0	0	6
**Subtotal**	**1**	**1**	**1**	**1**	**0**	**0**	**2**	**0**	**0**	**0**	**0**	**0**	**568**
**Morganella morganii/bla KPC**													
Detectável	0	0	0	0	1	0	0	0	0	0	0	0	1
Não Detectável	0	0	1	2	1	1	0	0	0	0	0	0	5
**Subtotal**	**0**	**0**	**1**	**2**	**2**	**1**	**0**	**0**	**0**	**0**	**0**	**0**	**574**
**Morganella morganii/bla NDM**													
Não Detectável	0	0	1	2	1	0	0	0	0	0	0	0	4
**Subtotal**	**0**	**0**	**1**	**2**	**1**	**0**	**0**	**0**	**0**	**0**	**0**	**0**	**578**
**Morganella morganii/bla OXA-48**													
Não Detectável	0	0	0	2	0	0	0	0	0	0	0	0	2
**Subtotal**	**0**	**0**	**0**	**2**	**0**	**0**	**0**	**0**	**0**	**0**	**0**	**0**	**580**
**Morganella morganii/bla SPM**													
Inconclusivo	0	0	0	0	1	0	0	0	0	0	0	0	1
**Subtotal**	**0**	**0**	**0**	**0**	**1**	**0**	**0**	**0**	**0**	**0**	**0**	**0**	**581**
**Proteus mirabilis/bla KPC**													
Inconclusivo	0	0	0	0	0	1	0	0	0	0	0	0	1
Não Detectável	0	0	0	1	1	0	0	0	0	0	0	0	2
**Subtotal**	**0**	**0**	**0**	**1**	**1**	**1**	**0**	**0**	**0**	**0**	**0**	**0**	**584**
**Proteus mirabilis/bla NDM**													

Relatório Pesquisa de Genes de Resistência

Microrganismo /Gene Pesquisado	Jan/2020	Fev/2020	Mar/2020	Abr/2020	Maio/2020	Jun/2020	Jul/2020	Ago/2020	Set/2020	Out/2020	Nov/2020	Dez/2020	Total
Não Detectável	0	0	0	1	1	0	0	0	0	0	0	0	2
**Subtotal**	**0**	**0**	**0**	**1**	**1**	**0**	**0**	**0**	**0**	**0**	**0**	**0**	**586**
**Proteus mirabilis/bla OXA-48**													
Não Detectável	0	0	0	1	1	0	0	0	0	0	0	0	2
**Subtotal**	**0**	**0**	**0**	**1**	**1**	**0**	**0**	**0**	**0**	**0**	**0**	**0**	**588**
**Proteus vulgaris/bla KPC**													
Não Detectável	0	0	1	0	0	0	0	0	0	0	0	0	1
**Subtotal**	**0**	**0**	**1**	**0**	**0**	**0**	**0**	**0**	**0**	**0**	**0**	**0**	**589**
**Proteus vulgaris/bla NDM**													
Não Detectável	0	0	1	0	0	0	0	0	0	0	0	0	1
**Subtotal**	**0**	**0**	**1**	**0**	**0**	**0**	**0**	**0**	**0**	**0**	**0**	**0**	**590**
**Providencia stuartii/bla IMP**													
Não Detectável	0	0	0	1	0	0	0	0	0	0	0	0	1
**Subtotal**	**0**	**0**	**0**	**1**	**0**	**0**	**0**	**0**	**0**	**0**	**0**	**0**	**591**
**Providencia stuartii/bla KPC**													
Detectável	0	0	1	0	0	0	0	0	0	0	0	0	1
Não Detectável	0	2	2	2	0	0	0	0	1	0	0	0	7
**Subtotal**	**0**	**2**	**3**	**2**	**0**	**0**	**0**	**0**	**1**	**0**	**0**	**0**	**599**
**Providencia stuartii/bla NDM**													
Não Detectável	0	2	1	2	0	0	0	0	1	0	0	0	6
**Subtotal**	**0**	**2**	**1**	**2**	**0**	**0**	**0**	**0**	**1**	**0**	**0**	**0**	**605**
**Providencia stuartii/bla OXA-48**													
Não Detectável	0	0	1	2	0	0	0	0	1	0	0	0	4
**Subtotal**	**0**	**0**	**1**	**2**	**0**	**0**	**0**	**0**	**1**	**0**	**0**	**0**	**609**
**Providencia stuartii/bla SPM**													
Não Detectável	0	0	1	1	0	0	0	0	0	0	0	0	2
**Subtotal**	**0**	**0**	**1**	**1**	**0**	**0**	**0**	**0**	**0**	**0**	**0**	**0**	**611**
**Pseudomonas aeruginosa/bla IMP**													
Não Detectável	0	0	0	3	0	0	3	0	0	0	0	0	6
**Subtotal**	**0**	**0**	**0**	**3**	**0**	**0**	**3**	**0**	**0**	**0**	**0**	**0**	**617**
**Pseudomonas aeruginosa/bla KPC**													
Detectável	0	1	0	0	0	0	0	0	0	0	0	0	1
Não Detectável	0	0	6	8	0	0	6	2	0	0	0	0	22
**Subtotal**	**0**	**1**	**6**	**8**	**0**	**0**	**6**	**2**	**0**	**0**	**0**	**0**	**640**
**Pseudomonas aeruginosa/bla NDM**													
Não Detectável	0	0	1	9	0	0	8	1	0	0	0	0	19

Relatório Pesquisa de Genes de Resistência

Microrganismo /Gene Pesquisado	Jan/2020	Fev/2020	Mar/2020	Abr/2020	Maio/2020	Jun/2020	Jul/2020	Ago/2020	Set/2020	Out/2020	Nov/2020	Dez/2020	Total
**Subtotal**	**0**	**0**	**1**	**9**	**0**	**0**	**8**	**1**	**0**	**0**	**0**	**0**	**659**
**Pseudomonas aeruginosa/bla OXA-23**													
Detectável	0	0	0	0	0	0	1	0	0	0	0	0	1
**Subtotal**	**0**	**0**	**0**	**0**	**0**	**0**	**1**	**0**	**0**	**0**	**0**	**0**	**660**
**Pseudomonas aeruginosa/bla OXA-48**													
Não Detectável	0	0	8	8	0	0	13	1	0	0	0	0	30
**Subtotal**	**0**	**0**	**8**	**8**	**0**	**0**	**13**	**1**	**0**	**0**	**0**	**0**	**690**
**Pseudomonas aeruginosa/bla OXA-51**													
Detectável	0	0	0	0	0	0	1	0	0	0	0	0	1
**Subtotal**	**0**	**0**	**0**	**0**	**0**	**0**	**1**	**0**	**0**	**0**	**0**	**0**	**691**
**Pseudomonas aeruginosa/bla SPM**													
Detectável	0	1	1	0	0	0	0	0	0	0	0	0	2
Inconclusivo	0	0	0	0	1	0	0	0	0	0	0	0	1
Não Detectável	3	2	12	4	0	0	14	2	0	0	0	0	37
**Subtotal**	**3**	**3**	**13**	**4**	**1**	**0**	**14**	**2**	**0**	**0**	**0**	**0**	**731**
**Pseudomonas aeruginosa/blaVIM**													
Detectável	0	0	1	0	0	0	0	0	0	0	0	0	1
Não Detectável	3	2	7	1	0	0	10	0	0	0	0	0	23
**Subtotal**	**3**	**2**	**8**	**1**	**0**	**0**	**10**	**0**	**0**	**0**	**0**	**0**	**755**
**Pseudomonas aeruginosa/SPM**													
Não Detectável	2	0	0	0	0	0	0	0	0	0	0	0	2
**Subtotal**	**2**	**0**	**0**	**0**	**0**	**0**	**0**	**0**	**0**	**0**	**0**	**0**	**757**
**Pseudomonas aeruginosa/VIM**													
Não Detectável	2	0	0	0	0	0	0	0	0	0	0	0	2
**Subtotal**	**2**	**0**	**0**	**0**	**0**	**0**	**0**	**0**	**0**	**0**	**0**	**0**	**759**
**Pseudomonas putida/bla SPM**													
Não Detectável	0	0	1	0	0	0	0	0	0	0	0	0	1
**Subtotal**	**0**	**0**	**1**	**0**	**0**	**0**	**0**	**0**	**0**	**0**	**0**	**0**	**760**
**Pseudomonas putida/blaVIM**													
Não Detectável	0	0	1	0	0	0	0	0	0	0	0	0	1
**Subtotal**	**0**	**0**	**1**	**0**	**0**	**0**	**0**	**0**	**0**	**0**	**0**	**0**	**761**
**Serratia marcescens/bla KPC**													
Não Detectável	0	0	2	2	1	1	0	0	0	0	0	0	6
**Subtotal**	**0**	**0**	**2**	**2**	**1**	**1**	**0**	**0**	**0**	**0**	**0**	**0**	**767**
**Serratia marcescens/bla NDM**													
Não Detectável	0	1	2	1	1	0	0	0	0	0	0	0	5

Relatório Pesquisa de Genes de Resistência

Microrganismo /Gene Pesquisado	Jan/2020	Fev/2020	Mar/2020	Abr/2020	Maio/2020	Jun/2020	Jul/2020	Ago/2020	Set/2020	Out/2020	Nov/2020	Dez/2020	Total
**Subtotal**	**0**	**1**	**2**	**1**	**1**	**0**	**0**	**0**	**0**	**0**	**0**	**0**	**772**
**Serratia marcescens/bla OXA-48**													
Não Detectável	0	0	2	1	1	0	0	0	0	0	0	0	4
**Subtotal**	**0**	**0**	**2**	**1**	**1**	**0**	**0**	**0**	**0**	**0**	**0**	**0**	**776**
**Serratia marcescens/bla SPM**													
Não Detectável	0	0	1	0	0	0	0	0	0	0	0	0	1
**Subtotal**	**0**	**0**	**1**	**0**	**0**	**0**	**0**	**0**	**0**	**0**	**0**	**0**	**777**
**Serratia plymuthica/bla KPC**													
Não Detectável	0	0	0	0	0	2	0	0	0	0	0	0	2
**Subtotal**	**0**	**0**	**0**	**0**	**0**	**2**	**0**	**0**	**0**	**0**	**0**	**0**	**779**
**Serratia plymuthica/bla NDM**													
Inconclusivo	0	0	0	0	0	1	0	0	0	0	0	0	1
**Subtotal**	**0**	**0**	**0**	**0**	**0**	**1**	**0**	**0**	**0**	**0**	**0**	**0**	**780**
**Staphylococcus aureus/bla KPC**													
Não Detectável	1	0	0	0	0	0	0	0	0	0	0	0	1
**Subtotal**	**1**	**0**	**0**	**0**	**0**	**0**	**0**	**0**	**0**	**0**	**0**	**0**	**781**
**Staphylococcus aureus/bla NDM**													
Não Detectável	1	0	0	0	0	0	0	0	0	0	0	0	1
**Subtotal**	**1**	**0**	**0**	**0**	**0**	**0**	**0**	**0**	**0**	**0**	**0**	**0**	**782**
**Staphylococcus aureus/mec a**													
Detectável	0	1	1	0	0	0	0	0	0	0	0	0	2
Não Detectável	0	0	0	2	0	1	3	5	1	0	0	0	12
**Subtotal**	**0**	**1**	**1**	**2**	**0**	**1**	**3**	**5**	**1**	**0**	**0**	**0**	**796**
**Staphylococcus aureus/outros**													
Detectável	0	0	0	0	0	0	1	0	0	0	0	0	1
**Subtotal**	**0**	**0**	**0**	**0**	**0**	**0**	**1**	**0**	**0**	**0**	**0**	**0**	**797**
**Stenotrophomonas maltophilia/bla KPC**													
Não Detectável	0	0	0	1	0	0	0	0	0	0	0	0	1
**Subtotal**	**0**	**0**	**0**	**1**	**0**	**0**	**0**	**0**	**0**	**0**	**0**	**0**	**798**
**Stenotrophomonas maltophilia/bla OXA-48**													
Não Detectável	0	0	0	1	0	0	0	0	0	0	0	0	1
**Subtotal**	**0**	**0**	**0**	**1**	**0**	**0**	**0**	**0**	**0**	**0**	**0**	**0**	**799**
**Stenotrophomonas maltophilia/bla OXA-51**													
Detectável	0	0	0	0	1	0	0	0	0	0	0	0	1
**Subtotal**	**0**	**0**	**0**	**0**	**1**	**0**	**0**	**0**	**0**	**0**	**0**	**0**	**800**
**Stenotrophomonas maltophilia/bla SPM**													

Relatório Pesquisa de Genes de Resistência

Microrganismo /Gene Pesquisado	Jan/2020	Fev/2020	Mar/2020	Abr/2020	Maio/2020	Jun/2020	Jul/2020	Ago/2020	Set/2020	Out/2020	Nov/2020	Dez/2020	Total
Detectável	0	0	0	0	1	0	0	0	0	0	0	0	1
Não Detectável	0	0	0	1	0	0	0	0	0	0	0	0	1
**Subtotal**	**0**	**0**	**0**	**1**	**1**	**0**	**0**	**0**	**0**	**0**	**0**	**0**	**802**
	2	0	0	2	3	1	0	0	0	0	0	0	8
**Subtotal**	**2**	**0**	**0**	**2**	**3**	**1**	**0**	**0**	**0**	**0**	**0**	**0**	**810**
