# Supplementary material for: Biodiversity of carbapenem-resistant bacteria in clinical samples from the Southwest Amazon region (Rondônia/Brazil)
Source: Sci Rep. 2024 Apr 23;14:9383. doi: 10.1038/s41598-024-59733-w (PMC11039742; doi:10.1038/s41598-024-59733-w)
Supplement: Supplementary file 3 — Supplementary Information 3. [file 41598_2024_59733_MOESM3_ESM.pdf]

Relatório Pesquisa de Genes de Resistência

Data Início: 01/01/2021

Data Fim: 31/12/2021

Qtd. de Exame/Metodologia: 1107

Qtd. de Microrganismo/Gene Pesq.: 255

Todos os Laboratórios

Microrganismo /Gene Pesquisado	Jan/2021	Fev/2021	Mar/2021	Abr/2021	Maio/2021	Jun/2021	Jul/2021	Ago/2021	Set/2021	Out/2021	Nov/2021	Dez/2021	Total
**Acinetobacter baumannii/bla IMP**													
Inconclusivo	0	0	0	0	0	1	0	0	0	0	0	0	1
Não Detectável	0	0	0	1	7	30	9	0	0	0	0	0	47
**Subtotal**	**0**	**0**	**0**	**1**	**7**	**31**	**9**	**0**	**0**	**0**	**0**	**0**	**48**
**Acinetobacter baumannii/bla KPC**													
Inconclusivo	0	0	0	0	1	1	0	0	0	0	0	0	2
Não Detectável	0	0	0	1	24	55	38	4	0	7	8	12	149
**Subtotal**	**0**	**0**	**0**	**1**	**25**	**56**	**38**	**4**	**0**	**7**	**8**	**12**	**199**
**Acinetobacter baumannii/bla NDM**													
Detectado traços	0	0	0	0	0	1	0	0	0	0	0	0	1
Detectável	0	0	0	0	0	0	1	0	0	0	1	1	3
Não Detectável	0	0	0	0	26	43	37	4	0	7	7	11	135
**Subtotal**	**0**	**0**	**0**	**0**	**26**	**44**	**38**	**4**	**0**	**7**	**8**	**12**	**338**
**Acinetobacter baumannii/bla OXA-143**													
Detectável	0	11	0	14	0	0	0	0	0	0	0	0	25
Inconclusivo	0	0	0	0	0	1	0	0	0	0	0	0	1
Não Detectável	0	60	70	57	61	62	39	4	0	7	8	13	381
**Subtotal**	**0**	**71**	**70**	**71**	**61**	**63**	**39**	**4**	**0**	**7**	**8**	**13**	**745**
**Acinetobacter baumannii/bla OXA-23**													
Detectado traços	0	0	0	0	0	1	1	0	0	0	0	0	2
Detectável	0	58	63	26	5	6	4	0	0	7	6	12	187
Inconclusivo	0	0	0	0	0	1	0	0	0	0	0	0	1
Não Detectável	0	15	14	50	60	57	38	6	0	0	3	0	243
**Subtotal**	**0**	**73**	**77**	**76**	**65**	**65**	**43**	**6**	**0**	**7**	**9**	**12**	**1178**
**Acinetobacter baumannii/bla OXA-24**													

Relatório Pesquisa de Genes de Resistência

Microrganismo /Gene Pesquisado	Jan/2021	Fev/2021	Mar/2021	Abr/2021	Maio/2021	Jun/2021	Jul/2021	Ago/2021	Set/2021	Out/2021	Nov/2021	Dez/2021	Total
Detectável	0	0	0	0	0	0	0	0	0	0	2	0	2
Não Detectável	0	0	0	0	0	0	0	0	0	0	1	0	1
**Subtotal**	**0**	**0**	**0**	**0**	**0**	**0**	**0**	**0**	**0**	**0**	**3**	**0**	**1181**
**Acinetobacter baumannii/bla OXA-48**													
Detectável	0	0	0	0	0	3	0	0	0	0	0	0	3
Inconclusivo	0	0	0	0	0	1	0	0	0	0	0	0	1
Não Detectável	0	1	1	0	16	41	37	4	0	7	8	11	126
**Subtotal**	**0**	**1**	**1**	**0**	**16**	**45**	**37**	**4**	**0**	**7**	**8**	**11**	**1311**
**Acinetobacter baumannii/bla OXA-51**													
Detectado traços	0	0	0	0	0	1	0	0	0	0	0	0	1
Detectável	0	23	72	24	9	20	10	0	0	7	7	12	184
Inconclusivo	0	0	0	0	0	1	0	0	0	0	0	0	1
Não Detectável	0	50	5	52	56	44	33	6	0	0	0	0	246
**Subtotal**	**0**	**73**	**77**	**76**	**65**	**66**	**43**	**6**	**0**	**7**	**7**	**12**	**1743**
**Acinetobacter baumannii/bla OXA-58**													
Detectado traços	0	0	0	0	0	2	0	0	0	0	0	0	2
Detectável	0	37	23	12	1	2	0	0	0	0	0	0	75
Inconclusivo	0	0	0	0	0	1	0	0	0	0	0	0	1
Não Detectável	0	37	53	63	64	54	43	6	0	0	2	0	322
**Subtotal**	**0**	**74**	**76**	**75**	**65**	**59**	**43**	**6**	**0**	**0**	**2**	**0**	**2143**
**Acinetobacter baumannii/bla SPM**													
Inconclusivo	0	0	0	0	0	1	0	0	0	0	0	0	1
Não Detectável	0	0	0	1	14	38	14	4	0	0	0	0	71
**Subtotal**	**0**	**0**	**0**	**1**	**14**	**39**	**14**	**4**	**0**	**0**	**0**	**0**	**2215**
**Acinetobacter baumannii/blaVIM**													
Inconclusivo	0	0	0	0	0	1	0	0	0	0	0	0	1
Não Detectável	0	0	0	0	9	27	31	4	0	0	0	0	71
**Subtotal**	**0**	**0**	**0**	**0**	**9**	**28**	**31**	**4**	**0**	**0**	**0**	**0**	**2287**
**Acinetobacter calcoaceticus/bla IMP**													
Não Detectável	0	0	0	0	3	1	0	0	0	0	0	0	4
**Subtotal**	**0**	**0**	**0**	**0**	**3**	**1**	**0**	**0**	**0**	**0**	**0**	**0**	**2291**
**Acinetobacter calcoaceticus/bla KPC**													
Não Detectável	0	0	0	0	3	1	0	0	0	0	0	0	4
**Subtotal**	**0**	**0**	**0**	**0**	**3**	**1**	**0**	**0**	**0**	**0**	**0**	**0**	**2295**
**Acinetobacter calcoaceticus/bla NDM**													
Não Detectável	0	0	0	0	3	1	0	0	0	0	0	0	4

Relatório Pesquisa de Genes de Resistência

Microrganismo /Gene Pesquisado	Jan/2021	Fev/2021	Mar/2021	Abr/2021	Maio/2021	Jun/2021	Jul/2021	Ago/2021	Set/2021	Out/2021	Nov/2021	Dez/2021	Total
**Subtotal**	**0**	**0**	**0**	**0**	**3**	**1**	**0**	**0**	**0**	**0**	**0**	**0**	**2299**
**Acinetobacter calcoaceticus/bla OXA-143**													
Não Detectável	0	0	0	0	3	1	0	0	0	0	0	0	4
**Subtotal**	**0**	**0**	**0**	**0**	**3**	**1**	**0**	**0**	**0**	**0**	**0**	**0**	**2303**
**Acinetobacter calcoaceticus/bla OXA-23**													
Não Detectável	0	0	0	0	3	2	0	0	0	0	0	0	5
**Subtotal**	**0**	**0**	**0**	**0**	**3**	**2**	**0**	**0**	**0**	**0**	**0**	**0**	**2308**
**Acinetobacter calcoaceticus/bla OXA-48**													
Não Detectável	0	0	0	0	3	1	0	0	0	0	0	0	4
**Subtotal**	**0**	**0**	**0**	**0**	**3**	**1**	**0**	**0**	**0**	**0**	**0**	**0**	**2312**
**Acinetobacter calcoaceticus/bla OXA-51**													
Detectável	0	0	0	0	1	2	0	0	0	0	0	0	3
Não Detectável	0	0	0	0	2	0	0	0	0	0	0	0	2
**Subtotal**	**0**	**0**	**0**	**0**	**3**	**2**	**0**	**0**	**0**	**0**	**0**	**0**	**2317**
**Acinetobacter calcoaceticus/bla OXA-58**													
Detectável	0	0	0	0	0	1	0	0	0	0	0	0	1
Não Detectável	0	0	0	0	3	1	0	0	0	0	0	0	4
**Subtotal**	**0**	**0**	**0**	**0**	**3**	**2**	**0**	**0**	**0**	**0**	**0**	**0**	**2322**
**Acinetobacter calcoaceticus/bla SPM**													
Não Detectável	0	0	0	0	3	1	0	0	0	0	0	0	4
**Subtotal**	**0**	**0**	**0**	**0**	**3**	**1**	**0**	**0**	**0**	**0**	**0**	**0**	**2326**
**Acinetobacter lwoffii/bla OXA-143**													
Não Detectável	0	0	0	0	2	0	0	0	0	0	0	0	2
**Subtotal**	**0**	**0**	**0**	**0**	**2**	**0**	**0**	**0**	**0**	**0**	**0**	**0**	**2328**
**Acinetobacter lwoffii/bla OXA-23**													
Não Detectável	0	0	0	0	2	0	0	0	0	0	0	0	2
**Subtotal**	**0**	**0**	**0**	**0**	**2**	**0**	**0**	**0**	**0**	**0**	**0**	**0**	**2330**
**Acinetobacter lwoffii/bla OXA-51**													
Não Detectável	0	0	0	0	2	0	0	0	0	0	0	0	2
**Subtotal**	**0**	**0**	**0**	**0**	**2**	**0**	**0**	**0**	**0**	**0**	**0**	**0**	**2332**
**Acinetobacter lwoffii/bla OXA-58**													
Não Detectável	0	0	0	0	2	0	0	0	0	0	0	0	2
**Subtotal**	**0**	**0**	**0**	**0**	**2**	**0**	**0**	**0**	**0**	**0**	**0**	**0**	**2334**
**Acinetobacter sp./bla IMP**													
Não Detectável	0	0	0	0	0	2	1	0	0	0	0	0	3
**Subtotal**	**0**	**0**	**0**	**0**	**0**	**2**	**1**	**0**	**0**	**0**	**0**	**0**	**2337**

Relatório Pesquisa de Genes de Resistência

Microrganismo /Gene Pesquisado	Jan/2021	Fev/2021	Mar/2021	Abr/2021	Maio/2021	Jun/2021	Jul/2021	Ago/2021	Set/2021	Out/2021	Nov/2021	Dez/2021	Total
**Acinetobacter sp./bla KPC**													
Não Detectável	0	0	0	0	4	3	3	0	0	0	0	1	11
**Subtotal**	**0**	**0**	**0**	**0**	**4**	**3**	**3**	**0**	**0**	**0**	**0**	**1**	**2348**
**Acinetobacter sp./bla NDM**													
Não Detectável	0	0	0	0	4	3	3	0	0	0	0	1	11
**Subtotal**	**0**	**0**	**0**	**0**	**4**	**3**	**3**	**0**	**0**	**0**	**0**	**1**	**2359**
**Acinetobacter sp./bla OXA-143**													
Detectável	0	1	0	0	0	0	0	0	0	0	0	0	1
Não Detectável	0	1	0	2	6	3	3	0	0	0	0	1	16
**Subtotal**	**0**	**2**	**0**	**2**	**6**	**3**	**3**	**0**	**0**	**0**	**0**	**1**	**2376**
**Acinetobacter sp./bla OXA-23**													
Detectável	0	1	0	0	0	1	1	0	0	0	0	0	3
Não Detectável	0	1	0	2	6	2	3	0	0	0	0	1	15
**Subtotal**	**0**	**2**	**0**	**2**	**6**	**3**	**4**	**0**	**0**	**0**	**0**	**1**	**2394**
**Acinetobacter sp./bla OXA-24**													
Não Detectável	0	0	0	0	0	0	0	0	0	0	0	1	1
**Subtotal**	**0**	**0**	**0**	**0**	**0**	**0**	**0**	**0**	**0**	**0**	**0**	**1**	**2395**
**Acinetobacter sp./bla OXA-48**													
Não Detectável	0	0	0	0	0	3	3	0	0	0	0	1	7
**Subtotal**	**0**	**0**	**0**	**0**	**0**	**3**	**3**	**0**	**0**	**0**	**0**	**1**	**2402**
**Acinetobacter sp./bla OXA-51**													
Detectável	0	1	0	0	0	2	1	0	0	0	0	0	4
Não Detectável	0	1	0	2	6	1	3	0	0	0	0	1	14
**Subtotal**	**0**	**2**	**0**	**2**	**6**	**3**	**4**	**0**	**0**	**0**	**0**	**1**	**2420**
**Acinetobacter sp./bla OXA-58**													
Detectado traços	0	0	0	0	0	1	0	0	0	0	0	0	1
Detectável	0	0	0	0	0	1	1	0	0	0	0	0	2
Não Detectável	0	2	0	2	6	1	3	0	0	0	0	1	15
**Subtotal**	**0**	**2**	**0**	**2**	**6**	**3**	**4**	**0**	**0**	**0**	**0**	**1**	**2438**
**Acinetobacter sp./bla SPM**													
Não Detectável	0	0	0	0	0	2	2	0	0	0	0	0	4
**Subtotal**	**0**	**0**	**0**	**0**	**0**	**2**	**2**	**0**	**0**	**0**	**0**	**0**	**2442**
**Acinetobacter sp./blaVIM**													
Não Detectável	0	0	0	0	0	2	2	0	0	0	0	0	4
**Subtotal**	**0**	**0**	**0**	**0**	**0**	**2**	**2**	**0**	**0**	**0**	**0**	**0**	**2446**
**Burkholderia cepacia/bla KPC**													

Relatório Pesquisa de Genes de Resistência

Microrganismo /Gene Pesquisado	Jan/2021	Fev/2021	Mar/2021	Abr/2021	Maio/2021	Jun/2021	Jul/2021	Ago/2021	Set/2021	Out/2021	Nov/2021	Dez/2021	Total
Não Detectável	0	0	0	0	0	0	0	0	0	0	1	0	1
**Subtotal**	**0**	**0**	**0**	**0**	**0**	**0**	**0**	**0**	**0**	**0**	**1**	**0**	**2447**
**Burkholderia cepacia/bla NDM**													
Não Detectável	0	0	0	0	0	0	0	0	0	0	1	0	1
**Subtotal**	**0**	**0**	**0**	**0**	**0**	**0**	**0**	**0**	**0**	**0**	**1**	**0**	**2448**
**Burkholderia cepacia/bla OXA-48**													
Não Detectável	0	0	0	0	0	0	0	0	0	0	1	0	1
**Subtotal**	**0**	**0**	**0**	**0**	**0**	**0**	**0**	**0**	**0**	**0**	**1**	**0**	**2449**
**Burkholderia cepacia complex/bla IMP**													
Não Detectável	0	1	0	0	0	0	0	0	0	0	0	0	1
**Subtotal**	**0**	**1**	**0**	**0**	**0**	**0**	**0**	**0**	**0**	**0**	**0**	**0**	**2450**
**Burkholderia cepacia complex/bla KPC**													
Não Detectável	0	1	0	0	0	0	0	0	0	0	0	0	1
**Subtotal**	**0**	**1**	**0**	**0**	**0**	**0**	**0**	**0**	**0**	**0**	**0**	**0**	**2451**
**Burkholderia cepacia complex/bla NDM**													
Não Detectável	0	1	0	0	0	0	0	0	0	0	0	0	1
**Subtotal**	**0**	**1**	**0**	**0**	**0**	**0**	**0**	**0**	**0**	**0**	**0**	**0**	**2452**
**Burkholderia cepacia complex/bla OXA-48**													
Não Detectável	0	1	0	0	0	0	0	0	0	0	0	0	1
**Subtotal**	**0**	**1**	**0**	**0**	**0**	**0**	**0**	**0**	**0**	**0**	**0**	**0**	**2453**
**Burkholderia cepacia complex/bla SPM**													
Não Detectável	0	1	0	0	0	0	0	0	0	0	0	0	1
**Subtotal**	**0**	**1**	**0**	**0**	**0**	**0**	**0**	**0**	**0**	**0**	**0**	**0**	**2454**
**Burkholderia cepacia complex/blaVIM**													
Não Detectável	0	1	0	0	0	0	0	0	0	0	0	0	1
**Subtotal**	**0**	**1**	**0**	**0**	**0**	**0**	**0**	**0**	**0**	**0**	**0**	**0**	**2455**
**Cedecea lapagei/bla KPC**													
Não Detectável	0	0	0	0	0	0	0	0	0	0	0	1	1
**Subtotal**	**0**	**0**	**0**	**0**	**0**	**0**	**0**	**0**	**0**	**0**	**0**	**1**	**2456**
**Cedecea lapagei/bla NDM**													
Detectável	0	0	0	0	0	0	0	0	0	0	0	1	1
**Subtotal**	**0**	**0**	**0**	**0**	**0**	**0**	**0**	**0**	**0**	**0**	**0**	**1**	**2457**
**Cedecea lapagei/bla OXA-48**													
Não Detectável	0	0	0	0	0	0	0	0	0	0	0	1	1
**Subtotal**	**0**	**0**	**0**	**0**	**0**	**0**	**0**	**0**	**0**	**0**	**0**	**1**	**2458**
**Cedecea lapagei/mcr-1**													

Relatório Pesquisa de Genes de Resistência

Microrganismo /Gene Pesquisado	Jan/2021	Fev/2021	Mar/2021	Abr/2021	Maio/2021	Jun/2021	Jul/2021	Ago/2021	Set/2021	Out/2021	Nov/2021	Dez/2021	Total
Não Detectável	0	0	0	0	0	0	0	0	0	0	0	1	1
**Subtotal**	**0**	**0**	**0**	**0**	**0**	**0**	**0**	**0**	**0**	**0**	**0**	**1**	**2459**
**Citrobacter braakii/bla IMP**													
Não Detectável	0	0	0	0	1	0	0	0	0	0	0	0	1
**Subtotal**	**0**	**0**	**0**	**0**	**1**	**0**	**0**	**0**	**0**	**0**	**0**	**0**	**2460**
**Citrobacter braakii/bla KPC**													
Não Detectável	0	0	0	0	1	0	0	0	0	0	0	0	1
**Subtotal**	**0**	**0**	**0**	**0**	**1**	**0**	**0**	**0**	**0**	**0**	**0**	**0**	**2461**
**Citrobacter braakii/bla NDM**													
Não Detectável	0	0	0	0	1	0	0	0	0	0	0	0	1
**Subtotal**	**0**	**0**	**0**	**0**	**1**	**0**	**0**	**0**	**0**	**0**	**0**	**0**	**2462**
**Citrobacter braakii/bla OXA-48**													
Não Detectável	0	0	0	0	1	0	0	0	0	0	0	0	1
**Subtotal**	**0**	**0**	**0**	**0**	**1**	**0**	**0**	**0**	**0**	**0**	**0**	**0**	**2463**
**Citrobacter braakii/bla SPM**													
Não Detectável	0	0	0	0	1	0	0	0	0	0	0	0	1
**Subtotal**	**0**	**0**	**0**	**0**	**1**	**0**	**0**	**0**	**0**	**0**	**0**	**0**	**2464**
**Citrobacter braakii/blaVIM**													
Não Detectável	0	0	0	0	1	0	0	0	0	0	0	0	1
**Subtotal**	**0**	**0**	**0**	**0**	**1**	**0**	**0**	**0**	**0**	**0**	**0**	**0**	**2465**
**Citrobacter freundii/bla IMP**													
Não Detectável	0	2	0	0	0	0	0	0	0	0	0	0	2
**Subtotal**	**0**	**2**	**0**	**0**	**0**	**0**	**0**	**0**	**0**	**0**	**0**	**0**	**2467**
**Citrobacter freundii/bla KPC**													
Não Detectável	0	2	0	0	0	0	0	0	0	0	0	0	2
**Subtotal**	**0**	**2**	**0**	**0**	**0**	**0**	**0**	**0**	**0**	**0**	**0**	**0**	**2469**
**Citrobacter freundii/bla NDM**													
Não Detectável	0	2	0	0	0	0	0	0	0	0	0	0	2
**Subtotal**	**0**	**2**	**0**	**0**	**0**	**0**	**0**	**0**	**0**	**0**	**0**	**0**	**2471**
**Citrobacter freundii/bla OXA-48**													
Não Detectável	0	1	0	0	0	0	0	0	0	0	0	0	1
**Subtotal**	**0**	**1**	**0**	**0**	**0**	**0**	**0**	**0**	**0**	**0**	**0**	**0**	**2472**
**Citrobacter freundii/bla SPM**													
Não Detectável	0	2	0	0	0	0	0	0	0	0	0	0	2
**Subtotal**	**0**	**2**	**0**	**0**	**0**	**0**	**0**	**0**	**0**	**0**	**0**	**0**	**2474**
**Citrobacter freundii/blaVIM**													

Relatório Pesquisa de Genes de Resistência

Microrganismo /Gene Pesquisado	Jan/2021	Fev/2021	Mar/2021	Abr/2021	Maio/2021	Jun/2021	Jul/2021	Ago/2021	Set/2021	Out/2021	Nov/2021	Dez/2021	Total
Não Detectável	0	2	0	0	0	0	0	0	0	0	0	0	2
**Subtotal**	**0**	**2**	**0**	**0**	**0**	**0**	**0**	**0**	**0**	**0**	**0**	**0**	**2476**
**Citrobacter koseri/bla IMP**													
Não Detectável	0	0	0	0	1	0	0	0	0	0	0	0	1
**Subtotal**	**0**	**0**	**0**	**0**	**1**	**0**	**0**	**0**	**0**	**0**	**0**	**0**	**2477**
**Citrobacter koseri/bla KPC**													
Não Detectável	0	0	0	0	1	0	0	0	0	0	0	0	1
**Subtotal**	**0**	**0**	**0**	**0**	**1**	**0**	**0**	**0**	**0**	**0**	**0**	**0**	**2478**
**Citrobacter koseri/bla NDM**													
Não Detectável	0	0	0	0	1	0	0	0	0	0	0	0	1
**Subtotal**	**0**	**0**	**0**	**0**	**1**	**0**	**0**	**0**	**0**	**0**	**0**	**0**	**2479**
**Citrobacter koseri/bla OXA-48**													
Não Detectável	0	0	0	0	1	0	0	0	0	0	0	0	1
**Subtotal**	**0**	**0**	**0**	**0**	**1**	**0**	**0**	**0**	**0**	**0**	**0**	**0**	**2480**
**Citrobacter koseri/bla SPM**													
Não Detectável	0	0	0	0	1	0	0	0	0	0	0	0	1
**Subtotal**	**0**	**0**	**0**	**0**	**1**	**0**	**0**	**0**	**0**	**0**	**0**	**0**	**2481**
**Citrobacter koseri/blaVIM**													
Não Detectável	0	0	0	0	1	0	0	0	0	0	0	0	1
**Subtotal**	**0**	**0**	**0**	**0**	**1**	**0**	**0**	**0**	**0**	**0**	**0**	**0**	**2482**
**Enterobacter cancerogenus/bla IMP**													
Não Detectável	0	0	1	0	0	0	0	0	0	0	0	0	1
**Subtotal**	**0**	**0**	**1**	**0**	**0**	**0**	**0**	**0**	**0**	**0**	**0**	**0**	**2483**
**Enterobacter cancerogenus/bla KPC**													
Não Detectável	0	0	1	0	0	0	0	0	0	0	0	0	1
**Subtotal**	**0**	**0**	**1**	**0**	**0**	**0**	**0**	**0**	**0**	**0**	**0**	**0**	**2484**
**Enterobacter cancerogenus/bla NDM**													
Não Detectável	0	0	1	0	0	0	0	0	0	0	0	0	1
**Subtotal**	**0**	**0**	**1**	**0**	**0**	**0**	**0**	**0**	**0**	**0**	**0**	**0**	**2485**
**Enterobacter cancerogenus/bla OXA-48**													
Não Detectável	0	0	1	0	0	0	0	0	0	0	0	0	1
**Subtotal**	**0**	**0**	**1**	**0**	**0**	**0**	**0**	**0**	**0**	**0**	**0**	**0**	**2486**
**Enterobacter cancerogenus/bla SPM**													
Não Detectável	0	0	1	0	0	0	0	0	0	0	0	0	1
**Subtotal**	**0**	**0**	**1**	**0**	**0**	**0**	**0**	**0**	**0**	**0**	**0**	**0**	**2487**
**Enterobacter cancerogenus/blaVIM**													

Relatório Pesquisa de Genes de Resistência

Microrganismo /Gene Pesquisado	Jan/2021	Fev/2021	Mar/2021	Abr/2021	Maio/2021	Jun/2021	Jul/2021	Ago/2021	Set/2021	Out/2021	Nov/2021	Dez/2021	Total
Não Detectável	0	0	1	0	0	0	0	0	0	0	0	0	1
**Subtotal**	**0**	**0**	**1**	**0**	**0**	**0**	**0**	**0**	**0**	**0**	**0**	**0**	**2488**
**Enterobacter cloacae/bla IMP**													
Não Detectável	0	3	0	0	2	4	0	0	0	0	0	0	9
**Subtotal**	**0**	**3**	**0**	**0**	**2**	**4**	**0**	**0**	**0**	**0**	**0**	**0**	**2497**
**Enterobacter cloacae/bla KPC**													
Detectável	0	0	0	0	0	0	0	0	0	0	0	2	2
Não Detectável	0	3	0	1	2	5	0	0	0	0	0	0	11
**Subtotal**	**0**	**3**	**0**	**1**	**2**	**5**	**0**	**0**	**0**	**0**	**0**	**2**	**2510**
**Enterobacter cloacae/bla NDM**													
Não Detectável	0	3	0	1	2	3	0	0	0	0	0	2	11
**Subtotal**	**0**	**3**	**0**	**1**	**2**	**3**	**0**	**0**	**0**	**0**	**0**	**2**	**2521**
**Enterobacter cloacae/bla OXA-143**													
Não Detectável	0	0	0	0	0	1	0	0	0	0	0	0	1
**Subtotal**	**0**	**0**	**0**	**0**	**0**	**1**	**0**	**0**	**0**	**0**	**0**	**0**	**2522**
**Enterobacter cloacae/bla OXA-23**													
Não Detectável	0	0	0	0	0	1	0	0	0	0	0	0	1
**Subtotal**	**0**	**0**	**0**	**0**	**0**	**1**	**0**	**0**	**0**	**0**	**0**	**0**	**2523**
**Enterobacter cloacae/bla OXA-48**													
Não Detectável	0	2	0	1	2	2	0	0	0	0	0	2	9
**Subtotal**	**0**	**2**	**0**	**1**	**2**	**2**	**0**	**0**	**0**	**0**	**0**	**2**	**2532**
**Enterobacter cloacae/bla OXA-51**													
Não Detectável	0	0	0	0	0	1	0	0	0	0	0	0	1
**Subtotal**	**0**	**0**	**0**	**0**	**0**	**1**	**0**	**0**	**0**	**0**	**0**	**0**	**2533**
**Enterobacter cloacae/bla SPM**													
Não Detectável	0	2	0	1	2	5	0	0	0	0	0	0	10
**Subtotal**	**0**	**2**	**0**	**1**	**2**	**5**	**0**	**0**	**0**	**0**	**0**	**0**	**2543**
**Enterobacter cloacae/blaVIM**													
Não Detectável	0	2	0	1	2	2	0	0	0	0	0	0	7
**Subtotal**	**0**	**2**	**0**	**1**	**2**	**2**	**0**	**0**	**0**	**0**	**0**	**0**	**2550**
**Enterobacter cloacae/mcr-1**													
Não Detectável	0	0	0	0	0	0	0	0	0	0	0	2	2
**Subtotal**	**0**	**0**	**0**	**0**	**0**	**0**	**0**	**0**	**0**	**0**	**0**	**2**	**2552**
**Enterococcus faecium/vanA**													
Detectável	0	0	0	0	0	0	0	0	0	0	0	1	1
**Subtotal**	**0**	**0**	**0**	**0**	**0**	**0**	**0**	**0**	**0**	**0**	**0**	**1**	**2553**

Relatório Pesquisa de Genes de Resistência

Microrganismo /Gene Pesquisado	Jan/2021	Fev/2021	Mar/2021	Abr/2021	Maio/2021	Jun/2021	Jul/2021	Ago/2021	Set/2021	Out/2021	Nov/2021	Dez/2021	Total
**Enterococcus faecium/vanB**													
Não Detectável	0	0	0	0	0	0	0	0	0	0	0	1	1
**Subtotal**	**0**	**0**	**0**	**0**	**0**	**0**	**0**	**0**	**0**	**0**	**0**	**1**	**2554**
**Escherichia coli/bla IMP**													
Não Detectável	0	3	6	4	12	11	1	0	0	0	0	0	37
**Subtotal**	**0**	**3**	**6**	**4**	**12**	**11**	**1**	**0**	**0**	**0**	**0**	**0**	**2591**
**Escherichia coli/bla KPC**													
Detectável	0	0	0	1	0	0	0	0	0	0	0	0	1
Não Detectável	0	3	6	10	13	12	1	0	0	4	3	1	53
**Subtotal**	**0**	**3**	**6**	**11**	**13**	**12**	**1**	**0**	**0**	**4**	**3**	**1**	**2645**
**Escherichia coli/bla NDM**													
Detectável	0	0	1	0	0	0	0	0	0	0	0	0	1
Não Detectável	0	3	4	10	13	12	1	0	0	4	3	1	51
**Subtotal**	**0**	**3**	**5**	**10**	**13**	**12**	**1**	**0**	**0**	**4**	**3**	**1**	**2697**
**Escherichia coli/bla OXA-143**													
Não Detectável	0	0	0	0	0	1	0	0	0	0	0	0	1
**Subtotal**	**0**	**0**	**0**	**0**	**0**	**1**	**0**	**0**	**0**	**0**	**0**	**0**	**2698**
**Escherichia coli/bla OXA-23**													
Não Detectável	0	0	0	0	1	5	0	0	0	0	0	0	6
**Subtotal**	**0**	**0**	**0**	**0**	**1**	**5**	**0**	**0**	**0**	**0**	**0**	**0**	**2704**
**Escherichia coli/bla OXA-48**													
Detectável	0	0	0	0	0	1	0	0	0	0	0	0	1
Não Detectável	0	3	5	11	13	10	1	0	0	4	3	1	51
**Subtotal**	**0**	**3**	**5**	**11**	**13**	**11**	**1**	**0**	**0**	**4**	**3**	**1**	**2756**
**Escherichia coli/bla OXA-51**													
Detectável	0	0	0	0	0	1	0	0	0	0	0	0	1
Não Detectável	0	0	0	0	1	4	0	0	0	0	0	0	5
**Subtotal**	**0**	**0**	**0**	**0**	**1**	**5**	**0**	**0**	**0**	**0**	**0**	**0**	**2762**
**Escherichia coli/bla OXA-58**													
Não Detectável	0	0	0	0	1	4	0	0	0	0	0	0	5
**Subtotal**	**0**	**0**	**0**	**0**	**1**	**4**	**0**	**0**	**0**	**0**	**0**	**0**	**2767**
**Escherichia coli/bla SPM**													
Não Detectável	0	3	6	10	14	12	1	0	0	0	0	0	46
**Subtotal**	**0**	**3**	**6**	**10**	**14**	**12**	**1**	**0**	**0**	**0**	**0**	**0**	**2813**
**Escherichia coli/blaVIM**													
Não Detectável	0	3	5	11	13	11	1	0	0	0	0	0	44

Relatório Pesquisa de Genes de Resistência

Microrganismo /Gene Pesquisado	Jan/2021	Fev/2021	Mar/2021	Abr/2021	Maio/2021	Jun/2021	Jul/2021	Ago/2021	Set/2021	Out/2021	Nov/2021	Dez/2021	Total
**Subtotal**	**0**	**3**	**5**	**11**	**13**	**11**	**1**	**0**	**0**	**0**	**0**	**0**	**2857**
**Escherichia coli/ipa H**													
Não Detectável	0	1	0	0	0	0	0	0	0	0	0	0	1
**Subtotal**	**0**	**1**	**0**	**0**	**0**	**0**	**0**	**0**	**0**	**0**	**0**	**0**	**2858**
**Escherichia coli/mcr-1**													
Não Detectável	0	0	0	0	0	0	0	0	0	4	3	1	8
**Subtotal**	**0**	**0**	**0**	**0**	**0**	**0**	**0**	**0**	**0**	**4**	**3**	**1**	**2866**
**Escherichia coli/outros**													
Não Detectável	0	1	0	0	0	0	0	0	0	0	0	0	1
**Subtotal**	**0**	**1**	**0**	**0**	**0**	**0**	**0**	**0**	**0**	**0**	**0**	**0**	**2867**
**Klebsiella aerogenes/bla IMP**													
Não Detectável	0	0	1	0	1	0	0	0	0	0	0	0	2
**Subtotal**	**0**	**0**	**1**	**0**	**1**	**0**	**0**	**0**	**0**	**0**	**0**	**0**	**2869**
**Klebsiella aerogenes/bla KPC**													
Não Detectável	0	0	2	2	1	0	0	0	0	0	1	1	7
**Subtotal**	**0**	**0**	**2**	**2**	**1**	**0**	**0**	**0**	**0**	**0**	**1**	**1**	**2876**
**Klebsiella aerogenes/bla NDM**													
Não Detectável	0	0	2	2	1	0	0	0	0	0	1	1	7
**Subtotal**	**0**	**0**	**2**	**2**	**1**	**0**	**0**	**0**	**0**	**0**	**1**	**1**	**2883**
**Klebsiella aerogenes/bla OXA-48**													
Não Detectável	0	0	1	2	1	0	0	0	0	0	1	1	6
**Subtotal**	**0**	**0**	**1**	**2**	**1**	**0**	**0**	**0**	**0**	**0**	**1**	**1**	**2889**
**Klebsiella aerogenes/bla SPM**													
Não Detectável	0	0	2	2	1	0	0	0	0	0	0	0	5
**Subtotal**	**0**	**0**	**2**	**2**	**1**	**0**	**0**	**0**	**0**	**0**	**0**	**0**	**2894**
**Klebsiella aerogenes/blaVIM**													
Não Detectável	0	0	1	2	1	0	0	0	0	0	0	0	4
**Subtotal**	**0**	**0**	**1**	**2**	**1**	**0**	**0**	**0**	**0**	**0**	**0**	**0**	**2898**
**Klebsiella aerogenes/mcr-1**													
Não Detectável	0	0	0	0	0	0	0	0	0	0	1	1	2
**Subtotal**	**0**	**0**	**0**	**0**	**0**	**0**	**0**	**0**	**0**	**0**	**1**	**1**	**2900**
**Klebsiella oxytoca/bla IMP**													
Não Detectável	0	0	1	0	1	0	1	0	0	0	0	0	3
**Subtotal**	**0**	**0**	**1**	**0**	**1**	**0**	**1**	**0**	**0**	**0**	**0**	**0**	**2903**
**Klebsiella oxytoca/bla KPC**													
Não Detectável	0	0	1	0	1	0	1	0	0	0	0	0	3

Relatório Pesquisa de Genes de Resistência

Microrganismo /Gene Pesquisado	Jan/2021	Fev/2021	Mar/2021	Abr/2021	Maio/2021	Jun/2021	Jul/2021	Ago/2021	Set/2021	Out/2021	Nov/2021	Dez/2021	Total
**Subtotal**	**0**	**0**	**1**	**0**	**1**	**0**	**1**	**0**	**0**	**0**	**0**	**0**	**2906**
**Klebsiella oxytoca/bla NDM**													
Não Detectável	0	0	1	0	1	0	1	0	0	0	0	0	3
**Subtotal**	**0**	**0**	**1**	**0**	**1**	**0**	**1**	**0**	**0**	**0**	**0**	**0**	**2909**
**Klebsiella oxytoca/bla OXA-23**													
Não Detectável	0	0	0	0	1	0	0	0	0	0	0	0	1
**Subtotal**	**0**	**0**	**0**	**0**	**1**	**0**	**0**	**0**	**0**	**0**	**0**	**0**	**2910**
**Klebsiella oxytoca/bla OXA-48**													
Não Detectável	0	0	1	0	1	0	1	0	0	0	0	0	3
**Subtotal**	**0**	**0**	**1**	**0**	**1**	**0**	**1**	**0**	**0**	**0**	**0**	**0**	**2913**
**Klebsiella oxytoca/bla OXA-51**													
Não Detectável	0	0	0	0	1	0	0	0	0	0	0	0	1
**Subtotal**	**0**	**0**	**0**	**0**	**1**	**0**	**0**	**0**	**0**	**0**	**0**	**0**	**2914**
**Klebsiella oxytoca/bla OXA-58**													
Não Detectável	0	0	0	0	1	0	0	0	0	0	0	0	1
**Subtotal**	**0**	**0**	**0**	**0**	**1**	**0**	**0**	**0**	**0**	**0**	**0**	**0**	**2915**
**Klebsiella oxytoca/bla SPM**													
Não Detectável	0	0	1	0	1	0	1	0	0	0	0	0	3
**Subtotal**	**0**	**0**	**1**	**0**	**1**	**0**	**1**	**0**	**0**	**0**	**0**	**0**	**2918**
**Klebsiella oxytoca/blaVIM**													
Não Detectável	0	0	1	0	1	0	1	0	0	0	0	0	3
**Subtotal**	**0**	**0**	**1**	**0**	**1**	**0**	**1**	**0**	**0**	**0**	**0**	**0**	**2921**
**Klebsiella ozaenae/bla IMP**													
Não Detectável	0	0	0	0	0	5	0	1	0	0	0	0	6
**Subtotal**	**0**	**0**	**0**	**0**	**0**	**5**	**0**	**1**	**0**	**0**	**0**	**0**	**2927**
**Klebsiella ozaenae/bla KPC**													
Detectável	0	0	0	0	0	1	0	1	0	0	0	0	2
Não Detectável	0	0	0	1	0	4	0	0	0	0	0	0	5
**Subtotal**	**0**	**0**	**0**	**1**	**0**	**5**	**0**	**1**	**0**	**0**	**0**	**0**	**2934**
**Klebsiella ozaenae/bla NDM**													
Não Detectável	0	0	0	1	0	5	0	1	0	0	0	0	7
**Subtotal**	**0**	**0**	**0**	**1**	**0**	**5**	**0**	**1**	**0**	**0**	**0**	**0**	**2941**
**Klebsiella ozaenae/bla OXA-48**													
Não Detectável	0	0	0	1	0	5	0	1	0	0	0	0	7
**Subtotal**	**0**	**0**	**0**	**1**	**0**	**5**	**0**	**1**	**0**	**0**	**0**	**0**	**2948**
**Klebsiella ozaenae/bla SPM**													

Relatório Pesquisa de Genes de Resistência

Microrganismo /Gene Pesquisado	Jan/2021	Fev/2021	Mar/2021	Abr/2021	Maio/2021	Jun/2021	Jul/2021	Ago/2021	Set/2021	Out/2021	Nov/2021	Dez/2021	Total
Não Detectável	0	0	0	1	0	5	0	1	0	0	0	0	7
**Subtotal**	**0**	**0**	**0**	**1**	**0**	**5**	**0**	**1**	**0**	**0**	**0**	**0**	**2955**
**Klebsiella ozaenae/blaVIM**													
Não Detectável	0	0	0	1	0	5	0	1	0	0	0	0	7
**Subtotal**	**0**	**0**	**0**	**1**	**0**	**5**	**0**	**1**	**0**	**0**	**0**	**0**	**2962**
**Klebsiella pneumoniae/bla IMP**													
Detectável	0	1	0	0	0	0	0	0	0	0	0	0	1
Não Detectável	0	46	44	61	70	47	19	5	0	0	0	0	292
**Subtotal**	**0**	**47**	**44**	**61**	**70**	**47**	**19**	**5**	**0**	**0**	**0**	**0**	**3255**
**Klebsiella pneumoniae/bla KPC**													
Detectável	0	18	14	17	14	12	7	3	0	3	5	10	103
Inconclusivo	0	0	0	0	1	0	0	0	0	0	0	0	1
Não Detectável	0	49	31	77	63	36	21	2	0	5	8	8	300
**Subtotal**	**0**	**67**	**45**	**94**	**78**	**48**	**28**	**5**	**0**	**8**	**13**	**18**	**3659**
**Klebsiella pneumoniae/bla NDM**													
Detectável	0	0	0	1	0	0	0	0	0	0	0	0	1
Não Detectável	0	47	45	92	72	47	28	5	0	8	13	18	375
**Subtotal**	**0**	**47**	**45**	**93**	**72**	**47**	**28**	**5**	**0**	**8**	**13**	**18**	**4035**
**Klebsiella pneumoniae/bla OXA-143**													
Não Detectável	0	0	0	0	0	11	0	0	0	0	0	0	11
**Subtotal**	**0**	**0**	**0**	**0**	**0**	**11**	**0**	**0**	**0**	**0**	**0**	**0**	**4046**
**Klebsiella pneumoniae/bla OXA-23**													
Detectável	0	0	0	0	1	1	0	0	0	0	0	0	2
Não Detectável	0	0	0	0	23	28	0	0	0	0	0	0	51
**Subtotal**	**0**	**0**	**0**	**0**	**24**	**29**	**0**	**0**	**0**	**0**	**0**	**0**	**4099**
**Klebsiella pneumoniae/bla OXA-48**													
Detectado traços	0	0	0	0	0	3	0	0	0	0	0	0	3
Detectável	0	0	0	0	0	6	0	0	0	0	0	0	6
Não Detectável	0	43	45	92	69	37	30	5	0	8	13	18	360
**Subtotal**	**0**	**43**	**45**	**92**	**69**	**46**	**30**	**5**	**0**	**8**	**13**	**18**	**4468**
**Klebsiella pneumoniae/bla OXA-51**													
Detectável	0	0	0	0	1	0	0	0	0	0	0	0	1
Não Detectável	0	0	0	0	23	29	0	0	0	0	0	0	52
**Subtotal**	**0**	**0**	**0**	**0**	**24**	**29**	**0**	**0**	**0**	**0**	**0**	**0**	**4521**
**Klebsiella pneumoniae/bla OXA-58**													
Não Detectável	0	0	0	0	23	18	0	0	0	0	0	0	41

Relatório Pesquisa de Genes de Resistência

Microrganismo /Gene Pesquisado	Jan/2021	Fev/2021	Mar/2021	Abr/2021	Maio/2021	Jun/2021	Jul/2021	Ago/2021	Set/2021	Out/2021	Nov/2021	Dez/2021	Total
**Subtotal**	**0**	**0**	**0**	**0**	**23**	**18**	**0**	**0**	**0**	**0**	**0**	**0**	**4562**
**Klebsiella pneumoniae/bla SPM**													
Não Detectável	0	47	43	95	71	47	28	5	0	0	0	0	336
**Subtotal**	**0**	**47**	**43**	**95**	**71**	**47**	**28**	**5**	**0**	**0**	**0**	**0**	**4898**
**Klebsiella pneumoniae/blaVIM**													
Não Detectável	0	43	44	91	67	44	28	5	0	0	0	0	322
**Subtotal**	**0**	**43**	**44**	**91**	**67**	**44**	**28**	**5**	**0**	**0**	**0**	**0**	**5220**
**Klebsiella pneumoniae/IMP**													
Não Detectável	0	0	0	0	0	0	1	0	0	0	0	0	1
**Subtotal**	**0**	**0**	**0**	**0**	**0**	**0**	**1**	**0**	**0**	**0**	**0**	**0**	**5221**
**Klebsiella pneumoniae/KPC**													
Não Detectável	0	0	0	0	0	0	2	0	0	0	0	0	2
**Subtotal**	**0**	**0**	**0**	**0**	**0**	**0**	**2**	**0**	**0**	**0**	**0**	**0**	**5223**
**Klebsiella pneumoniae/mcr-1**													
Não Detectável	0	0	0	0	0	0	0	0	0	8	13	18	39
**Subtotal**	**0**	**0**	**0**	**0**	**0**	**0**	**0**	**0**	**0**	**8**	**13**	**18**	**5262**
**Klebsiella pneumoniae/NDM**													
Não Detectável	0	0	0	0	0	0	2	0	0	0	0	0	2
**Subtotal**	**0**	**0**	**0**	**0**	**0**	**0**	**2**	**0**	**0**	**0**	**0**	**0**	**5264**
**Klebsiella pneumoniae/outros**													
Não Detectável	0	0	1	0	0	0	0	0	0	0	0	0	1
**Subtotal**	**0**	**0**	**1**	**0**	**0**	**0**	**0**	**0**	**0**	**0**	**0**	**0**	**5265**
**Klebsiella pneumoniae/SPM**													
Não Detectável	0	0	0	0	0	0	2	0	0	0	0	0	2
**Subtotal**	**0**	**0**	**0**	**0**	**0**	**0**	**2**	**0**	**0**	**0**	**0**	**0**	**5267**
**Klebsiella pneumoniae/VIM**													
Não Detectável	0	0	1	0	0	0	2	0	0	0	0	0	3
**Subtotal**	**0**	**0**	**1**	**0**	**0**	**0**	**2**	**0**	**0**	**0**	**0**	**0**	**5270**
**Klebsiella sp./bla IMP**													
Não Detectável	0	3	0	0	0	0	0	0	0	0	0	0	3
**Subtotal**	**0**	**3**	**0**	**0**	**0**	**0**	**0**	**0**	**0**	**0**	**0**	**0**	**5273**
**Klebsiella sp./bla KPC**													
Detectável	0	2	0	0	0	0	1	0	0	0	0	0	3
Não Detectável	0	2	0	0	0	0	5	0	0	0	0	0	7
**Subtotal**	**0**	**4**	**0**	**0**	**0**	**0**	**6**	**0**	**0**	**0**	**0**	**0**	**5283**
**Klebsiella sp./bla NDM**													

Relatório Pesquisa de Genes de Resistência

Microrganismo /Gene Pesquisado	Jan/2021	Fev/2021	Mar/2021	Abr/2021	Maio/2021	Jun/2021	Jul/2021	Ago/2021	Set/2021	Out/2021	Nov/2021	Dez/2021	Total
Não Detectável	0	3	0	0	0	0	6	0	0	0	0	0	9
**Subtotal**	**0**	**3**	**0**	**0**	**0**	**0**	**6**	**0**	**0**	**0**	**0**	**0**	**5292**
**Klebsiella sp./bla OXA-48**													
Não Detectável	0	3	0	0	0	0	6	0	0	0	0	0	9
**Subtotal**	**0**	**3**	**0**	**0**	**0**	**0**	**6**	**0**	**0**	**0**	**0**	**0**	**5301**
**Klebsiella sp./bla SPM**													
Não Detectável	0	3	0	0	0	0	6	0	0	0	0	0	9
**Subtotal**	**0**	**3**	**0**	**0**	**0**	**0**	**6**	**0**	**0**	**0**	**0**	**0**	**5310**
**Klebsiella sp./blaVIM**													
Não Detectável	0	3	0	0	0	0	6	0	0	0	0	0	9
**Subtotal**	**0**	**3**	**0**	**0**	**0**	**0**	**6**	**0**	**0**	**0**	**0**	**0**	**5319**
**Morganella morganii/bla IMP**													
Não Detectável	0	1	0	0	2	3	2	0	0	0	0	0	8
**Subtotal**	**0**	**1**	**0**	**0**	**2**	**3**	**2**	**0**	**0**	**0**	**0**	**0**	**5327**
**Morganella morganii/bla KPC**													
Não Detectável	0	1	0	2	1	3	2	0	0	0	2	0	11
**Subtotal**	**0**	**1**	**0**	**2**	**1**	**3**	**2**	**0**	**0**	**0**	**2**	**0**	**5338**
**Morganella morganii/bla NDM**													
Não Detectável	0	1	0	2	2	3	2	0	0	0	2	0	12
**Subtotal**	**0**	**1**	**0**	**2**	**2**	**3**	**2**	**0**	**0**	**0**	**2**	**0**	**5350**
**Morganella morganii/bla OXA-23**													
Não Detectável	0	0	0	0	0	1	0	0	0	0	0	0	1
**Subtotal**	**0**	**0**	**0**	**0**	**0**	**1**	**0**	**0**	**0**	**0**	**0**	**0**	**5351**
**Morganella morganii/bla OXA-48**													
Detectável	0	0	0	0	0	2	0	0	0	0	0	0	2
Não Detectável	0	1	0	2	2	1	2	0	0	0	2	0	10
**Subtotal**	**0**	**1**	**0**	**2**	**2**	**3**	**2**	**0**	**0**	**0**	**2**	**0**	**5363**
**Morganella morganii/bla OXA-51**													
Não Detectável	0	0	0	0	0	1	0	0	0	0	0	0	1
**Subtotal**	**0**	**0**	**0**	**0**	**0**	**1**	**0**	**0**	**0**	**0**	**0**	**0**	**5364**
**Morganella morganii/bla OXA-58**													
Não Detectável	0	0	0	0	0	1	0	0	0	0	0	0	1
**Subtotal**	**0**	**0**	**0**	**0**	**0**	**1**	**0**	**0**	**0**	**0**	**0**	**0**	**5365**
**Morganella morganii/bla SPM**													
Não Detectável	0	0	0	2	2	3	2	0	0	0	0	0	9
**Subtotal**	**0**	**0**	**0**	**2**	**2**	**3**	**2**	**0**	**0**	**0**	**0**	**0**	**5374**

Relatório Pesquisa de Genes de Resistência

Microrganismo /Gene Pesquisado	Jan/2021	Fev/2021	Mar/2021	Abr/2021	Maio/2021	Jun/2021	Jul/2021	Ago/2021	Set/2021	Out/2021	Nov/2021	Dez/2021	Total
**Morganella morganii/blaVIM**													
Não Detectável	0	1	0	2	2	3	2	0	0	0	0	0	10
**Subtotal**	**0**	**1**	**0**	**2**	**2**	**3**	**2**	**0**	**0**	**0**	**0**	**0**	**5384**
**Morganella morganii/KPC**													
Não Detectável	0	0	0	0	1	0	0	0	0	0	0	0	1
**Subtotal**	**0**	**0**	**0**	**0**	**1**	**0**	**0**	**0**	**0**	**0**	**0**	**0**	**5385**
**Morganella morganii/mcr-1**													
Não Detectável	0	0	0	0	0	0	0	0	0	0	2	0	2
**Subtotal**	**0**	**0**	**0**	**0**	**0**	**0**	**0**	**0**	**0**	**0**	**2**	**0**	**5387**
**Morganella morganii/SPM**													
Não Detectável	0	1	0	0	0	0	0	0	0	0	0	0	1
**Subtotal**	**0**	**1**	**0**	**0**	**0**	**0**	**0**	**0**	**0**	**0**	**0**	**0**	**5388**
**Pantoea agglomerans/bla KPC**													
Não Detectável	0	0	0	0	0	0	0	0	0	0	0	1	1
**Subtotal**	**0**	**0**	**0**	**0**	**0**	**0**	**0**	**0**	**0**	**0**	**0**	**1**	**5389**
**Pantoea agglomerans/bla NDM**													
Detectável	0	0	0	0	0	0	0	0	0	0	0	1	1
**Subtotal**	**0**	**0**	**0**	**0**	**0**	**0**	**0**	**0**	**0**	**0**	**0**	**1**	**5390**
**Pantoea agglomerans/bla OXA-48**													
Não Detectável	0	0	0	0	0	0	0	0	0	0	0	1	1
**Subtotal**	**0**	**0**	**0**	**0**	**0**	**0**	**0**	**0**	**0**	**0**	**0**	**1**	**5391**
**Pantoea agglomerans/mcr-1**													
Não Detectável	0	0	0	0	0	0	0	0	0	0	0	1	1
**Subtotal**	**0**	**0**	**0**	**0**	**0**	**0**	**0**	**0**	**0**	**0**	**0**	**1**	**5392**
**Proteus mirabilis/bla IMP**													
Não Detectável	0	3	0	0	1	0	0	0	0	0	0	0	4
**Subtotal**	**0**	**3**	**0**	**0**	**1**	**0**	**0**	**0**	**0**	**0**	**0**	**0**	**5396**
**Proteus mirabilis/bla KPC**													
Não Detectável	0	3	0	0	1	0	0	0	0	1	1	2	8
**Subtotal**	**0**	**3**	**0**	**0**	**1**	**0**	**0**	**0**	**0**	**1**	**1**	**2**	**5404**
**Proteus mirabilis/bla NDM**													
Não Detectável	0	3	0	0	1	0	0	0	0	1	1	2	8
**Subtotal**	**0**	**3**	**0**	**0**	**1**	**0**	**0**	**0**	**0**	**1**	**1**	**2**	**5412**
**Proteus mirabilis/bla OXA-23**													
Não Detectável	0	0	0	0	1	0	0	0	0	0	0	0	1
**Subtotal**	**0**	**0**	**0**	**0**	**1**	**0**	**0**	**0**	**0**	**0**	**0**	**0**	**5413**

Relatório Pesquisa de Genes de Resistência

Microrganismo /Gene Pesquisado	Jan/2021	Fev/2021	Mar/2021	Abr/2021	Maio/2021	Jun/2021	Jul/2021	Ago/2021	Set/2021	Out/2021	Nov/2021	Dez/2021	Total
**Proteus mirabilis/bla OXA-48**													
Não Detectável	0	3	0	0	1	0	0	0	0	1	1	2	8
**Subtotal**	**0**	**3**	**0**	**0**	**1**	**0**	**0**	**0**	**0**	**1**	**1**	**2**	**5421**
**Proteus mirabilis/bla OXA-51**													
Não Detectável	0	0	0	0	1	0	0	0	0	0	0	0	1
**Subtotal**	**0**	**0**	**0**	**0**	**1**	**0**	**0**	**0**	**0**	**0**	**0**	**0**	**5422**
**Proteus mirabilis/bla OXA-58**													
Não Detectável	0	0	0	0	1	0	0	0	0	0	0	0	1
**Subtotal**	**0**	**0**	**0**	**0**	**1**	**0**	**0**	**0**	**0**	**0**	**0**	**0**	**5423**
**Proteus mirabilis/bla SPM**													
Não Detectável	0	3	0	0	1	0	0	0	0	0	0	0	4
**Subtotal**	**0**	**3**	**0**	**0**	**1**	**0**	**0**	**0**	**0**	**0**	**0**	**0**	**5427**
**Proteus mirabilis/blaVIM**													
Não Detectável	0	3	0	0	1	0	0	0	0	0	0	0	4
**Subtotal**	**0**	**3**	**0**	**0**	**1**	**0**	**0**	**0**	**0**	**0**	**0**	**0**	**5431**
**Proteus mirabilis/mcr-1**													
Não Detectável	0	0	0	0	0	0	0	0	0	1	1	2	4
**Subtotal**	**0**	**0**	**0**	**0**	**0**	**0**	**0**	**0**	**0**	**1**	**1**	**2**	**5435**
**Proteus sp./bla IMP**													
Não Detectável	0	1	0	0	0	0	0	0	0	0	0	0	1
**Subtotal**	**0**	**1**	**0**	**0**	**0**	**0**	**0**	**0**	**0**	**0**	**0**	**0**	**5436**
**Proteus sp./bla KPC**													
Não Detectável	0	1	0	0	0	0	0	0	0	0	0	0	1
**Subtotal**	**0**	**1**	**0**	**0**	**0**	**0**	**0**	**0**	**0**	**0**	**0**	**0**	**5437**
**Proteus sp./bla NDM**													
Não Detectável	0	1	0	0	0	0	0	0	0	0	0	0	1
**Subtotal**	**0**	**1**	**0**	**0**	**0**	**0**	**0**	**0**	**0**	**0**	**0**	**0**	**5438**
**Proteus sp./bla OXA-48**													
Não Detectável	0	1	0	0	0	0	0	0	0	0	0	0	1
**Subtotal**	**0**	**1**	**0**	**0**	**0**	**0**	**0**	**0**	**0**	**0**	**0**	**0**	**5439**
**Proteus sp./bla SPM**													
Não Detectável	0	1	0	0	0	0	0	0	0	0	0	0	1
**Subtotal**	**0**	**1**	**0**	**0**	**0**	**0**	**0**	**0**	**0**	**0**	**0**	**0**	**5440**
**Proteus sp./blaVIM**													
Não Detectável	0	1	0	0	0	0	0	0	0	0	0	0	1
**Subtotal**	**0**	**1**	**0**	**0**	**0**	**0**	**0**	**0**	**0**	**0**	**0**	**0**	**5441**

Relatório Pesquisa de Genes de Resistência

Microrganismo /Gene Pesquisado	Jan/2021	Fev/2021	Mar/2021	Abr/2021	Maio/2021	Jun/2021	Jul/2021	Ago/2021	Set/2021	Out/2021	Nov/2021	Dez/2021	Total
**Proteus vulgaris/bla KPC**													
Não Detectável	0	0	0	1	0	0	0	0	0	1	0	0	2
**Subtotal**	**0**	**0**	**0**	**1**	**0**	**0**	**0**	**0**	**0**	**1**	**0**	**0**	**5443**
**Proteus vulgaris/bla NDM**													
Não Detectável	0	0	0	1	0	0	0	0	0	1	0	0	2
**Subtotal**	**0**	**0**	**0**	**1**	**0**	**0**	**0**	**0**	**0**	**1**	**0**	**0**	**5445**
**Proteus vulgaris/bla OXA-48**													
Não Detectável	0	0	0	1	0	0	0	0	0	1	0	0	2
**Subtotal**	**0**	**0**	**0**	**1**	**0**	**0**	**0**	**0**	**0**	**1**	**0**	**0**	**5447**
**Proteus vulgaris/bla SPM**													
Não Detectável	0	0	0	1	0	0	0	0	0	0	0	0	1
**Subtotal**	**0**	**0**	**0**	**1**	**0**	**0**	**0**	**0**	**0**	**0**	**0**	**0**	**5448**
**Proteus vulgaris/blaVIM**													
Não Detectável	0	0	0	1	0	0	0	0	0	0	0	0	1
**Subtotal**	**0**	**0**	**0**	**1**	**0**	**0**	**0**	**0**	**0**	**0**	**0**	**0**	**5449**
**Proteus vulgaris/mcr-1**													
Não Detectável	0	0	0	0	0	0	0	0	0	1	0	0	1
**Subtotal**	**0**	**0**	**0**	**0**	**0**	**0**	**0**	**0**	**0**	**1**	**0**	**0**	**5450**
**Providencia rettgeri/bla IMP**													
Não Detectável	0	0	1	0	1	0	0	0	0	0	0	0	2
**Subtotal**	**0**	**0**	**1**	**0**	**1**	**0**	**0**	**0**	**0**	**0**	**0**	**0**	**5452**
**Providencia rettgeri/bla KPC**													
Não Detectável	0	0	1	0	1	1	0	0	0	0	0	0	3
**Subtotal**	**0**	**0**	**1**	**0**	**1**	**1**	**0**	**0**	**0**	**0**	**0**	**0**	**5455**
**Providencia rettgeri/bla NDM**													
Não Detectável	0	0	1	0	1	1	0	0	0	0	0	0	3
**Subtotal**	**0**	**0**	**1**	**0**	**1**	**1**	**0**	**0**	**0**	**0**	**0**	**0**	**5458**
**Providencia rettgeri/bla OXA-23**													
Não Detectável	0	0	0	0	1	0	0	0	0	0	0	0	1
**Subtotal**	**0**	**0**	**0**	**0**	**1**	**0**	**0**	**0**	**0**	**0**	**0**	**0**	**5459**
**Providencia rettgeri/bla OXA-48**													
Detectável	0	0	0	0	0	1	0	0	0	0	0	0	1
Não Detectável	0	0	1	0	1	0	0	0	0	0	0	0	2
**Subtotal**	**0**	**0**	**1**	**0**	**1**	**1**	**0**	**0**	**0**	**0**	**0**	**0**	**5462**
**Providencia rettgeri/bla OXA-51**													
Não Detectável	0	0	0	0	1	0	0	0	0	0	0	0	1

Relatório Pesquisa de Genes de Resistência

Microrganismo /Gene Pesquisado	Jan/2021	Fev/2021	Mar/2021	Abr/2021	Maio/2021	Jun/2021	Jul/2021	Ago/2021	Set/2021	Out/2021	Nov/2021	Dez/2021	Total
**Subtotal**	**0**	**0**	**0**	**0**	**1**	**0**	**0**	**0**	**0**	**0**	**0**	**0**	**5463**
**Providencia rettgeri/bla OXA-58**													
Não Detectável	0	0	0	0	1	0	0	0	0	0	0	0	1
**Subtotal**	**0**	**0**	**0**	**0**	**1**	**0**	**0**	**0**	**0**	**0**	**0**	**0**	**5464**
**Providencia rettgeri/bla SPM**													
Não Detectável	0	0	2	0	1	0	0	0	0	0	0	0	3
**Subtotal**	**0**	**0**	**2**	**0**	**1**	**0**	**0**	**0**	**0**	**0**	**0**	**0**	**5467**
**Providencia rettgeri/blaVIM**													
Não Detectável	0	0	0	0	1	0	0	0	0	0	0	0	1
**Subtotal**	**0**	**0**	**0**	**0**	**1**	**0**	**0**	**0**	**0**	**0**	**0**	**0**	**5468**
**Providencia rustigianii/bla IMP**													
Não Detectável	0	0	0	0	1	0	0	0	0	0	0	0	1
**Subtotal**	**0**	**0**	**0**	**0**	**1**	**0**	**0**	**0**	**0**	**0**	**0**	**0**	**5469**
**Providencia rustigianii/bla KPC**													
Não Detectável	0	0	0	0	1	0	0	0	0	0	0	0	1
**Subtotal**	**0**	**0**	**0**	**0**	**1**	**0**	**0**	**0**	**0**	**0**	**0**	**0**	**5470**
**Providencia rustigianii/bla NDM**													
Não Detectável	0	0	0	0	1	0	0	0	0	0	0	0	1
**Subtotal**	**0**	**0**	**0**	**0**	**1**	**0**	**0**	**0**	**0**	**0**	**0**	**0**	**5471**
**Providencia rustigianii/bla OXA-23**													
Não Detectável	0	0	0	0	1	0	0	0	0	0	0	0	1
**Subtotal**	**0**	**0**	**0**	**0**	**1**	**0**	**0**	**0**	**0**	**0**	**0**	**0**	**5472**
**Providencia rustigianii/bla OXA-48**													
Não Detectável	0	0	0	0	1	0	0	0	0	0	0	0	1
**Subtotal**	**0**	**0**	**0**	**0**	**1**	**0**	**0**	**0**	**0**	**0**	**0**	**0**	**5473**
**Providencia rustigianii/bla OXA-51**													
Não Detectável	0	0	0	0	1	0	0	0	0	0	0	0	1
**Subtotal**	**0**	**0**	**0**	**0**	**1**	**0**	**0**	**0**	**0**	**0**	**0**	**0**	**5474**
**Providencia rustigianii/bla OXA-58**													
Não Detectável	0	0	0	0	1	0	0	0	0	0	0	0	1
**Subtotal**	**0**	**0**	**0**	**0**	**1**	**0**	**0**	**0**	**0**	**0**	**0**	**0**	**5475**
**Providencia rustigianii/bla SPM**													
Não Detectável	0	0	0	0	1	0	0	0	0	0	0	0	1
**Subtotal**	**0**	**0**	**0**	**0**	**1**	**0**	**0**	**0**	**0**	**0**	**0**	**0**	**5476**
**Providencia rustigianii/blaVIM**													
Não Detectável	0	0	0	0	1	0	0	0	0	0	0	0	1

Relatório Pesquisa de Genes de Resistência

Microrganismo /Gene Pesquisado	Jan/2021	Fev/2021	Mar/2021	Abr/2021	Maio/2021	Jun/2021	Jul/2021	Ago/2021	Set/2021	Out/2021	Nov/2021	Dez/2021	Total
**Subtotal**	**0**	**0**	**0**	**0**	**1**	**0**	**0**	**0**	**0**	**0**	**0**	**0**	**5477**
**Providencia stuartii/bla IMP**													
Não Detectável	0	4	0	0	1	0	0	0	0	0	0	0	5
**Subtotal**	**0**	**4**	**0**	**0**	**1**	**0**	**0**	**0**	**0**	**0**	**0**	**0**	**5482**
**Providencia stuartii/bla KPC**													
Não Detectável	0	4	0	0	1	0	0	0	0	0	2	3	10
**Subtotal**	**0**	**4**	**0**	**0**	**1**	**0**	**0**	**0**	**0**	**0**	**2**	**3**	**5492**
**Providencia stuartii/bla NDM**													
Detectável	0	1	0	0	0	0	0	0	0	0	1	3	5
Não Detectável	0	3	0	0	1	0	0	0	0	0	1	0	5
**Subtotal**	**0**	**4**	**0**	**0**	**1**	**0**	**0**	**0**	**0**	**0**	**2**	**3**	**5502**
**Providencia stuartii/bla OXA-23**													
Não Detectável	0	0	0	0	1	0	0	0	0	0	0	0	1
**Subtotal**	**0**	**0**	**0**	**0**	**1**	**0**	**0**	**0**	**0**	**0**	**0**	**0**	**5503**
**Providencia stuartii/bla OXA-48**													
Não Detectável	0	4	0	0	1	0	0	0	0	0	2	3	10
**Subtotal**	**0**	**4**	**0**	**0**	**1**	**0**	**0**	**0**	**0**	**0**	**2**	**3**	**5513**
**Providencia stuartii/bla OXA-51**													
Não Detectável	0	0	0	0	1	0	0	0	0	0	0	0	1
**Subtotal**	**0**	**0**	**0**	**0**	**1**	**0**	**0**	**0**	**0**	**0**	**0**	**0**	**5514**
**Providencia stuartii/bla OXA-58**													
Não Detectável	0	0	0	0	1	0	0	0	0	0	0	0	1
**Subtotal**	**0**	**0**	**0**	**0**	**1**	**0**	**0**	**0**	**0**	**0**	**0**	**0**	**5515**
**Providencia stuartii/bla SPM**													
Não Detectável	0	4	0	0	1	0	0	0	0	0	0	0	5
**Subtotal**	**0**	**4**	**0**	**0**	**1**	**0**	**0**	**0**	**0**	**0**	**0**	**0**	**5520**
**Providencia stuartii/blaVIM**													
Não Detectável	0	3	0	0	1	0	0	0	0	0	0	0	4
**Subtotal**	**0**	**3**	**0**	**0**	**1**	**0**	**0**	**0**	**0**	**0**	**0**	**0**	**5524**
**Providencia stuartii/mcr-1**													
Não Detectável	0	0	0	0	0	0	0	0	0	0	2	3	5
**Subtotal**	**0**	**0**	**0**	**0**	**0**	**0**	**0**	**0**	**0**	**0**	**2**	**3**	**5529**
**Pseudomonas aeruginosa/bla IMP**													
Não Detectável	0	13	11	3	11	14	8	0	0	0	0	1	61
**Subtotal**	**0**	**13**	**11**	**3**	**11**	**14**	**8**	**0**	**0**	**0**	**0**	**1**	**5590**
**Pseudomonas aeruginosa/bla KPC**													

Relatório Pesquisa de Genes de Resistência

Microrganismo /Gene Pesquisado	Jan/2021	Fev/2021	Mar/2021	Abr/2021	Maio/2021	Jun/2021	Jul/2021	Ago/2021	Set/2021	Out/2021	Nov/2021	Dez/2021	Total
Detectável	0	0	0	0	0	0	0	0	0	0	1	0	1
Não Detectável	0	14	11	10	12	15	11	0	0	7	5	10	95
**Subtotal**	**0**	**14**	**11**	**10**	**12**	**15**	**11**	**0**	**0**	**7**	**6**	**10**	**5686**
**Pseudomonas aeruginosa/bla NDM**													
Não Detectável	0	14	9	9	12	14	12	0	0	7	6	10	93
**Subtotal**	**0**	**14**	**9**	**9**	**12**	**14**	**12**	**0**	**0**	**7**	**6**	**10**	**5779**
**Pseudomonas aeruginosa/bla OXA-143**													
Não Detectável	0	0	0	0	0	5	0	0	0	0	0	0	5
**Subtotal**	**0**	**0**	**0**	**0**	**0**	**5**	**0**	**0**	**0**	**0**	**0**	**0**	**5784**
**Pseudomonas aeruginosa/bla OXA-23**													
Não Detectável	0	0	0	0	3	8	0	0	0	0	0	0	11
**Subtotal**	**0**	**0**	**0**	**0**	**3**	**8**	**0**	**0**	**0**	**0**	**0**	**0**	**5795**
**Pseudomonas aeruginosa/bla OXA-48**													
Detectável	0	0	0	0	0	3	0	0	0	0	0	0	3
Não Detectável	0	13	9	10	10	12	10	0	0	7	6	10	87
**Subtotal**	**0**	**13**	**9**	**10**	**10**	**15**	**10**	**0**	**0**	**7**	**6**	**10**	**5885**
**Pseudomonas aeruginosa/bla OXA-51**													
Detectável	0	0	0	0	0	1	0	0	0	0	0	0	1
Não Detectável	0	0	0	0	3	7	0	0	0	0	0	0	10
**Subtotal**	**0**	**0**	**0**	**0**	**3**	**8**	**0**	**0**	**0**	**0**	**0**	**0**	**5896**
**Pseudomonas aeruginosa/bla OXA-58**													
Não Detectável	0	0	0	0	3	3	0	0	0	0	0	0	6
**Subtotal**	**0**	**0**	**0**	**0**	**3**	**3**	**0**	**0**	**0**	**0**	**0**	**0**	**5902**
**Pseudomonas aeruginosa/bla SPM**													
Não Detectável	0	16	11	10	11	14	11	0	0	6	6	10	95
**Subtotal**	**0**	**16**	**11**	**10**	**11**	**14**	**11**	**0**	**0**	**6**	**6**	**10**	**5997**
**Pseudomonas aeruginosa/blaVIM**													
Não Detectável	0	15	9	10	10	14	10	0	0	6	6	10	90
**Subtotal**	**0**	**15**	**9**	**10**	**10**	**14**	**10**	**0**	**0**	**6**	**6**	**10**	**6087**
**Pseudomonas aeruginosa/IMP**													
Não Detectável	0	0	0	1	0	0	0	0	0	0	0	0	1
**Subtotal**	**0**	**0**	**0**	**1**	**0**	**0**	**0**	**0**	**0**	**0**	**0**	**0**	**6088**
**Pseudomonas aeruginosa/KPC**													
Não Detectável	0	0	0	0	0	0	1	0	0	0	0	0	1
**Subtotal**	**0**	**0**	**0**	**0**	**0**	**0**	**1**	**0**	**0**	**0**	**0**	**0**	**6089**
**Pseudomonas aeruginosa/SPM**													

Relatório Pesquisa de Genes de Resistência

Microrganismo /Gene Pesquisado	Jan/2021	Fev/2021	Mar/2021	Abr/2021	Maio/2021	Jun/2021	Jul/2021	Ago/2021	Set/2021	Out/2021	Nov/2021	Dez/2021	Total
Não Detectável	0	0	0	0	0	0	1	0	0	0	0	0	1
**Subtotal**	**0**	**0**	**0**	**0**	**0**	**0**	**1**	**0**	**0**	**0**	**0**	**0**	**6090**
**Pseudomonas fluorescens/bla IMP**													
Não Detectável	0	0	0	0	0	1	0	0	0	0	0	0	1
**Subtotal**	**0**	**0**	**0**	**0**	**0**	**1**	**0**	**0**	**0**	**0**	**0**	**0**	**6091**
**Pseudomonas fluorescens/bla KPC**													
Não Detectável	0	0	0	0	0	1	0	0	0	0	0	0	1
**Subtotal**	**0**	**0**	**0**	**0**	**0**	**1**	**0**	**0**	**0**	**0**	**0**	**0**	**6092**
**Pseudomonas fluorescens/bla SPM**													
Não Detectável	0	0	0	0	0	1	0	0	0	0	0	0	1
**Subtotal**	**0**	**0**	**0**	**0**	**0**	**1**	**0**	**0**	**0**	**0**	**0**	**0**	**6093**
**Pseudomonas putida/bla IMP**													
Não Detectável	0	0	1	0	1	1	0	0	0	0	0	0	3
**Subtotal**	**0**	**0**	**1**	**0**	**1**	**1**	**0**	**0**	**0**	**0**	**0**	**0**	**6096**
**Pseudomonas putida/bla KPC**													
Não Detectável	0	0	1	1	1	1	0	0	0	0	0	0	4
**Subtotal**	**0**	**0**	**1**	**1**	**1**	**1**	**0**	**0**	**0**	**0**	**0**	**0**	**6100**
**Pseudomonas putida/bla NDM**													
Não Detectável	0	0	0	1	1	1	0	0	0	0	0	0	3
**Subtotal**	**0**	**0**	**0**	**1**	**1**	**1**	**0**	**0**	**0**	**0**	**0**	**0**	**6103**
**Pseudomonas putida/bla OXA-48**													
Não Detectável	0	0	0	1	1	1	0	0	0	0	0	0	3
**Subtotal**	**0**	**0**	**0**	**1**	**1**	**1**	**0**	**0**	**0**	**0**	**0**	**0**	**6106**
**Pseudomonas putida/bla SPM**													
Não Detectável	0	0	1	1	1	1	0	0	0	0	0	0	4
**Subtotal**	**0**	**0**	**1**	**1**	**1**	**1**	**0**	**0**	**0**	**0**	**0**	**0**	**6110**
**Pseudomonas putida/blaVIM**													
Não Detectável	0	0	0	1	1	1	0	0	0	0	0	0	3
**Subtotal**	**0**	**0**	**0**	**1**	**1**	**1**	**0**	**0**	**0**	**0**	**0**	**0**	**6113**
**Pseudomonas sp./bla KPC**													
Não Detectável	0	0	0	0	0	0	1	0	0	0	0	0	1
**Subtotal**	**0**	**0**	**0**	**0**	**0**	**0**	**1**	**0**	**0**	**0**	**0**	**0**	**6114**
**Pseudomonas sp./bla NDM**													
Não Detectável	0	0	0	0	0	0	1	0	0	0	0	0	1
**Subtotal**	**0**	**0**	**0**	**0**	**0**	**0**	**1**	**0**	**0**	**0**	**0**	**0**	**6115**
**Pseudomonas sp./bla OXA-48**													

Relatório Pesquisa de Genes de Resistência

Microrganismo /Gene Pesquisado	Jan/2021	Fev/2021	Mar/2021	Abr/2021	Maio/2021	Jun/2021	Jul/2021	Ago/2021	Set/2021	Out/2021	Nov/2021	Dez/2021	Total
Não Detectável	0	0	0	0	0	0	1	0	0	0	0	0	1
**Subtotal**	**0**	**0**	**0**	**0**	**0**	**0**	**1**	**0**	**0**	**0**	**0**	**0**	**6116**
**Pseudomonas sp./bla SPM**													
Não Detectável	0	0	0	0	0	0	1	0	0	0	0	0	1
**Subtotal**	**0**	**0**	**0**	**0**	**0**	**0**	**1**	**0**	**0**	**0**	**0**	**0**	**6117**
**Pseudomonas sp./blaVIM**													
Não Detectável	0	0	0	0	0	0	1	0	0	0	0	0	1
**Subtotal**	**0**	**0**	**0**	**0**	**0**	**0**	**1**	**0**	**0**	**0**	**0**	**0**	**6118**
**Serratia ficaria/bla IMP**													
Não Detectável	0	0	0	0	0	1	0	0	0	0	0	0	1
**Subtotal**	**0**	**0**	**0**	**0**	**0**	**1**	**0**	**0**	**0**	**0**	**0**	**0**	**6119**
**Serratia ficaria/bla KPC**													
Não Detectável	0	0	0	0	0	1	0	0	0	0	0	0	1
**Subtotal**	**0**	**0**	**0**	**0**	**0**	**1**	**0**	**0**	**0**	**0**	**0**	**0**	**6120**
**Serratia ficaria/bla NDM**													
Não Detectável	0	0	0	0	0	1	0	0	0	0	0	0	1
**Subtotal**	**0**	**0**	**0**	**0**	**0**	**1**	**0**	**0**	**0**	**0**	**0**	**0**	**6121**
**Serratia ficaria/bla OXA-48**													
Não Detectável	0	0	0	0	0	1	0	0	0	0	0	0	1
**Subtotal**	**0**	**0**	**0**	**0**	**0**	**1**	**0**	**0**	**0**	**0**	**0**	**0**	**6122**
**Serratia ficaria/bla SPM**													
Não Detectável	0	0	0	0	0	1	0	0	0	0	0	0	1
**Subtotal**	**0**	**0**	**0**	**0**	**0**	**1**	**0**	**0**	**0**	**0**	**0**	**0**	**6123**
**Serratia ficaria/blaVIM**													
Não Detectável	0	0	0	0	0	1	0	0	0	0	0	0	1
**Subtotal**	**0**	**0**	**0**	**0**	**0**	**1**	**0**	**0**	**0**	**0**	**0**	**0**	**6124**
**Serratia marcescens/bla IMP**													
Não Detectável	0	3	1	1	2	6	4	0	0	0	0	0	17
**Subtotal**	**0**	**3**	**1**	**1**	**2**	**6**	**4**	**0**	**0**	**0**	**0**	**0**	**6141**
**Serratia marcescens/bla KPC**													
Não Detectável	0	3	1	3	5	6	4	0	0	0	2	2	26
**Subtotal**	**0**	**3**	**1**	**3**	**5**	**6**	**4**	**0**	**0**	**0**	**2**	**2**	**6167**
**Serratia marcescens/bla NDM**													
Não Detectável	0	3	1	3	5	5	4	0	0	0	2	2	25
**Subtotal**	**0**	**3**	**1**	**3**	**5**	**5**	**4**	**0**	**0**	**0**	**2**	**2**	**6192**
**Serratia marcescens/bla OXA-23**													

Relatório Pesquisa de Genes de Resistência

Microrganismo /Gene Pesquisado	Jan/2021	Fev/2021	Mar/2021	Abr/2021	Maio/2021	Jun/2021	Jul/2021	Ago/2021	Set/2021	Out/2021	Nov/2021	Dez/2021	Total
Não Detectável	0	0	0	0	1	2	0	0	0	0	0	0	3
**Subtotal**	**0**	**0**	**0**	**0**	**1**	**2**	**0**	**0**	**0**	**0**	**0**	**0**	**6195**
**Serratia marcescens/bla OXA-48**													
Não Detectável	0	3	1	2	3	5	3	0	0	0	2	2	21
**Subtotal**	**0**	**3**	**1**	**2**	**3**	**5**	**3**	**0**	**0**	**0**	**2**	**2**	**6216**
**Serratia marcescens/bla OXA-51**													
Não Detectável	0	0	0	0	1	2	0	0	0	0	0	0	3
**Subtotal**	**0**	**0**	**0**	**0**	**1**	**2**	**0**	**0**	**0**	**0**	**0**	**0**	**6219**
**Serratia marcescens/bla OXA-58**													
Não Detectável	0	0	0	0	1	2	0	0	0	0	0	0	3
**Subtotal**	**0**	**0**	**0**	**0**	**1**	**2**	**0**	**0**	**0**	**0**	**0**	**0**	**6222**
**Serratia marcescens/bla SPM**													
Não Detectável	0	3	1	3	5	6	3	0	0	0	0	0	21
**Subtotal**	**0**	**3**	**1**	**3**	**5**	**6**	**3**	**0**	**0**	**0**	**0**	**0**	**6243**
**Serratia marcescens/blaVIM**													
Não Detectável	0	3	1	2	3	5	3	0	0	0	0	0	17
**Subtotal**	**0**	**3**	**1**	**2**	**3**	**5**	**3**	**0**	**0**	**0**	**0**	**0**	**6260**
**Serratia marcescens/mcr-1**													
Não Detectável	0	0	0	0	0	0	0	0	0	0	2	2	4
**Subtotal**	**0**	**0**	**0**	**0**	**0**	**0**	**0**	**0**	**0**	**0**	**2**	**2**	**6264**
**Staphylococcus aureus/mec a**													
Detectável	0	1	0	3	0	0	0	0	0	0	1	3	8
Não Detectável	0	3	0	4	1	0	4	0	0	0	0	0	12
**Subtotal**	**0**	**4**	**0**	**7**	**1**	**0**	**4**	**0**	**0**	**0**	**1**	**3**	**6284**
**Staphylococcus aureus/outros**													
Não Detectável	0	4	0	4	0	0	0	0	0	0	0	0	8
**Subtotal**	**0**	**4**	**0**	**4**	**0**	**0**	**0**	**0**	**0**	**0**	**0**	**0**	**6292**
**Staphylococcus lugdunensis/mec a**													
Detectável	0	0	0	1	0	0	0	0	0	0	0	0	1
**Subtotal**	**0**	**0**	**0**	**1**	**0**	**0**	**0**	**0**	**0**	**0**	**0**	**0**	**6293**
	0	1	0	2	0	2	1	0	0	0	0	0	6
**Subtotal**	**0**	**1**	**0**	**2**	**0**	**2**	**1**	**0**	**0**	**0**	**0**	**0**	**6299**
