# Supplementary material for: Biodiversity of carbapenem-resistant bacteria in clinical samples from the Southwest Amazon region (Rondônia/Brazil)
Source: Sci Rep. 2024 Apr 23;14:9383. doi: 10.1038/s41598-024-59733-w (PMC11039742; doi:10.1038/s41598-024-59733-w)
Supplement: Supplementary file 4 — Supplementary Information 4. [file 41598_2024_59733_MOESM4_ESM.pdf]

Análise de Resistência Bacteriana - Teste de Sensibilidade

Exame: Bactérias, Teste de Sensibilidade
Bactérias, Teste de Sensibilidade II
Cólera, Teste de Sensibilidade
Febre Tifóide, TSA
Meningite Bacteriana, TSA

Metodologia: Teste de Sensibilidade

Data Início: 01/01/2018 **Data Fim:** 31/12/2021

Antibiotico: Ertapenem, Imipenem, Meropenem

Total Exame/Metodologia: 15025

Antibiótico/Microrganismo	Sensível	Intermediário	Resistente	Não Testado	% Resistentes	Total
**Ertapenem**						
Acinetobacter baumannii	5	0	61	0	**92.42%**	66
Acinetobacter haemolyticus	0	0	1	0	**100%**	1
Acinetobacter sp.	3	0	18	0	**85.71%**	21
Alcaligenes faecalis	0	0	1	0	**100%**	1
Burkholderia cepacia	0	0	6	0	**100%**	6
Burkholderia cepacia complex	0	0	7	0	**100%**	7
Cedecea lapagei	0	0	1	0	**100%**	1
Citrobacter braakii	2	0	2	0	**50%**	4
Citrobacter farmeri	2	0	0	0	**0%**	2
Citrobacter freundii	31	1	7	0	**17.95%**	39
Citrobacter koseri	29	0	6	0	**17.14%**	35
Citrobacter sp.	4	0	1	0	**20%**	5
Edwardsiella tarda	2	0	0	0	**0%**	2
Enterobacter aerogenes	42	1	0	0	**0%**	43
Enterobacter asburiae	3	0	1	0	**25%**	4
Enterobacter cancerogenus	1	0	1	0	**50%**	2
Enterobacter cloacae	221	18	86	0	**26.46%**	325
Enterobacter sakazakii	1	0	1	0	**50%**	2
Enterobacter sp.	0	0	2	0	**100%**	2
Enterococcus faecium	1	0	0	0	**0%**	1
Escherichia coli	1823	11	221	0	**10.75%**	2055
Escherichia coli enteroinvasora	1	0	0	0	**0%**	1
Escherichia coli enteropatogênica	1	0	0	0	**0%**	1
Escherichia fergusonii	1	0	0	0	**0%**	1
Escherichia sp.	1	0	0	0	**0%**	1
Hafnia alvei	0	0	1	0	**100%**	1
Kingella denitrificans	0	0	1	0	**100%**	1
Klebsiella aerogenes	39	1	18	0	**31.03%**	58
Klebsiella oxytoca	25	0	15	0	**37.5%**	40
Klebsiella ozaenae	8	1	12	0	**57.14%**	21
Klebsiella planticola	0	0	1	0	**100%**	1
Klebsiella pneumoniae	1108	73	875	0	**42.56%**	2056
Klebsiella pneumoniae subsp. pneumoniae	54	0	16	0	**22.86%**	70
Klebsiella sp.	58	1	30	0	**33.71%**	89
Kluyvera ascorbata	1	0	5	0	**83.33%**	6
Kluyvera sp.	1	0	0	0	**0%**	1
Leclercia adecarboxylata	0	0	1	0	**100%**	1
Morganella morganii	87	0	3	0	**3.33%**	90
Morganella morganii subsp. morganii	7	0	0	0	**0%**	7
Morganella morganii subsp. sibonii	1	0	0	0	**0%**	1
Morganella sp.	1	0	0	0	**0%**	1

Análise de Resistência Bacteriana - Teste de Sensibilidade

Antibiótico/Microrganismo	Sensível	Intermediário	Resistente	Não Testado	% Resistentes	Total
Pantoea agglomerans	6	1	7	0	**50%**	14
Pantoea sp.	1	0	2	0	**66.67%**	3
Pluralibacter gergoviae	1	0	1	0	**50%**	2
Proteus mirabilis	213	1	27	0	**11.2%**	241
Proteus penneri	3	0	0	0	**0%**	3
Proteus sp.	4	0	0	0	**0%**	4
Proteus vulgaris	11	0	1	0	**8.33%**	12
Providencia alcalifaciens	0	0	1	0	**100%**	1
Providencia rettgeri	6	0	3	0	**33.33%**	9
Providencia rustigianii	2	0	0	0	**0%**	2
Providencia stuartii	51	0	23	0	**31.08%**	74
Pseudomonas aeruginosa	6	1	15	0	**68.18%**	22
Pseudomonas sp.	1	0	1	0	**50%**	2
Raoultella ornithinolytica	1	0	0	0	**0%**	1
Salmonella enterica	4	0	1	0	**20%**	5
Salmonella enterica subsp. enterica	1	0	0	0	**0%**	1
Salmonella Enteritidis	2	0	4	0	**66.67%**	6
Salmonella sp.	9	0	0	0	**0%**	9
Serratia ficaria	0	0	2	0	**100%**	2
Serratia liquefaciens	3	0	1	0	**25%**	4
Serratia marcescens	202	3	92	0	**30.98%**	297
Serratia plymuthica	7	0	2	0	**22.22%**	9
Serratia rubidaea	2	0	2	0	**50%**	4
Serratia sp.	7	0	3	0	**30%**	10
Shigella boydii	2	0	0	0	**0%**	2
Shigella flexneri	2	0	0	0	**0%**	2
Shigella sonnei	1	0	0	0	**0%**	1
Staphylococcus aureus	0	0	2	0	**100%**	2
Staphylococcus cohnii subsp. cohnii	0	0	1	0	**100%**	1
Staphylococcus epidermidis	0	0	1	0	**100%**	1
Staphylococcus haemolyticus	0	0	3	0	**100%**	3
Staphylococcus schleiferi	0	0	1	0	**100%**	1
Stenotrophomonas maltophilia	1	0	1	0	**50%**	2
Streptococcus pneumoniae	1	0	0	0	**0%**	1
Streptococcus pyogenes (beta hemolítico grupo A)	1	0	0	0	**0%**	1
Yersinia intermedia	0	0	1	0	**100%**	1
**Subtotal**	**4115**	**113**	**1599**	**0**	**27.44%**	**5827**
**Imipenem**						
Achromobacter sp.	4	1	0	0	**0%**	5
Achromobacter xylosoxidans	7	1	0	0	**0%**	8
Acinetobacter anitratus	0	0	1	0	**100%**	1
Acinetobacter baumannii	430	18	1123	0	**71.48%**	1571
Acinetobacter haemolyticus	2	0	0	0	**0%**	2
Acinetobacter lwoffii	3	0	3	0	**50%**	6
Acinetobacter sp.	26	2	83	0	**74.77%**	111
Aeromonas caviae	1	0	0	0	**0%**	1
Aeromonas hydrophila	0	1	0	0	**0%**	1
Aeromonas hydrophila/Aeromonas caviae	5	0	0	0	**0%**	5
Aeromonas sp.	0	1	0	0	**0%**	1
Alcaligenes faecalis	4	0	1	0	**20%**	5
Burkholderia cepacia	5	1	27	0	**81.82%**	33

Análise de Resistência Bacteriana - Teste de Sensibilidade

Antibiótico/Microrganismo	Sensível	Intermediário	Resistente	Não Testado	% Resistentes	Total
Burkholderia cepacia complex	0	0	1	0	**100%**	1
Cedecea lapagei	0	0	1	0	**100%**	1
Chryseobacterium indologenes	0	0	2	0	**100%**	2
Citrobacter braakii	3	0	1	0	**25%**	4
Citrobacter farmeri	2	0	0	0	**0%**	2
Citrobacter freundii	25	4	5	0	**14.71%**	34
Citrobacter koseri	31	2	2	0	**5.71%**	35
Citrobacter sp.	6	0	0	0	**0%**	6
Delftia acidovorans	0	0	2	0	**100%**	2
Edwardsiella tarda	2	0	0	0	**0%**	2
Elizabethkingia meningoseptica (Chryseobacterium meningosepticum)	0	0	6	0	**100%**	6
Enterobacter aerogenes	36	7	0	0	**0%**	43
Enterobacter asburiae	3	0	1	0	**25%**	4
Enterobacter cancerogenus	2	0	0	0	**0%**	2
Enterobacter cloacae	233	17	60	0	**19.35%**	310
Enterobacter sakazakii	2	0	0	0	**0%**	2
Enterobacter sp.	6	0	3	0	**33.33%**	9
Enterococcus faecalis	1	0	0	0	**0%**	1
Enterococcus sp.	1	0	0	0	**0%**	1
Escherichia coli	1772	11	89	0	**4.75%**	1872
Escherichia coli enteropatogênica clássica B O142	1	0	0	0	**0%**	1
Escherichia fergusonii	1	0	0	0	**0%**	1
Escherichia sp.	1	0	0	0	**0%**	1
Hafnia alvei	1	0	0	0	**0%**	1
Klebsiella aerogenes	29	19	11	0	**18.64%**	59
Klebsiella oxytoca	28	0	13	0	**31.71%**	41
Klebsiella ozaenae	13	1	8	0	**36.36%**	22
Klebsiella planticola	1	0	1	0	**50%**	2
Klebsiella pneumoniae	1300	29	725	0	**35.3%**	2054
Klebsiella pneumoniae subsp. pneumoniae	52	0	15	0	**22.39%**	67
Klebsiella sp.	70	8	31	0	**28.44%**	109
Kluyvera ascorbata	4	1	1	0	**16.67%**	6
Kluyvera sp.	1	0	0	0	**0%**	1
Leclercia adecarboxylata	0	1	0	0	**0%**	1
Morganella morganii	5	4	46	0	**83.64%**	55
Morganella morganii subsp. morganii	1	0	2	0	**66.67%**	3
Ochrobactrum anthropi	1	0	0	0	**0%**	1
Pantoea agglomerans	3	0	0	0	**0%**	3
Pluralibacter gergoviae	1	0	1	0	**50%**	2
Proteus mirabilis	24	3	1	0	**3.57%**	28
Proteus sp.	4	0	0	0	**0%**	4
Proteus vulgaris	1	0	8	0	**88.89%**	9
Providencia alcalifaciens	1	0	0	0	**0%**	1
Providencia rettgeri	0	2	4	0	**66.67%**	6
Providencia rustigianii	1	0	1	0	**50%**	2
Providencia sp.	1	0	0	0	**0%**	1
Providencia stuartii	2	3	49	0	**90.74%**	54
Pseudomonas aeruginosa	538	132	386	0	**36.55%**	1056
Pseudomonas fluorescens	4	0	0	0	**0%**	4

Análise de Resistência Bacteriana - Teste de Sensibilidade

Antibiótico/Microrganismo	Sensível	Intermediário	Resistente	Não Testado	% Resistentes	Total
Pseudomonas luteola	4	0	0	0	**0%**	4
Pseudomonas mendocina	2	0	0	0	**0%**	2
Pseudomonas oryzihabitans	2	0	0	0	**0%**	2
Pseudomonas pseudoalcaligenes	0	0	1	0	**100%**	1
Pseudomonas putida	18	1	3	0	**13.64%**	22
Pseudomonas sp.	12	1	9	0	**40.91%**	22
Pseudomonas stutzeri	2	0	0	0	**0%**	2
Ralstonia pickettii	2	0	0	0	**0%**	2
Raoultella ornithinolytica	1	0	0	0	**0%**	1
Salmonella enterica	2	0	1	0	**33.33%**	3
Salmonella enterica subsp. enterica	1	0	0	0	**0%**	1
Salmonella Enteritidis	1	4	2	0	**28.57%**	7
Salmonella sp.	13	0	0	0	**0%**	13
Serratia ficaria	2	0	0	0	**0%**	2
Serratia liquefaciens	2	1	1	0	**25%**	4
Serratia marcescens	48	52	50	0	**33.33%**	150
Serratia plymuthica	3	6	0	0	**0%**	9
Serratia rubidaea	2	0	2	0	**50%**	4
Serratia sp.	15	1	4	0	**20%**	20
Shewanella putrefaciens	1	0	0	0	**0%**	1
Shigella boydii	1	1	0	0	**0%**	2
Shigella flexneri	1	1	0	0	**0%**	2
Shigella sonnei	1	0	0	0	**0%**	1
Shigella sp.	1	0	0	0	**0%**	1
Sphingobacterium sp.	0	0	2	0	**100%**	2
Sphingobacterium spiritivorum	0	0	1	0	**100%**	1
Sphingomonas paucimobilis	18	0	3	0	**14.29%**	21
Staphylococcus aureus	0	0	1	0	**100%**	1
Stenotrophomonas maltophilia	1	0	2	0	**66.67%**	3
Streptococcus mitis	1	0	0	0	**0%**	1
Streptococcus pneumoniae	1	0	0	0	**0%**	1
Yersinia intermedia	0	0	1	0	**100%**	1
**Subtotal**	**4859**	**337**	**2797**	**0**	**34.99%**	**7993**
**Meropenem**						
Achromobacter denitrificans	1	0	0	0	**0%**	1
Achromobacter xylosoxidans	8	0	0	0	**0%**	8
Acinetobacter anitratus	0	0	1	0	**100%**	1
Acinetobacter baumannii	440	12	1129	0	**71.41%**	1581
Acinetobacter haemolyticus	3	0	0	0	**0%**	3
Acinetobacter lwoffii	2	1	3	0	**50%**	6
Acinetobacter sp.	28	3	78	0	**71.56%**	109
Aeromonas hydrophila	0	0	1	0	**100%**	1
Aeromonas hydrophila/Aeromonas caviae	5	0	2	0	**28.57%**	7
Burkholderia cenocepacia	0	0	1	0	**100%**	1
Burkholderia cepacia	87	7	34	0	**26.56%**	128
Burkholderia cepacia complex	158	9	15	0	**8.24%**	182
Burkholderia gladioli	3	0	2	0	**40%**	5
Burkholderia sp.	0	1	2	0	**66.67%**	3
Cedecea lapagei	0	0	1	0	**100%**	1
Chromobacterium violaceum	1	0	0	0	**0%**	1
Chryseobacterium indologenes	0	0	2	0	**100%**	2

Análise de Resistência Bacteriana - Teste de Sensibilidade

Antibiótico/Microrganismo	Sensível	Intermediário	Resistente	Não Testado	% Resistentes	Total
Citrobacter braakii	3	0	1	0	**25%**	4
Citrobacter farmeri	2	0	0	0	**0%**	2
Citrobacter freundii	35	0	6	0	**14.63%**	41
Citrobacter koseri	32	0	4	0	**11.11%**	36
Citrobacter sp.	5	0	1	0	**16.67%**	6
Edwardsiella tarda	2	0	0	0	**0%**	2
Elizabethkingia meningoseptica (Chryseobacterium meningosepticum)	0	0	3	0	**100%**	3
Enterobacter aerogenes	47	0	0	0	**0%**	47
Enterobacter asburiae	3	0	1	0	**25%**	4
Enterobacter cancerogenus	1	1	0	0	**0%**	2
Enterobacter cloacae	265	9	56	0	**16.97%**	330
Enterobacter sakazakii	2	0	0	0	**0%**	2
Enterobacter sp.	5	0	3	0	**37.5%**	8
Enterococcus faecalis	3	0	0	0	**0%**	3
Enterococcus faecium	1	0	0	0	**0%**	1
Enterococcus sp.	2	0	0	0	**0%**	2
Escherichia coli	1981	14	150	0	**6.99%**	2145
Escherichia coli enteroinvasora	1	0	0	0	**0%**	1
Escherichia coli enteropatogênica	1	0	0	0	**0%**	1
Escherichia coli enteropatogênica clássica B O142	1	0	0	0	**0%**	1
Escherichia fergusonii	1	0	0	0	**0%**	1
Escherichia sp.	1	0	0	0	**0%**	1
Hafnia alvei	0	1	0	0	**0%**	1
Kingella denitrificans	0	0	1	0	**100%**	1
Klebsiella aerogenes	48	1	11	0	**18.33%**	60
Klebsiella oxytoca	28	1	13	0	**30.95%**	42
Klebsiella ozaenae	14	0	8	0	**36.36%**	22
Klebsiella planticola	1	0	1	0	**50%**	2
Klebsiella pneumoniae	1286	28	790	0	**37.55%**	2104
Klebsiella pneumoniae subsp. pneumoniae	57	0	15	0	**20.83%**	72
Klebsiella sp.	72	4	33	0	**30.28%**	109
Kluyvera ascorbata	5	0	1	0	**16.67%**	6
Kluyvera sp.	1	0	0	0	**0%**	1
Leclercia adecarboxylata	1	0	0	0	**0%**	1
Morganella morganii	90	1	3	0	**3.19%**	94
Morganella morganii subsp. morganii	7	0	0	0	**0%**	7
Morganella morganii subsp. sibonii	1	0	0	0	**0%**	1
Morganella sp.	1	0	0	0	**0%**	1
Neisseria meningitidis	2	0	0	0	**0%**	2
Ochrobactrum anthropi	1	0	0	0	**0%**	1
Pantoea agglomerans	9	0	6	0	**40%**	15
Pantoea sp.	2	0	1	0	**33.33%**	3
Pluralibacter gergoviae	1	0	1	0	**50%**	2
Proteus mirabilis	222	6	25	0	**9.88%**	253
Proteus penneri	3	0	0	0	**0%**	3
Proteus sp.	5	0	0	0	**0%**	5
Proteus vulgaris	14	0	1	0	**6.67%**	15
Providencia alcalifaciens	0	0	1	0	**100%**	1
Providencia rettgeri	6	0	3	0	**33.33%**	9

Análise de Resistência Bacteriana - Teste de Sensibilidade

Antibiótico/Microrganismo	Sensível	Intermediário	Resistente	Não Testado	% Resistentes	Total
Providencia rustigianii	1	1	0	0	**0%**	2
Providencia sp.	1	0	0	0	**0%**	1
Providencia stuartii	57	1	18	0	**23.68%**	76
Pseudomonas aeruginosa	683	63	319	0	**29.95%**	1065
Pseudomonas fluorescens	4	0	0	0	**0%**	4
Pseudomonas luteola	4	0	0	0	**0%**	4
Pseudomonas mendocina	2	0	0	0	**0%**	2
Pseudomonas oryzihabitans	1	0	0	0	**0%**	1
Pseudomonas pseudoalcaligenes	0	0	1	0	**100%**	1
Pseudomonas putida	15	4	4	0	**17.39%**	23
Pseudomonas sp.	15	1	6	0	**27.27%**	22
Pseudomonas stutzeri	2	0	0	0	**0%**	2
Ralstonia pickettii	2	0	0	0	**0%**	2
Raoultella ornithinolytica	1	0	0	0	**0%**	1
Salmonella enterica	4	0	1	0	**20%**	5
Salmonella enterica subsp. enterica	1	0	0	0	**0%**	1
Salmonella Enteritidis	3	2	2	0	**28.57%**	7
Salmonella sp.	11	0	0	0	**0%**	11
Serratia ficaria	0	2	0	0	**0%**	2
Serratia liquefaciens	3	0	1	0	**25%**	4
Serratia marcescens	217	6	80	0	**26.4%**	303
Serratia plymuthica	8	0	1	0	**11.11%**	9
Serratia rubidaea	3	0	1	0	**25%**	4
Serratia sp.	15	0	4	0	**21.05%**	19
Shigella boydii	2	0	0	0	**0%**	2
Shigella flexneri	2	0	0	0	**0%**	2
Shigella sonnei	1	0	0	0	**0%**	1
Sphingobacterium spiritivorum	0	0	1	0	**100%**	1
Sphingomonas paucimobilis	17	0	2	0	**10.53%**	19
Staphylococcus aureus	2	0	1	0	**33.33%**	3
Stenotrophomonas maltophilia	1	1	2	0	**50%**	4
Streptococcus agalactiae	1	0	0	0	**0%**	1
Streptococcus mitis	1	0	0	0	**0%**	1
Streptococcus pneumoniae	2	0	0	0	**0%**	2
Streptococcus pyogenes (beta hemolítico grupo A)	1	0	0	0	**0%**	1
Streptococcus pyogenes (grupo A - Lancefield)	1	0	0	0	**0%**	1
Yersinia intermedia	0	0	1	0	**100%**	1
**Subtotal**	**6091**	**180**	**2855**	**0**	**31.28%**	**9126**
Total Geral	***15065***	***630***	***7251***	***0***	***31.6%***	***22946***

<u>Observação:</u>

% Resistentes - *O percentual de resistentes é calculado levando em consideração o valor da coluna "Resistentes" em relação ao valor da coluna "Total".*
