# Supplementary material for: Biodiversity of carbapenem-resistant bacteria in clinical samples from the Southwest Amazon region (Rondônia/Brazil)
Source: Sci Rep. 2024 Apr 23;14:9383. doi: 10.1038/s41598-024-59733-w (PMC11039742; doi:10.1038/s41598-024-59733-w)
Supplement: Supplementary file 5 — Supplementary Information 5. [file 41598_2024_59733_MOESM5_ESM.pdf]

Análise de Resistência Bacteriana - Teste de Sensibilidade

Exame: Bactérias, Teste de Sensibilidade
Bactérias, Teste de Sensibilidade II
Cólera, Teste de Sensibilidade
Febre Tifóide, TSA
Meningite Bacteriana, TSA

Metodologia: Teste de Sensibilidade

Data Início: 01/01/2018

Data Fim: 31/12/2021

Total Exame/Metodologia: 15025

Microrganismo/Antibiótico	Sensível	Intermediário	Resistente	Não Testado	% Resistentes	Total
**Achromobacter denitrificans**						
Amicacina	1	0	0	0	**0%**	1
Ceftriaxona	1	0	0	0	**0%**	1
Ciprofloxacina	0	1	0	0	**0%**	1
Gentamicina	1	0	0	0	**0%**	1
Meropenem	1	0	0	0	**0%**	1
Norfloxacina	0	1	0	0	**0%**	1
Piperacilina/Tazobactam	1	0	0	0	**0%**	1
**Subtotal**	**5**	**2**	**0**	**0**	**0%**	**7**
**Achromobacter sp.**						
Cefepime	0	2	3	0	**60%**	5
Ceftazidima	3	2	0	0	**0%**	5
Ciprofloxacina	0	0	5	0	**100%**	5
Gentamicina	0	0	4	0	**100%**	4
Imipenem	4	1	0	0	**0%**	5
Levofloxacina	1	4	0	0	**0%**	5
Piperacilina/Tazobactam	5	0	0	0	**0%**	5
Sulfametoxazol/trimetoprim	1	0	0	0	**0%**	1
Trimetoprim/Sulfametoxazol	4	0	0	0	**0%**	4
**Subtotal**	**18**	**9**	**12**	**0**	**30.77%**	**39**
**Achromobacter xylosoxidans**						
Amicacina	3	1	4	0	**50%**	8
Ampicilina	0	1	3	0	**75%**	4
Ampicilina/Sulbactam	0	3	1	0	**25%**	4
Cefepime	2	3	3	0	**37.5%**	8
Cefoxitina	0	0	4	0	**100%**	4
Ceftazidima	8	0	0	0	**0%**	8
Ceftriaxona	0	1	7	0	**87.5%**	8
Cefuroxima	0	0	4	0	**100%**	4
Cefuroxima axetil	0	0	3	0	**100%**	3
Ciprofloxacina	0	4	4	0	**50%**	8
Gentamicina	1	4	3	0	**37.5%**	8
Imipenem	7	1	0	0	**0%**	8
Meropenem	8	0	0	0	**0%**	8
Piperacilina	1	0	0	0	**0%**	1
Piperacilina/Tazobactam	6	0	1	0	**14.29%**	7
Tigeciclina	8	0	0	0	**0%**	8
**Subtotal**	**44**	**18**	**37**	**0**	**37.37%**	**99**
**Acinetobacter anitratus**						
Amicacina	0	0	1	0	**100%**	1
Ampicilina/Sulbactam	0	0	1	0	**100%**	1
Cefepime	0	0	1	0	**100%**	1
Ceftazidima	0	0	1	0	**100%**	1
Ciprofloxacina	0	0	1	0	**100%**	1

Análise de Resistência Bacteriana - Teste de Sensibilidade

Microrganismo/Antibiótico	Sensível	Intermediário	Resistente	Não Testado	% Resistentes	Total
Gentamicina	0	0	1	0	**100%**	1
Imipenem	0	0	1	0	**100%**	1
Levofloxacina	0	0	1	0	**100%**	1
Meropenem	0	0	1	0	**100%**	1
Piperacilina/Tazobactam	0	0	1	0	**100%**	1
Trimetoprim/Sulfametoxazol	0	0	1	0	**100%**	1
**Subtotal**	**0**	**0**	**11**	**0**	**100%**	**11**
**Acinetobacter baumannii**						
Acido Nalidíxico	8	0	18	0	**69.23%**	26
Amicacina	569	85	864	0	**56.92%**	1518
Amoxacilina/Ácido Clavulânico	2	0	48	0	**96%**	50
Amoxicilina	0	0	2	0	**100%**	2
Ampicilina	4	0	374	0	**98.94%**	378
Ampicilina/Sulbactam	336	181	671	0	**56.48%**	1188
Azitromicina	0	0	1	0	**100%**	1
Aztreonam	0	0	10	0	**100%**	10
Carbenicilina	0	0	0	0	**0%**	0
Cefalotina	1	0	4	0	**80%**	5
Cefazolina	0	0	2	0	**100%**	2
Cefepime	382	47	1095	0	**71.85%**	1524
Cefotaxima	0	0	24	0	**100%**	24
Cefoxitina	3	1	372	0	**98.94%**	376
Ceftaroline	0	0	1	0	**100%**	1
Ceftazidima	368	53	1039	0	**71.16%**	1460
Ceftriaxona	50	114	378	0	**69.74%**	542
Cefuroxima	3	0	360	0	**99.17%**	363
Cefuroxima axetil	1	0	333	0	**99.7%**	334
Ciprofloxacina	411	20	1084	0	**71.55%**	1515
Cloranfenicol	1	0	7	0	**87.5%**	8
Colistin	64	0	6	0	**8.57%**	70
Doxiciclina	1	0	3	0	**75%**	4
Ertapenem	5	0	61	0	**92.42%**	66
Gentamicina	405	118	956	0	**64.64%**	1479
Gentamicina 120 mg	1	1	2	0	**50%**	4
Imipenem	430	18	1123	0	**71.48%**	1571
Levofloxacina	247	2	763	0	**75.4%**	1012
Lomefloxacina	0	0	3	0	**100%**	3
Meropenem	440	12	1129	0	**71.41%**	1581
Minociclina	0	0	0	0	**0%**	0
Nitrofurantoína	0	0	29	0	**100%**	29
Norfloxacina	9	1	18	0	**64.29%**	28
Ofloxacina	1	0	2	0	**66.67%**	3
Penicilina	0	0	1	0	**100%**	1
Piperacilina	0	0	3	0	**100%**	3
Piperacilina/Tazobactam	381	31	1065	0	**72.11%**	1477
Polimixina "B"	33	0	1	0	**2.94%**	34
Sulfametoxazol/trimetoprim	23	0	81	0	**77.88%**	104
Sulfazotrim	0	0	4	0	**100%**	4
Tetraciclina	36	6	52	0	**55.32%**	94
Tigeciclina	265	60	55	0	**14.47%**	380
Tobramicina	1	0	2	0	**66.67%**	3
Trimetoprim/Sulfametoxazol	300	0	711	0	**70.33%**	1011

Análise de Resistência Bacteriana - Teste de Sensibilidade

Microrganismo/Antibiótico	Sensível	Intermediário	Resistente	Não Testado	% Resistentes	Total
**Subtotal**	**4781**	**750**	**12757**	**0**	**69.76%**	**18288**
**Acinetobacter haemolyticus**						
Amicacina	3	0	0	0	**0%**	3
Ampicilina	0	0	1	0	**100%**	1
Ampicilina/Sulbactam	2	0	1	0	**33.33%**	3
Cefepime	2	0	1	0	**33.33%**	3
Cefoxitina	0	0	1	0	**100%**	1
Ceftazidima	3	0	0	0	**0%**	3
Ceftriaxona	3	0	0	0	**0%**	3
Cefuroxima	0	0	1	0	**100%**	1
Cefuroxima axetil	0	0	1	0	**100%**	1
Ciprofloxacina	2	0	1	0	**33.33%**	3
Ertapenem	0	0	1	0	**100%**	1
Gentamicina	3	0	0	0	**0%**	3
Imipenem	2	0	0	0	**0%**	2
Levofloxacina	1	1	0	0	**0%**	2
Meropenem	3	0	0	0	**0%**	3
Piperacilina/Tazobactam	2	0	1	0	**33.33%**	3
Tigeciclina	1	0	0	0	**0%**	1
Trimetoprim/Sulfametoxazol	1	0	1	0	**50%**	2
**Subtotal**	**28**	**1**	**10**	**0**	**25.64%**	**39**
**Acinetobacter lwoffii**						
Amicacina	3	0	3	0	**50%**	6
Ampicilina	2	0	2	0	**50%**	4
Ampicilina/Sulbactam	2	2	1	0	**20%**	5
Cefepime	2	0	4	0	**66.67%**	6
Cefoxitina	2	0	2	0	**50%**	4
Ceftazidima	1	1	4	0	**66.67%**	6
Ceftriaxona	2	0	2	0	**50%**	4
Cefuroxima	1	0	3	0	**75%**	4
Cefuroxima axetil	1	0	3	0	**75%**	4
Ciprofloxacina	4	0	2	0	**33.33%**	6
Colistin	0	0	1	0	**100%**	1
Gentamicina	3	0	3	0	**50%**	6
Imipenem	3	0	3	0	**50%**	6
Levofloxacina	0	0	2	0	**100%**	2
Meropenem	2	1	3	0	**50%**	6
Piperacilina/Tazobactam	2	0	3	0	**60%**	5
Tetraciclina	0	1	0	0	**0%**	1
Tigeciclina	4	0	0	0	**0%**	4
Trimetoprim/Sulfametoxazol	0	0	2	0	**100%**	2
**Subtotal**	**34**	**5**	**43**	**0**	**52.44%**	**82**
**Acinetobacter sp.**						
Amicacina	30	20	34	0	**40.48%**	84
Amoxacilina/Ácido Clavulânico	2	0	3	0	**60%**	5
Ampicilina	0	0	26	0	**100%**	26
Ampicilina/Sulbactam	21	28	18	0	**26.87%**	67
Azitromicina	0	0	3	0	**100%**	3
Aztreonam	1	0	2	0	**66.67%**	3
Cefalotina	0	0	3	0	**100%**	3
Cefazolina	0	0	4	0	**100%**	4
Cefepime	23	1	74	0	**75.51%**	98

Análise de Resistência Bacteriana - Teste de Sensibilidade

Microrganismo/Antibiótico	Sensível	Intermediário	Resistente	Não Testado	% Resistentes	Total
Cefotaxima	1	0	5	0	**83.33%**	6
Cefoxitina	4	0	29	0	**87.88%**	33
Ceftazidima	19	3	68	0	**75.56%**	90
Ceftriaxona	12	2	37	0	**72.55%**	51
Cefuroxima	1	0	13	0	**92.86%**	14
Cefuroxima axetil	1	0	10	0	**90.91%**	11
Ciprofloxacina	26	1	65	0	**70.65%**	92
Clofazilina	1	0	1	0	**50%**	2
Cloranfenicol	0	0	10	0	**100%**	10
Colistin	0	0	1	0	**100%**	1
Doxiciclina	8	0	3	0	**27.27%**	11
Ertapenem	3	0	18	0	**85.71%**	21
Gentamicina	22	2	61	0	**71.76%**	85
Gentamicina 120 mg	1	0	0	0	**0%**	1
Imipenem	26	2	83	0	**74.77%**	111
Levofloxacina	16	0	50	0	**75.76%**	66
Meropenem	28	3	78	0	**71.56%**	109
Nitrofurantoína	1	0	0	0	**0%**	1
Norfloxacina	1	0	0	0	**0%**	1
Ofloxacina	1	0	6	0	**85.71%**	7
Piperacilina	0	0	2	0	**100%**	2
Piperacilina/Tazobactam	22	5	63	0	**70%**	90
Polimixina "B"	1	0	1	0	**50%**	2
Sulfametoxazol/trimetoprim	5	0	22	0	**81.48%**	27
Tetraciclina	3	0	21	0	**87.5%**	24
Tigeciclina	9	2	3	0	**21.43%**	14
Tobramicina	6	1	7	0	**50%**	14
Trimetoprim/Sulfametoxazol	42	1	10	0	**18.87%**	53
**Subtotal**	**337**	**71**	**834**	**0**	**67.15%**	**1242**
**Aeromonas caviae**						
Amicacina	1	0	0	0	**0%**	1
Ampicilina/Sulbactam	0	0	1	0	**100%**	1
Cefepime	3	0	0	0	**0%**	3
Cefoxitina	0	0	1	0	**100%**	1
Ceftazidima	3	0	0	0	**0%**	3
Ceftriaxona	0	2	0	0	**0%**	2
Cefuroxima	1	0	0	0	**0%**	1
Ciprofloxacina	2	0	1	0	**33.33%**	3
Gentamicina	1	0	0	0	**0%**	1
Imipenem	1	0	0	0	**0%**	1
Levofloxacina	2	0	0	0	**0%**	2
Piperacilina/Tazobactam	1	0	0	0	**0%**	1
Tigeciclina	1	0	0	0	**0%**	1
Trimetoprim/Sulfametoxazol	2	0	0	0	**0%**	2
**Subtotal**	**18**	**2**	**3**	**0**	**13.04%**	**23**
**Aeromonas hydrophila**						
Amicacina	1	0	0	0	**0%**	1
Ampicilina	0	0	1	0	**100%**	1
Ampicilina/Sulbactam	0	0	1	0	**100%**	1
Cefepime	2	3	1	0	**16.67%**	6
Cefoxitina	0	1	0	0	**0%**	1
Ceftazidima	5	0	1	0	**16.67%**	6

Análise de Resistência Bacteriana - Teste de Sensibilidade

Microrganismo/Antibiótico	Sensível	Intermediário	Resistente	Não Testado	% Resistentes	Total
Ceftriaxona	4	0	2	0	**33.33%**	6
Cefuroxima	1	0	0	0	**0%**	1
Cefuroxima axetil	1	0	0	0	**0%**	1
Ciprofloxacina	5	1	0	0	**0%**	6
Gentamicina	1	0	0	0	**0%**	1
Imipenem	0	1	0	0	**0%**	1
Levofloxacina	4	0	1	0	**20%**	5
Meropenem	0	0	1	0	**100%**	1
Piperacilina/Tazobactam	1	0	0	0	**0%**	1
Tigeciclina	1	0	0	0	**0%**	1
Trimetoprim/Sulfametoxazol	4	0	1	0	**20%**	5
**Subtotal**	**30**	**6**	**9**	**0**	**20%**	**45**
**Aeromonas hydrophila/Aeromonas caviae**						
Amicacina	6	0	1	0	**14.29%**	7
Amoxacilina/Ácido Clavulânico	0	1	0	0	**0%**	1
Ampicilina	0	0	7	0	**100%**	7
Ampicilina/Sulbactam	0	1	5	0	**83.33%**	6
Cefepime	7	0	0	0	**0%**	7
Cefoxitina	2	0	3	0	**60%**	5
Ceftazidima	5	0	0	0	**0%**	5
Ceftriaxona	3	0	4	0	**57.14%**	7
Cefuroxima	3	0	4	0	**57.14%**	7
Cefuroxima axetil	3	0	4	0	**57.14%**	7
Ciprofloxacina	7	0	0	0	**0%**	7
Gentamicina	3	1	3	0	**42.86%**	7
Imipenem	5	0	0	0	**0%**	5
Meropenem	5	0	2	0	**28.57%**	7
Nitrofurantoína	2	0	0	0	**0%**	2
Norfloxacina	2	0	0	0	**0%**	2
Piperacilina/Tazobactam	6	0	1	0	**14.29%**	7
Tigeciclina	5	0	0	0	**0%**	5
Trimetoprim/Sulfametoxazol	1	0	1	0	**50%**	2
**Subtotal**	**65**	**3**	**35**	**0**	**33.98%**	**103**
**Aeromonas sp.**						
Amicacina	1	0	0	0	**0%**	1
Ampicilina/Sulbactam	0	0	1	0	**100%**	1
Cefepime	2	0	0	0	**0%**	2
Cefoxitina	0	0	1	0	**100%**	1
Ceftazidima	2	0	0	0	**0%**	2
Ceftriaxona	1	0	0	0	**0%**	1
Cefuroxima	1	0	0	0	**0%**	1
Ciprofloxacina	2	0	0	0	**0%**	2
Gentamicina	1	0	0	0	**0%**	1
Imipenem	0	1	0	0	**0%**	1
Levofloxacina	1	0	0	0	**0%**	1
Piperacilina/Tazobactam	1	0	0	0	**0%**	1
Tigeciclina	1	0	0	0	**0%**	1
Trimetoprim/Sulfametoxazol	1	0	0	0	**0%**	1
**Subtotal**	**14**	**1**	**2**	**0**	**11.76%**	**17**
**Aeromonas veronii**						
Cefepime	4	1	1	0	**16.67%**	6
Ceftazidima	5	0	1	0	**16.67%**	6

Análise de Resistência Bacteriana - Teste de Sensibilidade

Microrganismo/Antibiótico	Sensível	Intermediário	Resistente	Não Testado	% Resistentes	Total
Ceftriaxona	5	0	1	0	**16.67%**	6
Ciprofloxacina	6	0	0	0	**0%**	6
Levofloxacina	6	0	0	0	**0%**	6
Trimetoprim/Sulfametoxazol	6	0	0	0	**0%**	6
**Subtotal**	**32**	**1**	**3**	**0**	**8.33%**	**36**
**Alcaligenes faecalis**						
Amicacina	4	0	1	0	**20%**	5
Ampicilina	0	0	1	0	**100%**	1
Cefazolina	0	0	1	0	**100%**	1
Cefepime	5	0	0	0	**0%**	5
Cefoxitina	0	0	1	0	**100%**	1
Ceftazidima	3	1	1	0	**20%**	5
Ciprofloxacina	1	0	4	0	**80%**	5
Ertapenem	0	0	1	0	**100%**	1
Gentamicina	2	1	2	0	**40%**	5
Imipenem	4	0	1	0	**20%**	5
Levofloxacina	1	0	4	0	**80%**	5
Piperacilina/Tazobactam	4	0	1	0	**20%**	5
Trimetoprim/Sulfametoxazol	1	0	4	0	**80%**	5
**Subtotal**	**25**	**2**	**22**	**0**	**44.9%**	**49**
**Bacillus cereus**						
Ampicilina	0	0	4	0	**100%**	4
Cefoxitina	0	0	4	0	**100%**	4
Ceftaroline	0	0	4	0	**100%**	4
Penicilina	0	0	4	0	**100%**	4
**Subtotal**	**0**	**0**	**16**	**0**	**100%**	**16**
**Bacillus megaterium**						
Penicilina	0	0	0	0	**0%**	0
**Subtotal**	**0**	**0**	**0**	**0**	**0%**	**0**
**Burkholderia cenocepacia**						
Ceftazidima	0	0	1	0	**100%**	1
Meropenem	0	0	1	0	**100%**	1
**Subtotal**	**0**	**0**	**2**	**0**	**100%**	**2**
**Burkholderia cepacia**						
Amicacina	8	3	18	0	**62.07%**	29
Amoxacilina/Ácido Clavulânico	0	0	1	0	**100%**	1
Ampicilina	1	1	18	0	**90%**	20
Ampicilina/Sulbactam	2	4	14	0	**70%**	20
Aztreonam	2	0	1	0	**33.33%**	3
Cefalotina	0	0	1	0	**100%**	1
Cefepime	14	0	18	0	**56.25%**	32
Cefoxitina	0	0	21	0	**100%**	21
Ceftazidima	87	7	30	0	**24.19%**	124
Ceftriaxona	1	4	20	0	**80%**	25
Cefuroxima	1	0	20	0	**95.24%**	21
Cefuroxima axetil	1	0	19	0	**95%**	20
Ciprofloxacina	4	0	20	0	**83.33%**	24
Cloranfenicol	1	0	0	0	**0%**	1
Colistin	2	0	2	0	**50%**	4
Ertapenem	0	0	6	0	**100%**	6
Gentamicina	7	0	21	0	**75%**	28
Imipenem	5	1	27	0	**81.82%**	33

Análise de Resistência Bacteriana - Teste de Sensibilidade

Microrganismo/Antibiótico	Sensível	Intermediário	Resistente	Não Testado	% Resistentes	Total
Levofloxacina	51	10	21	0	**25.61%**	82
Meropenem	87	7	34	0	**26.56%**	128
Minociclina	1	0	0	0	**0%**	1
Nitrofurantoína	0	0	1	0	**100%**	1
Norfloxacina	0	0	1	0	**100%**	1
Piperacilina/Tazobactam	7	0	19	0	**73.08%**	26
Sulfametoxazol/trimetoprim	18	0	3	0	**14.29%**	21
Tetraciclina	1	0	2	0	**66.67%**	3
Ticarcilina/Ácido Clavulânico	0	0	0	0	**0%**	0
Tigeciclina	13	5	6	0	**25%**	24
Trimetoprim/Sulfametoxazol	59	0	12	0	**16.9%**	71
**Subtotal**	**373**	**42**	**356**	**0**	**46.17%**	**771**
**Burkholderia cepacia complex**						
Cefepime	0	0	1	0	**100%**	1
Ceftazidima	144	17	20	0	**11.05%**	181
Ertapenem	0	0	7	0	**100%**	7
Imipenem	0	0	1	0	**100%**	1
Levofloxacina	136	23	22	0	**12.15%**	181
Meropenem	158	9	15	0	**8.24%**	182
Polimixina "B"	0	0	1	0	**100%**	1
Sulfametoxazol/trimetoprim	2	0	0	0	**0%**	2
Trimetoprim/Sulfametoxazol	166	0	14	0	**7.78%**	180
**Subtotal**	**606**	**49**	**81**	**0**	**11.01%**	**736**
**Burkholderia gladioli**						
Amicacina	5	0	0	0	**0%**	5
Ceftazidima	1	2	2	0	**40%**	5
Ciprofloxacina	5	0	0	0	**0%**	5
Gentamicina	4	1	0	0	**0%**	5
Levofloxacina	5	0	0	0	**0%**	5
Meropenem	3	0	2	0	**40%**	5
Piperacilina/Tazobactam	4	1	0	0	**0%**	5
Trimetoprim/Sulfametoxazol	3	0	2	0	**40%**	5
**Subtotal**	**30**	**4**	**6**	**0**	**15%**	**40**
**Burkholderia sp.**						
Amicacina	0	1	2	0	**66.67%**	3
Ceftazidima	0	0	3	0	**100%**	3
Ciprofloxacina	2	0	1	0	**33.33%**	3
Gentamicina	0	0	3	0	**100%**	3
Levofloxacina	2	0	1	0	**33.33%**	3
Meropenem	0	1	2	0	**66.67%**	3
Piperacilina/Tazobactam	2	0	1	0	**33.33%**	3
Sulfametoxazol/trimetoprim	1	0	0	0	**0%**	1
Trimetoprim/Sulfametoxazol	1	0	1	0	**50%**	2
**Subtotal**	**8**	**2**	**14**	**0**	**58.33%**	**24**
**Cedecea lapagei**						
Amicacina	0	0	1	0	**100%**	1
Amoxacilina/Ácido Clavulânico	0	0	1	0	**100%**	1
Ampicilina	0	0	1	0	**100%**	1
Cefazolina	0	0	1	0	**100%**	1
Cefepime	0	0	1	0	**100%**	1
Cefoxitina	0	0	1	0	**100%**	1
Ceftriaxona	0	0	1	0	**100%**	1

Análise de Resistência Bacteriana - Teste de Sensibilidade

Microrganismo/Antibiótico	Sensível	Intermediário	Resistente	Não Testado	% Resistentes	Total
Cefuroxima	0	0	1	0	**100%**	1
Ciprofloxacina	0	0	1	0	**100%**	1
Ertapenem	0	0	1	0	**100%**	1
Gentamicina	0	0	1	0	**100%**	1
Imipenem	0	0	1	0	**100%**	1
Levofloxacina	0	0	1	0	**100%**	1
Meropenem	0	0	1	0	**100%**	1
Piperacilina/Tazobactam	0	1	0	0	**0%**	1
Polimixina "B"	1	0	0	0	**0%**	1
Trimetoprim/Sulfametoxazol	0	0	1	0	**100%**	1
**Subtotal**	**1**	**1**	**15**	**0**	**88.24%**	**17**
**Chromobacterium violaceum**						
Acido Nalidíxico	1	0	0	0	**0%**	1
Amicacina	1	0	0	0	**0%**	1
Amoxacilina/Ácido Clavulânico	0	0	1	0	**100%**	1
Ampicilina	0	0	2	0	**100%**	2
Aztreonam	1	0	0	0	**0%**	1
Cefoxitina	0	0	1	0	**100%**	1
Cefuroxima	0	0	1	0	**100%**	1
Ciprofloxacina	1	0	0	0	**0%**	1
Gentamicina	2	0	0	0	**0%**	2
Meropenem	1	0	0	0	**0%**	1
Nitrofurantoína	1	0	0	0	**0%**	1
Polimixina "B"	0	0	1	0	**100%**	1
Sulfametoxazol/trimetoprim	1	0	0	0	**0%**	1
Sulfazotrim	1	0	0	0	**0%**	1
Tetraciclina	1	0	0	0	**0%**	1
**Subtotal**	**11**	**0**	**6**	**0**	**35.29%**	**17**
**Chryseobacterium gleum**						
Cefepime	0	0	1	0	**100%**	1
Ceftazidima	0	0	1	0	**100%**	1
Ciprofloxacina	1	0	0	0	**0%**	1
Gentamicina	0	0	1	0	**100%**	1
Levofloxacina	1	0	0	0	**0%**	1
Piperacilina/Tazobactam	1	0	0	0	**0%**	1
Trimetoprim/Sulfametoxazol	1	0	0	0	**0%**	1
**Subtotal**	**4**	**0**	**3**	**0**	**42.86%**	**7**
**Chryseobacterium indologenes**						
Amicacina	0	0	2	0	**100%**	2
Ampicilina	0	0	2	0	**100%**	2
Ampicilina/Sulbactam	0	0	2	0	**100%**	2
Cefepime	0	0	2	0	**100%**	2
Cefoxitina	0	0	2	0	**100%**	2
Ceftazidima	0	0	2	0	**100%**	2
Ceftriaxona	0	0	2	0	**100%**	2
Cefuroxima	0	0	2	0	**100%**	2
Cefuroxima axetil	0	0	2	0	**100%**	2
Ciprofloxacina	0	2	0	0	**0%**	2
Gentamicina	0	0	2	0	**100%**	2
Imipenem	0	0	2	0	**100%**	2
Meropenem	0	0	2	0	**100%**	2
Piperacilina/Tazobactam	0	0	2	0	**100%**	2

Análise de Resistência Bacteriana - Teste de Sensibilidade

Microrganismo/Antibiótico	Sensível	Intermediário	Resistente	Não Testado	% Resistentes	Total
Tigeciclina	2	0	0	0	**0%**	2
**Subtotal**	**2**	**2**	**26**	**0**	**86.67%**	**30**
**Citrobacter braakii**						
Amicacina	3	1	0	0	**0%**	4
Amoxacilina/Ácido Clavulânico	0	0	2	0	**100%**	2
Ampicilina	0	0	3	0	**100%**	3
Ampicilina/Sulbactam	0	0	2	0	**100%**	2
Cefazolina	1	0	1	0	**50%**	2
Cefepime	1	0	3	0	**75%**	4
Cefoxitina	0	0	4	0	**100%**	4
Ceftaroline	0	0	1	0	**100%**	1
Ceftriaxona	1	0	3	0	**75%**	4
Cefuroxima	2	0	0	0	**0%**	2
Ciprofloxacina	3	0	1	0	**25%**	4
Ertapenem	2	0	2	0	**50%**	4
Gentamicina	2	1	1	0	**25%**	4
Imipenem	3	0	1	0	**25%**	4
Levofloxacina	2	0	1	0	**33.33%**	3
Meropenem	3	0	1	0	**25%**	4
Nitrofurantoína	1	0	0	0	**0%**	1
Piperacilina/Tazobactam	2	0	2	0	**50%**	4
Tigeciclina	1	1	0	0	**0%**	2
Trimetoprim/Sulfametoxazol	2	0	2	0	**50%**	4
**Subtotal**	**29**	**3**	**30**	**0**	**48.39%**	**62**
**Citrobacter farmeri**						
Amicacina	2	0	0	0	**0%**	2
Amoxacilina/Ácido Clavulânico	1	0	0	0	**0%**	1
Ampicilina	0	0	2	0	**100%**	2
Ampicilina/Sulbactam	1	0	0	0	**0%**	1
Cefazolina	0	0	1	0	**100%**	1
Cefepime	2	0	0	0	**0%**	2
Cefoxitina	2	0	0	0	**0%**	2
Ceftazidima	1	0	0	0	**0%**	1
Ceftriaxona	2	0	0	0	**0%**	2
Cefuroxima	0	0	2	0	**100%**	2
Cefuroxima axetil	0	0	1	0	**100%**	1
Ciprofloxacina	2	0	0	0	**0%**	2
Ertapenem	2	0	0	0	**0%**	2
Gentamicina	2	0	0	0	**0%**	2
Imipenem	2	0	0	0	**0%**	2
Levofloxacina	1	0	0	0	**0%**	1
Meropenem	2	0	0	0	**0%**	2
Piperacilina/Tazobactam	2	0	0	0	**0%**	2
Tigeciclina	1	0	0	0	**0%**	1
Trimetoprim/Sulfametoxazol	1	0	0	0	**0%**	1
**Subtotal**	**26**	**0**	**6**	**0**	**18.75%**	**32**
**Citrobacter freundii**						
Acido Nalidíxico	4	0	1	0	**20%**	5
Amicacina	31	2	7	0	**17.5%**	40
Amoxacilina/Ácido Clavulânico	3	0	11	0	**78.57%**	14
Ampicilina	7	0	31	0	**81.58%**	38
Ampicilina/Sulbactam	3	0	21	0	**87.5%**	24

Análise de Resistência Bacteriana - Teste de Sensibilidade

Microrganismo/Antibiótico	Sensível	Intermediário	Resistente	Não Testado	% Resistentes	Total
Aztreonam	1	0	0	0	**0%**	1
Cefalotina	1	0	7	0	**87.5%**	8
Cefazolina	0	0	5	0	**100%**	5
Cefepime	25	4	12	0	**29.27%**	41
Cefoxitina	6	0	25	0	**80.65%**	31
Ceftazidima	9	4	7	0	**35%**	20
Ceftriaxona	18	0	22	0	**55%**	40
Cefuroxima	3	0	24	0	**88.89%**	27
Cefuroxima axetil	4	0	20	0	**83.33%**	24
Ciprofloxacina	23	5	13	0	**31.71%**	41
Colistin	0	0	1	0	**100%**	1
Ertapenem	31	1	7	0	**17.95%**	39
Gentamicina	24	3	12	0	**30.77%**	39
Imipenem	25	4	5	0	**14.71%**	34
Levofloxacina	1	0	6	0	**85.71%**	7
Meropenem	35	0	6	0	**14.63%**	41
Nitrofurantoína	11	0	1	0	**8.33%**	12
Norfloxacina	7	0	1	0	**12.5%**	8
Piperacilina/Tazobactam	22	8	7	0	**18.92%**	37
Sulfametoxazol/trimetoprim	0	0	1	0	**100%**	1
Tigeciclina	21	3	2	0	**7.69%**	26
Trimetoprim/Sulfametoxazol	15	0	5	0	**25%**	20
**Subtotal**	**330**	**34**	**260**	**0**	**41.67%**	**624**
**Citrobacter koseri**						
Acido Nalidíxico	1	0	0	0	**0%**	1
Amicacina	32	0	2	0	**5.88%**	34
Amoxacilina/Ácido Clavulânico	9	0	1	0	**10%**	10
Ampicilina	0	0	32	0	**100%**	32
Ampicilina/Sulbactam	21	1	3	0	**12%**	25
Cefalotina	1	0	0	0	**0%**	1
Cefazolina	16	0	2	0	**11.11%**	18
Cefepime	31	1	3	0	**8.57%**	35
Cefoxitina	31	1	1	0	**3.03%**	33
Ceftazidima	12	0	2	0	**14.29%**	14
Ceftriaxona	30	0	4	0	**11.76%**	34
Cefuroxima	17	2	3	0	**13.64%**	22
Cefuroxima axetil	4	7	3	0	**21.43%**	14
Ciprofloxacina	31	0	5	0	**13.89%**	36
Colistin	5	0	0	0	**0%**	5
Ertapenem	29	0	6	0	**17.14%**	35
Gentamicina	33	0	1	0	**2.94%**	34
Imipenem	31	2	2	0	**5.71%**	35
Levofloxacina	13	0	2	0	**13.33%**	15
Meropenem	32	0	4	0	**11.11%**	36
Nitrofurantoína	2	4	0	0	**0%**	6
Norfloxacina	1	0	0	0	**0%**	1
Piperacilina/Tazobactam	31	1	2	0	**5.88%**	34
Sulfametoxazol/trimetoprim	1	0	0	0	**0%**	1
Tigeciclina	25	0	0	0	**0%**	25
Trimetoprim/Sulfametoxazol	21	0	0	0	**0%**	21
**Subtotal**	**460**	**19**	**78**	**0**	**14%**	**557**
**Citrobacter sp.**						

Análise de Resistência Bacteriana - Teste de Sensibilidade

Microrganismo/Antibiótico	Sensível	Intermediário	Resistente	Não Testado	% Resistentes	Total
Amicacina	5	0	0	0	**0%**	5
Amoxacilina/Ácido Clavulânico	0	0	1	0	**100%**	1
Ampicilina	0	0	2	0	**100%**	2
Ampicilina/Sulbactam	4	0	1	0	**20%**	5
Cefazolina	0	0	2	0	**100%**	2
Cefepime	5	0	1	0	**16.67%**	6
Cefoxitina	0	0	5	0	**100%**	5
Ceftazidima	1	0	0	0	**0%**	1
Ceftriaxona	5	0	0	0	**0%**	5
Ciprofloxacina	5	0	2	0	**28.57%**	7
Ertapenem	4	0	1	0	**20%**	5
Gentamicina	5	0	2	0	**28.57%**	7
Imipenem	6	0	0	0	**0%**	6
Levofloxacina	4	0	0	0	**0%**	4
Meropenem	5	0	1	0	**16.67%**	6
Piperacilina/Tazobactam	4	0	0	0	**0%**	4
Sulfametoxazol/trimetoprim	1	0	1	0	**50%**	2
Tetraciclina	0	0	2	0	**100%**	2
Tigeciclina	3	1	0	0	**0%**	4
Trimetoprim/Sulfametoxazol	4	0	0	0	**0%**	4
**Subtotal**	**61**	**1**	**21**	**0**	**25.3%**	**83**
**Delftia acidovorans**						
Amicacina	0	0	2	0	**100%**	2
Cefepime	0	0	2	0	**100%**	2
Ceftazidima	1	1	0	0	**0%**	2
Ciprofloxacina	0	0	2	0	**100%**	2
Gentamicina	0	0	2	0	**100%**	2
Imipenem	0	0	2	0	**100%**	2
Levofloxacina	0	0	2	0	**100%**	2
Piperacilina/Tazobactam	1	1	0	0	**0%**	2
Trimetoprim/Sulfametoxazol	1	0	1	0	**50%**	2
**Subtotal**	**3**	**2**	**13**	**0**	**72.22%**	**18**
**Edwardsiella tarda**						
Amicacina	2	0	0	0	**0%**	2
Ampicilina	2	0	0	0	**0%**	2
Ampicilina/Sulbactam	2	0	0	0	**0%**	2
Cefazolina	1	0	0	0	**0%**	1
Cefepime	2	0	0	0	**0%**	2
Cefoxitina	2	0	0	0	**0%**	2
Ceftazidima	1	0	0	0	**0%**	1
Ceftriaxona	2	0	0	0	**0%**	2
Cefuroxima	1	0	0	0	**0%**	1
Cefuroxima axetil	1	0	0	0	**0%**	1
Ciprofloxacina	2	0	0	0	**0%**	2
Ertapenem	2	0	0	0	**0%**	2
Gentamicina	2	0	0	0	**0%**	2
Imipenem	2	0	0	0	**0%**	2
Levofloxacina	1	0	0	0	**0%**	1
Meropenem	2	0	0	0	**0%**	2
Piperacilina/Tazobactam	2	0	0	0	**0%**	2
Tigeciclina	2	0	0	0	**0%**	2
Trimetoprim/Sulfametoxazol	1	0	0	0	**0%**	1

Análise de Resistência Bacteriana - Teste de Sensibilidade

Microrganismo/Antibiótico	Sensível	Intermediário	Resistente	Não Testado	% Resistentes	Total
**Subtotal**	**32**	**0**	**0**	**0**	**0%**	**32**
**Elizabethkingia meningoseptica (Chryseobacterium meningosepticum)**						
Amicacina	1	0	5	0	**83.33%**	6
Ampicilina	0	0	3	0	**100%**	3
Ampicilina/Sulbactam	0	0	3	0	**100%**	3
Cefepime	0	0	6	0	**100%**	6
Cefoxitina	1	0	2	0	**66.67%**	3
Ceftazidima	0	0	6	0	**100%**	6
Ceftriaxona	0	0	3	0	**100%**	3
Cefuroxima	0	0	3	0	**100%**	3
Cefuroxima axetil	0	0	3	0	**100%**	3
Ciprofloxacina	5	0	1	0	**16.67%**	6
Colistin	0	0	2	0	**100%**	2
Gentamicina	1	0	5	0	**83.33%**	6
Imipenem	0	0	6	0	**100%**	6
Levofloxacina	2	0	1	0	**33.33%**	3
Meropenem	0	0	3	0	**100%**	3
Piperacilina/Tazobactam	2	0	4	0	**66.67%**	6
Tigeciclina	0	3	0	0	**0%**	3
Trimetoprim/Sulfametoxazol	2	0	1	0	**33.33%**	3
**Subtotal**	**14**	**3**	**57**	**0**	**77.03%**	**74**
**Enterobacter aerogenes**						
Acido Nalidíxico	2	0	1	0	**33.33%**	3
Amicacina	41	1	5	0	**10.64%**	47
Amoxacilina/Ácido Clavulânico	0	0	13	0	**100%**	13
Ampicilina	2	1	41	0	**93.18%**	44
Ampicilina/Sulbactam	4	0	23	0	**85.19%**	27
Cefalotina	0	0	4	0	**100%**	4
Cefazolina	0	0	16	0	**100%**	16
Cefepime	34	2	9	0	**20%**	45
Cefoxitina	0	0	42	0	**100%**	42
Ceftazidima	17	2	7	0	**26.92%**	26
Ceftriaxona	29	0	16	0	**35.56%**	45
Cefuroxima	6	0	25	0	**80.65%**	31
Cefuroxima axetil	5	0	22	0	**81.48%**	27
Ciprofloxacina	36	2	7	0	**15.56%**	45
Colistin	3	0	0	0	**0%**	3
Eritromicina	1	0	0	0	**0%**	1
Ertapenem	42	1	0	0	**0%**	43
Gentamicina	33	0	9	0	**21.43%**	42
Imipenem	36	7	0	0	**0%**	43
Levofloxacina	16	0	2	0	**11.11%**	18
Meropenem	47	0	0	0	**0%**	47
Nitrofurantoína	1	5	2	0	**25%**	8
Norfloxacina	3	0	1	0	**25%**	4
Piperacilina/Tazobactam	26	6	10	0	**23.81%**	42
Sulfametoxazol/trimetoprim	4	0	2	0	**33.33%**	6
Tigeciclina	26	6	1	0	**3.03%**	33
Trimetoprim/Sulfametoxazol	15	0	3	0	**16.67%**	18
**Subtotal**	**429**	**33**	**261**	**0**	**36.1%**	**723**
**Enterobacter asburiae**						
Amicacina	4	0	0	0	**0%**	4

Análise de Resistência Bacteriana - Teste de Sensibilidade

Microrganismo/Antibiótico	Sensível	Intermediário	Resistente	Não Testado	% Resistentes	Total
Ampicilina	1	0	3	0	**75%**	4
Ampicilina/Sulbactam	1	0	3	0	**75%**	4
Cefepime	3	0	1	0	**25%**	4
Cefoxitina	0	0	4	0	**100%**	4
Ceftazidima	3	0	0	0	**0%**	3
Ceftriaxona	3	0	1	0	**25%**	4
Cefuroxima	1	0	2	0	**66.67%**	3
Cefuroxima axetil	1	0	2	0	**66.67%**	3
Ciprofloxacina	3	0	1	0	**25%**	4
Ertapenem	3	0	1	0	**25%**	4
Gentamicina	3	0	1	0	**25%**	4
Imipenem	3	0	1	0	**25%**	4
Meropenem	3	0	1	0	**25%**	4
Piperacilina/Tazobactam	4	0	0	0	**0%**	4
Tigeciclina	4	0	0	0	**0%**	4
Trimetoprim/Sulfametoxazol	0	0	1	0	**100%**	1
**Subtotal**	**40**	**0**	**22**	**0**	**35.48%**	**62**
**Enterobacter cancerogenus**						
Amicacina	2	0	0	0	**0%**	2
Ampicilina	0	0	2	0	**100%**	2
Ampicilina/Sulbactam	0	1	0	0	**0%**	1
Cefazolina	0	0	1	0	**100%**	1
Cefepime	2	0	0	0	**0%**	2
Cefoxitina	0	0	1	0	**100%**	1
Ceftriaxona	2	0	0	0	**0%**	2
Cefuroxima	0	0	1	0	**100%**	1
Ciprofloxacina	2	0	0	0	**0%**	2
Ertapenem	1	0	1	0	**50%**	2
Gentamicina	2	0	0	0	**0%**	2
Imipenem	2	0	0	0	**0%**	2
Levofloxacina	1	0	0	0	**0%**	1
Meropenem	1	1	0	0	**0%**	2
Piperacilina/Tazobactam	2	0	0	0	**0%**	2
Tigeciclina	1	0	0	0	**0%**	1
Trimetoprim/Sulfametoxazol	2	0	0	0	**0%**	2
**Subtotal**	**20**	**2**	**6**	**0**	**21.43%**	**28**
**Enterobacter cloacae**						
Acido Nalidíxico	12	0	8	0	**40%**	20
Amicacina	297	14	21	0	**6.33%**	332
Amoxacilina/Ácido Clavulânico	0	0	99	0	**100%**	99
Ampicilina	5	3	288	0	**97.3%**	296
Ampicilina/Sulbactam	9	2	169	0	**93.89%**	180
Azitromicina	0	0	1	0	**100%**	1
Aztreonam	0	0	1	0	**100%**	1
Cefalotina	1	0	25	0	**96.15%**	26
Cefazolina	0	0	110	0	**100%**	110
Cefepime	171	32	127	0	**38.48%**	330
Cefotaxima	0	0	2	0	**100%**	2
Cefoxitina	10	0	275	0	**96.49%**	285
Ceftazidima	49	5	41	0	**43.16%**	95
Ceftriaxona	150	4	173	0	**52.91%**	327
Cefuroxima	31	8	147	0	**79.03%**	186

Análise de Resistência Bacteriana - Teste de Sensibilidade

Microrganismo/Antibiótico	Sensível	Intermediário	Resistente	Não Testado	% Resistentes	Total
Cefuroxima axetil	16	1	89	0	**83.96%**	106
Ciprofloxacina	205	29	100	0	**29.94%**	334
Clindamicina	0	0	1	0	**100%**	1
Cloranfenicol	0	0	2	0	**100%**	2
Colistin	17	0	5	0	**22.73%**	22
Daptomicina	1	0	0	0	**0%**	1
Eritromicina	0	0	1	0	**100%**	1
Ertapenem	221	18	86	0	**26.46%**	325
Gentamicina	211	17	102	0	**30.91%**	330
Imipenem	233	17	60	0	**19.35%**	310
Levofloxacina	127	2	52	0	**28.73%**	181
Linezolida	2	0	0	0	**0%**	2
Meropenem	265	9	56	0	**16.97%**	330
Moxifloxacina	0	0	1	0	**100%**	1
Nitrofurantoína	20	18	11	0	**22.45%**	49
Norfloxacina	20	2	5	0	**18.52%**	27
Penicilina	1	0	3	0	**75%**	4
Piperacilina	1	0	0	0	**0%**	1
Piperacilina/Tazobactam	179	39	91	0	**29.45%**	309
Polimixina "B"	2	0	0	0	**0%**	2
Sulfametoxazol/trimetoprim	6	0	5	0	**45.45%**	11
Teicoplanina	1	0	0	0	**0%**	1
Tetraciclina	0	0	3	0	**100%**	3
Ticarcilina	2	0	0	0	**0%**	2
Tigeciclina	162	42	19	0	**8.52%**	223
Trimetoprim/Sulfametoxazol	168	0	67	0	**28.51%**	235
Vancomicina	0	0	1	0	**100%**	1
**Subtotal**	**2595**	**262**	**2247**	**0**	**44.02%**	**5104**
**Enterobacter sakazakii**						
Amicacina	1	0	1	0	**50%**	2
Amoxacilina/Ácido Clavulânico	1	0	1	0	**50%**	2
Ampicilina	1	0	1	0	**50%**	2
Ampicilina/Sulbactam	0	0	1	0	**100%**	1
Aztreonam	1	0	0	0	**0%**	1
Cefepime	2	0	0	0	**0%**	2
Cefoxitina	0	0	1	0	**100%**	1
Ceftazidima	1	0	1	0	**50%**	2
Ciprofloxacina	0	0	2	0	**100%**	2
Ertapenem	1	0	1	0	**50%**	2
Gentamicina	1	0	0	0	**0%**	1
Imipenem	2	0	0	0	**0%**	2
Meropenem	2	0	0	0	**0%**	2
Sulfametoxazol/trimetoprim	1	0	1	0	**50%**	2
Tetraciclina	1	0	0	0	**0%**	1
**Subtotal**	**15**	**0**	**10**	**0**	**40%**	**25**
**Enterobacter sp.**						
Acido Nalidíxico	1	0	0	0	**0%**	1
Amicacina	3	0	2	0	**40%**	5
Amoxacilina/Ácido Clavulânico	0	0	2	0	**100%**	2
Ampicilina	1	0	5	0	**83.33%**	6
Ampicilina/Sulbactam	0	0	1	0	**100%**	1
Aztreonam	2	0	0	0	**0%**	2

Análise de Resistência Bacteriana - Teste de Sensibilidade

Microrganismo/Antibiótico	Sensível	Intermediário	Resistente	Não Testado	% Resistentes	Total
Cefepime	3	0	3	0	**50%**	6
Cefoxitina	2	0	4	0	**66.67%**	6
Ceftazidima	3	0	4	0	**57.14%**	7
Ceftriaxona	0	0	3	0	**100%**	3
Cefuroxima	1	0	2	0	**66.67%**	3
Cefuroxima axetil	0	0	1	0	**100%**	1
Ciprofloxacina	0	0	3	0	**100%**	3
Cloranfenicol	1	0	1	0	**50%**	2
Ertapenem	0	0	2	0	**100%**	2
Gentamicina	1	0	1	0	**50%**	2
Imipenem	6	0	3	0	**33.33%**	9
Levofloxacina	4	0	1	0	**20%**	5
Meropenem	5	0	3	0	**37.5%**	8
Piperacilina/Tazobactam	0	0	1	0	**100%**	1
Sulfametoxazol/trimetoprim	2	0	2	0	**50%**	4
Sulfazotrim	0	0	1	0	**100%**	1
Tetraciclina	2	0	2	0	**50%**	4
Tigeciclina	0	1	0	0	**0%**	1
**Subtotal**	**37**	**1**	**47**	**0**	**55.29%**	**85**
**Enterococcus avium**						
Ampicilina	2	0	0	0	**0%**	2
Clindamicina	0	0	1	0	**100%**	1
Daptomicina	1	0	0	0	**0%**	1
Eritromicina	1	0	0	0	**0%**	1
Estreptomicina 300 mcg	0	0	1	0	**100%**	1
Gentamicina 120 mg	1	0	0	0	**0%**	1
Levofloxacina	1	0	0	0	**0%**	1
Linezolida	2	0	0	0	**0%**	2
Nitrofurantoína	1	0	0	0	**0%**	1
Penicilina	1	0	0	0	**0%**	1
Teicoplanina	1	0	0	0	**0%**	1
Tigeciclina	1	0	0	0	**0%**	1
Trimetoprim/Sulfametoxazol	1	0	0	0	**0%**	1
Vancomicina	1	0	1	0	**50%**	2
**Subtotal**	**14**	**0**	**3**	**0**	**17.65%**	**17**
**Enterococcus casseliflavus**						
Ampicilina	10	0	3	0	**23.08%**	13
Ciprofloxacina	1	0	1	0	**50%**	2
Clindamicina	0	0	2	0	**100%**	2
Daptomicina	8	0	0	0	**0%**	8
Eritromicina	0	0	2	0	**100%**	2
Linezolida	9	1	3	0	**23.08%**	13
Moxifloxacina	1	0	1	0	**50%**	2
Nitrofurantoína	1	0	0	0	**0%**	1
Norfloxacina	0	1	1	0	**50%**	2
Penicilina	8	0	4	0	**33.33%**	12
Teicoplanina	2	0	0	0	**0%**	2
Tigeciclina	2	0	0	0	**0%**	2
Vancomicina	1	0	3	0	**75%**	4
**Subtotal**	**43**	**2**	**20**	**0**	**30.77%**	**65**
**Enterococcus durans**						
Ampicilina	2	0	0	0	**0%**	2

Análise de Resistência Bacteriana - Teste de Sensibilidade

Microrganismo/Antibiótico	Sensível	Intermediário	Resistente	Não Testado	% Resistentes	Total
Clindamicina	1	0	1	0	**50%**	2
Eritromicina	1	0	1	0	**50%**	2
Estreptomicina	1	0	0	0	**0%**	1
Gentamicina	1	0	0	0	**0%**	1
Levofloxacina	2	0	0	0	**0%**	2
Linezolida	2	0	0	0	**0%**	2
Nitrofurantoína	0	1	1	0	**50%**	2
Teicoplanina	2	0	0	0	**0%**	2
Tigeciclina	2	0	0	0	**0%**	2
Trimetoprim/Sulfametoxazol	2	0	0	0	**0%**	2
Vancomicina	2	0	0	0	**0%**	2
**Subtotal**	**18**	**1**	**3**	**0**	**13.64%**	**22**
**Enterococcus faecalis**						
Amicacina	5	0	1	0	**16.67%**	6
Ampicilina	646	0	13	0	**1.97%**	659
Azitromicina	0	0	1	0	**100%**	1
Cefoxitina	1	0	2	0	**66.67%**	3
Ceftaroline	1	0	27	0	**96.43%**	28
Ceftriaxona	1	0	1	0	**50%**	2
Ciprofloxacina	139	31	155	0	**47.69%**	325
Clindamicina	3	0	159	0	**98.15%**	162
Cloranfenicol	1	0	1	0	**50%**	2
Dapsona	5	0	0	0	**0%**	5
Daptomicina	580	0	5	0	**0.85%**	585
Eritromicina	17	63	127	0	**61.35%**	207
Estreptomicina	17	0	11	0	**39.29%**	28
Estreptomicina 300 mcg	10	0	9	0	**47.37%**	19
Gentamicina	24	0	16	0	**40%**	40
Gentamicina 120 mg	11	0	7	0	**38.89%**	18
Imipenem	1	0	0	0	**0%**	1
Levofloxacina	76	2	47	0	**37.6%**	125
Linezolida	640	18	6	0	**0.9%**	664
Lomefloxacina	0	0	1	0	**100%**	1
Meropenem	3	0	0	0	**0%**	3
Minociclina	1	0	0	0	**0%**	1
Moxifloxacina	26	2	13	0	**31.71%**	41
Nitrofurantoína	292	32	15	0	**4.42%**	339
Norfloxacina	16	7	31	0	**57.41%**	54
Ofloxacina	0	0	1	0	**100%**	1
Oxacilina	0	0	1	0	**100%**	1
Penicilina	473	0	28	0	**5.59%**	501
Rifampicina	2	0	2	0	**50%**	4
Sulfametoxazol/trimetoprim	3	0	2	0	**40%**	5
Teicoplanina	190	0	7	0	**3.55%**	197
Tetraciclina	9	0	11	0	**55%**	20
Ticarcilina	1	0	0	0	**0%**	1
Tigeciclina	600	0	0	0	**0%**	600
Trimetoprim/Sulfametoxazol	30	0	33	0	**52.38%**	63
Vancomicina	622	2	23	0	**3.55%**	647
**Subtotal**	**4446**	**157**	**756**	**0**	**14.11%**	**5359**
**Enterococcus faecium**						
Amicacina	1	0	1	0	**50%**	2

Análise de Resistência Bacteriana - Teste de Sensibilidade

Microrganismo/Antibiótico	Sensível	Intermediário	Resistente	Não Testado	% Resistentes	Total
Ampicilina	34	0	126	0	**78.75%**	160
Ampicilina/Sulbactam	0	0	1	0	**100%**	1
Cefepime	1	0	0	0	**0%**	1
Cefoxitina	0	1	0	0	**0%**	1
Ceftazidima	1	0	0	0	**0%**	1
Ceftriaxona	0	0	1	0	**100%**	1
Cefuroxima	0	0	1	0	**100%**	1
Cefuroxima axetil	0	0	1	0	**100%**	1
Ciprofloxacina	13	3	73	0	**82.02%**	89
Clindamicina	5	0	49	0	**90.74%**	54
Dapsona	2	0	0	0	**0%**	2
Daptomicina	98	1	1	0	**1%**	100
Eritromicina	6	6	49	0	**80.33%**	61
Ertapenem	1	0	0	0	**0%**	1
Estreptomicina	4	0	11	0	**73.33%**	15
Estreptomicina 300 mcg	2	0	5	0	**71.43%**	7
Gentamicina	12	0	8	0	**40%**	20
Gentamicina 120 mg	7	0	2	0	**22.22%**	9
Levofloxacina	8	3	15	0	**57.69%**	26
Linezolida	126	24	10	0	**6.25%**	160
Meropenem	1	0	0	0	**0%**	1
Moxifloxacina	2	0	19	0	**90.48%**	21
Nitrofurantoína	11	9	40	0	**66.67%**	60
Norfloxacina	2	2	21	0	**84%**	25
Oxacilina	0	0	2	0	**100%**	2
Penicilina	31	0	96	0	**75.59%**	127
Piperacilina/Tazobactam	1	0	0	0	**0%**	1
Sulfametoxazol/trimetoprim	0	0	1	0	**100%**	1
Teicoplanina	19	0	38	0	**66.67%**	57
Tetraciclina	6	0	2	0	**25%**	8
Tigeciclina	48	0	0	0	**0%**	48
Trimetoprim/Sulfametoxazol	3	1	16	0	**80%**	20
Vancomicina	76	1	94	0	**54.97%**	171
**Subtotal**	**521**	**51**	**683**	**0**	**54.42%**	**1255**
**Enterococcus gallinarum**						
Ampicilina	2	0	0	0	**0%**	2
Clindamicina	0	0	2	0	**100%**	2
Eritromicina	2	0	0	0	**0%**	2
Levofloxacina	2	0	0	0	**0%**	2
Linezolida	1	1	0	0	**0%**	2
Nitrofurantoína	2	0	0	0	**0%**	2
Teicoplanina	2	0	0	0	**0%**	2
Tigeciclina	2	0	0	0	**0%**	2
Trimetoprim/Sulfametoxazol	1	0	1	0	**50%**	2
Vancomicina	1	0	1	0	**50%**	2
**Subtotal**	**15**	**1**	**4**	**0**	**20%**	**20**
**Enterococcus hirae**						
Ampicilina	3	0	0	0	**0%**	3
Daptomicina	3	0	0	0	**0%**	3
Linezolida	2	1	0	0	**0%**	3
Nitrofurantoína	2	0	0	0	**0%**	2
Penicilina	2	0	1	0	**33.33%**	3

Análise de Resistência Bacteriana - Teste de Sensibilidade

Microrganismo/Antibiótico	Sensível	Intermediário	Resistente	Não Testado	% Resistentes	Total
Vancomicina	3	0	0	0	**0%**	3
**Subtotal**	**15**	**1**	**1**	**0**	**5.88%**	**17**
**Enterococcus raffinosus**						
Ampicilina	1	0	2	0	**66.67%**	3
Daptomicina	2	0	0	0	**0%**	2
Linezolida	3	0	0	0	**0%**	3
Nitrofurantoína	0	0	1	0	**100%**	1
Penicilina	0	0	2	0	**100%**	2
Vancomicina	2	0	1	0	**33.33%**	3
**Subtotal**	**8**	**0**	**6**	**0**	**42.86%**	**14**
**Enterococcus sp.**						
Acido Nalidíxico	0	0	2	0	**100%**	2
Amicacina	1	0	0	0	**0%**	1
Amoxacilina/Ácido Clavulânico	1	0	0	0	**0%**	1
Ampicilina	28	0	6	0	**17.65%**	34
Ampicilina/Sulbactam	1	0	0	0	**0%**	1
Azitromicina	0	0	2	0	**100%**	2
Aztreonam	0	0	1	0	**100%**	1
Cefalotina	0	0	1	0	**100%**	1
Cefepime	1	0	4	0	**80%**	5
Cefotaxima	0	0	3	0	**100%**	3
Cefoxitina	0	0	5	0	**100%**	5
Ceftazidima	0	0	1	0	**100%**	1
Ceftriaxona	0	0	1	0	**100%**	1
Cefuroxima	0	0	4	0	**100%**	4
Ciprofloxacina	15	1	15	0	**48.39%**	31
Claritromicina	1	0	0	0	**0%**	1
Clindamicina	3	0	25	0	**89.29%**	28
Cloranfenicol	3	0	1	0	**25%**	4
Daptomicina	2	0	0	0	**0%**	2
Eritromicina	7	2	21	0	**70%**	30
Estreptomicina	4	0	2	0	**33.33%**	6
Estreptomicina 300 mcg	2	0	1	0	**33.33%**	3
Gentamicina	6	0	10	0	**62.5%**	16
Gentamicina 120 mg	2	0	1	0	**33.33%**	3
Imipenem	1	0	0	0	**0%**	1
Levofloxacina	6	0	4	0	**40%**	10
Linezolida	34	2	1	0	**2.7%**	37
Meropenem	2	0	0	0	**0%**	2
Minociclina	4	0	1	0	**20%**	5
Moxifloxacina	9	0	5	0	**35.71%**	14
Nitrofurantoína	7	2	1	0	**10%**	10
Norfloxacina	8	3	5	0	**31.25%**	16
Oxacilina	0	0	5	0	**100%**	5
Penicilina	29	0	13	0	**30.95%**	42
Rifampicina	8	0	3	0	**27.27%**	11
Sulfametoxazol/trimetoprim	3	0	1	0	**25%**	4
Teicoplanina	15	0	3	0	**16.67%**	18
Tetraciclina	3	0	3	0	**50%**	6
Tigeciclina	16	0	0	0	**0%**	16
Trimetoprim/Sulfametoxazol	1	0	5	0	**83.33%**	6
Vancomicina	38	0	10	0	**20.83%**	48

Análise de Resistência Bacteriana - Teste de Sensibilidade

Microrganismo/Antibiótico	Sensível	Intermediário	Resistente	Não Testado	% Resistentes	Total
**Subtotal**	**261**	**10**	**166**	**0**	**37.99%**	**437**
**Escherichia coli**						
Acido Nalidíxico	101	0	173	0	**63.14%**	274
Amicacina	2026	22	36	0	**1.73%**	2084
Amoxacilina/Ácido Clavulânico	665	192	147	0	**14.64%**	1004
Amoxicilina	2	2	3	0	**42.86%**	7
Ampicilina	529	22	1551	0	**73.79%**	2102
Ampicilina/Sulbactam	443	209	444	0	**40.51%**	1096
Azitromicina	6	0	1	0	**14.29%**	7
Aztreonam	64	0	9	0	**12.33%**	73
Carbenicilina	1	0	0	0	**0%**	1
Cefalotina	124	70	155	0	**44.41%**	349
Cefazolina	20	1	281	0	**93.05%**	302
Cefepime	1352	38	636	0	**31.39%**	2026
Cefotaxima	25	1	8	0	**23.53%**	34
Cefoxitina	1520	113	152	0	**8.52%**	1785
Ceftazidima	568	4	180	0	**23.94%**	752
Ceftriaxona	1223	5	869	0	**41.44%**	2097
Cefuroxima	876	64	618	0	**39.67%**	1558
Cefuroxima axetil	483	111	293	0	**33.03%**	887
Ciprofloxacina	970	52	1093	0	**51.68%**	2115
Clindamicina	1	0	1	0	**50%**	2
Cloranfenicol	47	0	8	0	**14.55%**	55
Colistin	104	1	2	0	**1.87%**	107
Daptomicina	1	0	0	0	**0%**	1
Doxiciclina	3	0	2	0	**40%**	5
Eritromicina	4	0	2	0	**33.33%**	6
Ertapenem	1823	11	221	0	**10.75%**	2055
Estreptomicina	2	0	2	0	**50%**	4
Fosfomicina - FOS 200	3	0	0	0	**0%**	3
Gentamicina	1684	14	380	0	**18.29%**	2078
Gentamicina 120 mg	1	0	0	0	**0%**	1
Imipenem	1772	11	89	0	**4.75%**	1872
Levofloxacina	473	6	558	0	**53.81%**	1037
Linezolida	3	0	0	0	**0%**	3
Lomefloxacina	0	0	3	0	**100%**	3
Meropenem	1981	14	150	0	**6.99%**	2145
Nitrofurantoína	639	37	35	0	**4.92%**	711
Norfloxacina	170	5	183	0	**51.12%**	358
Ofloxacina	2	0	2	0	**50%**	4
Oxacilina	1	0	1	0	**50%**	2
Penicilina	0	0	5	0	**100%**	5
Piperacilina	9	0	0	0	**0%**	9
Piperacilina/Tazobactam	1742	59	135	0	**6.97%**	1936
Polimixina "B"	4	0	0	0	**0%**	4
Rifampicina	2	0	0	0	**0%**	2
Sulfametoxazol/trimetoprim	94	0	113	0	**54.59%**	207
Sulfazotrim	3	0	8	0	**72.73%**	11
Sulfonamida	0	0	1	0	**100%**	1
Teicoplanina	2	0	0	0	**0%**	2
Tetraciclina	45	0	36	0	**44.44%**	81
Ticarcilina	3	0	0	0	**0%**	3

Análise de Resistência Bacteriana - Teste de Sensibilidade

Microrganismo/Antibiótico	Sensível	Intermediário	Resistente	Não Testado	% Resistentes	Total
Tigeciclina	1015	17	7	0	**0.67%**	1039
Tobramicina	5	2	1	0	**12.5%**	8
Trimetoprim/Sulfametoxazol	548	1	770	0	**58.38%**	1319
Vancomicina	2	0	0	0	**0%**	2
**Subtotal**	**23186**	**1084**	**9364**	**0**	**27.84%**	**33634**
**Escherichia coli enteroinvasora**						
Acido Nalidíxico	0	0	1	0	**100%**	1
Amicacina	1	0	0	0	**0%**	1
Amoxacilina/Ácido Clavulânico	0	1	0	0	**0%**	1
Ampicilina	0	0	1	0	**100%**	1
Cefalotina	0	0	1	0	**100%**	1
Cefepime	1	0	0	0	**0%**	1
Ceftriaxona	0	0	1	0	**100%**	1
Cefuroxima	0	0	1	0	**100%**	1
Cefuroxima axetil	0	0	1	0	**100%**	1
Ciprofloxacina	0	0	1	0	**100%**	1
Ertapenem	1	0	0	0	**0%**	1
Gentamicina	0	0	1	0	**100%**	1
Meropenem	1	0	0	0	**0%**	1
Nitrofurantoína	1	0	0	0	**0%**	1
Norfloxacina	0	0	1	0	**100%**	1
Piperacilina/Tazobactam	1	0	0	0	**0%**	1
Trimetoprim/Sulfametoxazol	0	0	1	0	**100%**	1
**Subtotal**	**6**	**1**	**10**	**0**	**58.82%**	**17**
**Escherichia coli enteropatogênica**						
Acido Nalidíxico	1	0	0	0	**0%**	1
Amicacina	1	0	0	0	**0%**	1
Amoxacilina/Ácido Clavulânico	1	1	0	0	**0%**	2
Ampicilina	1	0	2	0	**66.67%**	3
Ampicilina/Sulbactam	2	0	0	0	**0%**	2
Cefalotina	0	1	0	0	**0%**	1
Cefepime	1	0	0	0	**0%**	1
Cefotaxima	1	0	0	0	**0%**	1
Ceftriaxona	2	0	0	0	**0%**	2
Cefuroxima	1	0	0	0	**0%**	1
Cefuroxima axetil	1	0	0	0	**0%**	1
Ciprofloxacina	3	0	0	0	**0%**	3
Cloranfenicol	2	0	0	0	**0%**	2
Ertapenem	1	0	0	0	**0%**	1
Gentamicina	1	0	0	0	**0%**	1
Meropenem	1	0	0	0	**0%**	1
Nitrofurantoína	1	0	0	0	**0%**	1
Norfloxacina	1	0	0	0	**0%**	1
Piperacilina/Tazobactam	1	0	0	0	**0%**	1
Trimetoprim/Sulfametoxazol	0	0	1	0	**100%**	1
**Subtotal**	**23**	**2**	**3**	**0**	**10.71%**	**28**
**Escherichia coli enteropatogênica clássica B O142**						
Amicacina	1	0	0	0	**0%**	1
Ampicilina	0	0	1	0	**100%**	1
Cefepime	0	0	1	0	**100%**	1
Ceftriaxona	1	0	0	0	**0%**	1
Ciprofloxacina	1	0	0	0	**0%**	1

Análise de Resistência Bacteriana - Teste de Sensibilidade

Microrganismo/Antibiótico	Sensível	Intermediário	Resistente	Não Testado	% Resistentes	Total
Imipenem	1	0	0	0	**0%**	1
Meropenem	1	0	0	0	**0%**	1
Sulfametoxazol/trimetoprim	0	0	1	0	**100%**	1
**Subtotal**	**5**	**0**	**3**	**0**	**37.5%**	**8**
**Escherichia fergusonii**						
Amicacina	1	0	1	0	**50%**	2
Ampicilina	0	0	1	0	**100%**	1
Aztreonam	1	0	0	0	**0%**	1
Cefalotina	0	0	1	0	**100%**	1
Cefepime	1	0	0	0	**0%**	1
Cefoxitina	1	0	0	0	**0%**	1
Ceftazidima	1	0	0	0	**0%**	1
Ceftriaxona	1	0	0	0	**0%**	1
Ciprofloxacina	0	0	1	0	**100%**	1
Cloranfenicol	1	0	0	0	**0%**	1
Ertapenem	1	0	0	0	**0%**	1
Imipenem	1	0	0	0	**0%**	1
Meropenem	1	0	0	0	**0%**	1
Sulfazotrim	0	0	1	0	**100%**	1
Tetraciclina	0	0	1	0	**100%**	1
**Subtotal**	**10**	**0**	**6**	**0**	**37.5%**	**16**
**Escherichia sp.**						
Amicacina	1	0	0	0	**0%**	1
Ampicilina	0	0	1	0	**100%**	1
Ampicilina/Sulbactam	1	0	0	0	**0%**	1
Cefepime	1	0	0	0	**0%**	1
Cefoxitina	1	0	0	0	**0%**	1
Ceftazidima	1	0	0	0	**0%**	1
Ceftriaxona	1	0	0	0	**0%**	1
Cefuroxima	1	0	0	0	**0%**	1
Cefuroxima axetil	1	0	0	0	**0%**	1
Ciprofloxacina	1	0	0	0	**0%**	1
Ertapenem	1	0	0	0	**0%**	1
Gentamicina	1	0	0	0	**0%**	1
Imipenem	1	0	0	0	**0%**	1
Meropenem	1	0	0	0	**0%**	1
Piperacilina/Tazobactam	1	0	0	0	**0%**	1
Tigeciclina	1	0	0	0	**0%**	1
**Subtotal**	**15**	**0**	**1**	**0**	**6.25%**	**16**
**Hafnia alvei**						
Amicacina	1	0	0	0	**0%**	1
Ampicilina	0	0	1	0	**100%**	1
Ampicilina/Sulbactam	0	0	1	0	**100%**	1
Cefepime	1	0	0	0	**0%**	1
Cefoxitina	0	0	1	0	**100%**	1
Ceftazidima	1	0	0	0	**0%**	1
Ceftriaxona	0	0	1	0	**100%**	1
Cefuroxima	0	0	1	0	**100%**	1
Cefuroxima axetil	0	0	1	0	**100%**	1
Ciprofloxacina	0	0	1	0	**100%**	1
Ertapenem	0	0	1	0	**100%**	1
Gentamicina	1	0	0	0	**0%**	1

Análise de Resistência Bacteriana - Teste de Sensibilidade

Microrganismo/Antibiótico	Sensível	Intermediário	Resistente	Não Testado	% Resistentes	Total
Imipenem	1	0	0	0	**0%**	1
Meropenem	0	1	0	0	**0%**	1
Piperacilina/Tazobactam	1	0	0	0	**0%**	1
Tigeciclina	0	0	1	0	**100%**	1
**Subtotal**	**6**	**1**	**9**	**0**	**56.25%**	**16**
**Kingella denitrificans**						
Amicacina	1	0	0	0	**0%**	1
Ampicilina	0	0	1	0	**100%**	1
Ampicilina/Sulbactam	0	0	1	0	**100%**	1
Cefepime	0	0	1	0	**100%**	1
Cefoxitina	0	0	1	0	**100%**	1
Ceftriaxona	0	0	1	0	**100%**	1
Ciprofloxacina	0	0	1	0	**100%**	1
Ertapenem	0	0	1	0	**100%**	1
Gentamicina	0	1	0	0	**0%**	1
Levofloxacina	0	1	0	0	**0%**	1
Meropenem	0	0	1	0	**100%**	1
Piperacilina/Tazobactam	0	1	0	0	**0%**	1
Tigeciclina	0	1	0	0	**0%**	1
Trimetoprim/Sulfametoxazol	1	0	0	0	**0%**	1
**Subtotal**	**2**	**4**	**8**	**0**	**57.14%**	**14**
**Klebsiella aerogenes**						
Acido Nalidíxico	2	0	0	0	**0%**	2
Amicacina	54	3	4	0	**6.56%**	61
Amoxacilina/Ácido Clavulânico	0	0	20	0	**100%**	20
Amoxicilina	0	0	1	0	**100%**	1
Ampicilina	0	0	58	0	**100%**	58
Ampicilina/Sulbactam	0	0	28	0	**100%**	28
Aztreonam	0	0	1	0	**100%**	1
Cefalotina	0	0	2	0	**100%**	2
Cefazolina	0	0	19	0	**100%**	19
Cefepime	33	1	28	0	**45.16%**	62
Cefotaxima	1	0	0	0	**0%**	1
Cefoxitina	0	0	55	0	**100%**	55
Ceftazidima	3	0	6	0	**66.67%**	9
Ceftriaxona	32	0	27	0	**45.76%**	59
Cefuroxima	10	0	17	0	**62.96%**	27
Cefuroxima axetil	0	0	6	0	**100%**	6
Ciprofloxacina	40	3	19	0	**30.65%**	62
Ertapenem	39	1	18	0	**31.03%**	58
Gentamicina	44	0	17	0	**27.87%**	61
Imipenem	29	19	11	0	**18.64%**	59
Levofloxacina	42	0	4	0	**8.7%**	46
Meropenem	48	1	11	0	**18.33%**	60
Nitrofurantoína	2	3	3	0	**37.5%**	8
Norfloxacina	2	0	0	0	**0%**	2
Piperacilina/Tazobactam	36	6	17	0	**28.81%**	59
Polimixina "B"	2	0	0	0	**0%**	2
Sulfametoxazol/trimetoprim	3	0	0	0	**0%**	3
Tetraciclina	1	0	1	0	**50%**	2
Tigeciclina	28	5	4	0	**10.81%**	37
Trimetoprim/Sulfametoxazol	41	0	12	0	**22.64%**	53

Análise de Resistência Bacteriana - Teste de Sensibilidade

Microrganismo/Antibiótico	Sensível	Intermediário	Resistente	Não Testado	% Resistentes	Total
**Subtotal**	**492**	**42**	**389**	**0**	**42.15%**	**923**
**Klebsiella oxytoca**						
Acido Nalidíxico	1	0	0	0	**0%**	1
Amicacina	35	3	4	0	**9.52%**	42
Amoxacilina/Ácido Clavulânico	5	3	5	0	**38.46%**	13
Ampicilina	0	4	34	0	**89.47%**	38
Ampicilina/Sulbactam	7	1	20	0	**71.43%**	28
Cefalotina	3	0	0	0	**0%**	3
Cefazolina	5	1	9	0	**60%**	15
Cefepime	13	5	19	0	**51.35%**	37
Cefoxitina	25	0	14	0	**35.9%**	39
Ceftazidima	5	3	11	0	**57.89%**	19
Ceftriaxona	12	0	29	0	**70.73%**	41
Cefuroxima	8	0	19	0	**70.37%**	27
Cefuroxima axetil	4	0	13	0	**76.47%**	17
Ciprofloxacina	22	5	14	0	**34.15%**	41
Colistin	2	0	2	0	**50%**	4
Ertapenem	25	0	15	0	**37.5%**	40
Gentamicina	23	2	17	0	**40.48%**	42
Imipenem	28	0	13	0	**31.71%**	41
Levofloxacina	15	0	2	0	**11.76%**	17
Meropenem	28	1	13	0	**30.95%**	42
Nitrofurantoína	2	0	0	0	**0%**	2
Norfloxacina	1	0	0	0	**0%**	1
Piperacilina/Tazobactam	23	2	14	0	**35.9%**	39
Polimixina "B"	1	0	0	0	**0%**	1
Sulfametoxazol/trimetoprim	1	0	0	0	**0%**	1
Tetraciclina	1	0	0	0	**0%**	1
Tigeciclina	21	6	2	0	**6.9%**	29
Trimetoprim/Sulfametoxazol	14	0	9	0	**39.13%**	23
**Subtotal**	**330**	**36**	**278**	**0**	**43.17%**	**644**
**Klebsiella ozaenae**						
Amicacina	19	0	2	0	**9.52%**	21
Amoxacilina/Ácido Clavulânico	4	0	4	0	**50%**	8
Ampicilina	1	0	19	0	**95%**	20
Ampicilina/Sulbactam	4	0	11	0	**73.33%**	15
Cefazolina	0	0	8	0	**100%**	8
Cefepime	4	1	15	0	**75%**	20
Cefoxitina	10	1	10	0	**47.62%**	21
Ceftazidima	0	0	2	0	**100%**	2
Ceftriaxona	4	0	18	0	**81.82%**	22
Cefuroxima	1	0	5	0	**83.33%**	6
Ciprofloxacina	10	1	10	0	**47.62%**	21
Ertapenem	8	1	12	0	**57.14%**	21
Gentamicina	10	1	10	0	**47.62%**	21
Imipenem	13	1	8	0	**36.36%**	22
Levofloxacina	11	0	9	0	**45%**	20
Meropenem	14	0	8	0	**36.36%**	22
Nitrofurantoína	2	0	0	0	**0%**	2
Piperacilina/Tazobactam	11	3	7	0	**33.33%**	21
Tigeciclina	6	7	1	0	**7.14%**	14
Trimetoprim/Sulfametoxazol	12	0	10	0	**45.45%**	22

Análise de Resistência Bacteriana - Teste de Sensibilidade

Microrganismo/Antibiótico	Sensível	Intermediário	Resistente	Não Testado	% Resistentes	Total
**Subtotal**	**144**	**16**	**169**	**0**	**51.37%**	**329**
**Klebsiella planticola**						
Amicacina	0	0	1	0	**100%**	1
Ampicilina	0	0	1	0	**100%**	1
Ampicilina/Sulbactam	0	0	1	0	**100%**	1
Cefepime	0	0	1	0	**100%**	1
Cefoxitina	0	1	0	0	**0%**	1
Ceftriaxona	0	0	1	0	**100%**	1
Ciprofloxacina	0	0	1	0	**100%**	1
Ertapenem	0	0	1	0	**100%**	1
Gentamicina	0	0	1	0	**100%**	1
Imipenem	1	0	1	0	**50%**	2
Levofloxacina	0	0	1	0	**100%**	1
Meropenem	1	0	1	0	**50%**	2
Piperacilina/Tazobactam	0	0	1	0	**100%**	1
Tigeciclina	0	0	1	0	**100%**	1
Trimetoprim/Sulfametoxazol	0	0	1	0	**100%**	1
**Subtotal**	**2**	**1**	**14**	**0**	**82.35%**	**17**
**Klebsiella pneumoniae**						
Acido Nalidíxico	28	1	45	0	**60.81%**	74
Amicacina	1640	217	208	0	**10.07%**	2065
Amoxacilina/Ácido Clavulânico	169	139	299	0	**49.26%**	607
Amoxicilina	2	0	1	0	**33.33%**	3
Ampicilina	13	5	1982	0	**99.1%**	2000
Ampicilina/Sulbactam	308	61	1079	0	**74.52%**	1448
Azitromicina	2	0	0	0	**0%**	2
Aztreonam	7	0	15	0	**68.18%**	22
Cefalotina	41	1	73	0	**63.48%**	115
Cefazolina	13	2	389	0	**96.29%**	404
Cefepime	546	14	1474	0	**72.47%**	2034
Cefotaxima	6	0	15	0	**71.43%**	21
Cefoxitina	1082	107	776	0	**39.49%**	1965
Ceftaroline	0	0	2	0	**100%**	2
Ceftazidima	194	3	353	0	**64.18%**	550
Ceftriaxona	487	4	1560	0	**76.06%**	2051
Cefuroxima	264	7	786	0	**74.36%**	1057
Cefuroxima axetil	170	6	364	0	**67.41%**	540
Ciprofloxacina	556	124	1378	0	**66.96%**	2058
Cloranfenicol	5	0	6	0	**54.55%**	11
Colistin	64	0	8	0	**11.11%**	72
Doxiciclina	1	0	1	0	**50%**	2
Ertapenem	1108	73	875	0	**42.56%**	2056
Gentamicina	826	39	1175	0	**57.6%**	2040
Imipenem	1300	29	725	0	**35.3%**	2054
Levofloxacina	414	20	934	0	**68.27%**	1368
Lomefloxacina	0	0	3	0	**100%**	3
Meropenem	1286	28	790	0	**37.55%**	2104
Nitrofurantoína	68	53	194	0	**61.59%**	315
Norfloxacina	48	3	54	0	**51.43%**	105
Ofloxacina	0	0	1	0	**100%**	1
Penicilina	0	0	6	0	**100%**	6
Piperacilina	1	1	1	0	**33.33%**	3

Análise de Resistência Bacteriana - Teste de Sensibilidade

Microrganismo/Antibiótico	Sensível	Intermediário	Resistente	Não Testado	% Resistentes	Total
Piperacilina/Tazobactam	799	198	967	0	**49.24%**	1964
Polimixina "B"	37	0	4	0	**9.76%**	41
Rifampicina	0	0	1	0	**100%**	1
Sulfametoxazol/trimetoprim	18	0	73	0	**80.22%**	91
Sulfazotrim	1	0	1	0	**50%**	2
Tetraciclina	3	0	12	0	**80%**	15
Tigeciclina	767	375	266	0	**18.89%**	1408
Tobramicina	0	0	1	0	**100%**	1
Trimetoprim/Sulfametoxazol	450	0	1023	0	**69.45%**	1473
**Subtotal**	**12724**	**1510**	**17920**	**0**	**55.73%**	**32154**
**Klebsiella pneumoniae subsp. pneumoniae**						
Acido Nalidíxico	2	0	3	0	**60%**	5
Amicacina	53	7	13	0	**17.81%**	73
Amoxacilina/Ácido Clavulânico	4	1	7	0	**58.33%**	12
Ampicilina	1	0	71	0	**98.61%**	72
Ampicilina/Sulbactam	15	1	49	0	**75.38%**	65
Cefalotina	2	0	4	0	**66.67%**	6
Cefepime	30	0	42	0	**58.33%**	72
Cefotaxima	1	0	0	0	**0%**	1
Cefoxitina	36	7	22	0	**33.85%**	65
Ceftazidima	21	0	45	0	**68.18%**	66
Ceftriaxona	21	0	51	0	**70.83%**	72
Cefuroxima	18	0	52	0	**74.29%**	70
Cefuroxima axetil	17	0	52	0	**75.36%**	69
Ciprofloxacina	19	5	48	0	**66.67%**	72
Colistin	46	1	0	0	**0%**	47
Ertapenem	54	0	16	0	**22.86%**	70
Gentamicina	31	3	35	0	**50.72%**	69
Imipenem	52	0	15	0	**22.39%**	67
Levofloxacina	2	0	4	0	**66.67%**	6
Meropenem	57	0	15	0	**20.83%**	72
Nitrofurantoína	2	0	6	0	**75%**	8
Norfloxacina	2	0	7	0	**77.78%**	9
Piperacilina/Tazobactam	28	6	35	0	**50.72%**	69
Sulfametoxazol/trimetoprim	4	0	6	0	**60%**	10
Tetraciclina	0	0	1	0	**100%**	1
Tigeciclina	32	17	13	0	**20.97%**	62
Trimetoprim/Sulfametoxazol	2	1	3	0	**50%**	6
**Subtotal**	**552**	**49**	**615**	**0**	**50.58%**	**1216**
**Klebsiella sp.**						
Acido Nalidíxico	2	0	5	0	**71.43%**	7
Amicacina	71	3	25	0	**25.25%**	99
Amoxacilina/Ácido Clavulânico	7	2	31	0	**77.5%**	40
Ampicilina	1	0	97	0	**98.98%**	98
Ampicilina/Sulbactam	18	1	37	0	**66.07%**	56
Azitromicina	1	0	2	0	**66.67%**	3
Aztreonam	3	1	10	0	**71.43%**	14
Cefalotina	2	0	11	0	**84.62%**	13
Cefazolina	1	0	1	0	**50%**	2
Cefepime	41	1	69	0	**62.16%**	111
Cefotaxima	2	0	8	0	**80%**	10
Cefoxitina	42	2	50	0	**53.19%**	94

Análise de Resistência Bacteriana - Teste de Sensibilidade

Microrganismo/Antibiótico	Sensível	Intermediário	Resistente	Não Testado	% Resistentes	Total
Ceftazidima	31	1	50	0	**60.98%**	82
Ceftriaxona	36	0	66	0	**64.71%**	102
Cefuroxima	19	2	33	0	**61.11%**	54
Cefuroxima axetil	16	2	29	0	**61.7%**	47
Ciprofloxacina	34	1	58	0	**62.37%**	93
Cloranfenicol	7	0	10	0	**58.82%**	17
Colistin	1	0	0	0	**0%**	1
Doxiciclina	3	0	1	0	**25%**	4
Ertapenem	58	1	30	0	**33.71%**	89
Gentamicina	42	0	41	0	**49.4%**	83
Imipenem	70	8	31	0	**28.44%**	109
Levofloxacina	10	0	12	0	**54.55%**	22
Meropenem	72	4	33	0	**30.28%**	109
Nitrofurantoína	2	4	8	0	**57.14%**	14
Norfloxacina	6	0	7	0	**53.85%**	13
Ofloxacina	0	0	1	0	**100%**	1
Piperacilina/Tazobactam	31	4	29	0	**45.31%**	64
Sulfametoxazol/trimetoprim	6	0	32	0	**84.21%**	38
Sulfazotrim	0	0	5	0	**100%**	5
Tetraciclina	5	0	14	0	**73.68%**	19
Ticarcilina	0	1	0	0	**0%**	1
Tigeciclina	26	19	3	0	**6.25%**	48
Tobramicina	2	0	2	0	**50%**	4
Trimetoprim/Sulfametoxazol	7	0	6	0	**46.15%**	13
**Subtotal**	**675**	**57**	**847**	**0**	**53.64%**	**1579**
**Kluyvera ascorbata**						
Amicacina	6	0	0	0	**0%**	6
Amoxacilina/Ácido Clavulânico	0	0	1	0	**100%**	1
Ampicilina	0	0	4	0	**100%**	4
Ampicilina/Sulbactam	2	1	2	0	**40%**	5
Cefazolina	1	0	1	0	**50%**	2
Cefepime	3	0	3	0	**50%**	6
Cefoxitina	3	0	3	0	**50%**	6
Ceftriaxona	3	0	3	0	**50%**	6
Cefuroxima	1	0	0	0	**0%**	1
Ciprofloxacina	4	1	1	0	**16.67%**	6
Ertapenem	1	0	5	0	**83.33%**	6
Gentamicina	5	0	1	0	**16.67%**	6
Imipenem	4	1	1	0	**16.67%**	6
Levofloxacina	3	0	1	0	**25%**	4
Meropenem	5	0	1	0	**16.67%**	6
Piperacilina/Tazobactam	4	1	1	0	**16.67%**	6
Tigeciclina	4	1	0	0	**0%**	5
Trimetoprim/Sulfametoxazol	2	0	4	0	**66.67%**	6
**Subtotal**	**51**	**5**	**32**	**0**	**36.36%**	**88**
**Kluyvera sp.**						
Amicacina	1	0	0	0	**0%**	1
Ampicilina	0	0	1	0	**100%**	1
Cefalotina	1	0	0	0	**0%**	1
Cefazolina	1	0	0	0	**0%**	1
Cefoxitina	1	0	0	0	**0%**	1
Ceftazidima	1	0	0	0	**0%**	1

Análise de Resistência Bacteriana - Teste de Sensibilidade

Microrganismo/Antibiótico	Sensível	Intermediário	Resistente	Não Testado	% Resistentes	Total
Ceftriaxona	1	0	0	0	**0%**	1
Cefuroxima	1	0	0	0	**0%**	1
Ertapenem	1	0	0	0	**0%**	1
Imipenem	1	0	0	0	**0%**	1
Meropenem	1	0	0	0	**0%**	1
**Subtotal**	**10**	**0**	**1**	**0**	**9.09%**	**11**
**Leclercia adecarboxylata**						
Amicacina	1	0	0	0	**0%**	1
Amoxacilina/Ácido Clavulânico	0	0	1	0	**100%**	1
Ampicilina	0	0	1	0	**100%**	1
Cefazolina	0	0	1	0	**100%**	1
Cefepime	0	0	1	0	**100%**	1
Cefoxitina	0	0	1	0	**100%**	1
Ceftriaxona	0	0	1	0	**100%**	1
Cefuroxima	0	0	1	0	**100%**	1
Ciprofloxacina	0	0	1	0	**100%**	1
Ertapenem	0	0	1	0	**100%**	1
Gentamicina	0	0	1	0	**100%**	1
Imipenem	0	1	0	0	**0%**	1
Levofloxacina	0	0	1	0	**100%**	1
Meropenem	1	0	0	0	**0%**	1
Piperacilina/Tazobactam	0	0	1	0	**100%**	1
Trimetoprim/Sulfametoxazol	0	0	1	0	**100%**	1
**Subtotal**	**2**	**1**	**13**	**0**	**81.25%**	**16**
**Micrococcus luteus**						
Aztreonam	0	0	1	0	**100%**	1
Cefoxitina	0	0	1	0	**100%**	1
Clindamicina	0	0	1	0	**100%**	1
Eritromicina	0	0	1	0	**100%**	1
Gentamicina	0	0	1	0	**100%**	1
Minociclina	1	0	0	0	**0%**	1
Penicilina	0	0	1	0	**100%**	1
Rifampicina	1	0	0	0	**0%**	1
Vancomicina	1	0	0	0	**0%**	1
**Subtotal**	**3**	**0**	**6**	**0**	**66.67%**	**9**
**Morganella morganii**						
Acido Nalidíxico	0	0	6	0	**100%**	6
Amicacina	86	0	6	0	**6.52%**	92
Amoxacilina/Ácido Clavulânico	0	0	31	0	**100%**	31
Ampicilina	0	0	94	0	**100%**	94
Ampicilina/Sulbactam	5	19	40	0	**62.5%**	64
Aztreonam	1	0	0	0	**0%**	1
Cefalotina	0	0	10	0	**100%**	10
Cefazolina	0	0	31	0	**100%**	31
Cefepime	82	2	11	0	**11.58%**	95
Cefoxitina	38	30	18	0	**20.93%**	86
Ceftazidima	26	3	6	0	**17.14%**	35
Ceftriaxona	72	5	15	0	**16.3%**	92
Cefuroxima	0	1	57	0	**98.28%**	58
Cefuroxima axetil	0	1	36	0	**97.3%**	37
Ciprofloxacina	36	7	50	0	**53.76%**	93
Cloranfenicol	0	0	2	0	**100%**	2

Análise de Resistência Bacteriana - Teste de Sensibilidade

Microrganismo/Antibiótico	Sensível	Intermediário	Resistente	Não Testado	% Resistentes	Total
Colistin	0	0	9	0	**100%**	9
Ertapenem	87	0	3	0	**3.33%**	90
Gentamicina	57	13	22	0	**23.91%**	92
Gentamicina 120 mg	1	0	0	0	**0%**	1
Imipenem	5	4	46	0	**83.64%**	55
Levofloxacina	26	0	26	0	**50%**	52
Meropenem	90	1	3	0	**3.19%**	94
Nitrofurantoína	2	0	10	0	**83.33%**	12
Norfloxacina	3	0	6	0	**66.67%**	9
Piperacilina/Tazobactam	87	2	0	0	**0%**	89
Sulfametoxazol/trimetoprim	2	0	7	0	**77.78%**	9
Tetraciclina	3	0	2	0	**40%**	5
Tigeciclina	0	0	34	0	**100%**	34
Trimetoprim/Sulfametoxazol	23	0	34	0	**59.65%**	57
**Subtotal**	**732**	**88**	**615**	**0**	**42.86%**	**1435**
**Morganella morganii subsp. morganii**						
Acido Nalidíxico	1	0	1	0	**50%**	2
Amicacina	7	0	0	0	**0%**	7
Amoxacilina/Ácido Clavulânico	0	0	4	0	**100%**	4
Ampicilina	0	0	7	0	**100%**	7
Ampicilina/Sulbactam	0	0	3	0	**100%**	3
Cefalotina	0	0	2	0	**100%**	2
Cefazolina	0	0	2	0	**100%**	2
Cefepime	6	1	0	0	**0%**	7
Cefoxitina	2	3	0	0	**0%**	5
Ceftazidima	3	0	0	0	**0%**	3
Ceftriaxona	6	0	1	0	**14.29%**	7
Cefuroxima	0	0	7	0	**100%**	7
Cefuroxima axetil	0	0	5	0	**100%**	5
Ciprofloxacina	4	0	3	0	**42.86%**	7
Ertapenem	7	0	0	0	**0%**	7
Gentamicina	5	1	1	0	**14.29%**	7
Imipenem	1	0	2	0	**66.67%**	3
Levofloxacina	1	0	1	0	**50%**	2
Meropenem	7	0	0	0	**0%**	7
Nitrofurantoína	0	0	2	0	**100%**	2
Norfloxacina	1	0	1	0	**50%**	2
Piperacilina/Tazobactam	7	0	0	0	**0%**	7
Tigeciclina	0	0	1	0	**100%**	1
Trimetoprim/Sulfametoxazol	2	0	2	0	**50%**	4
**Subtotal**	**60**	**5**	**45**	**0**	**40.91%**	**110**
**Morganella morganii subsp. sibonii**						
Acido Nalidíxico	0	0	1	0	**100%**	1
Amicacina	1	0	0	0	**0%**	1
Amoxacilina/Ácido Clavulânico	0	0	1	0	**100%**	1
Ampicilina	0	0	1	0	**100%**	1
Cefalotina	0	0	1	0	**100%**	1
Cefepime	1	0	0	0	**0%**	1
Ceftriaxona	1	0	0	0	**0%**	1
Cefuroxima	0	0	1	0	**100%**	1
Cefuroxima axetil	0	0	1	0	**100%**	1
Ciprofloxacina	0	1	0	0	**0%**	1

Análise de Resistência Bacteriana - Teste de Sensibilidade

Microrganismo/Antibiótico	Sensível	Intermediário	Resistente	Não Testado	% Resistentes	Total
Ertapenem	1	0	0	0	**0%**	1
Gentamicina	1	0	0	0	**0%**	1
Meropenem	1	0	0	0	**0%**	1
Nitrofurantoína	0	0	1	0	**100%**	1
Norfloxacina	1	0	0	0	**0%**	1
Piperacilina/Tazobactam	0	1	0	0	**0%**	1
Trimetoprim/Sulfametoxazol	0	0	1	0	**100%**	1
**Subtotal**	**7**	**2**	**8**	**0**	**47.06%**	**17**
**Morganella sp.**						
Acido Nalidíxico	0	0	1	0	**100%**	1
Amicacina	1	0	0	0	**0%**	1
Amoxacilina/Ácido Clavulânico	0	0	1	0	**100%**	1
Ampicilina	0	0	1	0	**100%**	1
Cefalotina	0	0	1	0	**100%**	1
Cefepime	1	0	0	0	**0%**	1
Ceftriaxona	1	0	0	0	**0%**	1
Cefuroxima	0	0	1	0	**100%**	1
Cefuroxima axetil	0	0	1	0	**100%**	1
Ciprofloxacina	0	0	1	0	**100%**	1
Ertapenem	1	0	0	0	**0%**	1
Gentamicina	0	1	0	0	**0%**	1
Meropenem	1	0	0	0	**0%**	1
Nitrofurantoína	0	0	1	0	**100%**	1
Norfloxacina	0	0	1	0	**100%**	1
Piperacilina/Tazobactam	1	0	0	0	**0%**	1
Trimetoprim/Sulfametoxazol	1	0	0	0	**0%**	1
**Subtotal**	**7**	**1**	**9**	**0**	**52.94%**	**17**
**Neisseria meningitidis**						
Azitromicina	2	0	0	0	**0%**	2
Ceftriaxona	2	0	0	0	**0%**	2
Ciprofloxacina	2	0	0	0	**0%**	2
Cloranfenicol	2	0	0	0	**0%**	2
Meropenem	2	0	0	0	**0%**	2
Rifampicina	2	0	0	0	**0%**	2
Sulfametoxazol/trimetoprim	0	0	2	0	**100%**	2
**Subtotal**	**12**	**0**	**2**	**0**	**14.29%**	**14**
**Ochrobactrum anthropi**						
Amicacina	1	0	0	0	**0%**	1
Ciprofloxacina	1	0	0	0	**0%**	1
Gentamicina	1	0	0	0	**0%**	1
Imipenem	1	0	0	0	**0%**	1
Levofloxacina	1	0	0	0	**0%**	1
Meropenem	1	0	0	0	**0%**	1
Piperacilina/Tazobactam	0	0	1	0	**100%**	1
Trimetoprim/Sulfametoxazol	1	0	0	0	**0%**	1
**Subtotal**	**7**	**0**	**1**	**0**	**12.5%**	**8**
**Pantoea agglomerans**						
Amicacina	11	1	3	0	**20%**	15
Amoxacilina/Ácido Clavulânico	2	0	2	0	**50%**	4
Ampicilina	6	1	8	0	**53.33%**	15
Ampicilina/Sulbactam	6	0	5	0	**45.45%**	11
Cefalotina	1	0	0	0	**0%**	1

Análise de Resistência Bacteriana - Teste de Sensibilidade

Microrganismo/Antibiótico	Sensível	Intermediário	Resistente	Não Testado	% Resistentes	Total
Cefazolina	3	0	3	0	**50%**	6
Cefepime	6	4	5	0	**33.33%**	15
Cefoxitina	5	2	8	0	**53.33%**	15
Ceftazidima	2	0	0	0	**0%**	2
Ceftriaxona	7	0	8	0	**53.33%**	15
Cefuroxima	4	0	2	0	**33.33%**	6
Cefuroxima axetil	2	0	0	0	**0%**	2
Ciprofloxacina	8	0	7	0	**46.67%**	15
Ertapenem	6	1	7	0	**50%**	14
Gentamicina	9	1	5	0	**33.33%**	15
Imipenem	3	0	0	0	**0%**	3
Levofloxacina	7	1	5	0	**38.46%**	13
Meropenem	9	0	6	0	**40%**	15
Nitrofurantoína	1	0	0	0	**0%**	1
Piperacilina/Tazobactam	9	3	3	0	**20%**	15
Polimixina "B"	1	0	0	0	**0%**	1
Tigeciclina	7	4	0	0	**0%**	11
Trimetoprim/Sulfametoxazol	8	0	5	0	**38.46%**	13
**Subtotal**	**123**	**18**	**82**	**0**	**36.77%**	**223**
**Pantoea sp.**						
Acido Nalidíxico	0	0	1	0	**100%**	1
Amicacina	3	0	1	0	**25%**	4
Amoxacilina/Ácido Clavulânico	0	0	1	0	**100%**	1
Ampicilina	2	0	1	0	**33.33%**	3
Ampicilina/Sulbactam	2	0	1	0	**33.33%**	3
Cefepime	2	0	2	0	**50%**	4
Cefoxitina	0	1	2	0	**66.67%**	3
Ceftriaxona	1	0	2	0	**66.67%**	3
Ciprofloxacina	2	0	2	0	**50%**	4
Ertapenem	1	0	2	0	**66.67%**	3
Gentamicina	2	1	1	0	**25%**	4
Levofloxacina	1	0	2	0	**66.67%**	3
Meropenem	2	0	1	0	**33.33%**	3
Nitrofurantoína	1	0	0	0	**0%**	1
Norfloxacina	0	0	1	0	**100%**	1
Piperacilina/Tazobactam	2	0	2	0	**50%**	4
Sulfametoxazol/trimetoprim	1	0	0	0	**0%**	1
Tetraciclina	1	0	0	0	**0%**	1
Tigeciclina	2	1	0	0	**0%**	3
Trimetoprim/Sulfametoxazol	2	0	1	0	**33.33%**	3
**Subtotal**	**27**	**3**	**23**	**0**	**43.4%**	**53**
**Pluralibacter gergoviae**						
Amicacina	1	0	1	0	**50%**	2
Ampicilina	0	0	2	0	**100%**	2
Ampicilina/Sulbactam	0	1	1	0	**50%**	2
Cefazolina	0	0	1	0	**100%**	1
Cefepime	1	0	1	0	**50%**	2
Cefoxitina	0	0	2	0	**100%**	2
Ceftriaxona	1	0	1	0	**50%**	2
Ciprofloxacina	1	0	1	0	**50%**	2
Colistin	0	0	1	0	**100%**	1
Ertapenem	1	0	1	0	**50%**	2

Análise de Resistência Bacteriana - Teste de Sensibilidade

Microrganismo/Antibiótico	Sensível	Intermediário	Resistente	Não Testado	% Resistentes	Total
Gentamicina	1	0	1	0	**50%**	2
Imipenem	1	0	1	0	**50%**	2
Levofloxacina	1	1	0	0	**0%**	2
Meropenem	1	0	1	0	**50%**	2
Piperacilina/Tazobactam	1	0	1	0	**50%**	2
Tigeciclina	1	0	1	0	**50%**	2
Trimetoprim/Sulfametoxazol	1	0	1	0	**50%**	2
**Subtotal**	**12**	**2**	**18**	**0**	**56.25%**	**32**
**Proteus mirabilis**						
Acido Nalidíxico	10	0	4	0	**28.57%**	14
Amicacina	197	1	54	0	**21.43%**	252
Amoxacilina/Ácido Clavulânico	66	2	14	0	**17.07%**	82
Amoxicilina	1	0	1	0	**50%**	2
Ampicilina	96	3	146	0	**59.59%**	245
Ampicilina/Sulbactam	90	17	61	0	**36.31%**	168
Aztreonam	8	0	0	0	**0%**	8
Cefalotina	14	0	6	0	**30%**	20
Cefazolina	2	0	16	0	**88.89%**	18
Cefepime	167	5	74	0	**30.08%**	246
Cefotaxima	2	0	1	0	**33.33%**	3
Cefoxitina	199	13	18	0	**7.83%**	230
Ceftaroline	0	0	1	0	**100%**	1
Ceftazidima	97	2	24	0	**19.51%**	123
Ceftriaxona	152	3	92	0	**37.25%**	247
Cefuroxima	104	0	72	0	**40.91%**	176
Cefuroxima axetil	63	1	52	0	**44.83%**	116
Ciprofloxacina	148	25	76	0	**30.52%**	249
Cloranfenicol	2	0	2	0	**50%**	4
Colistin	0	0	24	0	**100%**	24
Eritromicina	2	0	0	0	**0%**	2
Ertapenem	213	1	27	0	**11.2%**	241
Gentamicina	146	15	80	0	**33.2%**	241
Imipenem	24	3	1	0	**3.57%**	28
Levofloxacina	55	3	39	0	**40.21%**	97
Meropenem	222	6	25	0	**9.88%**	253
Nitrofurantoína	1	1	40	0	**95.24%**	42
Norfloxacina	15	0	2	0	**11.76%**	17
Ofloxacina	2	0	0	0	**0%**	2
Penicilina	1	0	0	0	**0%**	1
Piperacilina/Tazobactam	212	6	16	0	**6.84%**	234
Sulfametoxazol/trimetoprim	9	0	11	0	**55%**	20
Tetraciclina	0	0	7	0	**100%**	7
Tigeciclina	5	2	86	0	**92.47%**	93
Tobramicina	0	0	1	0	**100%**	1
Trimetoprim/Sulfametoxazol	76	0	46	0	**37.7%**	122
**Subtotal**	**2401**	**109**	**1119**	**0**	**30.83%**	**3629**
**Proteus penneri**						
Amicacina	3	0	0	0	**0%**	3
Ampicilina	0	0	3	0	**100%**	3
Ampicilina/Sulbactam	0	1	2	0	**66.67%**	3
Cefepime	3	0	0	0	**0%**	3
Cefoxitina	2	1	0	0	**0%**	3

Análise de Resistência Bacteriana - Teste de Sensibilidade

Microrganismo/Antibiótico	Sensível	Intermediário	Resistente	Não Testado	% Resistentes	Total
Ceftazidima	3	0	0	0	**0%**	3
Ceftriaxona	2	0	1	0	**33.33%**	3
Cefuroxima	0	0	3	0	**100%**	3
Cefuroxima axetil	0	0	3	0	**100%**	3
Ciprofloxacina	2	0	1	0	**33.33%**	3
Colistin	0	0	1	0	**100%**	1
Ertapenem	3	0	0	0	**0%**	3
Gentamicina	2	1	0	0	**0%**	3
Meropenem	3	0	0	0	**0%**	3
Piperacilina/Tazobactam	3	0	0	0	**0%**	3
Tigeciclina	1	0	2	0	**66.67%**	3
**Subtotal**	**27**	**3**	**16**	**0**	**34.78%**	**46**
**Proteus sp.**						
Amicacina	2	0	1	0	**33.33%**	3
Amoxacilina/Ácido Clavulânico	2	0	1	0	**33.33%**	3
Ampicilina	1	0	3	0	**75%**	4
Ampicilina/Sulbactam	1	0	0	0	**0%**	1
Aztreonam	4	0	0	0	**0%**	4
Cefepime	3	0	1	0	**25%**	4
Cefotaxima	0	0	1	0	**100%**	1
Cefoxitina	5	0	0	0	**0%**	5
Ceftazidima	4	0	1	0	**20%**	5
Ceftriaxona	2	0	1	0	**33.33%**	3
Cefuroxima	1	0	2	0	**66.67%**	3
Cefuroxima axetil	0	0	1	0	**100%**	1
Ciprofloxacina	1	0	3	0	**75%**	4
Cloranfenicol	1	0	1	0	**50%**	2
Doxiciclina	0	0	1	0	**100%**	1
Ertapenem	4	0	0	0	**0%**	4
Gentamicina	2	0	1	0	**33.33%**	3
Imipenem	4	0	0	0	**0%**	4
Levofloxacina	2	0	0	0	**0%**	2
Meropenem	5	0	0	0	**0%**	5
Ofloxacina	1	0	0	0	**0%**	1
Piperacilina/Tazobactam	1	0	0	0	**0%**	1
Sulfametoxazol/trimetoprim	1	0	1	0	**50%**	2
Tetraciclina	0	0	1	0	**100%**	1
Tigeciclina	0	0	1	0	**100%**	1
Tobramicina	0	0	1	0	**100%**	1
**Subtotal**	**47**	**0**	**22**	**0**	**31.88%**	**69**
**Proteus vulgaris**						
Acido Nalidíxico	1	0	0	0	**0%**	1
Amicacina	13	0	2	0	**13.33%**	15
Amoxacilina/Ácido Clavulânico	2	2	0	0	**0%**	4
Ampicilina	0	0	13	0	**100%**	13
Ampicilina/Sulbactam	6	0	5	0	**45.45%**	11
Cefalotina	1	0	0	0	**0%**	1
Cefazolina	0	0	5	0	**100%**	5
Cefepime	10	2	3	0	**20%**	15
Cefoxitina	9	3	2	0	**14.29%**	14
Ceftazidima	5	1	1	0	**14.29%**	7
Ceftriaxona	5	1	7	0	**53.85%**	13

Análise de Resistência Bacteriana - Teste de Sensibilidade

Microrganismo/Antibiótico	Sensível	Intermediário	Resistente	Não Testado	% Resistentes	Total
Cefuroxima	0	0	9	0	**100%**	9
Cefuroxima axetil	0	0	5	0	**100%**	5
Ciprofloxacina	11	1	3	0	**20%**	15
Colistin	0	0	1	0	**100%**	1
Ertapenem	11	0	1	0	**8.33%**	12
Gentamicina	10	1	4	0	**26.67%**	15
Imipenem	1	0	8	0	**88.89%**	9
Levofloxacina	5	0	0	0	**0%**	5
Meropenem	14	0	1	0	**6.67%**	15
Nitrofurantoína	1	0	2	0	**66.67%**	3
Norfloxacina	1	0	0	0	**0%**	1
Piperacilina/Tazobactam	14	0	1	0	**6.67%**	15
Sulfametoxazol/trimetoprim	2	0	0	0	**0%**	2
Tigeciclina	0	0	4	0	**100%**	4
Trimetoprim/Sulfametoxazol	7	0	1	0	**12.5%**	8
**Subtotal**	**129**	**11**	**78**	**0**	**35.78%**	**218**
**Providencia alcalifaciens**						
Amicacina	1	0	0	0	**0%**	1
Amoxacilina/Ácido Clavulânico	0	0	1	0	**100%**	1
Cefepime	1	0	0	0	**0%**	1
Cefoxitina	1	0	0	0	**0%**	1
Ceftriaxona	1	0	0	0	**0%**	1
Cefuroxima	1	0	0	0	**0%**	1
Ciprofloxacina	1	0	0	0	**0%**	1
Ertapenem	0	0	1	0	**100%**	1
Gentamicina	1	0	0	0	**0%**	1
Imipenem	1	0	0	0	**0%**	1
Meropenem	0	0	1	0	**100%**	1
Piperacilina/Tazobactam	1	0	0	0	**0%**	1
Trimetoprim/Sulfametoxazol	1	0	0	0	**0%**	1
**Subtotal**	**10**	**0**	**3**	**0**	**23.08%**	**13**
**Providencia rettgeri**						
Acido Nalidíxico	0	0	1	0	**100%**	1
Amicacina	7	0	2	0	**22.22%**	9
Amoxacilina/Ácido Clavulânico	0	0	3	0	**100%**	3
Ampicilina	0	0	7	0	**100%**	7
Ampicilina/Sulbactam	3	1	1	0	**20%**	5
Cefalotina	0	0	1	0	**100%**	1
Cefazolina	0	0	3	0	**100%**	3
Cefepime	5	1	3	0	**33.33%**	9
Cefoxitina	5	0	3	0	**37.5%**	8
Ceftazidima	2	0	0	0	**0%**	2
Ceftriaxona	5	0	4	0	**44.44%**	9
Cefuroxima	3	0	3	0	**50%**	6
Cefuroxima axetil	3	0	0	0	**0%**	3
Ciprofloxacina	4	0	5	0	**55.56%**	9
Ertapenem	6	0	3	0	**33.33%**	9
Gentamicina	6	1	2	0	**22.22%**	9
Imipenem	0	2	4	0	**66.67%**	6
Levofloxacina	3	1	2	0	**33.33%**	6
Meropenem	6	0	3	0	**33.33%**	9
Nitrofurantoína	0	0	3	0	**100%**	3

Análise de Resistência Bacteriana - Teste de Sensibilidade

Microrganismo/Antibiótico	Sensível	Intermediário	Resistente	Não Testado	% Resistentes	Total
Norfloxacina	0	0	1	0	**100%**	1
Piperacilina	1	0	0	0	**0%**	1
Piperacilina/Tazobactam	7	0	1	0	**12.5%**	8
Tigeciclina	0	0	2	0	**100%**	2
Trimetoprim/Sulfametoxazol	3	0	4	0	**57.14%**	7
**Subtotal**	**69**	**6**	**61**	**0**	**44.85%**	**136**
**Providencia rustigianii**						
Amicacina	1	0	1	0	**50%**	2
Amoxacilina/Ácido Clavulânico	1	0	1	0	**50%**	2
Ampicilina	0	0	1	0	**100%**	1
Cefazolina	0	0	1	0	**100%**	1
Cefepime	1	0	1	0	**50%**	2
Cefoxitina	2	0	0	0	**0%**	2
Ceftriaxona	1	0	1	0	**50%**	2
Cefuroxima	1	0	1	0	**50%**	2
Ciprofloxacina	0	0	2	0	**100%**	2
Ertapenem	2	0	0	0	**0%**	2
Gentamicina	1	0	1	0	**50%**	2
Imipenem	1	0	1	0	**50%**	2
Levofloxacina	0	0	2	0	**100%**	2
Meropenem	1	1	0	0	**0%**	2
Nitrofurantoína	0	0	2	0	**100%**	2
Piperacilina/Tazobactam	2	0	0	0	**0%**	2
Trimetoprim/Sulfametoxazol	1	0	1	0	**50%**	2
**Subtotal**	**15**	**1**	**16**	**0**	**50%**	**32**
**Providencia sp.**						
Amicacina	1	0	0	0	**0%**	1
Amoxacilina/Ácido Clavulânico	1	0	0	0	**0%**	1
Ampicilina	0	0	1	0	**100%**	1
Cefepime	1	0	0	0	**0%**	1
Cefotaxima	1	0	0	0	**0%**	1
Ceftazidima	1	0	0	0	**0%**	1
Ciprofloxacina	1	0	0	0	**0%**	1
Imipenem	1	0	0	0	**0%**	1
Levofloxacina	1	0	0	0	**0%**	1
Meropenem	1	0	0	0	**0%**	1
Sulfametoxazol/trimetoprim	1	0	0	0	**0%**	1
**Subtotal**	**10**	**0**	**1**	**0**	**9.09%**	**11**
**Providencia stuartii**						
Acido Nalidíxico	1	0	4	0	**80%**	5
Amicacina	55	5	16	0	**21.05%**	76
Amoxacilina/Ácido Clavulânico	0	0	22	0	**100%**	22
Ampicilina	1	0	70	0	**98.59%**	71
Ampicilina/Sulbactam	4	3	47	0	**87.04%**	54
Aztreonam	0	0	1	0	**100%**	1
Cefalotina	0	0	6	0	**100%**	6
Cefazolina	0	0	25	0	**100%**	25
Cefepime	25	4	47	0	**61.84%**	76
Cefoxitina	43	9	19	0	**26.76%**	71
Ceftazidima	13	5	3	0	**14.29%**	21
Ceftriaxona	19	0	56	0	**74.67%**	75
Cefuroxima	9	4	26	0	**66.67%**	39

Análise de Resistência Bacteriana - Teste de Sensibilidade

Microrganismo/Antibiótico	Sensível	Intermediário	Resistente	Não Testado	% Resistentes	Total
Cefuroxima axetil	7	2	15	0	**62.5%**	24
Ciprofloxacina	14	3	60	0	**77.92%**	77
Colistin	0	0	7	0	**100%**	7
Ertapenem	51	0	23	0	**31.08%**	74
Gentamicina	2	1	63	0	**95.45%**	66
Imipenem	2	3	49	0	**90.74%**	54
Levofloxacina	6	1	42	0	**85.71%**	49
Meropenem	57	1	18	0	**23.68%**	76
Nitrofurantoína	0	0	14	0	**100%**	14
Norfloxacina	1	0	6	0	**85.71%**	7
Piperacilina/Tazobactam	56	0	18	0	**24.32%**	74
Sulfametoxazol/trimetoprim	0	0	3	0	**100%**	3
Tetraciclina	0	0	2	0	**100%**	2
Tigeciclina	0	0	21	0	**100%**	21
Trimetoprim/Sulfametoxazol	33	0	22	0	**40%**	55
**Subtotal**	**399**	**41**	**705**	**0**	**61.57%**	**1145**
**Pseudomonas aeruginosa**						
Acido Nalidíxico	1	0	17	0	**94.44%**	18
Amicacina	886	13	130	0	**12.63%**	1029
Amoxacilina/Ácido Clavulânico	0	1	40	0	**97.56%**	41
Amoxicilina	1	0	0	0	**0%**	1
Ampicilina	1	1	277	0	**99.28%**	279
Ampicilina/Sulbactam	8	2	235	0	**95.92%**	245
Azitromicina	4	0	0	0	**0%**	4
Aztreonam	23	1	4	0	**14.29%**	28
Cefalotina	0	0	7	0	**100%**	7
Cefazolina	0	0	3	0	**100%**	3
Cefepime	724	18	301	0	**28.86%**	1043
Cefotaxima	1	0	10	0	**90.91%**	11
Cefoxitina	7	2	274	0	**96.82%**	283
Ceftazidima	682	71	254	0	**25.22%**	1007
Ceftriaxona	17	7	273	0	**91.92%**	297
Cefuroxima	2	2	269	0	**98.53%**	273
Cefuroxima axetil	2	2	247	0	**98.41%**	251
Ciprofloxacina	731	39	265	0	**25.6%**	1035
Cloranfenicol	1	0	13	0	**92.86%**	14
Colistin	38	3	2	0	**4.65%**	43
Doxiciclina	1	0	0	0	**0%**	1
Ertapenem	6	1	15	0	**68.18%**	22
Gentamicina	727	47	214	0	**21.66%**	988
Gentamicina 120 mg	2	0	1	0	**33.33%**	3
Imipenem	538	132	386	0	**36.55%**	1056
Levofloxacina	428	73	175	0	**25.89%**	676
Linezolida	1	0	0	0	**0%**	1
Lomefloxacina	0	0	1	0	**100%**	1
Meropenem	683	63	319	0	**29.95%**	1065
Minociclina	0	0	1	0	**100%**	1
Nitrofurantoína	0	0	25	0	**100%**	25
Norfloxacina	22	0	9	0	**29.03%**	31
Ofloxacina	1	0	3	0	**75%**	4
Penicilina	0	0	2	0	**100%**	2
Piperacilina	3	1	1	0	**20%**	5

Análise de Resistência Bacteriana - Teste de Sensibilidade

Microrganismo/Antibiótico	Sensível	Intermediário	Resistente	Não Testado	% Resistentes	Total
Piperacilina/Tazobactam	707	114	113	0	**12.1%**	934
Polimixina "B"	17	0	6	0	**26.09%**	23
Rifampicina	1	0	0	0	**0%**	1
Sulfametoxazol/trimetoprim	2	0	48	0	**96%**	50
Sulfazotrim	0	0	1	0	**100%**	1
Tetraciclina	3	0	28	0	**90.32%**	31
Ticarcilina	0	0	1	0	**100%**	1
Tigeciclina	14	4	215	0	**92.27%**	233
Tobramicina	4	0	1	0	**20%**	5
Trimetoprim/Sulfametoxazol	1	0	27	0	**96.43%**	28
Vancomicina	1	0	0	0	**0%**	1
**Subtotal**	**6291**	**597**	**4213**	**0**	**37.95%**	**11101**
**Pseudomonas fluorescens**						
Amicacina	2	0	1	0	**33.33%**	3
Ampicilina	1	0	1	0	**50%**	2
Ampicilina/Sulbactam	1	0	1	0	**50%**	2
Cefepime	4	0	0	0	**0%**	4
Cefoxitina	1	0	2	0	**66.67%**	3
Ceftazidima	2	2	0	0	**0%**	4
Ceftriaxona	2	1	0	0	**0%**	3
Cefuroxima	1	0	1	0	**50%**	2
Cefuroxima axetil	1	0	1	0	**50%**	2
Ciprofloxacina	3	0	1	0	**25%**	4
Colistin	1	0	0	0	**0%**	1
Gentamicina	1	0	2	0	**66.67%**	3
Imipenem	4	0	0	0	**0%**	4
Levofloxacina	1	0	0	0	**0%**	1
Meropenem	4	0	0	0	**0%**	4
Piperacilina/Tazobactam	3	1	0	0	**0%**	4
Sulfametoxazol/trimetoprim	0	0	1	0	**100%**	1
Tetraciclina	1	0	0	0	**0%**	1
Tigeciclina	2	0	0	0	**0%**	2
Trimetoprim/Sulfametoxazol	1	0	0	0	**0%**	1
**Subtotal**	**36**	**4**	**11**	**0**	**21.57%**	**51**
**Pseudomonas luteola**						
Amicacina	4	0	0	0	**0%**	4
Ampicilina	1	2	1	0	**25%**	4
Ampicilina/Sulbactam	3	0	1	0	**25%**	4
Cefepime	4	0	0	0	**0%**	4
Cefoxitina	3	0	1	0	**25%**	4
Ceftazidima	3	1	0	0	**0%**	4
Ceftriaxona	3	1	0	0	**0%**	4
Cefuroxima	3	0	1	0	**25%**	4
Cefuroxima axetil	3	0	1	0	**25%**	4
Ciprofloxacina	4	0	0	0	**0%**	4
Colistin	0	0	1	0	**100%**	1
Gentamicina	4	0	0	0	**0%**	4
Imipenem	4	0	0	0	**0%**	4
Meropenem	4	0	0	0	**0%**	4
Piperacilina/Tazobactam	4	0	0	0	**0%**	4
Tigeciclina	3	0	1	0	**25%**	4
**Subtotal**	**50**	**4**	**7**	**0**	**11.48%**	**61**

Análise de Resistência Bacteriana - Teste de Sensibilidade

Microrganismo/Antibiótico	Sensível	Intermediário	Resistente	Não Testado	% Resistentes	Total
**Pseudomonas mendocina**						
Amicacina	1	1	0	0	**0%**	2
Ampicilina	0	0	1	0	**100%**	1
Ampicilina/Sulbactam	0	0	1	0	**100%**	1
Cefepime	1	0	1	0	**50%**	2
Cefoxitina	0	0	1	0	**100%**	1
Ceftazidima	2	0	0	0	**0%**	2
Ceftriaxona	0	1	0	0	**0%**	1
Cefuroxima	0	0	1	0	**100%**	1
Cefuroxima axetil	0	0	1	0	**100%**	1
Ciprofloxacina	1	1	0	0	**0%**	2
Gentamicina	1	0	1	0	**50%**	2
Imipenem	2	0	0	0	**0%**	2
Levofloxacina	1	0	0	0	**0%**	1
Meropenem	2	0	0	0	**0%**	2
Piperacilina/Tazobactam	2	0	0	0	**0%**	2
Tigeciclina	0	1	0	0	**0%**	1
Trimetoprim/Sulfametoxazol	1	0	0	0	**0%**	1
**Subtotal**	**14**	**4**	**7**	**0**	**28%**	**25**
**Pseudomonas oryzihabitans**						
Amicacina	2	0	0	0	**0%**	2
Ampicilina	1	0	0	0	**0%**	1
Ampicilina/Sulbactam	1	0	0	0	**0%**	1
Cefepime	2	0	0	0	**0%**	2
Cefoxitina	0	0	1	0	**100%**	1
Ceftazidima	2	0	0	0	**0%**	2
Ceftriaxona	1	0	0	0	**0%**	1
Cefuroxima	0	0	1	0	**100%**	1
Cefuroxima axetil	0	0	1	0	**100%**	1
Ciprofloxacina	2	0	0	0	**0%**	2
Gentamicina	2	0	0	0	**0%**	2
Imipenem	2	0	0	0	**0%**	2
Levofloxacina	1	0	0	0	**0%**	1
Meropenem	1	0	0	0	**0%**	1
Piperacilina/Tazobactam	2	0	0	0	**0%**	2
Tigeciclina	1	0	0	0	**0%**	1
Trimetoprim/Sulfametoxazol	1	0	0	0	**0%**	1
**Subtotal**	**21**	**0**	**3**	**0**	**12.5%**	**24**
**Pseudomonas pseudoalcaligenes**						
Amicacina	0	1	0	0	**0%**	1
Cefepime	0	1	0	0	**0%**	1
Ceftazidima	0	0	1	0	**100%**	1
Ciprofloxacina	0	0	1	0	**100%**	1
Gentamicina	0	0	1	0	**100%**	1
Imipenem	0	0	1	0	**100%**	1
Levofloxacina	0	0	1	0	**100%**	1
Meropenem	0	0	1	0	**100%**	1
Piperacilina/Tazobactam	0	0	1	0	**100%**	1
Trimetoprim/Sulfametoxazol	1	0	0	0	**0%**	1
**Subtotal**	**1**	**2**	**7**	**0**	**70%**	**10**
**Pseudomonas putida**						
Amicacina	20	1	2	0	**8.7%**	23

Análise de Resistência Bacteriana - Teste de Sensibilidade

Microrganismo/Antibiótico	Sensível	Intermediário	Resistente	Não Testado	% Resistentes	Total
Amoxacilina/Ácido Clavulânico	0	0	1	0	**100%**	1
Ampicilina	0	0	2	0	**100%**	2
Ampicilina/Sulbactam	0	0	1	0	**100%**	1
Cefepime	18	1	4	0	**17.39%**	23
Cefoxitina	0	0	1	0	**100%**	1
Ceftazidima	17	0	5	0	**22.73%**	22
Ceftriaxona	0	2	0	0	**0%**	2
Cefuroxima	0	0	2	0	**100%**	2
Cefuroxima axetil	0	0	2	0	**100%**	2
Ciprofloxacina	15	0	8	0	**34.78%**	23
Gentamicina	19	0	4	0	**17.39%**	23
Imipenem	18	1	3	0	**13.64%**	22
Levofloxacina	12	1	8	0	**38.1%**	21
Meropenem	15	4	4	0	**17.39%**	23
Piperacilina	1	0	0	0	**0%**	1
Piperacilina/Tazobactam	16	2	3	0	**14.29%**	21
Sulfametoxazol/trimetoprim	0	0	1	0	**100%**	1
Tetraciclina	0	0	2	0	**100%**	2
Tigeciclina	1	0	1	0	**50%**	2
Trimetoprim/Sulfametoxazol	6	0	15	0	**71.43%**	21
**Subtotal**	**158**	**12**	**69**	**0**	**28.87%**	**239**
**Pseudomonas sp.**						
Acido Nalidíxico	0	0	1	0	**100%**	1
Amicacina	8	1	7	0	**43.75%**	16
Amoxacilina/Ácido Clavulânico	0	0	2	0	**100%**	2
Ampicilina	0	1	8	0	**88.89%**	9
Ampicilina/Sulbactam	1	0	8	0	**88.89%**	9
Aztreonam	2	1	1	0	**25%**	4
Cefepime	13	0	5	0	**27.78%**	18
Cefoxitina	0	1	6	0	**85.71%**	7
Ceftazidima	10	2	2	0	**14.29%**	14
Ceftriaxona	3	1	7	0	**63.64%**	11
Cefuroxima	0	0	8	0	**100%**	8
Cefuroxima axetil	0	0	7	0	**100%**	7
Ciprofloxacina	10	1	5	0	**31.25%**	16
Colistin	1	0	1	0	**50%**	2
Ertapenem	1	0	1	0	**50%**	2
Gentamicina	7	2	8	0	**47.06%**	17
Imipenem	12	1	9	0	**40.91%**	22
Levofloxacina	3	0	0	0	**0%**	3
Meropenem	15	1	6	0	**27.27%**	22
Ofloxacina	1	0	1	0	**50%**	2
Piperacilina/Tazobactam	9	2	3	0	**21.43%**	14
Sulfametoxazol/trimetoprim	0	0	4	0	**100%**	4
Sulfazotrim	0	0	1	0	**100%**	1
Tetraciclina	0	0	3	0	**100%**	3
Tigeciclina	2	0	4	0	**66.67%**	6
Tobramicina	1	0	1	0	**50%**	2
Trimetoprim/Sulfametoxazol	1	0	1	0	**50%**	2
**Subtotal**	**100**	**14**	**110**	**0**	**49.11%**	**224**
**Pseudomonas stutzeri**						
Amicacina	2	0	0	0	**0%**	2

Análise de Resistência Bacteriana - Teste de Sensibilidade

Microrganismo/Antibiótico	Sensível	Intermediário	Resistente	Não Testado	% Resistentes	Total
Ampicilina	2	0	0	0	**0%**	2
Ampicilina/Sulbactam	2	0	0	0	**0%**	2
Cefepime	2	0	0	0	**0%**	2
Cefoxitina	0	0	2	0	**100%**	2
Ceftazidima	2	0	0	0	**0%**	2
Ceftriaxona	2	0	0	0	**0%**	2
Cefuroxima	0	0	2	0	**100%**	2
Cefuroxima axetil	0	0	2	0	**100%**	2
Ciprofloxacina	2	0	0	0	**0%**	2
Gentamicina	2	0	0	0	**0%**	2
Imipenem	2	0	0	0	**0%**	2
Meropenem	2	0	0	0	**0%**	2
Piperacilina/Tazobactam	2	0	0	0	**0%**	2
Tigeciclina	2	0	0	0	**0%**	2
**Subtotal**	**24**	**0**	**6**	**0**	**20%**	**30**
**Ralstonia pickettii**						
Amicacina	7	1	10	0	**55.56%**	18
Ampicilina	2	0	0	0	**0%**	2
Ampicilina/Sulbactam	2	0	0	0	**0%**	2
Cefepime	2	0	0	0	**0%**	2
Cefoxitina	1	1	0	0	**0%**	2
Ceftazidima	0	3	15	0	**83.33%**	18
Ceftriaxona	2	0	0	0	**0%**	2
Cefuroxima	2	0	0	0	**0%**	2
Cefuroxima axetil	2	0	0	0	**0%**	2
Ciprofloxacina	17	1	0	0	**0%**	18
Gentamicina	7	0	11	0	**61.11%**	18
Imipenem	2	0	0	0	**0%**	2
Levofloxacina	16	0	0	0	**0%**	16
Meropenem	2	0	0	0	**0%**	2
Piperacilina/Tazobactam	10	3	5	0	**27.78%**	18
Tigeciclina	2	0	0	0	**0%**	2
Trimetoprim/Sulfametoxazol	16	0	0	0	**0%**	16
**Subtotal**	**92**	**9**	**41**	**0**	**28.87%**	**142**
**Raoultella ornithinolytica**						
Amicacina	1	0	0	0	**0%**	1
Ampicilina	0	0	1	0	**100%**	1
Ampicilina/Sulbactam	1	0	0	0	**0%**	1
Cefepime	1	0	0	0	**0%**	1
Cefoxitina	1	0	0	0	**0%**	1
Ceftazidima	1	0	0	0	**0%**	1
Ceftriaxona	1	0	0	0	**0%**	1
Cefuroxima	0	0	1	0	**100%**	1
Cefuroxima axetil	0	0	1	0	**100%**	1
Ciprofloxacina	1	0	0	0	**0%**	1
Ertapenem	1	0	0	0	**0%**	1
Gentamicina	1	0	0	0	**0%**	1
Imipenem	1	0	0	0	**0%**	1
Meropenem	1	0	0	0	**0%**	1
Piperacilina/Tazobactam	1	0	0	0	**0%**	1
Tigeciclina	1	0	0	0	**0%**	1
**Subtotal**	**13**	**0**	**3**	**0**	**18.75%**	**16**

Análise de Resistência Bacteriana - Teste de Sensibilidade

Microrganismo/Antibiótico	Sensível	Intermediário	Resistente	Não Testado	% Resistentes	Total
**Salmonella enterica**						
Amicacina	0	0	1	0	**100%**	1
Amoxacilina/Ácido Clavulânico	2	0	0	0	**0%**	2
Ampicilina	4	0	1	0	**20%**	5
Ampicilina/Sulbactam	2	0	1	0	**33.33%**	3
Cefalotina	1	0	0	0	**0%**	1
Cefazolina	0	0	1	0	**100%**	1
Cefepime	4	0	1	0	**20%**	5
Ceftazidima	2	0	1	0	**33.33%**	3
Ceftriaxona	4	0	1	0	**20%**	5
Cefuroxima	0	0	1	0	**100%**	1
Cefuroxima axetil	0	0	1	0	**100%**	1
Ciprofloxacina	4	0	1	0	**20%**	5
Ertapenem	4	0	1	0	**20%**	5
Gentamicina	0	0	1	0	**100%**	1
Imipenem	2	0	1	0	**33.33%**	3
Levofloxacina	0	0	1	0	**100%**	1
Meropenem	4	0	1	0	**20%**	5
Nitrofurantoína	2	0	0	0	**0%**	2
Norfloxacina	2	0	0	0	**0%**	2
Piperacilina/Tazobactam	5	0	0	0	**0%**	5
Tigeciclina	1	0	1	0	**50%**	2
Trimetoprim/Sulfametoxazol	2	0	1	0	**33.33%**	3
**Subtotal**	**45**	**0**	**17**	**0**	**27.42%**	**62**
**Salmonella enterica subsp. enterica**						
Amoxacilina/Ácido Clavulânico	0	1	0	0	**0%**	1
Ampicilina	0	0	1	0	**100%**	1
Cefepime	1	0	0	0	**0%**	1
Ceftazidima	1	0	0	0	**0%**	1
Ceftriaxona	1	0	0	0	**0%**	1
Ciprofloxacina	0	0	1	0	**100%**	1
Ertapenem	1	0	0	0	**0%**	1
Imipenem	1	0	0	0	**0%**	1
Levofloxacina	0	0	1	0	**100%**	1
Meropenem	1	0	0	0	**0%**	1
Nitrofurantoína	1	0	0	0	**0%**	1
Piperacilina/Tazobactam	1	0	0	0	**0%**	1
Trimetoprim/Sulfametoxazol	1	0	0	0	**0%**	1
**Subtotal**	**9**	**1**	**3**	**0**	**23.08%**	**13**
**Salmonella Enteritidis**						
Amicacina	0	0	5	0	**100%**	5
Ampicilina	1	0	5	0	**83.33%**	6
Ampicilina/Sulbactam	1	0	5	0	**83.33%**	6
Cefepime	2	0	4	0	**66.67%**	6
Cefoxitina	1	0	6	0	**85.71%**	7
Ceftazidima	2	0	4	0	**66.67%**	6
Ceftriaxona	1	0	7	0	**87.5%**	8
Cefuroxima	1	0	5	0	**83.33%**	6
Cefuroxima axetil	1	0	5	0	**83.33%**	6
Ciprofloxacina	5	2	0	0	**0%**	7
Cloranfenicol	1	0	0	0	**0%**	1
Ertapenem	2	0	4	0	**66.67%**	6

Análise de Resistência Bacteriana - Teste de Sensibilidade

Microrganismo/Antibiótico	Sensível	Intermediário	Resistente	Não Testado	% Resistentes	Total
Gentamicina	1	0	6	0	**85.71%**	7
Imipenem	1	4	2	0	**28.57%**	7
Meropenem	3	2	2	0	**28.57%**	7
Piperacilina/Tazobactam	2	0	4	0	**66.67%**	6
Sulfametoxazol/trimetoprim	1	0	1	0	**50%**	2
Tigeciclina	4	2	0	0	**0%**	6
**Subtotal**	**30**	**10**	**65**	**0**	**61.9%**	**105**
**Salmonella sp.**						
Acido Nalidíxico	3	0	0	0	**0%**	3
Amicacina	3	0	1	0	**25%**	4
Amoxacilina/Ácido Clavulânico	3	0	1	0	**25%**	4
Ampicilina	10	0	3	0	**23.08%**	13
Ampicilina/Sulbactam	5	0	1	0	**16.67%**	6
Aztreonam	0	0	1	0	**100%**	1
Cefazolina	0	0	1	0	**100%**	1
Cefepime	7	1	2	0	**20%**	10
Cefotaxima	0	0	1	0	**100%**	1
Cefoxitina	7	0	1	0	**12.5%**	8
Ceftaroline	0	0	1	0	**100%**	1
Ceftazidima	14	0	1	0	**6.67%**	15
Ceftriaxona	8	0	4	0	**33.33%**	12
Cefuroxima	3	0	3	0	**50%**	6
Cefuroxima axetil	3	0	1	0	**25%**	4
Ciprofloxacina	12	0	2	0	**14.29%**	14
Cloranfenicol	6	0	0	0	**0%**	6
Colistin	1	0	0	0	**0%**	1
Ertapenem	9	0	0	0	**0%**	9
Estreptomicina	4	0	1	0	**20%**	5
Gentamicina	7	0	3	0	**30%**	10
Imipenem	13	0	0	0	**0%**	13
Levofloxacina	0	0	2	0	**100%**	2
Meropenem	11	0	0	0	**0%**	11
Nitrofurantoína	2	0	1	0	**33.33%**	3
Piperacilina/Tazobactam	9	0	0	0	**0%**	9
Sulfametoxazol/trimetoprim	4	0	2	0	**33.33%**	6
Tetraciclina	2	0	1	0	**33.33%**	3
Tigeciclina	5	0	0	0	**0%**	5
Trimetoprim/Sulfametoxazol	2	0	2	0	**50%**	4
**Subtotal**	**153**	**1**	**36**	**0**	**18.95%**	**190**
**Salmonella Typhimurium**						
Acido Nalidíxico	1	0	0	0	**0%**	1
Ampicilina	1	0	0	0	**0%**	1
Cefoxitina	1	0	0	0	**0%**	1
Ceftazidima	1	0	0	0	**0%**	1
Ciprofloxacina	1	0	0	0	**0%**	1
Cloranfenicol	1	0	0	0	**0%**	1
Estreptomicina	1	0	0	0	**0%**	1
Gentamicina	1	0	0	0	**0%**	1
Nitrofurantoína	0	0	1	0	**100%**	1
Sulfametoxazol/trimetoprim	1	0	0	0	**0%**	1
**Subtotal**	**9**	**0**	**1**	**0**	**10%**	**10**
**Serratia ficaria**						

Análise de Resistência Bacteriana - Teste de Sensibilidade

Microrganismo/Antibiótico	Sensível	Intermediário	Resistente	Não Testado	% Resistentes	Total
Amicacina	0	0	2	0	**100%**	2
Cefazolina	0	0	2	0	**100%**	2
Cefepime	0	2	0	0	**0%**	2
Cefoxitina	2	0	0	0	**0%**	2
Ceftriaxona	2	0	0	0	**0%**	2
Ciprofloxacina	2	0	0	0	**0%**	2
Ertapenem	0	0	2	0	**100%**	2
Gentamicina	0	0	2	0	**100%**	2
Imipenem	2	0	0	0	**0%**	2
Levofloxacina	2	0	0	0	**0%**	2
Meropenem	0	2	0	0	**0%**	2
Piperacilina/Tazobactam	2	0	0	0	**0%**	2
Trimetoprim/Sulfametoxazol	2	0	0	0	**0%**	2
**Subtotal**	**14**	**4**	**8**	**0**	**30.77%**	**26**
**Serratia liquefaciens**						
Amicacina	3	0	1	0	**25%**	4
Ampicilina	0	0	4	0	**100%**	4
Ampicilina/Sulbactam	0	0	4	0	**100%**	4
Cefepime	3	0	1	0	**25%**	4
Cefoxitina	0	1	3	0	**75%**	4
Ceftazidima	2	0	2	0	**50%**	4
Ceftriaxona	1	0	3	0	**75%**	4
Cefuroxima	0	0	4	0	**100%**	4
Cefuroxima axetil	0	0	4	0	**100%**	4
Ciprofloxacina	2	1	1	0	**25%**	4
Colistin	0	0	1	0	**100%**	1
Ertapenem	3	0	1	0	**25%**	4
Gentamicina	2	0	2	0	**50%**	4
Imipenem	2	1	1	0	**25%**	4
Meropenem	3	0	1	0	**25%**	4
Piperacilina/Tazobactam	2	1	1	0	**25%**	4
Tigeciclina	3	0	1	0	**25%**	4
**Subtotal**	**26**	**4**	**35**	**0**	**53.85%**	**65**
**Serratia marcescens**						
Acido Nalidíxico	10	0	3	0	**23.08%**	13
Amicacina	202	47	53	0	**17.55%**	302
Amoxacilina/Ácido Clavulânico	0	0	45	0	**100%**	45
Amoxicilina	0	0	2	0	**100%**	2
Ampicilina	4	2	283	0	**97.92%**	289
Ampicilina/Sulbactam	5	0	223	0	**97.81%**	228
Aztreonam	2	0	0	0	**0%**	2
Cefalotina	0	0	22	0	**100%**	22
Cefazolina	0	0	49	0	**100%**	49
Cefepime	113	17	173	0	**57.1%**	303
Cefotaxima	0	0	2	0	**100%**	2
Cefoxitina	8	3	241	0	**95.63%**	252
Ceftazidima	55	5	92	0	**60.53%**	152
Ceftriaxona	94	3	198	0	**67.12%**	295
Cefuroxima	2	0	179	0	**98.9%**	181
Cefuroxima axetil	2	0	144	0	**98.63%**	146
Ciprofloxacina	137	62	100	0	**33.44%**	299
Cloranfenicol	1	0	0	0	**0%**	1

Análise de Resistência Bacteriana - Teste de Sensibilidade

Microrganismo/Antibiótico	Sensível	Intermediário	Resistente	Não Testado	% Resistentes	Total
Colistin	3	0	44	0	**93.62%**	47
Ertapenem	202	3	92	0	**30.98%**	297
Gentamicina	133	6	159	0	**53.36%**	298
Imipenem	48	52	50	0	**33.33%**	150
Levofloxacina	76	17	20	0	**17.7%**	113
Lomefloxacina	1	0	0	0	**0%**	1
Meropenem	217	6	80	0	**26.4%**	303
Nitrofurantoína	0	0	21	0	**100%**	21
Norfloxacina	12	0	6	0	**33.33%**	18
Piperacilina/Tazobactam	68	37	31	0	**22.79%**	136
Polimixina "B"	1	0	0	0	**0%**	1
Sulfametoxazol/trimetoprim	10	0	6	0	**37.5%**	16
Tetraciclina	4	0	4	0	**50%**	8
Tigeciclina	96	106	35	0	**14.77%**	237
Trimetoprim/Sulfametoxazol	73	0	71	0	**49.31%**	144
**Subtotal**	**1579**	**366**	**2428**	**0**	**55.52%**	**4373**
**Serratia plymuthica**						
Amicacina	7	0	1	0	**12.5%**	8
Amoxacilina/Ácido Clavulânico	0	0	4	0	**100%**	4
Ampicilina	1	0	6	0	**85.71%**	7
Ampicilina/Sulbactam	1	0	2	0	**66.67%**	3
Cefazolina	0	0	4	0	**100%**	4
Cefepime	1	1	7	0	**77.78%**	9
Cefoxitina	1	1	7	0	**77.78%**	9
Ceftazidima	0	0	1	0	**100%**	1
Ceftriaxona	1	1	7	0	**77.78%**	9
Cefuroxima	1	0	4	0	**80%**	5
Cefuroxima axetil	1	0	0	0	**0%**	1
Ciprofloxacina	1	0	7	0	**87.5%**	8
Ertapenem	7	0	2	0	**22.22%**	9
Gentamicina	3	1	4	0	**50%**	8
Imipenem	3	6	0	0	**0%**	9
Levofloxacina	4	0	0	0	**0%**	4
Meropenem	8	0	1	0	**11.11%**	9
Nitrofurantoína	0	0	1	0	**100%**	1
Piperacilina/Tazobactam	3	5	1	0	**11.11%**	9
Tigeciclina	2	2	0	0	**0%**	4
Trimetoprim/Sulfametoxazol	2	0	6	0	**75%**	8
**Subtotal**	**47**	**17**	**65**	**0**	**50.39%**	**129**
**Serratia rubidaea**						
Amicacina	3	0	1	0	**25%**	4
Ampicilina	0	2	2	0	**50%**	4
Ampicilina/Sulbactam	2	0	2	0	**50%**	4
Cefepime	2	0	2	0	**50%**	4
Cefoxitina	0	0	4	0	**100%**	4
Ceftazidima	2	0	1	0	**33.33%**	3
Ceftriaxona	2	0	2	0	**50%**	4
Cefuroxima	0	0	3	0	**100%**	3
Cefuroxima axetil	0	0	3	0	**100%**	3
Ciprofloxacina	3	0	1	0	**25%**	4
Ertapenem	2	0	2	0	**50%**	4
Gentamicina	3	0	1	0	**25%**	4

Análise de Resistência Bacteriana - Teste de Sensibilidade

Microrganismo/Antibiótico	Sensível	Intermediário	Resistente	Não Testado	% Resistentes	Total
Imipenem	2	0	2	0	**50%**	4
Levofloxacina	1	0	0	0	**0%**	1
Meropenem	3	0	1	0	**25%**	4
Piperacilina/Tazobactam	3	0	1	0	**25%**	4
Tigeciclina	2	1	1	0	**25%**	4
Trimetoprim/Sulfametoxazol	1	0	0	0	**0%**	1
**Subtotal**	**31**	**3**	**29**	**0**	**46.03%**	**63**
**Serratia sp.**						
Acido Nalidíxico	0	0	1	0	**100%**	1
Amicacina	4	0	7	0	**63.64%**	11
Amoxacilina/Ácido Clavulânico	0	0	4	0	**100%**	4
Amoxicilina	0	0	1	0	**100%**	1
Ampicilina	0	0	11	0	**100%**	11
Ampicilina/Sulbactam	2	0	2	0	**50%**	4
Azitromicina	1	0	0	0	**0%**	1
Aztreonam	1	0	1	0	**50%**	2
Cefalotina	1	0	2	0	**66.67%**	3
Cefepime	4	0	8	0	**66.67%**	12
Cefotaxima	0	0	4	0	**100%**	4
Cefoxitina	3	0	5	0	**62.5%**	8
Ceftazidima	3	0	6	0	**66.67%**	9
Ceftriaxona	5	0	7	0	**58.33%**	12
Cefuroxima	2	0	2	0	**50%**	4
Cefuroxima axetil	0	0	1	0	**100%**	1
Ciprofloxacina	4	1	10	0	**66.67%**	15
Cloranfenicol	3	0	0	0	**0%**	3
Doxiciclina	1	0	0	0	**0%**	1
Ertapenem	7	0	3	0	**30%**	10
Gentamicina	2	0	2	0	**50%**	4
Imipenem	15	1	4	0	**20%**	20
Levofloxacina	3	0	4	0	**57.14%**	7
Meropenem	15	0	4	0	**21.05%**	19
Nitrofurantoína	0	0	2	0	**100%**	2
Norfloxacina	0	0	1	0	**100%**	1
Piperacilina/Tazobactam	3	0	2	0	**40%**	5
Sulfametoxazol/trimetoprim	3	0	7	0	**70%**	10
Tetraciclina	3	0	3	0	**50%**	6
Tigeciclina	1	0	1	0	**50%**	2
Tobramicina	1	0	1	0	**50%**	2
Trimetoprim/Sulfametoxazol	0	0	1	0	**100%**	1
**Subtotal**	**87**	**2**	**107**	**0**	**54.59%**	**196**
**Shewanella putrefaciens**						
Amicacina	1	0	0	0	**0%**	1
Cefepime	1	0	0	0	**0%**	1
Ceftazidima	0	0	1	0	**100%**	1
Ciprofloxacina	1	0	0	0	**0%**	1
Gentamicina	1	0	0	0	**0%**	1
Imipenem	1	0	0	0	**0%**	1
Levofloxacina	1	0	0	0	**0%**	1
Piperacilina/Tazobactam	1	0	0	0	**0%**	1
Trimetoprim/Sulfametoxazol	0	0	1	0	**100%**	1
**Subtotal**	**7**	**0**	**2**	**0**	**22.22%**	**9**

Análise de Resistência Bacteriana - Teste de Sensibilidade

Microrganismo/Antibiótico	Sensível	Intermediário	Resistente	Não Testado	% Resistentes	Total
**Shigella boydii**						
Amoxacilina/Ácido Clavulânico	1	0	0	0	**0%**	1
Ampicilina	2	0	0	0	**0%**	2
Ampicilina/Sulbactam	1	0	0	0	**0%**	1
Cefepime	2	0	0	0	**0%**	2
Ceftriaxona	2	0	0	0	**0%**	2
Ciprofloxacina	2	0	0	0	**0%**	2
Ertapenem	2	0	0	0	**0%**	2
Imipenem	1	1	0	0	**0%**	2
Levofloxacina	2	0	0	0	**0%**	2
Meropenem	2	0	0	0	**0%**	2
Nitrofurantoína	1	0	0	0	**0%**	1
Piperacilina/Tazobactam	2	0	0	0	**0%**	2
Tigeciclina	1	0	0	0	**0%**	1
Trimetoprim/Sulfametoxazol	2	0	0	0	**0%**	2
**Subtotal**	**23**	**1**	**0**	**0**	**0%**	**24**
**Shigella flexneri**						
Ampicilina	0	0	2	0	**100%**	2
Ampicilina/Sulbactam	0	1	1	0	**50%**	2
Cefazolina	0	0	1	0	**100%**	1
Cefepime	2	0	0	0	**0%**	2
Ceftriaxona	2	0	0	0	**0%**	2
Ciprofloxacina	0	0	2	0	**100%**	2
Ertapenem	2	0	0	0	**0%**	2
Imipenem	1	1	0	0	**0%**	2
Levofloxacina	0	0	2	0	**100%**	2
Meropenem	2	0	0	0	**0%**	2
Piperacilina/Tazobactam	2	0	0	0	**0%**	2
Tigeciclina	2	0	0	0	**0%**	2
Trimetoprim/Sulfametoxazol	1	0	1	0	**50%**	2
**Subtotal**	**14**	**2**	**9**	**0**	**36%**	**25**
**Shigella sonnei**						
Amicacina	0	0	1	0	**100%**	1
Ampicilina	0	0	1	0	**100%**	1
Ampicilina/Sulbactam	0	0	1	0	**100%**	1
Cefepime	1	0	0	0	**0%**	1
Cefoxitina	0	0	1	0	**100%**	1
Ceftazidima	1	0	0	0	**0%**	1
Ceftriaxona	1	0	0	0	**0%**	1
Cefuroxima	0	0	1	0	**100%**	1
Ciprofloxacina	1	0	0	0	**0%**	1
Ertapenem	1	0	0	0	**0%**	1
Gentamicina	0	0	1	0	**100%**	1
Imipenem	1	0	0	0	**0%**	1
Meropenem	1	0	0	0	**0%**	1
**Subtotal**	**7**	**0**	**6**	**0**	**46.15%**	**13**
**Shigella sp.**						
Amoxacilina/Ácido Clavulânico	1	0	0	0	**0%**	1
Ampicilina/Sulbactam	1	0	0	0	**0%**	1
Cefoxitina	1	0	0	0	**0%**	1
Cloranfenicol	1	0	0	0	**0%**	1
Gentamicina	1	0	0	0	**0%**	1

Análise de Resistência Bacteriana - Teste de Sensibilidade

Microrganismo/Antibiótico	Sensível	Intermediário	Resistente	Não Testado	% Resistentes	Total
Imipenem	1	0	0	0	**0%**	1
Sulfametoxazol/trimetoprim	1	0	0	0	**0%**	1
**Subtotal**	**7**	**0**	**0**	**0**	**0%**	**7**
**Sphingobacterium sp.**						
Amicacina	0	0	2	0	**100%**	2
Cefepime	1	1	0	0	**0%**	2
Ceftazidima	2	0	0	0	**0%**	2
Ciprofloxacina	1	0	1	0	**50%**	2
Gentamicina	0	0	2	0	**100%**	2
Imipenem	0	0	2	0	**100%**	2
Levofloxacina	2	0	0	0	**0%**	2
Piperacilina/Tazobactam	2	0	0	0	**0%**	2
Trimetoprim/Sulfametoxazol	2	0	0	0	**0%**	2
**Subtotal**	**10**	**1**	**7**	**0**	**38.89%**	**18**
**Sphingobacterium spiritivorum**						
Amicacina	0	0	1	0	**100%**	1
Ampicilina	0	0	1	0	**100%**	1
Ampicilina/Sulbactam	0	0	1	0	**100%**	1
Cefepime	0	0	1	0	**100%**	1
Cefoxitina	0	1	0	0	**0%**	1
Ceftazidima	0	0	1	0	**100%**	1
Ceftriaxona	0	0	1	0	**100%**	1
Cefuroxima	0	0	1	0	**100%**	1
Cefuroxima axetil	0	0	1	0	**100%**	1
Ciprofloxacina	1	0	0	0	**0%**	1
Gentamicina	0	0	1	0	**100%**	1
Imipenem	0	0	1	0	**100%**	1
Meropenem	0	0	1	0	**100%**	1
Piperacilina/Tazobactam	0	0	1	0	**100%**	1
Tigeciclina	0	1	0	0	**0%**	1
**Subtotal**	**1**	**2**	**12**	**0**	**80%**	**15**
**Sphingomonas paucimobilis**						
Acido Nalidíxico	0	0	1	0	**100%**	1
Amicacina	16	0	7	0	**30.43%**	23
Amoxacilina/Ácido Clavulânico	2	0	0	0	**0%**	2
Ampicilina	10	2	7	0	**36.84%**	19
Ampicilina/Sulbactam	14	0	3	0	**17.65%**	17
Cefepime	13	1	8	0	**36.36%**	22
Cefoxitina	11	1	5	0	**29.41%**	17
Ceftazidima	10	3	8	0	**38.1%**	21
Ceftriaxona	13	3	3	0	**15.79%**	19
Cefuroxima	13	2	4	0	**21.05%**	19
Cefuroxima axetil	13	2	4	0	**21.05%**	19
Ciprofloxacina	17	1	5	0	**21.74%**	23
Colistin	3	0	7	0	**70%**	10
Gentamicina	16	2	5	0	**21.74%**	23
Imipenem	18	0	3	0	**14.29%**	21
Levofloxacina	4	0	0	0	**0%**	4
Meropenem	17	0	2	0	**10.53%**	19
Nitrofurantoína	0	0	2	0	**100%**	2
Norfloxacina	1	0	1	0	**50%**	2
Piperacilina/Tazobactam	11	5	2	0	**11.11%**	18

Análise de Resistência Bacteriana - Teste de Sensibilidade

Microrganismo/Antibiótico	Sensível	Intermediário	Resistente	Não Testado	% Resistentes	Total
Tigeciclina	17	0	0	0	**0%**	17
Trimetoprim/Sulfametoxazol	5	0	1	0	**16.67%**	6
**Subtotal**	**224**	**22**	**78**	**0**	**24.07%**	**324**
**Staphylococcus arlettae**						
Oxacilina	0	0	1	0	**100%**	1
**Subtotal**	**0**	**0**	**1**	**0**	**100%**	**1**
**Staphylococcus aureus**						
Amicacina	0	0	2	0	**100%**	2
Ampicilina	1	0	34	0	**97.14%**	35
Ampicilina/Sulbactam	0	0	1	0	**100%**	1
Azitromicina	10	0	13	0	**56.52%**	23
Aztreonam	0	0	2	0	**100%**	2
Cefazolina	5	0	0	0	**0%**	5
Cefepime	0	0	1	0	**100%**	1
Cefoxitina	17	0	8	0	**32%**	25
Ceftaroline	819	93	46	0	**4.8%**	958
Ceftazidima	2	0	1	0	**33.33%**	3
Ceftriaxona	5	0	3	0	**37.5%**	8
Ciprofloxacina	160	0	46	0	**22.33%**	206
Claritromicina	6	0	7	0	**53.85%**	13
Clindamicina	727	3	352	0	**32.53%**	1082
Cloranfenicol	26	0	1	0	**3.7%**	27
Dapsona	13	0	0	0	**0%**	13
Daptomicina	988	0	2	0	**0.2%**	990
Eritromicina	512	9	668	0	**56.18%**	1189
Ertapenem	0	0	2	0	**100%**	2
Gentamicina	392	1	31	0	**7.31%**	424
Imipenem	0	0	1	0	**100%**	1
Levofloxacina	148	2	67	0	**30.88%**	217
Linezolida	1177	0	16	0	**1.34%**	1193
Meropenem	2	0	1	0	**33.33%**	3
Minociclina	731	10	16	0	**2.11%**	757
Moxifloxacina	86	8	24	0	**20.34%**	118
Nitrofurantoína	184	0	1	0	**0.54%**	185
Norfloxacina	66	2	25	0	**26.88%**	93
Ofloxacina	1	0	2	0	**66.67%**	3
Oxacilina	805	0	603	0	**42.83%**	1408
Penicilina	80	0	991	0	**92.53%**	1071
Piperacilina	0	0	2	0	**100%**	2
Piperacilina/Tazobactam	0	0	1	0	**100%**	1
Rifampicina	1008	19	172	0	**14.35%**	1199
Sulfametoxazol/trimetoprim	63	0	7	0	**10%**	70
Sulfazotrim	2	0	0	0	**0%**	2
Teicoplanina	383	0	4	0	**1.03%**	387
Tetraciclina	37	0	12	0	**24.49%**	49
Tigeciclina	1085	0	0	0	**0%**	1085
Trimetoprim/Sulfametoxazol	1005	0	118	0	**10.51%**	1123
Vancomicina	1108	7	31	0	**2.71%**	1146
**Subtotal**	**11654**	**154**	**3314**	**0**	**21.92%**	**15122**
**Staphylococcus auricularis**						
Ciprofloxacina	0	0	1	0	**100%**	1
Clindamicina	1	0	1	0	**50%**	2

Análise de Resistência Bacteriana - Teste de Sensibilidade

Microrganismo/Antibiótico	Sensível	Intermediário	Resistente	Não Testado	% Resistentes	Total
Daptomicina	1	0	0	0	**0%**	1
Eritromicina	1	0	1	0	**50%**	2
Gentamicina	1	0	0	0	**0%**	1
Linezolida	2	0	0	0	**0%**	2
Minociclina	1	0	0	0	**0%**	1
Moxifloxacina	0	0	1	0	**100%**	1
Oxacilina	1	0	1	0	**50%**	2
Penicilina	0	0	1	0	**100%**	1
Rifampicina	2	0	0	0	**0%**	2
Teicoplanina	1	0	0	0	**0%**	1
Tigeciclina	1	0	0	0	**0%**	1
Trimetoprim/Sulfametoxazol	2	0	0	0	**0%**	2
Vancomicina	2	0	0	0	**0%**	2
**Subtotal**	**16**	**0**	**6**	**0**	**27.27%**	**22**
**Staphylococcus capitis**						
Ampicilina	0	0	6	0	**100%**	6
Ceftaroline	7	0	0	0	**0%**	7
Ciprofloxacina	2	3	20	0	**80%**	25
Clindamicina	24	2	128	0	**83.12%**	154
Daptomicina	150	0	0	0	**0%**	150
Eritromicina	19	1	157	0	**88.7%**	177
Gentamicina	19	9	14	0	**33.33%**	42
Levofloxacina	8	0	9	0	**52.94%**	17
Linezolida	143	0	34	0	**19.21%**	177
Minociclina	130	3	2	0	**1.48%**	135
Moxifloxacina	0	9	2	0	**18.18%**	11
Nitrofurantoína	17	0	0	0	**0%**	17
Norfloxacina	0	0	10	0	**100%**	10
Oxacilina	24	0	152	0	**86.36%**	176
Penicilina	3	0	45	0	**93.75%**	48
Rifampicina	145	0	30	0	**17.14%**	175
Sulfametoxazol/trimetoprim	1	0	1	0	**50%**	2
Teicoplanina	39	1	0	0	**0%**	40
Tetraciclina	7	0	0	0	**0%**	7
Tigeciclina	34	0	0	0	**0%**	34
Trimetoprim/Sulfametoxazol	136	0	35	0	**20.47%**	171
Vancomicina	163	0	7	0	**4.12%**	170
**Subtotal**	**1071**	**28**	**652**	**0**	**37.24%**	**1751**
**Staphylococcus caprae**						
Azitromicina	0	0	1	0	**100%**	1
Ciprofloxacina	0	0	1	0	**100%**	1
Clindamicina	5	0	9	0	**64.29%**	14
Cloranfenicol	0	0	1	0	**100%**	1
Daptomicina	18	0	0	0	**0%**	18
Eritromicina	4	0	14	0	**77.78%**	18
Linezolida	13	0	6	0	**31.58%**	19
Minociclina	18	0	0	0	**0%**	18
Oxacilina	4	0	14	0	**77.78%**	18
Penicilina	0	0	1	0	**100%**	1
Rifampicina	17	0	2	0	**10.53%**	19
Trimetoprim/Sulfametoxazol	13	0	5	0	**27.78%**	18
Vancomicina	17	0	1	0	**5.56%**	18

Análise de Resistência Bacteriana - Teste de Sensibilidade

Microrganismo/Antibiótico	Sensível	Intermediário	Resistente	Não Testado	% Resistentes	Total
**Subtotal**	**109**	**0**	**55**	**0**	**33.54%**	**164**
**Staphylococcus carnosus**						
Clindamicina	2	0	3	0	**60%**	5
Daptomicina	2	0	0	0	**0%**	2
Eritromicina	2	0	3	0	**60%**	5
Linezolida	3	0	2	0	**40%**	5
Minociclina	5	0	0	0	**0%**	5
Oxacilina	1	0	4	0	**80%**	5
Rifampicina	2	0	3	0	**60%**	5
Trimetoprim/Sulfametoxazol	3	0	2	0	**40%**	5
Vancomicina	2	1	1	0	**25%**	4
**Subtotal**	**22**	**1**	**18**	**0**	**43.9%**	**41**
**Staphylococcus chromogenes**						
Ciprofloxacina	1	0	0	0	**0%**	1
Clindamicina	1	0	0	0	**0%**	1
Daptomicina	1	0	0	0	**0%**	1
Eritromicina	1	0	0	0	**0%**	1
Gentamicina	1	0	0	0	**0%**	1
Linezolida	1	0	0	0	**0%**	1
Oxacilina	1	0	0	0	**0%**	1
Penicilina	1	0	0	0	**0%**	1
Rifampicina	1	0	0	0	**0%**	1
Sulfametoxazol/trimetoprim	1	0	0	0	**0%**	1
Teicoplanina	1	0	0	0	**0%**	1
Tetraciclina	1	0	0	0	**0%**	1
Vancomicina	1	0	0	0	**0%**	1
**Subtotal**	**13**	**0**	**0**	**0**	**0%**	**13**
**Staphylococcus coagulase-negativa**						
Ampicilina	1	0	1	0	**50%**	2
Azitromicina	6	0	9	0	**60%**	15
Aztreonam	1	0	2	0	**66.67%**	3
Cefoxitina	14	0	8	0	**36.36%**	22
Ceftaroline	2	0	0	0	**0%**	2
Ceftriaxona	9	0	3	0	**25%**	12
Ciprofloxacina	18	1	16	0	**45.71%**	35
Claritromicina	1	0	3	0	**75%**	4
Clindamicina	31	0	15	0	**32.61%**	46
Cloranfenicol	18	0	7	0	**28%**	25
Daptomicina	6	1	0	0	**0%**	7
Eritromicina	15	2	31	0	**64.58%**	48
Gentamicina	25	0	9	0	**26.47%**	34
Levofloxacina	8	0	0	0	**0%**	8
Linezolida	34	0	3	0	**8.11%**	37
Minociclina	10	0	0	0	**0%**	10
Moxifloxacina	4	0	2	0	**33.33%**	6
Nitrofurantoína	3	0	0	0	**0%**	3
Norfloxacina	3	0	1	0	**25%**	4
Oxacilina	21	3	20	0	**45.45%**	44
Penicilina	13	0	32	0	**71.11%**	45
Rifampicina	37	0	4	0	**9.76%**	41
Sulfametoxazol/trimetoprim	12	0	15	0	**55.56%**	27
Sulfazotrim	1	0	0	0	**0%**	1

Análise de Resistência Bacteriana - Teste de Sensibilidade

Microrganismo/Antibiótico	Sensível	Intermediário	Resistente	Não Testado	% Resistentes	Total
Teicoplanina	8	0	0	0	**0%**	8
Tetraciclina	12	0	4	0	**25%**	16
Tigeciclina	7	0	0	0	**0%**	7
Trimetoprim/Sulfametoxazol	6	0	5	0	**45.45%**	11
Vancomicina	17	0	0	0	**0%**	17
**Subtotal**	**343**	**7**	**190**	**0**	**35.19%**	**540**
**Staphylococcus cohnii**						
Ceftaroline	2	0	0	0	**0%**	2
Ciprofloxacina	1	0	0	0	**0%**	1
Clindamicina	7	1	2	0	**20%**	10
Daptomicina	13	0	1	0	**7.14%**	14
Eritromicina	4	0	10	0	**71.43%**	14
Gentamicina	4	0	1	0	**20%**	5
Levofloxacina	3	0	1	0	**25%**	4
Linezolida	10	0	4	0	**28.57%**	14
Minociclina	8	1	0	0	**0%**	9
Nitrofurantoína	3	0	1	0	**25%**	4
Oxacilina	4	0	9	0	**69.23%**	13
Penicilina	2	0	2	0	**50%**	4
Rifampicina	11	0	1	0	**8.33%**	12
Teicoplanina	4	0	0	0	**0%**	4
Tetraciclina	1	0	0	0	**0%**	1
Tigeciclina	3	0	1	0	**25%**	4
Trimetoprim/Sulfametoxazol	11	0	2	0	**15.38%**	13
Vancomicina	10	0	3	0	**23.08%**	13
**Subtotal**	**101**	**2**	**38**	**0**	**26.95%**	**141**
**Staphylococcus cohnii subsp. cohnii**						
Clindamicina	4	0	5	0	**55.56%**	9
Daptomicina	9	0	0	0	**0%**	9
Eritromicina	4	0	4	0	**50%**	8
Ertapenem	0	0	1	0	**100%**	1
Linezolida	5	0	4	0	**44.44%**	9
Minociclina	9	0	0	0	**0%**	9
Oxacilina	3	0	6	0	**66.67%**	9
Rifampicina	8	0	1	0	**11.11%**	9
Trimetoprim/Sulfametoxazol	8	0	1	0	**11.11%**	9
Vancomicina	9	0	0	0	**0%**	9
**Subtotal**	**59**	**0**	**22**	**0**	**27.16%**	**81**
**Staphylococcus cohnii subsp. urealyticus**						
Ceftaroline	1	0	0	0	**0%**	1
Clindamicina	0	0	7	0	**100%**	7
Daptomicina	7	0	0	0	**0%**	7
Eritromicina	0	0	7	0	**100%**	7
Gentamicina	1	0	0	0	**0%**	1
Levofloxacina	1	0	0	0	**0%**	1
Linezolida	6	0	1	0	**14.29%**	7
Minociclina	5	0	1	0	**16.67%**	6
Oxacilina	1	0	6	0	**85.71%**	7
Penicilina	0	0	1	0	**100%**	1
Rifampicina	6	0	1	0	**14.29%**	7
Teicoplanina	1	0	0	0	**0%**	1
Tigeciclina	1	0	0	0	**0%**	1

Análise de Resistência Bacteriana - Teste de Sensibilidade

Microrganismo/Antibiótico	Sensível	Intermediário	Resistente	Não Testado	% Resistentes	Total
Trimetoprim/Sulfametoxazol	2	0	5	0	**71.43%**	7
Vancomicina	6	0	1	0	**14.29%**	7
**Subtotal**	**38**	**0**	**30**	**0**	**44.12%**	**68**
**Staphylococcus epidermidis**						
Amicacina	1	0	1	0	**50%**	2
Ampicilina	0	0	28	0	**100%**	28
Azitromicina	1	0	5	0	**83.33%**	6
Cefepime	1	0	0	0	**0%**	1
Cefoxitina	2	0	3	0	**60%**	5
Ceftaroline	91	0	0	0	**0%**	91
Ciprofloxacina	46	7	88	0	**62.41%**	141
Claritromicina	1	0	1	0	**50%**	2
Clinafloxacino	0	0	1	0	**100%**	1
Clindamicina	360	5	679	0	**65.04%**	1044
Cloranfenicol	6	0	1	0	**14.29%**	7
Dapsona	3	0	0	0	**0%**	3
Daptomicina	942	1	4	0	**0.42%**	947
Eritromicina	144	12	936	0	**85.71%**	1092
Ertapenem	0	0	1	0	**100%**	1
Gentamicina	207	15	67	0	**23.18%**	289
Gentamicina 120 mg	1	0	0	0	**0%**	1
Levofloxacina	46	0	110	0	**70.51%**	156
Linezolida	1048	0	53	0	**4.81%**	1101
Minociclina	754	30	13	0	**1.63%**	797
Moxifloxacina	14	18	23	0	**41.82%**	55
Nitrofurantoína	158	0	1	0	**0.63%**	159
Norfloxacina	13	0	33	0	**71.74%**	46
Ofloxacina	0	0	2	0	**100%**	2
Oxacilina	181	0	917	0	**83.52%**	1098
Penicilina	13	0	961	0	**98.67%**	974
Piperacilina	0	0	1	0	**100%**	1
Piperacilina/Tazobactam	1	0	0	0	**0%**	1
Rifabutina	0	0	1	0	**100%**	1
Rifampicina	854	12	235	0	**21.34%**	1101
Sulfametoxazol/trimetoprim	10	0	8	0	**44.44%**	18
Teicoplanina	239	37	6	0	**2.13%**	282
Tetraciclina	26	0	5	0	**16.13%**	31
Tigeciclina	249	0	1	0	**0.4%**	250
Trimetoprim/Sulfametoxazol	448	0	635	0	**58.63%**	1083
Vancomicina	1018	12	35	0	**3.29%**	1065
**Subtotal**	**6878**	**149**	**4855**	**0**	**40.86%**	**11882**
**Staphylococcus equorum**						
Clindamicina	1	0	2	0	**66.67%**	3
Daptomicina	3	0	1	0	**25%**	4
Eritromicina	1	0	3	0	**75%**	4
Linezolida	4	0	0	0	**0%**	4
Minociclina	3	0	1	0	**25%**	4
Oxacilina	1	0	3	0	**75%**	4
Rifampicina	2	0	2	0	**50%**	4
Trimetoprim/Sulfametoxazol	1	0	3	0	**75%**	4
Vancomicina	3	0	1	0	**25%**	4
**Subtotal**	**19**	**0**	**16**	**0**	**45.71%**	**35**

Análise de Resistência Bacteriana - Teste de Sensibilidade

Microrganismo/Antibiótico	Sensível	Intermediário	Resistente	Não Testado	% Resistentes	Total
**Staphylococcus gallinarum**						
Ampicilina	0	0	1	0	**100%**	1
Clindamicina	1	0	3	0	**75%**	4
Daptomicina	4	0	0	0	**0%**	4
Eritromicina	1	0	3	0	**75%**	4
Linezolida	3	0	3	0	**50%**	6
Minociclina	2	1	1	0	**25%**	4
Nitrofurantoína	2	0	0	0	**0%**	2
Oxacilina	2	0	4	0	**66.67%**	6
Penicilina	0	0	1	0	**100%**	1
Rifampicina	4	0	2	0	**33.33%**	6
Trimetoprim/Sulfametoxazol	4	0	2	0	**33.33%**	6
Vancomicina	3	1	2	0	**33.33%**	6
**Subtotal**	**26**	**2**	**22**	**0**	**44%**	**50**
**Staphylococcus haemolyticus**						
Ampicilina	1	0	18	0	**94.74%**	19
Ceftaroline	35	0	3	0	**7.89%**	38
Ciprofloxacina	15	0	46	0	**75.41%**	61
Clindamicina	113	3	477	0	**80.44%**	593
Cloranfenicol	2	0	0	0	**0%**	2
Dapsona	1	0	0	0	**0%**	1
Daptomicina	607	1	2	0	**0.33%**	610
Eritromicina	36	2	572	0	**93.77%**	610
Ertapenem	0	0	3	0	**100%**	3
Gentamicina	49	9	73	0	**55.73%**	131
Levofloxacina	21	0	49	0	**70%**	70
Linezolida	645	1	12	0	**1.82%**	658
Minociclina	449	19	15	0	**3.11%**	483
Moxifloxacina	8	7	13	0	**46.43%**	28
Nitrofurantoína	115	2	0	0	**0%**	117
Norfloxacina	8	0	19	0	**70.37%**	27
Oxacilina	31	0	625	0	**95.27%**	656
Penicilina	5	0	142	0	**96.6%**	147
Rifampicina	308	2	344	0	**52.6%**	654
Sulfametoxazol/trimetoprim	6	0	9	0	**60%**	15
Teicoplanina	120	3	3	0	**2.38%**	126
Tetraciclina	9	0	2	0	**18.18%**	11
Ticarcilina	1	0	0	0	**0%**	1
Tigeciclina	107	0	0	0	**0%**	107
Trimetoprim/Sulfametoxazol	202	0	443	0	**68.68%**	645
Vancomicina	606	9	26	0	**4.06%**	641
**Subtotal**	**3500**	**58**	**2896**	**0**	**44.87%**	**6454**
**Staphylococcus hominis**						
Ampicilina	0	0	8	0	**100%**	8
Azitromicina	1	0	1	0	**50%**	2
Cefoxitina	1	0	0	0	**0%**	1
Ceftaroline	1	0	0	0	**0%**	1
Ciprofloxacina	26	1	17	0	**38.64%**	44
Clindamicina	140	1	155	0	**52.36%**	296
Cloranfenicol	2	0	0	0	**0%**	2
Dapsona	2	0	0	0	**0%**	2
Daptomicina	264	0	0	0	**0%**	264

Análise de Resistência Bacteriana - Teste de Sensibilidade

Microrganismo/Antibiótico	Sensível	Intermediário	Resistente	Não Testado	% Resistentes	Total
Eritromicina	58	4	247	0	**79.94%**	309
Gentamicina	95	4	5	0	**4.81%**	104
Levofloxacina	34	2	23	0	**38.98%**	59
Linezolida	301	0	10	0	**3.22%**	311
Minociclina	197	3	3	0	**1.48%**	203
Moxifloxacina	14	1	3	0	**16.67%**	18
Nitrofurantoína	48	2	1	0	**1.96%**	51
Norfloxacina	12	1	6	0	**31.58%**	19
Ofloxacina	1	0	0	0	**0%**	1
Oxacilina	85	0	220	0	**72.13%**	305
Penicilina	5	0	106	0	**95.5%**	111
Rifampicina	262	8	42	0	**13.46%**	312
Sulfametoxazol/trimetoprim	5	0	1	0	**16.67%**	6
Teicoplanina	94	2	3	0	**3.03%**	99
Tetraciclina	5	0	1	0	**16.67%**	6
Ticarcilina	1	0	0	0	**0%**	1
Tigeciclina	91	0	0	0	**0%**	91
Trimetoprim/Sulfametoxazol	160	0	145	0	**47.54%**	305
Vancomicina	290	2	12	0	**3.95%**	304
**Subtotal**	**2195**	**31**	**1009**	**0**	**31.19%**	**3235**
**Staphylococcus intermedius**						
Ampicilina	0	0	2	0	**100%**	2
Ceftaroline	0	0	0	0	**0%**	0
Clindamicina	10	1	6	0	**35.29%**	17
Daptomicina	20	0	0	0	**0%**	20
Eritromicina	10	0	10	0	**50%**	20
Gentamicina	2	1	0	0	**0%**	3
Levofloxacina	1	1	1	0	**33.33%**	3
Linezolida	20	0	1	0	**4.76%**	21
Minociclina	13	3	1	0	**5.88%**	17
Nitrofurantoína	2	0	1	0	**33.33%**	3
Oxacilina	16	0	5	0	**23.81%**	21
Penicilina	0	0	5	0	**100%**	5
Rifampicina	16	0	5	0	**23.81%**	21
Teicoplanina	1	0	2	0	**66.67%**	3
Tigeciclina	3	0	0	0	**0%**	3
Trimetoprim/Sulfametoxazol	10	0	11	0	**52.38%**	21
Vancomicina	19	1	1	0	**4.76%**	21
**Subtotal**	**143**	**7**	**51**	**0**	**25.37%**	**201**
**Staphylococcus kloosii**						
Clindamicina	4	0	1	0	**20%**	5
Daptomicina	4	0	0	0	**0%**	4
Eritromicina	2	0	3	0	**60%**	5
Linezolida	4	0	2	0	**33.33%**	6
Minociclina	5	0	0	0	**0%**	5
Nitrofurantoína	0	0	1	0	**100%**	1
Oxacilina	1	0	5	0	**83.33%**	6
Rifampicina	4	0	2	0	**33.33%**	6
Trimetoprim/Sulfametoxazol	4	0	2	0	**33.33%**	6
Vancomicina	4	0	2	0	**33.33%**	6
**Subtotal**	**32**	**0**	**18**	**0**	**36%**	**50**
**Staphylococcus lentus**						

Análise de Resistência Bacteriana - Teste de Sensibilidade

Microrganismo/Antibiótico	Sensível	Intermediário	Resistente	Não Testado	% Resistentes	Total
Ampicilina	0	0	2	0	**100%**	2
Ceftaroline	0	1	0	0	**0%**	1
Ciprofloxacina	3	3	3	0	**33.33%**	9
Clindamicina	10	0	19	0	**65.52%**	29
Daptomicina	23	0	0	0	**0%**	23
Eritromicina	10	0	20	0	**66.67%**	30
Gentamicina	7	1	1	0	**11.11%**	9
Linezolida	32	0	1	0	**3.03%**	33
Minociclina	18	2	1	0	**4.76%**	21
Moxifloxacina	4	4	1	0	**11.11%**	9
Nitrofurantoína	3	0	0	0	**0%**	3
Norfloxacina	2	0	6	0	**75%**	8
Oxacilina	11	0	22	0	**66.67%**	33
Penicilina	0	0	11	0	**100%**	11
Rifampicina	26	1	6	0	**18.18%**	33
Teicoplanina	7	2	0	0	**0%**	9
Tigeciclina	10	0	0	0	**0%**	10
Trimetoprim/Sulfametoxazol	20	0	12	0	**37.5%**	32
Vancomicina	28	1	4	0	**12.12%**	33
**Subtotal**	**214**	**15**	**109**	**0**	**32.25%**	**338**
**Staphylococcus lugdunensis**						
Ceftaroline	3	0	0	0	**0%**	3
Ciprofloxacina	2	1	1	0	**25%**	4
Clindamicina	13	0	10	0	**43.48%**	23
Daptomicina	24	0	0	0	**0%**	24
Eritromicina	11	0	12	0	**52.17%**	23
Gentamicina	6	0	1	0	**14.29%**	7
Levofloxacina	3	0	0	0	**0%**	3
Linezolida	28	0	0	0	**0%**	28
Minociclina	15	1	0	0	**0%**	16
Moxifloxacina	2	0	1	0	**33.33%**	3
Nitrofurantoína	7	0	0	0	**0%**	7
Norfloxacina	1	0	1	0	**50%**	2
Oxacilina	15	0	12	0	**44.44%**	27
Penicilina	0	0	7	0	**100%**	7
Rifampicina	26	1	1	0	**3.57%**	28
Teicoplanina	6	1	0	0	**0%**	7
Tigeciclina	7	0	0	0	**0%**	7
Trimetoprim/Sulfametoxazol	20	0	8	0	**28.57%**	28
Vancomicina	27	0	0	0	**0%**	27
**Subtotal**	**216**	**4**	**54**	**0**	**19.71%**	**274**
**Staphylococcus saprophyticus**						
Ampicilina	0	0	2	0	**100%**	2
Ceftaroline	10	0	0	0	**0%**	10
Ciprofloxacina	7	0	0	0	**0%**	7
Clindamicina	34	0	9	0	**20.93%**	43
Dapsona	1	0	0	0	**0%**	1
Daptomicina	52	0	0	0	**0%**	52
Eritromicina	19	0	29	0	**60.42%**	48
Gentamicina	19	0	1	0	**5%**	20
Levofloxacina	10	0	1	0	**9.09%**	11
Linezolida	57	0	1	0	**1.72%**	58

Análise de Resistência Bacteriana - Teste de Sensibilidade

Microrganismo/Antibiótico	Sensível	Intermediário	Resistente	Não Testado	% Resistentes	Total
Minociclina	27	0	1	0	**3.57%**	28
Moxifloxacina	1	0	0	0	**0%**	1
Nitrofurantoína	25	0	0	0	**0%**	25
Norfloxacina	3	0	0	0	**0%**	3
Oxacilina	20	0	36	0	**64.29%**	56
Penicilina	0	0	23	0	**100%**	23
Rifampicina	57	0	0	0	**0%**	57
Sulfametoxazol/trimetoprim	3	0	1	0	**25%**	4
Teicoplanina	17	0	0	0	**0%**	17
Tetraciclina	2	0	1	0	**33.33%**	3
Tigeciclina	13	0	0	0	**0%**	13
Trimetoprim/Sulfametoxazol	36	0	17	0	**32.08%**	53
Vancomicina	55	0	0	0	**0%**	55
**Subtotal**	**468**	**0**	**122**	**0**	**20.68%**	**590**
**Staphylococcus schleiferi**						
Ampicilina	0	0	4	0	**100%**	4
Claritromicina	0	0	1	0	**100%**	1
Clindamicina	15	0	25	0	**62.5%**	40
Daptomicina	37	0	0	0	**0%**	37
Eritromicina	8	0	33	0	**80.49%**	41
Ertapenem	0	0	1	0	**100%**	1
Gentamicina	0	1	0	0	**0%**	1
Levofloxacina	0	0	1	0	**100%**	1
Linezolida	37	0	6	0	**13.95%**	43
Minociclina	39	2	0	0	**0%**	41
Nitrofurantoína	0	1	2	0	**66.67%**	3
Oxacilina	9	0	34	0	**79.07%**	43
Penicilina	0	0	6	0	**100%**	6
Rifampicina	26	0	17	0	**39.53%**	43
Sulfametoxazol/trimetoprim	0	0	1	0	**100%**	1
Teicoplanina	0	0	1	0	**100%**	1
Trimetoprim/Sulfametoxazol	15	0	27	0	**64.29%**	42
Vancomicina	34	0	7	0	**17.07%**	41
**Subtotal**	**220**	**4**	**166**	**0**	**42.56%**	**390**
**Staphylococcus sciuri**						
Ampicilina	0	0	1	0	**100%**	1
Ceftaroline	1	0	0	0	**0%**	1
Ciprofloxacina	6	0	0	0	**0%**	6
Clindamicina	5	6	10	0	**47.62%**	21
Daptomicina	17	0	0	0	**0%**	17
Eritromicina	8	3	12	0	**52.17%**	23
Gentamicina	9	0	0	0	**0%**	9
Levofloxacina	2	0	1	0	**33.33%**	3
Linezolida	24	0	0	0	**0%**	24
Minociclina	13	1	0	0	**0%**	14
Moxifloxacina	2	2	0	0	**0%**	4
Nitrofurantoína	5	0	0	0	**0%**	5
Norfloxacina	4	0	0	0	**0%**	4
Oxacilina	4	0	20	0	**83.33%**	24
Penicilina	1	1	8	0	**80%**	10
Rifampicina	19	1	4	0	**16.67%**	24
Sulfametoxazol/trimetoprim	0	0	1	0	**100%**	1

Análise de Resistência Bacteriana - Teste de Sensibilidade

Microrganismo/Antibiótico	Sensível	Intermediário	Resistente	Não Testado	% Resistentes	Total
Teicoplanina	7	0	1	0	**12.5%**	8
Tetraciclina	0	0	1	0	**100%**	1
Tigeciclina	7	0	0	0	**0%**	7
Trimetoprim/Sulfametoxazol	14	0	9	0	**39.13%**	23
Vancomicina	23	1	0	0	**0%**	24
**Subtotal**	**171**	**15**	**68**	**0**	**26.77%**	**254**
**Staphylococcus simulans**						
Ceftaroline	1	0	0	0	**0%**	1
Clindamicina	2	0	2	0	**50%**	4
Daptomicina	4	0	0	0	**0%**	4
Eritromicina	0	0	4	0	**100%**	4
Gentamicina	1	0	0	0	**0%**	1
Levofloxacina	1	0	0	0	**0%**	1
Linezolida	3	0	1	0	**25%**	4
Minociclina	3	0	0	0	**0%**	3
Nitrofurantoína	1	0	0	0	**0%**	1
Oxacilina	2	0	2	0	**50%**	4
Penicilina	0	0	1	0	**100%**	1
Rifampicina	2	0	2	0	**50%**	4
Teicoplanina	1	0	0	0	**0%**	1
Tigeciclina	1	0	0	0	**0%**	1
Trimetoprim/Sulfametoxazol	3	0	1	0	**25%**	4
Vancomicina	3	0	1	0	**25%**	4
**Subtotal**	**28**	**0**	**14**	**0**	**33.33%**	**42**
**Staphylococcus vitulinus**						
Clindamicina	1	0	1	0	**50%**	2
Daptomicina	2	0	0	0	**0%**	2
Eritromicina	1	0	1	0	**50%**	2
Linezolida	2	0	0	0	**0%**	2
Minociclina	1	0	1	0	**50%**	2
Oxacilina	1	0	1	0	**50%**	2
Rifampicina	1	0	1	0	**50%**	2
Trimetoprim/Sulfametoxazol	2	0	0	0	**0%**	2
Vancomicina	1	0	1	0	**50%**	2
**Subtotal**	**12**	**0**	**6**	**0**	**33.33%**	**18**
**Staphylococcus warneri**						
Ampicilina	0	0	3	0	**100%**	3
Ceftaroline	8	0	0	0	**0%**	8
Ciprofloxacina	2	1	2	0	**40%**	5
Clindamicina	31	0	4	0	**11.43%**	35
Daptomicina	26	0	0	0	**0%**	26
Eritromicina	17	0	18	0	**51.43%**	35
Gentamicina	16	1	1	0	**5.56%**	18
Levofloxacina	11	0	1	0	**8.33%**	12
Linezolida	35	0	0	0	**0%**	35
Minociclina	16	1	0	0	**0%**	17
Moxifloxacina	2	0	1	0	**33.33%**	3
Nitrofurantoína	7	2	0	0	**0%**	9
Norfloxacina	1	0	2	0	**66.67%**	3
Oxacilina	25	0	7	0	**21.88%**	32
Penicilina	1	0	20	0	**95.24%**	21
Rifampicina	30	0	5	0	**14.29%**	35

Análise de Resistência Bacteriana - Teste de Sensibilidade

Microrganismo/Antibiótico	Sensível	Intermediário	Resistente	Não Testado	% Resistentes	Total
Sulfametoxazol/trimetoprim	1	0	0	0	**0%**	1
Teicoplanina	16	0	0	0	**0%**	16
Tigeciclina	14	0	0	0	**0%**	14
Trimetoprim/Sulfametoxazol	29	0	5	0	**14.71%**	34
Vancomicina	33	0	0	0	**0%**	33
**Subtotal**	**321**	**5**	**69**	**0**	**17.47%**	**395**
**Staphylococcus xylosus**						
Ampicilina	0	0	1	0	**100%**	1
Clindamicina	3	0	7	0	**70%**	10
Daptomicina	11	0	0	0	**0%**	11
Eritromicina	2	0	9	0	**81.82%**	11
Linezolida	9	0	2	0	**18.18%**	11
Minociclina	8	3	0	0	**0%**	11
Nitrofurantoína	1	0	0	0	**0%**	1
Oxacilina	2	0	8	0	**80%**	10
Penicilina	0	0	1	0	**100%**	1
Rifampicina	8	0	3	0	**27.27%**	11
Trimetoprim/Sulfametoxazol	7	0	4	0	**36.36%**	11
Vancomicina	10	0	1	0	**9.09%**	11
**Subtotal**	**61**	**3**	**36**	**0**	**36%**	**100**
**Stenotrophomonas maltophilia**						
Amicacina	2	0	1	0	**33.33%**	3
Ampicilina	0	0	2	0	**100%**	2
Ampicilina/Sulbactam	0	0	2	0	**100%**	2
Cefepime	1	0	2	0	**66.67%**	3
Cefotaxima	0	0	1	0	**100%**	1
Cefoxitina	2	0	0	0	**0%**	2
Ceftazidima	50	8	69	0	**54.33%**	127
Ceftriaxona	0	0	5	0	**100%**	5
Cefuroxima	0	0	1	0	**100%**	1
Cefuroxima axetil	0	0	1	0	**100%**	1
Ciprofloxacina	2	0	1	0	**33.33%**	3
Clindamicina	0	0	1	0	**100%**	1
Cloranfenicol	4	0	0	0	**0%**	4
Colistin	1	0	0	0	**0%**	1
Daptomicina	1	0	0	0	**0%**	1
Eritromicina	0	0	1	0	**100%**	1
Ertapenem	1	0	1	0	**50%**	2
Gentamicina	1	0	3	0	**75%**	4
Imipenem	1	0	2	0	**66.67%**	3
Levofloxacina	77	25	34	0	**25%**	136
Linezolida	1	0	0	0	**0%**	1
Meropenem	1	1	2	0	**50%**	4
Minociclina	2	0	1	0	**33.33%**	3
Oxacilina	0	0	1	0	**100%**	1
Piperacilina/Tazobactam	1	0	1	0	**50%**	2
Rifampicina	1	0	0	0	**0%**	1
Sulfametoxazol/trimetoprim	13	0	2	0	**13.33%**	15
Sulfazotrim	0	0	1	0	**100%**	1
Tetraciclina	0	0	4	0	**100%**	4
Tigeciclina	0	1	1	0	**50%**	2
Trimetoprim/Sulfametoxazol	103	0	23	0	**18.25%**	126

Análise de Resistência Bacteriana - Teste de Sensibilidade

Microrganismo/Antibiótico	Sensível	Intermediário	Resistente	Não Testado	% Resistentes	Total
Vancomicina	1	0	0	0	**0%**	1
**Subtotal**	**266**	**35**	**163**	**0**	**35.13%**	**464**
**Streptococcus acidominimus**						
Gentamicina	0	0	1	0	**100%**	1
**Subtotal**	**0**	**0**	**1**	**0**	**100%**	**1**
**Streptococcus agalactiae**						
Amoxicilina	2	0	0	0	**0%**	2
Ampicilina	14	0	0	0	**0%**	14
Cefepime	3	0	0	0	**0%**	3
Cefotaxima	3	0	0	0	**0%**	3
Ceftriaxona	1	0	0	0	**0%**	1
Ciprofloxacina	4	0	0	0	**0%**	4
Claritromicina	1	0	0	0	**0%**	1
Clindamicina	7	0	1	0	**12.5%**	8
Cloranfenicol	2	0	0	0	**0%**	2
Daptomicina	8	0	0	0	**0%**	8
Eritromicina	8	0	8	0	**50%**	16
Gentamicina	0	0	1	0	**100%**	1
Levofloxacina	10	0	0	0	**0%**	10
Linezolida	14	0	0	0	**0%**	14
Meropenem	1	0	0	0	**0%**	1
Moxifloxacina	3	0	0	0	**0%**	3
Nitrofurantoína	6	0	0	0	**0%**	6
Norfloxacina	1	0	3	0	**75%**	4
Ofloxacina	1	0	0	0	**0%**	1
Penicilina	8	0	0	0	**0%**	8
Teicoplanina	10	0	0	0	**0%**	10
Tetraciclina	1	0	0	0	**0%**	1
Tigeciclina	13	0	0	0	**0%**	13
Trimetoprim/Sulfametoxazol	8	0	0	0	**0%**	8
Vancomicina	12	0	0	0	**0%**	12
**Subtotal**	**141**	**0**	**13**	**0**	**8.44%**	**154**
**Streptococcus agalactiae (beta hemolítico do grupo B)**						
Amoxicilina	2	0	0	0	**0%**	2
Cefepime	2	0	0	0	**0%**	2
Cefotaxima	2	0	0	0	**0%**	2
Clindamicina	0	0	2	0	**100%**	2
Eritromicina	0	0	2	0	**100%**	2
Gentamicina	0	0	1	0	**100%**	1
Penicilina	2	0	0	0	**0%**	2
Vancomicina	2	0	0	0	**0%**	2
**Subtotal**	**10**	**0**	**5**	**0**	**33.33%**	**15**
**Streptococcus agalactiae hemolítico**						
Ampicilina	25	0	0	0	**0%**	25
Ampicilina/Sulbactam	2	0	0	0	**0%**	2
Ciprofloxacina	10	0	0	0	**0%**	10
Clindamicina	5	1	5	0	**45.45%**	11
Daptomicina	20	0	0	0	**0%**	20
Eritromicina	20	0	10	0	**33.33%**	30
Levofloxacina	19	0	0	0	**0%**	19
Linezolida	29	0	0	0	**0%**	29
Moxifloxacina	4	0	0	0	**0%**	4

Análise de Resistência Bacteriana - Teste de Sensibilidade

Microrganismo/Antibiótico	Sensível	Intermediário	Resistente	Não Testado	% Resistentes	Total
Nitrofurantoína	17	1	1	0	**5.26%**	19
Norfloxacina	0	0	5	0	**100%**	5
Oxacilina	1	0	0	0	**0%**	1
Penicilina	10	0	0	0	**0%**	10
Teicoplanina	19	0	0	0	**0%**	19
Tetraciclina	0	0	4	0	**100%**	4
Tigeciclina	22	0	0	0	**0%**	22
Trimetoprim/Sulfametoxazol	18	0	0	0	**0%**	18
Vancomicina	24	0	0	0	**0%**	24
**Subtotal**	**245**	**2**	**25**	**0**	**9.19%**	**272**
**Streptococcus agalactiae não-hemolítico**						
Ampicilina	7	0	0	0	**0%**	7
Ciprofloxacina	1	0	0	0	**0%**	1
Clindamicina	1	0	0	0	**0%**	1
Daptomicina	5	0	0	0	**0%**	5
Eritromicina	5	0	2	0	**28.57%**	7
Gentamicina	0	0	1	0	**100%**	1
Levofloxacina	6	0	0	0	**0%**	6
Linezolida	7	0	0	0	**0%**	7
Moxifloxacina	1	0	0	0	**0%**	1
Nitrofurantoína	5	0	0	0	**0%**	5
Penicilina	1	0	0	0	**0%**	1
Teicoplanina	4	0	0	0	**0%**	4
Tigeciclina	5	0	0	0	**0%**	5
Trimetoprim/Sulfametoxazol	3	0	0	0	**0%**	3
Vancomicina	6	0	0	0	**0%**	6
**Subtotal**	**57**	**0**	**3**	**0**	**5%**	**60**
**Streptococcus anginosus**						
Amoxicilina	1	0	0	0	**0%**	1
Azitromicina	1	0	0	0	**0%**	1
Cefepime	1	0	0	0	**0%**	1
Cefotaxima	1	0	0	0	**0%**	1
Clindamicina	1	0	0	0	**0%**	1
Eritromicina	1	0	0	0	**0%**	1
Linezolida	1	0	0	0	**0%**	1
Ofloxacina	1	0	0	0	**0%**	1
Penicilina	2	0	0	0	**0%**	2
Sulfazotrim	0	0	1	0	**100%**	1
Vancomicina	2	0	0	0	**0%**	2
**Subtotal**	**12**	**0**	**1**	**0**	**7.69%**	**13**
**Streptococcus Beta Hemolítico (não do grupo A - Lancefield)**						
Azitromicina	1	0	0	0	**0%**	1
Clindamicina	1	0	0	0	**0%**	1
Cloranfenicol	0	1	0	0	**0%**	1
Eritromicina	1	0	0	0	**0%**	1
Levofloxacina	0	0	1	0	**100%**	1
Penicilina	1	0	0	0	**0%**	1
Vancomicina	1	0	0	0	**0%**	1
**Subtotal**	**5**	**1**	**1**	**0**	**14.29%**	**7**
**Streptococcus constellatus/milleri**						
Amoxicilina	1	0	0	0	**0%**	1
Cefepime	1	0	0	0	**0%**	1

Análise de Resistência Bacteriana - Teste de Sensibilidade

Microrganismo/Antibiótico	Sensível	Intermediário	Resistente	Não Testado	% Resistentes	Total
Cefotaxima	1	0	0	0	**0%**	1
Gentamicina	0	0	1	0	**100%**	1
Penicilina	0	1	0	0	**0%**	1
Vancomicina	1	0	0	0	**0%**	1
**Subtotal**	**4**	**1**	**1**	**0**	**16.67%**	**6**
**Streptococcus intermedius**						
Clindamicina	1	0	0	0	**0%**	1
Daptomicina	1	0	0	0	**0%**	1
Eritromicina	1	0	0	0	**0%**	1
Linezolida	1	0	0	0	**0%**	1
Minociclina	1	0	0	0	**0%**	1
Oxacilina	1	0	0	0	**0%**	1
Rifampicina	1	0	0	0	**0%**	1
Trimetoprim/Sulfametoxazol	0	0	1	0	**100%**	1
Vancomicina	1	0	0	0	**0%**	1
**Subtotal**	**8**	**0**	**1**	**0**	**11.11%**	**9**
**Streptococcus mitis**						
Amicacina	2	0	0	0	**0%**	2
Amoxicilina	25	7	6	0	**15.79%**	38
Cefepime	30	5	6	0	**14.63%**	41
Cefotaxima	29	1	8	0	**21.05%**	38
Cefoxitina	2	0	0	0	**0%**	2
Ceftazidima	1	0	0	0	**0%**	1
Ciprofloxacina	1	0	0	0	**0%**	1
Gentamicina	0	1	0	0	**0%**	1
Imipenem	1	0	0	0	**0%**	1
Levofloxacina	1	0	0	0	**0%**	1
Meropenem	1	0	0	0	**0%**	1
Penicilina	21	10	8	0	**20.51%**	39
Piperacilina/Tazobactam	1	0	0	0	**0%**	1
Trimetoprim/Sulfametoxazol	1	0	0	0	**0%**	1
Vancomicina	38	0	0	0	**0%**	38
**Subtotal**	**154**	**24**	**28**	**0**	**13.59%**	**206**
**Streptococcus mitis/Streptococcus oralis**						
Amoxicilina	1	0	0	0	**0%**	1
Cefepime	1	0	0	0	**0%**	1
Cefotaxima	1	0	0	0	**0%**	1
Penicilina	1	0	0	0	**0%**	1
Vancomicina	1	0	0	0	**0%**	1
**Subtotal**	**5**	**0**	**0**	**0**	**0%**	**5**
**Streptococcus pneumoniae**						
Ampicilina	6	0	0	0	**0%**	6
Azitromicina	4	0	1	0	**20%**	5
Cefepime	2	0	0	0	**0%**	2
Cefotaxima	7	0	0	0	**0%**	7
Cefoxitina	1	0	0	0	**0%**	1
Ceftriaxona	7	0	0	0	**0%**	7
Cefuroxima	1	0	0	0	**0%**	1
Ciprofloxacina	1	0	0	0	**0%**	1
Claritromicina	0	0	1	0	**100%**	1
Clindamicina	21	0	1	0	**4.55%**	22
Cloranfenicol	8	0	0	0	**0%**	8

Análise de Resistência Bacteriana - Teste de Sensibilidade

Microrganismo/Antibiótico	Sensível	Intermediário	Resistente	Não Testado	% Resistentes	Total
Eritromicina	15	0	3	0	**16.67%**	18
Ertapenem	1	0	0	0	**0%**	1
Gentamicina	4	0	1	0	**20%**	5
Imipenem	1	0	0	0	**0%**	1
Levofloxacina	15	2	0	0	**0%**	17
Linezolida	16	0	0	0	**0%**	16
Meropenem	2	0	0	0	**0%**	2
Moxifloxacina	9	0	0	0	**0%**	9
Ofloxacina	4	0	0	0	**0%**	4
Oxacilina	2	0	1	0	**33.33%**	3
Penicilina	16	0	0	0	**0%**	16
Rifampicina	15	0	0	0	**0%**	15
Sulfametoxazol/trimetoprim	6	0	5	0	**45.45%**	11
Sulfazotrim	0	0	2	0	**100%**	2
Teicoplanina	5	0	0	0	**0%**	5
Tetraciclina	15	1	3	0	**15.79%**	19
Tigeciclina	7	0	0	0	**0%**	7
Trimetoprim/Sulfametoxazol	8	0	1	0	**11.11%**	9
Vancomicina	18	0	0	0	**0%**	18
**Subtotal**	**217**	**3**	**19**	**0**	**7.95%**	**239**
**Streptococcus pyogenes (beta hemolítico grupo A)**						
Amoxicilina	1	0	0	0	**0%**	1
Azitromicina	0	0	1	0	**100%**	1
Cefepime	2	0	0	0	**0%**	2
Cefotaxima	1	0	0	0	**0%**	1
Ceftriaxona	1	0	0	0	**0%**	1
Clindamicina	3	0	1	0	**25%**	4
Cloranfenicol	2	0	0	0	**0%**	2
Eritromicina	3	0	1	0	**25%**	4
Ertapenem	1	0	0	0	**0%**	1
Levofloxacina	1	0	1	0	**50%**	2
Linezolida	1	0	0	0	**0%**	1
Meropenem	1	0	0	0	**0%**	1
Ofloxacina	1	0	0	0	**0%**	1
Penicilina	3	0	0	0	**0%**	3
Tetraciclina	0	0	1	0	**100%**	1
Vancomicina	3	0	0	0	**0%**	3
**Subtotal**	**24**	**0**	**5**	**0**	**17.24%**	**29**
**Streptococcus pyogenes (grupo A - Lancefield)**						
Amicacina	0	0	1	0	**100%**	1
Amoxicilina	2	0	0	0	**0%**	2
Azitromicina	2	0	0	0	**0%**	2
Cefepime	4	0	0	0	**0%**	4
Cefotaxima	1	0	0	0	**0%**	1
Ceftriaxona	0	0	1	0	**100%**	1
Cefuroxima	1	0	0	0	**0%**	1
Claritromicina	2	0	0	0	**0%**	2
Clindamicina	6	0	0	0	**0%**	6
Cloranfenicol	1	1	0	0	**0%**	2
Daptomicina	1	0	0	0	**0%**	1
Eritromicina	6	0	0	0	**0%**	6
Levofloxacina	2	0	1	0	**33.33%**	3

Análise de Resistência Bacteriana - Teste de Sensibilidade

Microrganismo/Antibiótico	Sensível	Intermediário	Resistente	Não Testado	% Resistentes	Total
Linezolida	2	0	0	0	**0%**	2
Meropenem	1	0	0	0	**0%**	1
Ofloxacina	1	0	1	0	**50%**	2
Penicilina	5	0	0	0	**0%**	5
Teicoplanina	1	0	0	0	**0%**	1
Tetraciclina	2	0	0	0	**0%**	2
Vancomicina	5	0	0	0	**0%**	5
**Subtotal**	**45**	**1**	**4**	**0**	**8%**	**50**
**Streptococcus salivarius**						
Amoxicilina	1	0	0	0	**0%**	1
Cefepime	1	0	0	0	**0%**	1
Cefotaxima	1	0	0	0	**0%**	1
Gentamicina	0	0	1	0	**100%**	1
Penicilina	0	1	0	0	**0%**	1
Vancomicina	1	0	0	0	**0%**	1
**Subtotal**	**4**	**1**	**1**	**0**	**16.67%**	**6**
**Streptococcus sobrinus**						
Amoxicilina	0	0	1	0	**100%**	1
Cefepime	0	0	1	0	**100%**	1
Cefotaxima	0	0	1	0	**100%**	1
Penicilina	0	0	1	0	**100%**	1
**Subtotal**	**0**	**0**	**4**	**0**	**100%**	**4**
**Streptococcus sp.**						
Amoxicilina	2	1	1	0	**25%**	4
Ampicilina	2	0	0	0	**0%**	2
Azitromicina	0	0	1	0	**100%**	1
Cefepime	2	0	2	0	**50%**	4
Cefotaxima	4	0	1	0	**20%**	5
Cefoxitina	1	0	0	0	**0%**	1
Ceftaroline	1	0	0	0	**0%**	1
Ceftriaxona	1	0	0	0	**0%**	1
Ciprofloxacina	0	0	2	0	**100%**	2
Clindamicina	4	0	3	0	**42.86%**	7
Cloranfenicol	2	0	0	0	**0%**	2
Daptomicina	2	0	0	0	**0%**	2
Eritromicina	2	0	6	0	**75%**	8
Gentamicina	3	0	2	0	**40%**	5
Levofloxacina	3	0	0	0	**0%**	3
Linezolida	7	0	0	0	**0%**	7
Minociclina	1	0	0	0	**0%**	1
Moxifloxacina	1	0	0	0	**0%**	1
Nitrofurantoína	1	0	1	0	**50%**	2
Oxacilina	1	0	4	0	**80%**	5
Penicilina	3	3	2	0	**25%**	8
Rifampicina	7	0	0	0	**0%**	7
Sulfametoxazol/trimetoprim	3	0	2	0	**40%**	5
Teicoplanina	2	0	0	0	**0%**	2
Tetraciclina	1	0	0	0	**0%**	1
Tigeciclina	3	0	0	0	**0%**	3
Trimetoprim/Sulfametoxazol	2	0	0	0	**0%**	2
Vancomicina	8	0	0	0	**0%**	8
**Subtotal**	**69**	**4**	**27**	**0**	**27%**	**100**

Análise de Resistência Bacteriana - Teste de Sensibilidade

Microrganismo/Antibiótico	Sensível	Intermediário	Resistente	Não Testado	% Resistentes	Total
**Streptococcus thoraltensis**						
Clindamicina	0	0	1	0	**100%**	1
Cloranfenicol	0	0	1	0	**100%**	1
Eritromicina	0	0	1	0	**100%**	1
Levofloxacina	0	0	1	0	**100%**	1
Moxifloxacina	0	0	1	0	**100%**	1
Sulfametoxazol/trimetoprim	0	0	1	0	**100%**	1
Tetraciclina	0	0	1	0	**100%**	1
**Subtotal**	**0**	**0**	**7**	**0**	**100%**	**7**
**Streptococcus viridans**						
Ampicilina	0	2	0	0	**0%**	2
Cefotaxima	0	0	2	0	**100%**	2
Ceftriaxona	0	0	2	0	**100%**	2
Clindamicina	2	0	2	0	**50%**	4
Cloranfenicol	2	0	0	0	**0%**	2
Eritromicina	0	0	2	0	**100%**	2
Levofloxacina	3	0	0	0	**0%**	3
Linezolida	2	0	0	0	**0%**	2
Moxifloxacina	2	0	0	0	**0%**	2
Penicilina	0	2	0	0	**0%**	2
Rifampicina	1	0	0	0	**0%**	1
Sulfametoxazol/trimetoprim	1	0	0	0	**0%**	1
Tetraciclina	2	0	2	0	**50%**	4
Tigeciclina	2	0	0	0	**0%**	2
Vancomicina	2	0	0	0	**0%**	2
**Subtotal**	**19**	**4**	**10**	**0**	**30.3%**	**33**
**Yersinia intermedia**						
Amicacina	0	0	1	0	**100%**	1
Ampicilina	0	0	1	0	**100%**	1
Ampicilina/Sulbactam	0	0	1	0	**100%**	1
Cefepime	0	0	1	0	**100%**	1
Cefoxitina	0	0	1	0	**100%**	1
Ceftazidima	0	0	1	0	**100%**	1
Ceftriaxona	0	0	1	0	**100%**	1
Cefuroxima	0	0	1	0	**100%**	1
Cefuroxima axetil	0	0	1	0	**100%**	1
Ciprofloxacina	0	1	0	0	**0%**	1
Ertapenem	0	0	1	0	**100%**	1
Gentamicina	1	0	0	0	**0%**	1
Imipenem	0	0	1	0	**100%**	1
Meropenem	0	0	1	0	**100%**	1
Piperacilina/Tazobactam	0	0	1	0	**100%**	1
Tigeciclina	1	0	0	0	**0%**	1
**Subtotal**	**2**	**1**	**13**	**0**	**81.25%**	**16**
Total Geral	***96570***	***6297***	***73069***	***0***	***41.53%***	***175936***

Observação:

% Resistentes - *O percentual de resistentes é calculado levando em consideração o valor da coluna "Resistentes" em relação ao valor da coluna "Total".*
