# Supplementary material for: Biodiversity of carbapenem-resistant bacteria in clinical samples from the Southwest Amazon region (Rondônia/Brazil)
Source: Sci Rep. 2024 Apr 23;14:9383. doi: 10.1038/s41598-024-59733-w (PMC11039742; doi:10.1038/s41598-024-59733-w)
Supplement: Supplementary file 6 — Supplementary Information 6. [file 41598_2024_59733_MOESM6_ESM.pdf]

Data Início: 01/01/2018

Qtd. de Exame/Metodologia: 2127

Todos os Laboratórios

Microrganismo /Gene Pesquisado	Jan/2018	Fev/2018	Mar/2018	Abr/2018	Maio/2018	Jun/2018	Jul/2018	Ago/2018	Set/2018	Out/2018	Nov/2018	Dez/2018	Jan/2019	Fev/2019	Mar/2019	Abr/2019	Maio/2019	Jun/2019	Jul/2019
**Acinetobacter baumannii/bla IMP**																			
Inconclusivo	0	0	0	0	0	0	0	0	0	0	0	0	0	0	0	0	0	0	0
Não Detectável	0	0	0	5	0	0	0	0	0	0	0	0	0	0	0	0	0	0	0
**Subtotal**	**0**	**0**	**0**	**5**	**0**	**0**	**0**	**0**	**0**	**0**	**0**	**0**	**0**	**0**	**0**	**0**	**0**	**0**	**0**
**Acinetobacter baumannii/bla KPC**																			
Detectável	0	0	0	0	0	0	0	0	0	0	0	0	0	0	0	0	0	0	0
Inconclusivo	0	0	0	0	0	0	0	0	0	0	0	0	0	0	0	0	0	0	0
Não Detectável	0	0	0	5	0	0	0	0	0	0	0	0	0	0	0	0	0	0	0
**Subtotal**	**0**	**0**	**0**	**5**	**0**	**0**	**0**	**0**	**0**	**0**	**0**	**0**	**0**	**0**	**0**	**0**	**0**	**0**	**0**
**Acinetobacter baumannii/bla NDM**																			
Detectado traços	0	0	0	0	0	0	0	0	0	0	0	0	0	0	0	0	0	0	0
Detectável	0	0	0	0	0	0	0	0	0	0	0	0	0	0	0	0	0	0	0
Não Detectável	0	0	0	0	0	0	0	0	0	0	0	0	0	0	0	0	0	0	0
**Subtotal**	**0**	**0**	**0**	**0**	**0**	**0**	**0**	**0**	**0**	**0**	**0**	**0**	**0**	**0**	**0**	**0**	**0**	**0**	**0**
**Acinetobacter baumannii/bla OXA-143**																			
Detectável	0	0	0	0	1	0	0	0	0	0	0	0	0	0	0	0	0	0	0
Inconclusivo	0	0	0	0	0	0	0	0	0	0	0	0	0	0	0	0	0	0	0
Não Detectável	0	0	0	5	0	0	0	0	0	0	0	0	0	0	0	0	0	3	0
**Subtotal**	**0**	**0**	**0**	**5**	**1**	**0**	**0**	**0**	**0**	**0**	**0**	**0**	**0**	**0**	**0**	**0**	**0**	**3**	**0**
**Acinetobacter baumannii/bla OXA-23**																			
Detectado traços	0	0	0	0	0	0	0	0	0	0	0	0	0	0	0	0	0	0	0
Detectável	0	0	0	5	2	12	0	17	19	8	0	29	14	8	16	6	12	5	11
Inconclusivo	0	0	0	0	0	0	0	0	0	0	0	0	0	0	0	0	0	0	0
Não Detectável	0	0	0	0	0	1	0	8	5	3	0	5	0	0	4	1	2	3	8
**Subtotal**	**0**	**0**	**0**	**5**	**2**	**13**	**0**	**25**	**24**	**11**	**0**	**34**	**14**	**8**	**20**	**7**	**14**	**8**	**19**
**Acinetobacter baumannii/bla OXA-24**																			
Detectável	0	0	0	0	0	0	0	0	0	0	0	0	0	0	0	0	0	0	0
Não Detectável	0	0	0	5	0	0	0	0	0	0	0	0	0	0	0	0	0	0	0
**Subtotal**	**0**	**0**	**0**	**5**	**0**	**0**	**0**	**0**	**0**	**0**	**0**	**0**	**0**	**0**	**0**	**0**	**0**	**0**	**0**
**Acinetobacter baumannii/bla OXA-48**																			
Detectável	0	0	0	0	0	0	0	0	0	0	0	0	0	0	0	0	0	0	0
Inconclusivo	0	0	0	0	0	0	0	0	0	0	0	0	0	0	0	0	0	0	0
Não Detectável	0	0	0	5	0	0	0	4	0	0	0	0	0	0	0	0	1	0	1
**Subtotal**	**0**	**0**	**0**	**5**	**0**	**0**	**0**	**4**	**0**	**0**	**0**	**0**	**0**	**0**	**0**	**0**	**1**	**0**	**1**
**Acinetobacter baumannii/bla OXA-51**																			
Detectado traços	0	0	0	0	0	0	0	0	0	0	0	0	0	0	0	0	0	0	0
Detectável	0	0	0	5	0	14	0	5	2	0	0	20	14	8	15	6	14	7	18
Inconclusivo	0	0	0	0	0	0	0	0	0	0	0	0	0	0	0	0	0	0	0
Não Detectável	0	0	0	0	0	1	0	20	21	11	0	5	0	0	3	1	2	1	1
**Subtotal**	**0**	**0**	**0**	**5**	**0**	**15**	**0**	**25**	**23**	**11**	**0**	**25**	**14**	**8**	**18**	**7**	**16**	**8**	**19**
**Acinetobacter baumannii/bla OXA-58**																			
Detectado traços	0	0	0	0	0	0	0	0	0	0	0	0	0	0	0	0	0	0	0
Detectável	0	0	0	0	0	1	0	1	0	0	0	1	0	0	0	0	0	0	0
Inconclusivo	0	0	0	0	0	0	0	0	0	0	0	0	0	0	0	0	0	0	0

Microrganismo /Gene Pesquisado	Jan/2018	Fev/2018	Mar/2018	Abr/2018	Maio/2018	Jun/2018	Jul/2018	Ago/2018	Set/2018	Out/2018	Nov/2018	Dez/2018	Jan/2019	Fev/2019	Mar/2019	Abr/2019	Maio/2019	Jun/2019	Jul/2019
Não Detectável	0	0	0	5	0	1	0	10	0	0	0	0	0	0	0	0	0	2	0
**Subtotal**	**0**	**0**	**0**	**5**	**0**	**2**	**0**	**11**	**0**	**0**	**0**	**1**	**0**	**0**	**0**	**0**	**0**	**2**	**0**
**Acinetobacter baumannii/bla SPM**																			
Inconclusivo	0	0	0	0	0	0	0	0	0	0	0	0	0	0	0	0	0	0	0
Não Detectável	0	0	0	5	0	0	0	0	0	0	0	0	0	0	0	0	0	0	0
**Subtotal**	**0**	**0**	**0**	**5**	**0**	**0**	**0**	**0**	**0**	**0**	**0**	**0**	**0**	**0**	**0**	**0**	**0**	**0**	**0**
**Acinetobacter baumannii/blaVIM**																			
Inconclusivo	0	0	0	0	0	0	0	0	0	0	0	0	0	0	0	0	0	0	0
Não Detectável	0	0	0	0	0	0	0	0	0	0	0	0	0	0	0	0	0	0	0
**Subtotal**	**0**	**0**	**0**	**0**	**0**	**0**	**0**	**0**	**0**	**0**	**0**	**0**	**0**	**0**	**0**	**0**	**0**	**0**	**0**
**Acinetobacter baumannii/SPM**																			
Não Detectável	0	0	0	0	0	0	0	0	0	0	0	1	0	0	0	0	0	0	0
**Subtotal**	**0**	**0**	**0**	**0**	**0**	**0**	**0**	**0**	**0**	**0**	**0**	**1**	**0**	**0**	**0**	**0**	**0**	**0**	**0**
**Acinetobacter calcoaceticus/bla IMP**																			
Não Detectável	0	0	0	0	0	0	0	0	0	0	0	0	0	0	0	0	0	0	0
**Subtotal**	**0**	**0**	**0**	**0**	**0**	**0**	**0**	**0**	**0**	**0**	**0**	**0**	**0**	**0**	**0**	**0**	**0**	**0**	**0**
**Acinetobacter calcoaceticus/bla KPC**																			
Não Detectável	0	0	0	0	0	0	0	0	0	0	0	0	0	0	0	0	0	0	0
**Subtotal**	**0**	**0**	**0**	**0**	**0**	**0**	**0**	**0**	**0**	**0**	**0**	**0**	**0**	**0**	**0**	**0**	**0**	**0**	**0**
**Acinetobacter calcoaceticus/bla NDM**																			
Não Detectável	0	0	0	0	0	0	0	0	0	0	0	0	0	0	0	0	0	0	0
**Subtotal**	**0**	**0**	**0**	**0**	**0**	**0**	**0**	**0**	**0**	**0**	**0**	**0**	**0**	**0**	**0**	**0**	**0**	**0**	**0**
**Acinetobacter calcoaceticus/bla OXA-143**																			
Não Detectável	0	0	0	0	0	0	0	0	0	0	0	0	0	0	0	0	0	0	0
**Subtotal**	**0**	**0**	**0**	**0**	**0**	**0**	**0**	**0**	**0**	**0**	**0**	**0**	**0**	**0**	**0**	**0**	**0**	**0**	**0**
**Acinetobacter calcoaceticus/bla OXA-23**																			
Não Detectável	0	0	0	0	0	0	0	0	0	0	0	0	0	0	0	0	0	0	0
**Subtotal**	**0**	**0**	**0**	**0**	**0**	**0**	**0**	**0**	**0**	**0**	**0**	**0**	**0**	**0**	**0**	**0**	**0**	**0**	**0**
**Acinetobacter calcoaceticus/bla OXA-48**																			
Não Detectável	0	0	0	0	0	0	0	0	0	0	0	0	0	0	0	0	0	0	0
**Subtotal**	**0**	**0**	**0**	**0**	**0**	**0**	**0**	**0**	**0**	**0**	**0**	**0**	**0**	**0**	**0**	**0**	**0**	**0**	**0**
**Acinetobacter calcoaceticus/bla OXA-51**																			
Detectável	0	0	0	0	0	0	0	0	0	0	0	0	0	0	0	0	0	0	0
Não Detectável	0	0	0	0	0	0	0	0	0	0	0	0	0	0	0	0	0	0	0
**Subtotal**	**0**	**0**	**0**	**0**	**0**	**0**	**0**	**0**	**0**	**0**	**0**	**0**	**0**	**0**	**0**	**0**	**0**	**0**	**0**
**Acinetobacter calcoaceticus/bla OXA-58**																			
Detectável	0	0	0	0	0	0	0	0	0	0	0	0	0	0	0	0	0	0	0
Não Detectável	0	0	0	0	0	0	0	0	0	0	0	0	0	0	0	0	0	0	0
**Subtotal**	**0**	**0**	**0**	**0**	**0**	**0**	**0**	**0**	**0**	**0**	**0**	**0**	**0**	**0**	**0**	**0**	**0**	**0**	**0**
**Acinetobacter calcoaceticus/bla SPM**																			
Não Detectável	0	0	0	0	0	0	0	0	0	0	0	0	0	0	0	0	0	0	0
**Subtotal**	**0**	**0**	**0**	**0**	**0**	**0**	**0**	**0**	**0**	**0**	**0**	**0**	**0**	**0**	**0**	**0**	**0**	**0**	**0**
**Acinetobacter lwoffii/bla OXA-143**																			
Não Detectável	0	0	0	0	0	0	0	0	0	0	0	0	0	0	0	0	0	0	0
**Subtotal**	**0**	**0**	**0**	**0**	**0**	**0**	**0**	**0**	**0**	**0**	**0**	**0**	**0**	**0**	**0**	**0**	**0**	**0**	**0**
**Acinetobacter lwoffii/bla OXA-23**																			
Não Detectável	0	0	0	0	0	0	0	0	0	0	0	0	0	0	0	0	0	0	0
**Subtotal**	**0**	**0**	**0**	**0**	**0**	**0**	**0**	**0**	**0**	**0**	**0**	**0**	**0**	**0**	**0**	**0**	**0**	**0**	**0**
**Acinetobacter lwoffii/bla OXA-51**																			
Não Detectável	0	0	0	0	0	0	0	0	0	0	0	0	0	0	0	0	0	0	0
**Subtotal**	**0**	**0**	**0**	**0**	**0**	**0**	**0**	**0**	**0**	**0**	**0**	**0**	**0**	**0**	**0**	**0**	**0**	**0**	**0**
**Acinetobacter lwoffii/bla OXA-58**																			
Não Detectável	0	0	0	0	0	0	0	0	0	0	0	0	0	0	0	0	0	0	0
**Subtotal**	**0**	**0**	**0**	**0**	**0**	**0**	**0**	**0**	**0**	**0**	**0**	**0**	**0**	**0**	**0**	**0**	**0**	**0**	**0**

Microrganismo /Gene Pesquisado	Jan/2018	Fev/2018	Mar/2018	Abr/2018	Maio/2018	Jun/2018	Jul/2018	Ago/2018	Set/2018	Out/2018	Nov/2018	Dez/2018	Jan/2019	Fev/2019	Mar/2019	Abr/2019	Maio/2019	Jun/2019	Jul/2019
**Acinetobacter sp./bla IMP**																			
Não Detectável	0	0	0	0	0	0	0	0	0	0	0	0	0	0	0	0	0	0	0
**Subtotal**	**0**	**0**	**0**	**0**	**0**	**0**	**0**	**0**	**0**	**0**	**0**	**0**	**0**	**0**	**0**	**0**	**0**	**0**	**0**
**Acinetobacter sp./bla KPC**																			
Não Detectável	0	0	0	0	0	0	0	0	0	0	0	0	0	0	0	0	0	0	0
**Subtotal**	**0**	**0**	**0**	**0**	**0**	**0**	**0**	**0**	**0**	**0**	**0**	**0**	**0**	**0**	**0**	**0**	**0**	**0**	**0**
**Acinetobacter sp./bla NDM**																			
Não Detectável	0	0	0	0	0	0	0	0	0	0	0	0	0	0	0	0	0	0	0
**Subtotal**	**0**	**0**	**0**	**0**	**0**	**0**	**0**	**0**	**0**	**0**	**0**	**0**	**0**	**0**	**0**	**0**	**0**	**0**	**0**
**Acinetobacter sp./bla OXA-143**																			
Detectável	0	0	0	0	0	0	0	0	0	0	0	0	0	0	0	0	0	0	0
Não Detectável	0	0	0	0	0	0	0	0	0	0	0	0	0	0	0	0	0	0	0
**Subtotal**	**0**	**0**	**0**	**0**	**0**	**0**	**0**	**0**	**0**	**0**	**0**	**0**	**0**	**0**	**0**	**0**	**0**	**0**	**0**
**Acinetobacter sp./bla OXA-23**																			
Detectável	0	0	0	0	0	0	0	0	1	3	0	0	0	0	0	0	6	3	0
Não Detectável	0	0	0	0	0	0	0	0	0	0	0	1	0	0	0	0	0	0	0
**Subtotal**	**0**	**0**	**0**	**0**	**0**	**0**	**0**	**0**	**1**	**3**	**0**	**1**	**0**	**0**	**0**	**0**	**6**	**3**	**0**
**Acinetobacter sp./bla OXA-24**																			
Não Detectável	0	0	0	0	0	0	0	0	0	0	0	0	0	0	0	0	0	0	0
**Subtotal**	**0**	**0**	**0**	**0**	**0**	**0**	**0**	**0**	**0**	**0**	**0**	**0**	**0**	**0**	**0**	**0**	**0**	**0**	**0**
**Acinetobacter sp./bla OXA-48**																			
Não Detectável	0	0	0	0	0	0	0	0	0	0	0	0	0	0	0	0	0	0	0
**Subtotal**	**0**	**0**	**0**	**0**	**0**	**0**	**0**	**0**	**0**	**0**	**0**	**0**	**0**	**0**	**0**	**0**	**0**	**0**	**0**
**Acinetobacter sp./bla OXA-51**																			
Detectável	0	0	0	0	0	0	0	0	0	0	0	0	0	0	0	0	4	3	0
Não Detectável	0	0	0	0	0	0	0	0	0	3	0	1	0	0	0	0	0	0	0
**Subtotal**	**0**	**0**	**0**	**0**	**0**	**0**	**0**	**0**	**0**	**3**	**0**	**1**	**0**	**0**	**0**	**0**	**4**	**3**	**0**
**Acinetobacter sp./bla OXA-58**																			
Detectado traços	0	0	0	0	0	0	0	0	0	0	0	0	0	0	0	0	0	0	0
Detectável	0	0	0	0	0	0	0	0	0	0	0	0	0	0	0	0	2	0	0
Não Detectável	0	0	0	0	0	0	0	0	0	0	0	0	0	0	0	0	0	0	0
**Subtotal**	**0**	**0**	**0**	**0**	**0**	**0**	**0**	**0**	**0**	**0**	**0**	**0**	**0**	**0**	**0**	**0**	**2**	**0**	**0**
**Acinetobacter sp./bla SPM**																			
Não Detectável	0	0	0	0	0	0	0	0	0	0	0	0	0	0	0	0	0	0	0
**Subtotal**	**0**	**0**	**0**	**0**	**0**	**0**	**0**	**0**	**0**	**0**	**0**	**0**	**0**	**0**	**0**	**0**	**0**	**0**	**0**
**Acinetobacter sp./blaVIM**																			
Não Detectável	0	0	0	0	0	0	0	0	0	0	0	0	0	0	0	0	0	0	0
**Subtotal**	**0**	**0**	**0**	**0**	**0**	**0**	**0**	**0**	**0**	**0**	**0**	**0**	**0**	**0**	**0**	**0**	**0**	**0**	**0**
**Burkholderia cepacia/bla KPC**																			
Não Detectável	0	0	0	0	0	0	0	0	0	0	0	0	0	0	0	0	0	0	0
**Subtotal**	**0**	**0**	**0**	**0**	**0**	**0**	**0**	**0**	**0**	**0**	**0**	**0**	**0**	**0**	**0**	**0**	**0**	**0**	**0**
**Burkholderia cepacia/bla NDM**																			
Não Detectável	0	0	0	0	0	0	0	0	0	0	0	0	0	0	0	0	0	0	0
**Subtotal**	**0**	**0**	**0**	**0**	**0**	**0**	**0**	**0**	**0**	**0**	**0**	**0**	**0**	**0**	**0**	**0**	**0**	**0**	**0**
**Burkholderia cepacia/bla OXA-143**																			
Detectável	0	0	0	0	0	0	0	0	0	0	0	0	0	0	0	0	0	0	0
**Subtotal**	**0**	**0**	**0**	**0**	**0**	**0**	**0**	**0**	**0**	**0**	**0**	**0**	**0**	**0**	**0**	**0**	**0**	**0**	**0**
**Burkholderia cepacia/bla OXA-23**																			
Detectável	0	0	0	0	0	0	0	0	0	0	0	0	0	0	0	0	0	0	0
Não Detectável	0	0	0	0	0	0	0	0	0	0	0	0	0	0	0	0	0	0	0
**Subtotal**	**0**	**0**	**0**	**0**	**0**	**0**	**0**	**0**	**0**	**0**	**0**	**0**	**0**	**0**	**0**	**0**	**0**	**0**	**0**
**Burkholderia cepacia/bla OXA-48**																			
Não Detectável	0	0	0	0	0	0	0	0	0	0	0	0	0	0	0	0	0	0	0
**Subtotal**	**0**	**0**	**0**	**0**	**0**	**0**	**0**	**0**	**0**	**0**	**0**	**0**	**0**	**0**	**0**	**0**	**0**	**0**	**0**

**Microrganismo /Gene Pesquisado**	**Jan/2018**	**Fev/2018**	**Mar/2018**	**Abr/2018**	**Maio/2018**	**Jun/2018**	**Jul/2018**	**Ago/2018**	**Set/2018**	**Out/2018**	**Nov/2018**	**Dez/2018**	**Jan/2019**	**Fev/2019**	**Mar/2019**	**Abr/2019**	**Maio/2019**	**Jun/2019**	**Jul/2019**
**Burkholderia cepacia/bla OXA-51**																			
Detectável	0	0	0	0	0	0	0	0	0	0	0	0	0	0	0	0	0	0	0
Não Detectável	0	0	0	0	0	0	0	0	0	0	0	0	0	0	0	0	0	0	0
**Subtotal**	**0**	**0**	**0**	**0**	**0**	**0**	**0**	**0**	**0**	**0**	**0**	**0**	**0**	**0**	**0**	**0**	**0**	**0**	**0**
**Burkholderia cepacia complex/bla IMP**																			
Não Detectável	0	0	0	0	0	0	0	0	0	0	0	0	0	0	0	0	0	0	0
**Subtotal**	**0**	**0**	**0**	**0**	**0**	**0**	**0**	**0**	**0**	**0**	**0**	**0**	**0**	**0**	**0**	**0**	**0**	**0**	**0**
**Burkholderia cepacia complex/bla KPC**																			
Não Detectável	0	0	0	0	0	0	0	0	0	0	0	0	0	0	0	0	0	0	0
**Subtotal**	**0**	**0**	**0**	**0**	**0**	**0**	**0**	**0**	**0**	**0**	**0**	**0**	**0**	**0**	**0**	**0**	**0**	**0**	**0**
**Burkholderia cepacia complex/bla NDM**																			
Não Detectável	0	0	0	0	0	0	0	0	0	0	0	0	0	0	0	0	0	0	0
**Subtotal**	**0**	**0**	**0**	**0**	**0**	**0**	**0**	**0**	**0**	**0**	**0**	**0**	**0**	**0**	**0**	**0**	**0**	**0**	**0**
**Burkholderia cepacia complex/bla OXA-48**																			
Não Detectável	0	0	0	0	0	0	0	0	0	0	0	0	0	0	0	0	0	0	0
**Subtotal**	**0**	**0**	**0**	**0**	**0**	**0**	**0**	**0**	**0**	**0**	**0**	**0**	**0**	**0**	**0**	**0**	**0**	**0**	**0**
**Burkholderia cepacia complex/bla SPM**																			
Não Detectável	0	0	0	0	0	0	0	0	0	0	0	0	0	0	0	0	0	0	0
**Subtotal**	**0**	**0**	**0**	**0**	**0**	**0**	**0**	**0**	**0**	**0**	**0**	**0**	**0**	**0**	**0**	**0**	**0**	**0**	**0**
**Burkholderia cepacia complex/blaVIM**																			
Não Detectável	0	0	0	0	0	0	0	0	0	0	0	0	0	0	0	0	0	0	0
**Subtotal**	**0**	**0**	**0**	**0**	**0**	**0**	**0**	**0**	**0**	**0**	**0**	**0**	**0**	**0**	**0**	**0**	**0**	**0**	**0**
**Cedecea lapagei/bla KPC**																			
Não Detectável	0	0	0	0	0	0	0	0	0	0	0	0	0	0	0	0	0	0	0
**Subtotal**	**0**	**0**	**0**	**0**	**0**	**0**	**0**	**0**	**0**	**0**	**0**	**0**	**0**	**0**	**0**	**0**	**0**	**0**	**0**
**Cedecea lapagei/bla NDM**																			
Detectável	0	0	0	0	0	0	0	0	0	0	0	0	0	0	0	0	0	0	0
**Subtotal**	**0**	**0**	**0**	**0**	**0**	**0**	**0**	**0**	**0**	**0**	**0**	**0**	**0**	**0**	**0**	**0**	**0**	**0**	**0**
**Cedecea lapagei/bla OXA-48**																			
Não Detectável	0	0	0	0	0	0	0	0	0	0	0	0	0	0	0	0	0	0	0
**Subtotal**	**0**	**0**	**0**	**0**	**0**	**0**	**0**	**0**	**0**	**0**	**0**	**0**	**0**	**0**	**0**	**0**	**0**	**0**	**0**
**Cedecea lapagei/mcr-1**																			
Não Detectável	0	0	0	0	0	0	0	0	0	0	0	0	0	0	0	0	0	0	0
**Subtotal**	**0**	**0**	**0**	**0**	**0**	**0**	**0**	**0**	**0**	**0**	**0**	**0**	**0**	**0**	**0**	**0**	**0**	**0**	**0**
**Citrobacter braakii/bla IMP**																			
Não Detectável	0	0	0	0	0	0	0	0	0	0	0	0	0	0	0	0	0	0	0
**Subtotal**	**0**	**0**	**0**	**0**	**0**	**0**	**0**	**0**	**0**	**0**	**0**	**0**	**0**	**0**	**0**	**0**	**0**	**0**	**0**
**Citrobacter braakii/bla KPC**																			
Não Detectável	0	0	0	0	0	0	0	0	0	0	0	0	0	0	0	0	0	0	0
**Subtotal**	**0**	**0**	**0**	**0**	**0**	**0**	**0**	**0**	**0**	**0**	**0**	**0**	**0**	**0**	**0**	**0**	**0**	**0**	**0**
**Citrobacter braakii/bla NDM**																			
Não Detectável	0	0	0	0	0	0	0	0	0	0	0	0	0	0	0	0	0	0	0
**Subtotal**	**0**	**0**	**0**	**0**	**0**	**0**	**0**	**0**	**0**	**0**	**0**	**0**	**0**	**0**	**0**	**0**	**0**	**0**	**0**
**Citrobacter braakii/bla OXA-48**																			
Não Detectável	0	0	0	0	0	0	0	0	0	0	0	0	0	0	0	0	0	0	0
**Subtotal**	**0**	**0**	**0**	**0**	**0**	**0**	**0**	**0**	**0**	**0**	**0**	**0**	**0**	**0**	**0**	**0**	**0**	**0**	**0**
**Citrobacter braakii/bla SPM**																			
Não Detectável	0	0	0	0	0	0	0	0	0	0	0	0	0	0	0	0	0	0	0
**Subtotal**	**0**	**0**	**0**	**0**	**0**	**0**	**0**	**0**	**0**	**0**	**0**	**0**	**0**	**0**	**0**	**0**	**0**	**0**	**0**
**Citrobacter braakii/blaVIM**																			
Não Detectável	0	0	0	0	0	0	0	0	0	0	0	0	0	0	0	0	0	0	0
**Subtotal**	**0**	**0**	**0**	**0**	**0**	**0**	**0**	**0**	**0**	**0**	**0**	**0**	**0**	**0**	**0**	**0**	**0**	**0**	**0**
**Citrobacter freundii/bla IMP**																			
Não Detectável	0	0	0	0	0	0	0	0	0	0	0	1	0	0	0	0	0	0	0

Microrganismo /Gene Pesquisado	Jan/2018	Fev/2018	Mar/2018	Abr/2018	Maio/2018	Jun/2018	Jul/2018	Ago/2018	Set/2018	Out/2018	Nov/2018	Dez/2018	Jan/2019	Fev/2019	Mar/2019	Abr/2019	Maio/2019	Jun/2019	Jul/2019
**Subtotal**	**0**	**0**	**0**	**0**	**0**	**0**	**0**	**0**	**0**	**0**	**0**	**1**	**0**	**0**	**0**	**0**	**0**	**0**	**0**
**Citrobacter freundii/bla KPC**																			
Detectável	0	0	0	0	0	0	0	0	1	0	0	1	0	0	0	0	0	0	0
Não Detectável	0	0	0	0	0	0	0	0	0	0	0	0	0	0	0	0	0	0	0
**Subtotal**	**0**	**0**	**0**	**0**	**0**	**0**	**0**	**0**	**1**	**0**	**0**	**1**	**0**	**0**	**0**	**0**	**0**	**0**	**0**
**Citrobacter freundii/bla NDM**																			
Não Detectável	0	0	0	0	0	0	0	0	0	0	0	1	0	0	0	0	0	0	0
**Subtotal**	**0**	**0**	**0**	**0**	**0**	**0**	**0**	**0**	**0**	**0**	**0**	**1**	**0**	**0**	**0**	**0**	**0**	**0**	**0**
**Citrobacter freundii/bla OXA-48**																			
Não Detectável	0	0	0	0	0	0	0	0	0	0	0	0	0	0	0	0	0	0	0
**Subtotal**	**0**	**0**	**0**	**0**	**0**	**0**	**0**	**0**	**0**	**0**	**0**	**0**	**0**	**0**	**0**	**0**	**0**	**0**	**0**
**Citrobacter freundii/bla SPM**																			
Não Detectável	0	0	0	0	0	0	0	0	0	0	0	0	0	0	0	0	0	0	0
**Subtotal**	**0**	**0**	**0**	**0**	**0**	**0**	**0**	**0**	**0**	**0**	**0**	**0**	**0**	**0**	**0**	**0**	**0**	**0**	**0**
**Citrobacter freundii/blaVIM**																			
Não Detectável	0	0	0	0	0	0	0	0	0	0	0	0	0	0	0	0	0	0	0
**Subtotal**	**0**	**0**	**0**	**0**	**0**	**0**	**0**	**0**	**0**	**0**	**0**	**0**	**0**	**0**	**0**	**0**	**0**	**0**	**0**
**Citrobacter koseri/bla IMP**																			
Não Detectável	0	0	0	0	0	0	0	0	0	0	0	0	0	0	0	0	0	0	0
**Subtotal**	**0**	**0**	**0**	**0**	**0**	**0**	**0**	**0**	**0**	**0**	**0**	**0**	**0**	**0**	**0**	**0**	**0**	**0**	**0**
**Citrobacter koseri/bla KPC**																			
Detectável	0	0	0	0	0	0	0	0	0	0	0	0	0	0	0	0	0	0	0
Não Detectável	0	0	0	0	0	0	0	0	0	0	0	0	0	0	0	0	0	0	0
**Subtotal**	**0**	**0**	**0**	**0**	**0**	**0**	**0**	**0**	**0**	**0**	**0**	**0**	**0**	**0**	**0**	**0**	**0**	**0**	**0**
**Citrobacter koseri/bla NDM**																			
Não Detectável	0	0	0	0	0	0	0	0	0	0	0	0	0	0	0	0	0	0	0
**Subtotal**	**0**	**0**	**0**	**0**	**0**	**0**	**0**	**0**	**0**	**0**	**0**	**0**	**0**	**0**	**0**	**0**	**0**	**0**	**0**
**Citrobacter koseri/bla OXA-48**																			
Não Detectável	0	0	0	0	0	0	0	0	0	0	0	0	0	0	0	0	0	0	0
**Subtotal**	**0**	**0**	**0**	**0**	**0**	**0**	**0**	**0**	**0**	**0**	**0**	**0**	**0**	**0**	**0**	**0**	**0**	**0**	**0**
**Citrobacter koseri/bla SPM**																			
Não Detectável	0	0	0	0	0	0	0	0	0	0	0	0	0	0	0	0	0	0	0
**Subtotal**	**0**	**0**	**0**	**0**	**0**	**0**	**0**	**0**	**0**	**0**	**0**	**0**	**0**	**0**	**0**	**0**	**0**	**0**	**0**
**Citrobacter koseri/blaVIM**																			
Não Detectável	0	0	0	0	0	0	0	0	0	0	0	0	0	0	0	0	0	0	0
**Subtotal**	**0**	**0**	**0**	**0**	**0**	**0**	**0**	**0**	**0**	**0**	**0**	**0**	**0**	**0**	**0**	**0**	**0**	**0**	**0**
**Citrobacter sp./bla KPC**																			
Não Detectável	0	0	0	0	0	0	0	0	0	0	0	0	0	0	0	0	0	0	0
**Subtotal**	**0**	**0**	**0**	**0**	**0**	**0**	**0**	**0**	**0**	**0**	**0**	**0**	**0**	**0**	**0**	**0**	**0**	**0**	**0**
**Enterobacter aerogenes/bla KPC**																			
Detectável	0	0	0	0	0	0	0	0	1	0	0	0	0	0	0	0	0	0	0
Não Detectável	0	0	0	0	0	0	0	0	0	0	0	0	0	0	0	0	0	0	0
**Subtotal**	**0**	**0**	**0**	**0**	**0**	**0**	**0**	**0**	**1**	**0**	**0**	**0**	**0**	**0**	**0**	**0**	**0**	**0**	**0**
**Enterobacter aerogenes/NDM**																			
Não Detectável	0	0	0	0	0	0	0	0	0	0	0	0	0	0	0	0	0	0	0
**Subtotal**	**0**	**0**	**0**	**0**	**0**	**0**	**0**	**0**	**0**	**0**	**0**	**0**	**0**	**0**	**0**	**0**	**0**	**0**	**0**
**Enterobacter cancerogenus/bla IMP**																			
Não Detectável	0	0	0	0	0	0	0	0	0	0	0	0	0	0	0	0	0	0	0
**Subtotal**	**0**	**0**	**0**	**0**	**0**	**0**	**0**	**0**	**0**	**0**	**0**	**0**	**0**	**0**	**0**	**0**	**0**	**0**	**0**
**Enterobacter cancerogenus/bla KPC**																			
Não Detectável	0	0	0	0	0	0	0	0	0	0	0	0	0	0	0	0	0	0	0
**Subtotal**	**0**	**0**	**0**	**0**	**0**	**0**	**0**	**0**	**0**	**0**	**0**	**0**	**0**	**0**	**0**	**0**	**0**	**0**	**0**
**Enterobacter cancerogenus/bla NDM**																			
Não Detectável	0	0	0	0	0	0	0	0	0	0	0	0	0	0	0	0	0	0	0

Microrganismo /Gene Pesquisado	Jan/2018	Fev/2018	Mar/2018	Abr/2018	Maio/2018	Jun/2018	Jul/2018	Ago/2018	Set/2018	Out/2018	Nov/2018	Dez/2018	Jan/2019	Fev/2019	Mar/2019	Abr/2019	Maio/2019	Jun/2019	Jul/2019
**Subtotal**	**0**	**0**	**0**	**0**	**0**	**0**	**0**	**0**	**0**	**0**	**0**	**0**	**0**	**0**	**0**	**0**	**0**	**0**	**0**
**Enterobacter cancerogenus/bla OXA-48**																			
Não Detectável	0	0	0	0	0	0	0	0	0	0	0	0	0	0	0	0	0	0	0
**Subtotal**	**0**	**0**	**0**	**0**	**0**	**0**	**0**	**0**	**0**	**0**	**0**	**0**	**0**	**0**	**0**	**0**	**0**	**0**	**0**
**Enterobacter cancerogenus/bla SPM**																			
Não Detectável	0	0	0	0	0	0	0	0	0	0	0	0	0	0	0	0	0	0	0
**Subtotal**	**0**	**0**	**0**	**0**	**0**	**0**	**0**	**0**	**0**	**0**	**0**	**0**	**0**	**0**	**0**	**0**	**0**	**0**	**0**
**Enterobacter cancerogenus/blaVIM**																			
Não Detectável	0	0	0	0	0	0	0	0	0	0	0	0	0	0	0	0	0	0	0
**Subtotal**	**0**	**0**	**0**	**0**	**0**	**0**	**0**	**0**	**0**	**0**	**0**	**0**	**0**	**0**	**0**	**0**	**0**	**0**	**0**
**Enterobacter cloacae/bla IMP**																			
Não Detectável	0	0	0	0	0	0	0	0	0	0	0	0	0	0	0	0	0	0	0
**Subtotal**	**0**	**0**	**0**	**0**	**0**	**0**	**0**	**0**	**0**	**0**	**0**	**0**	**0**	**0**	**0**	**0**	**0**	**0**	**0**
**Enterobacter cloacae/bla KPC**																			
Detectável	0	0	0	0	0	0	0	1	0	0	0	0	0	0	0	1	1	0	0
Não Detectável	0	0	0	0	0	0	0	1	1	0	0	0	0	0	0	0	0	0	1
**Subtotal**	**0**	**0**	**0**	**0**	**0**	**0**	**0**	**2**	**1**	**0**	**0**	**0**	**0**	**0**	**0**	**1**	**1**	**0**	**1**
**Enterobacter cloacae/bla NDM**																			
Detectável	0	0	0	0	0	0	0	0	0	0	0	0	0	0	0	0	0	0	0
Não Detectável	0	0	0	0	0	0	0	1	0	0	0	0	0	0	0	0	0	0	0
**Subtotal**	**0**	**0**	**0**	**0**	**0**	**0**	**0**	**1**	**0**	**0**	**0**	**0**	**0**	**0**	**0**	**0**	**0**	**0**	**0**
**Enterobacter cloacae/bla OXA-143**																			
Não Detectável	0	0	0	0	0	0	0	0	0	0	0	0	0	0	0	0	0	0	0
**Subtotal**	**0**	**0**	**0**	**0**	**0**	**0**	**0**	**0**	**0**	**0**	**0**	**0**	**0**	**0**	**0**	**0**	**0**	**0**	**0**
**Enterobacter cloacae/bla OXA-23**																			
Não Detectável	0	0	0	0	0	0	0	0	0	0	0	0	0	0	0	0	0	0	0
**Subtotal**	**0**	**0**	**0**	**0**	**0**	**0**	**0**	**0**	**0**	**0**	**0**	**0**	**0**	**0**	**0**	**0**	**0**	**0**	**0**
**Enterobacter cloacae/bla OXA-48**																			
Não Detectável	0	0	0	0	0	0	0	0	0	0	0	0	0	0	0	0	0	0	0
**Subtotal**	**0**	**0**	**0**	**0**	**0**	**0**	**0**	**0**	**0**	**0**	**0**	**0**	**0**	**0**	**0**	**0**	**0**	**0**	**0**
**Enterobacter cloacae/bla OXA-51**																			
Não Detectável	0	0	0	0	0	0	0	0	0	0	0	0	0	0	0	0	0	0	0
**Subtotal**	**0**	**0**	**0**	**0**	**0**	**0**	**0**	**0**	**0**	**0**	**0**	**0**	**0**	**0**	**0**	**0**	**0**	**0**	**0**
**Enterobacter cloacae/bla SPM**																			
Não Detectável	0	0	0	0	0	0	0	0	0	0	0	0	0	0	0	0	0	0	0
**Subtotal**	**0**	**0**	**0**	**0**	**0**	**0**	**0**	**0**	**0**	**0**	**0**	**0**	**0**	**0**	**0**	**0**	**0**	**0**	**0**
**Enterobacter cloacae/blaVIM**																			
Não Detectável	0	0	0	0	0	0	0	0	0	0	0	0	0	0	0	0	0	0	0
**Subtotal**	**0**	**0**	**0**	**0**	**0**	**0**	**0**	**0**	**0**	**0**	**0**	**0**	**0**	**0**	**0**	**0**	**0**	**0**	**0**
**Enterobacter cloacae/mcr-1**																			
Não Detectável	0	0	0	0	0	0	0	0	0	0	0	0	0	0	0	0	0	0	0
**Subtotal**	**0**	**0**	**0**	**0**	**0**	**0**	**0**	**0**	**0**	**0**	**0**	**0**	**0**	**0**	**0**	**0**	**0**	**0**	**0**
**Enterobacter sp./bla KPC**																			
Não Detectável	0	0	0	0	0	0	0	0	0	0	0	0	0	0	0	0	0	1	0
**Subtotal**	**0**	**0**	**0**	**0**	**0**	**0**	**0**	**0**	**0**	**0**	**0**	**0**	**0**	**0**	**0**	**0**	**0**	**1**	**0**
**Enterococcus faecium/vanA**																			
Detectável	0	0	0	0	0	0	0	0	0	0	0	0	0	0	0	0	0	0	0
**Subtotal**	**0**	**0**	**0**	**0**	**0**	**0**	**0**	**0**	**0**	**0**	**0**	**0**	**0**	**0**	**0**	**0**	**0**	**0**	**0**
**Enterococcus faecium/vanB**																			
Não Detectável	0	0	0	0	0	0	0	0	0	0	0	0	0	0	0	0	0	0	0
**Subtotal**	**0**	**0**	**0**	**0**	**0**	**0**	**0**	**0**	**0**	**0**	**0**	**0**	**0**	**0**	**0**	**0**	**0**	**0**	**0**
**Escherichia coli/bla IMP**																			
Não Detectável	0	0	0	0	0	0	0	0	0	0	0	2	0	0	0	0	0	0	0
**Subtotal**	**0**	**0**	**0**	**0**	**0**	**0**	**0**	**0**	**0**	**0**	**0**	**2**	**0**	**0**	**0**	**0**	**0**	**0**	**0**

Microrganismo /Gene Pesquisado	Jan/2018	Fev/2018	Mar/2018	Abr/2018	Maio/2018	Jun/2018	Jul/2018	Ago/2018	Set/2018	Out/2018	Nov/2018	Dez/2018	Jan/2019	Fev/2019	Mar/2019	Abr/2019	Maio/2019	Jun/2019	Jul/2019
**Escherichia coli/bla KPC**																			
Detectável	0	0	0	0	1	1	0	0	1	0	0	2	1	0	2	0	2	0	0
Não Detectável	0	0	0	0	0	0	0	0	0	2	0	0	0	1	0	0	0	0	1
**Subtotal**	**0**	**0**	**0**	**0**	**1**	**1**	**0**	**0**	**1**	**2**	**0**	**2**	**1**	**1**	**2**	**0**	**2**	**0**	**1**
**Escherichia coli/bla NDM**																			
Detectável	0	0	0	0	0	0	0	0	0	0	0	0	0	0	0	0	0	0	0
Não Detectável	0	0	0	0	0	0	0	0	0	2	0	2	0	1	0	0	0	0	0
**Subtotal**	**0**	**0**	**0**	**0**	**0**	**0**	**0**	**0**	**0**	**2**	**0**	**2**	**0**	**1**	**0**	**0**	**0**	**0**	**0**
**Escherichia coli/bla OXA-143**																			
Não Detectável	0	0	0	0	0	0	0	0	0	0	0	0	0	0	0	0	0	0	0
**Subtotal**	**0**	**0**	**0**	**0**	**0**	**0**	**0**	**0**	**0**	**0**	**0**	**0**	**0**	**0**	**0**	**0**	**0**	**0**	**0**
**Escherichia coli/bla OXA-23**																			
Não Detectável	0	0	0	0	0	0	0	0	0	0	0	0	0	0	0	0	0	0	0
**Subtotal**	**0**	**0**	**0**	**0**	**0**	**0**	**0**	**0**	**0**	**0**	**0**	**0**	**0**	**0**	**0**	**0**	**0**	**0**	**0**
**Escherichia coli/bla OXA-48**																			
Detectável	0	0	0	0	0	0	0	0	0	0	0	0	0	0	0	0	0	0	0
Não Detectável	0	0	0	0	0	0	0	0	0	0	0	0	0	0	0	0	0	0	0
**Subtotal**	**0**	**0**	**0**	**0**	**0**	**0**	**0**	**0**	**0**	**0**	**0**	**0**	**0**	**0**	**0**	**0**	**0**	**0**	**0**
**Escherichia coli/bla OXA-51**																			
Detectável	0	0	0	0	0	0	0	0	0	0	0	0	0	0	0	0	0	0	0
Não Detectável	0	0	0	0	0	0	0	0	0	0	0	0	0	0	0	0	0	0	0
**Subtotal**	**0**	**0**	**0**	**0**	**0**	**0**	**0**	**0**	**0**	**0**	**0**	**0**	**0**	**0**	**0**	**0**	**0**	**0**	**0**
**Escherichia coli/bla OXA-58**																			
Não Detectável	0	0	0	0	0	0	0	0	0	0	0	0	0	0	0	0	0	0	0
**Subtotal**	**0**	**0**	**0**	**0**	**0**	**0**	**0**	**0**	**0**	**0**	**0**	**0**	**0**	**0**	**0**	**0**	**0**	**0**	**0**
**Escherichia coli/bla SPM**																			
Não Detectável	0	0	0	0	0	0	0	0	0	0	0	0	0	0	0	0	0	0	0
**Subtotal**	**0**	**0**	**0**	**0**	**0**	**0**	**0**	**0**	**0**	**0**	**0**	**0**	**0**	**0**	**0**	**0**	**0**	**0**	**0**
**Escherichia coli/blaVIM**																			
Não Detectável	0	0	0	0	0	0	0	0	0	0	0	0	0	0	0	0	0	0	0
**Subtotal**	**0**	**0**	**0**	**0**	**0**	**0**	**0**	**0**	**0**	**0**	**0**	**0**	**0**	**0**	**0**	**0**	**0**	**0**	**0**
**Escherichia coli cepas verotoxigênicas como O103/outros**																			
Detectável	0	0	0	0	0	0	0	0	0	0	0	0	0	0	0	0	0	0	0
**Subtotal**	**0**	**0**	**0**	**0**	**0**	**0**	**0**	**0**	**0**	**0**	**0**	**0**	**0**	**0**	**0**	**0**	**0**	**0**	**0**
**Escherichia coli enteroinvasora/outros**																			
Detectável	0	0	0	0	0	0	0	0	0	0	0	0	0	0	0	0	0	0	0
**Subtotal**	**0**	**0**	**0**	**0**	**0**	**0**	**0**	**0**	**0**	**0**	**0**	**0**	**0**	**0**	**0**	**0**	**0**	**0**	**0**
**Escherichia coli enterotoxigênica/bla KPC**																			
Não Detectável	0	0	0	0	0	0	0	0	0	0	0	0	0	0	0	0	0	0	1
**Subtotal**	**0**	**0**	**0**	**0**	**0**	**0**	**0**	**0**	**0**	**0**	**0**	**0**	**0**	**0**	**0**	**0**	**0**	**0**	**1**
**Escherichia coli enterotoxigênica/outros**																			
Detectável	0	0	0	0	0	0	0	0	0	0	0	0	0	0	0	0	0	0	1
**Subtotal**	**0**	**0**	**0**	**0**	**0**	**0**	**0**	**0**	**0**	**0**	**0**	**0**	**0**	**0**	**0**	**0**	**0**	**0**	**1**
**Escherichia coli/ipa H**																			
Não Detectável	0	0	0	0	0	0	0	0	0	0	0	0	0	0	0	0	0	0	0
**Subtotal**	**0**	**0**	**0**	**0**	**0**	**0**	**0**	**0**	**0**	**0**	**0**	**0**	**0**	**0**	**0**	**0**	**0**	**0**	**0**
**Escherichia coli/KPC**																			
Detectável	0	0	0	0	0	0	0	0	0	0	0	0	0	1	0	0	0	0	0
Não Detectável	0	0	0	0	0	0	0	0	0	0	0	0	0	0	0	0	0	0	0
**Subtotal**	**0**	**0**	**0**	**0**	**0**	**0**	**0**	**0**	**0**	**0**	**0**	**0**	**0**	**1**	**0**	**0**	**0**	**0**	**0**
**Escherichia coli/mcr-1**																			
Não Detectável	0	0	0	0	0	0	0	0	0	0	0	0	0	0	0	0	0	0	0
**Subtotal**	**0**	**0**	**0**	**0**	**0**	**0**	**0**	**0**	**0**	**0**	**0**	**0**	**0**	**0**	**0**	**0**	**0**	**0**	**0**
**Escherichia coli/outros**																			

Microrganismo /Gene Pesquisado	Jan/2018	Fev/2018	Mar/2018	Abr/2018	Maio/2018	Jun/2018	Jul/2018	Ago/2018	Set/2018	Out/2018	Nov/2018	Dez/2018	Jan/2019	Fev/2019	Mar/2019	Abr/2019	Maio/2019	Jun/2019	Jul/2019
Detectável	0	0	0	0	0	0	0	0	0	0	0	0	0	0	0	0	0	0	0
Não Detectável	0	0	0	0	0	0	0	0	0	0	0	0	0	0	0	0	0	0	0
**Subtotal**	**0**	**0**	**0**	**0**	**0**	**0**	**0**	**0**	**0**	**0**	**0**	**0**	**0**	**0**	**0**	**0**	**0**	**0**	**0**
**Klebsiella aerogenes/bla IMP**																			
Não Detectável	0	0	0	0	0	0	0	0	0	0	0	0	0	0	0	0	0	0	0
**Subtotal**	**0**	**0**	**0**	**0**	**0**	**0**	**0**	**0**	**0**	**0**	**0**	**0**	**0**	**0**	**0**	**0**	**0**	**0**	**0**
**Klebsiella aerogenes/bla KPC**																			
Não Detectável	0	0	0	0	0	0	0	0	0	0	0	0	0	0	0	0	0	0	0
**Subtotal**	**0**	**0**	**0**	**0**	**0**	**0**	**0**	**0**	**0**	**0**	**0**	**0**	**0**	**0**	**0**	**0**	**0**	**0**	**0**
**Klebsiella aerogenes/bla NDM**																			
Não Detectável	0	0	0	0	0	0	0	0	0	0	0	0	0	0	0	0	0	0	0
**Subtotal**	**0**	**0**	**0**	**0**	**0**	**0**	**0**	**0**	**0**	**0**	**0**	**0**	**0**	**0**	**0**	**0**	**0**	**0**	**0**
**Klebsiella aerogenes/bla OXA-48**																			
Não Detectável	0	0	0	0	0	0	0	0	0	0	0	0	0	0	0	0	0	0	0
**Subtotal**	**0**	**0**	**0**	**0**	**0**	**0**	**0**	**0**	**0**	**0**	**0**	**0**	**0**	**0**	**0**	**0**	**0**	**0**	**0**
**Klebsiella aerogenes/bla SPM**																			
Não Detectável	0	0	0	0	0	0	0	0	0	0	0	0	0	0	0	0	0	0	0
**Subtotal**	**0**	**0**	**0**	**0**	**0**	**0**	**0**	**0**	**0**	**0**	**0**	**0**	**0**	**0**	**0**	**0**	**0**	**0**	**0**
**Klebsiella aerogenes/blaVIM**																			
Não Detectável	0	0	0	0	0	0	0	0	0	0	0	0	0	0	0	0	0	0	0
**Subtotal**	**0**	**0**	**0**	**0**	**0**	**0**	**0**	**0**	**0**	**0**	**0**	**0**	**0**	**0**	**0**	**0**	**0**	**0**	**0**
**Klebsiella aerogenes/mcr-1**																			
Não Detectável	0	0	0	0	0	0	0	0	0	0	0	0	0	0	0	0	0	0	0
**Subtotal**	**0**	**0**	**0**	**0**	**0**	**0**	**0**	**0**	**0**	**0**	**0**	**0**	**0**	**0**	**0**	**0**	**0**	**0**	**0**
**Klebsiella oxytoca/bla IMP**																			
Não Detectável	0	0	0	0	0	0	0	0	0	0	0	1	0	0	0	0	0	0	0
**Subtotal**	**0**	**0**	**0**	**0**	**0**	**0**	**0**	**0**	**0**	**0**	**0**	**1**	**0**	**0**	**0**	**0**	**0**	**0**	**0**
**Klebsiella oxytoca/bla KPC**																			
Não Detectável	0	0	0	0	0	0	0	0	0	1	0	1	0	1	0	0	0	0	0
**Subtotal**	**0**	**0**	**0**	**0**	**0**	**0**	**0**	**0**	**0**	**1**	**0**	**1**	**0**	**1**	**0**	**0**	**0**	**0**	**0**
**Klebsiella oxytoca/bla NDM**																			
Não Detectável	0	0	0	0	0	0	0	0	0	1	0	1	0	0	0	0	0	0	0
**Subtotal**	**0**	**0**	**0**	**0**	**0**	**0**	**0**	**0**	**0**	**1**	**0**	**1**	**0**	**0**	**0**	**0**	**0**	**0**	**0**
**Klebsiella oxytoca/bla OXA-23**																			
Não Detectável	0	0	0	0	0	0	0	0	0	0	0	0	0	0	1	0	0	0	0
**Subtotal**	**0**	**0**	**0**	**0**	**0**	**0**	**0**	**0**	**0**	**0**	**0**	**0**	**0**	**0**	**1**	**0**	**0**	**0**	**0**
**Klebsiella oxytoca/bla OXA-48**																			
Não Detectável	0	0	0	0	0	0	0	0	0	0	0	0	0	0	0	0	0	0	0
**Subtotal**	**0**	**0**	**0**	**0**	**0**	**0**	**0**	**0**	**0**	**0**	**0**	**0**	**0**	**0**	**0**	**0**	**0**	**0**	**0**
**Klebsiella oxytoca/bla OXA-51**																			
Não Detectável	0	0	0	0	0	0	0	0	0	0	0	0	0	0	1	0	0	0	0
**Subtotal**	**0**	**0**	**0**	**0**	**0**	**0**	**0**	**0**	**0**	**0**	**0**	**0**	**0**	**0**	**1**	**0**	**0**	**0**	**0**
**Klebsiella oxytoca/bla OXA-58**																			
Não Detectável	0	0	0	0	0	0	0	0	0	0	0	0	0	0	0	0	0	0	0
**Subtotal**	**0**	**0**	**0**	**0**	**0**	**0**	**0**	**0**	**0**	**0**	**0**	**0**	**0**	**0**	**0**	**0**	**0**	**0**	**0**
**Klebsiella oxytoca/bla SPM**																			
Não Detectável	0	0	0	0	0	0	0	0	0	0	0	0	0	0	0	0	0	0	0
**Subtotal**	**0**	**0**	**0**	**0**	**0**	**0**	**0**	**0**	**0**	**0**	**0**	**0**	**0**	**0**	**0**	**0**	**0**	**0**	**0**
**Klebsiella oxytoca/blaVIM**																			
Não Detectável	0	0	0	0	0	0	0	0	0	0	0	0	0	0	0	0	0	0	0
**Subtotal**	**0**	**0**	**0**	**0**	**0**	**0**	**0**	**0**	**0**	**0**	**0**	**0**	**0**	**0**	**0**	**0**	**0**	**0**	**0**
**Klebsiella ozaenae/bla IMP**																			
Não Detectável	0	0	0	0	0	0	0	0	0	0	0	0	0	0	0	0	0	0	0
**Subtotal**	**0**	**0**	**0**	**0**	**0**	**0**	**0**	**0**	**0**	**0**	**0**	**0**	**0**	**0**	**0**	**0**	**0**	**0**	**0**

Microrganismo /Gene Pesquisado	Jan/2018	Fev/2018	Mar/2018	Abr/2018	Maio/2018	Jun/2018	Jul/2018	Ago/2018	Set/2018	Out/2018	Nov/2018	Dez/2018	Jan/2019	Fev/2019	Mar/2019	Abr/2019	Maio/2019	Jun/2019	Jul/2019
**Klebsiella ozaenae/bla KPC**																			
Detectável	0	0	0	0	0	0	0	0	0	0	0	0	0	0	0	0	0	0	0
Não Detectável	0	0	0	0	0	0	0	0	0	0	0	0	0	0	0	0	0	0	0
**Subtotal**	**0**	**0**	**0**	**0**	**0**	**0**	**0**	**0**	**0**	**0**	**0**	**0**	**0**	**0**	**0**	**0**	**0**	**0**	**0**
**Klebsiella ozaenae/bla NDM**																			
Não Detectável	0	0	0	0	0	0	0	0	0	0	0	0	0	0	0	0	0	0	0
**Subtotal**	**0**	**0**	**0**	**0**	**0**	**0**	**0**	**0**	**0**	**0**	**0**	**0**	**0**	**0**	**0**	**0**	**0**	**0**	**0**
**Klebsiella ozaenae/bla OXA-48**																			
Não Detectável	0	0	0	0	0	0	0	0	0	0	0	0	0	0	0	0	0	0	0
**Subtotal**	**0**	**0**	**0**	**0**	**0**	**0**	**0**	**0**	**0**	**0**	**0**	**0**	**0**	**0**	**0**	**0**	**0**	**0**	**0**
**Klebsiella ozaenae/bla SPM**																			
Não Detectável	0	0	0	0	0	0	0	0	0	0	0	0	0	0	0	0	0	0	0
**Subtotal**	**0**	**0**	**0**	**0**	**0**	**0**	**0**	**0**	**0**	**0**	**0**	**0**	**0**	**0**	**0**	**0**	**0**	**0**	**0**
**Klebsiella ozaenae/blaVIM**																			
Não Detectável	0	0	0	0	0	0	0	0	0	0	0	0	0	0	0	0	0	0	0
**Subtotal**	**0**	**0**	**0**	**0**	**0**	**0**	**0**	**0**	**0**	**0**	**0**	**0**	**0**	**0**	**0**	**0**	**0**	**0**	**0**
**Klebsiella pneumoniae/bla IMP**																			
Detectável	0	0	0	0	0	0	0	0	0	0	0	0	0	0	0	0	0	0	0
Não Detectável	0	0	0	1	0	0	0	0	0	0	0	5	2	0	0	0	0	1	1
**Subtotal**	**0**	**0**	**0**	**1**	**0**	**0**	**0**	**0**	**0**	**0**	**0**	**5**	**2**	**0**	**0**	**0**	**0**	**1**	**1**
**Klebsiella pneumoniae/bla KPC**																			
Detectável	0	0	2	1	1	2	0	18	9	3	2	7	11	1	18	3	1	1	8
Inconclusivo	0	0	0	0	0	0	0	0	0	0	0	0	0	0	0	0	0	0	0
Não Detectável	0	0	0	0	0	4	0	3	12	0	0	7	2	2	2	3	4	1	1
**Subtotal**	**0**	**0**	**2**	**1**	**1**	**6**	**0**	**21**	**21**	**3**	**2**	**14**	**13**	**3**	**20**	**6**	**5**	**2**	**9**
**Klebsiella pneumoniae/bla NDM**																			
Detectável	0	0	0	0	0	0	0	0	0	0	0	0	0	0	0	1	0	0	0
Não Detectável	0	0	1	1	0	1	0	0	1	0	0	11	11	0	0	1	0	1	2
**Subtotal**	**0**	**0**	**1**	**1**	**0**	**1**	**0**	**0**	**1**	**0**	**0**	**11**	**11**	**0**	**0**	**2**	**0**	**1**	**2**
**Klebsiella pneumoniae/bla OXA-143**																			
Não Detectável	0	0	0	0	0	0	0	0	0	0	0	0	0	0	0	0	0	0	0
**Subtotal**	**0**	**0**	**0**	**0**	**0**	**0**	**0**	**0**	**0**	**0**	**0**	**0**	**0**	**0**	**0**	**0**	**0**	**0**	**0**
**Klebsiella pneumoniae/bla OXA-23**																			
Detectável	0	0	0	0	0	0	0	0	0	0	0	0	0	0	0	0	0	0	0
Não Detectável	0	0	0	0	0	0	0	0	0	0	0	0	0	0	0	0	0	0	0
**Subtotal**	**0**	**0**	**0**	**0**	**0**	**0**	**0**	**0**	**0**	**0**	**0**	**0**	**0**	**0**	**0**	**0**	**0**	**0**	**0**
**Klebsiella pneumoniae/bla OXA-48**																			
Detectado traços	0	0	0	0	0	0	0	0	0	0	0	0	0	0	0	0	0	0	0
Detectável	0	0	0	0	0	0	0	0	0	0	0	0	0	0	0	0	0	0	0
Não Detectável	0	0	0	0	0	1	0	0	0	0	0	6	4	0	0	4	0	0	1
**Subtotal**	**0**	**0**	**0**	**0**	**0**	**1**	**0**	**0**	**0**	**0**	**0**	**6**	**4**	**0**	**0**	**4**	**0**	**0**	**1**
**Klebsiella pneumoniae/bla OXA-51**																			
Detectável	0	0	0	0	0	0	0	0	0	0	0	0	0	0	0	0	0	0	0
Não Detectável	0	0	0	0	0	0	0	0	0	0	0	0	0	0	0	0	0	0	0
**Subtotal**	**0**	**0**	**0**	**0**	**0**	**0**	**0**	**0**	**0**	**0**	**0**	**0**	**0**	**0**	**0**	**0**	**0**	**0**	**0**
**Klebsiella pneumoniae/bla OXA-58**																			
Detectável	0	0	0	0	0	0	0	0	0	0	0	0	0	0	0	0	0	0	0
Não Detectável	0	0	0	0	0	0	0	0	0	0	0	0	0	0	0	0	0	0	0
**Subtotal**	**0**	**0**	**0**	**0**	**0**	**0**	**0**	**0**	**0**	**0**	**0**	**0**	**0**	**0**	**0**	**0**	**0**	**0**	**0**
**Klebsiella pneumoniae/bla SPM**																			
Detectável	0	0	0	0	0	0	0	0	0	0	0	0	0	0	0	0	0	0	0
Não Detectável	0	0	0	0	0	0	0	0	0	0	0	0	2	0	0	0	0	0	0
**Subtotal**	**0**	**0**	**0**	**0**	**0**	**0**	**0**	**0**	**0**	**0**	**0**	**0**	**2**	**0**	**0**	**0**	**0**	**0**	**0**
**Klebsiella pneumoniae/blaVIM**																			

Microrganismo /Gene Pesquisado	Jan/2018	Fev/2018	Mar/2018	Abr/2018	Maio/2018	Jun/2018	Jul/2018	Ago/2018	Set/2018	Out/2018	Nov/2018	Dez/2018	Jan/2019	Fev/2019	Mar/2019	Abr/2019	Maio/2019	Jun/2019	Jul/2019
Não Detectável	0	0	1	0	0	0	0	0	0	0	0	0	3	0	0	0	0	0	0
**Subtotal**	**0**	**0**	**1**	**0**	**0**	**0**	**0**	**0**	**0**	**0**	**0**	**0**	**3**	**0**	**0**	**0**	**0**	**0**	**0**
**Klebsiella pneumoniae/IMP**																			
Não Detectável	0	0	0	0	0	0	0	0	0	0	0	0	0	0	0	0	0	0	0
**Subtotal**	**0**	**0**	**0**	**0**	**0**	**0**	**0**	**0**	**0**	**0**	**0**	**0**	**0**	**0**	**0**	**0**	**0**	**0**	**0**
**Klebsiella pneumoniae/KPC**																			
Detectável	0	0	0	0	0	0	0	7	0	0	0	0	0	1	0	0	0	0	0
Não Detectável	0	0	0	0	0	0	0	1	0	0	0	0	0	0	0	0	0	0	0
**Subtotal**	**0**	**0**	**0**	**0**	**0**	**0**	**0**	**8**	**0**	**0**	**0**	**0**	**0**	**1**	**0**	**0**	**0**	**0**	**0**
**Klebsiella pneumoniae/mcr-1**																			
Não Detectável	0	0	0	0	0	0	0	0	0	0	0	0	0	0	0	0	0	0	0
**Subtotal**	**0**	**0**	**0**	**0**	**0**	**0**	**0**	**0**	**0**	**0**	**0**	**0**	**0**	**0**	**0**	**0**	**0**	**0**	**0**
**Klebsiella pneumoniae/NDM**																			
Não Detectável	0	0	0	0	0	0	0	1	0	0	0	0	0	0	1	0	0	0	0
**Subtotal**	**0**	**0**	**0**	**0**	**0**	**0**	**0**	**1**	**0**	**0**	**0**	**0**	**0**	**0**	**1**	**0**	**0**	**0**	**0**
**Klebsiella pneumoniae/outros**																			
Não Detectável	0	0	0	0	0	0	0	0	0	0	0	0	0	0	0	0	0	0	0
**Subtotal**	**0**	**0**	**0**	**0**	**0**	**0**	**0**	**0**	**0**	**0**	**0**	**0**	**0**	**0**	**0**	**0**	**0**	**0**	**0**
**Klebsiella pneumoniae/SPM**																			
Não Detectável	0	0	0	0	0	0	0	0	0	0	0	0	0	0	0	0	0	0	0
**Subtotal**	**0**	**0**	**0**	**0**	**0**	**0**	**0**	**0**	**0**	**0**	**0**	**0**	**0**	**0**	**0**	**0**	**0**	**0**	**0**
**Klebsiella pneumoniae subsp. pneumoniae/bla KPC**																			
Detectável	0	0	0	0	0	0	0	0	0	2	0	0	0	0	0	0	0	1	0
Não Detectável	0	0	0	0	0	0	0	0	0	0	0	0	0	0	3	0	0	1	0
**Subtotal**	**0**	**0**	**0**	**0**	**0**	**0**	**0**	**0**	**0**	**2**	**0**	**0**	**0**	**0**	**3**	**0**	**0**	**2**	**0**
**Klebsiella pneumoniae/VIM**																			
Não Detectável	0	0	0	0	0	0	0	0	0	0	0	0	0	0	0	0	0	0	0
**Subtotal**	**0**	**0**	**0**	**0**	**0**	**0**	**0**	**0**	**0**	**0**	**0**	**0**	**0**	**0**	**0**	**0**	**0**	**0**	**0**
**Klebsiella sp./bla IMP**																			
Não Detectável	0	0	0	0	0	0	0	0	0	0	0	0	0	0	0	0	0	0	0
**Subtotal**	**0**	**0**	**0**	**0**	**0**	**0**	**0**	**0**	**0**	**0**	**0**	**0**	**0**	**0**	**0**	**0**	**0**	**0**	**0**
**Klebsiella sp./bla KPC**																			
Detectável	0	0	0	0	0	0	0	0	0	0	0	0	2	0	1	0	1	0	1
Não Detectável	0	0	0	0	0	0	0	0	0	1	0	0	0	0	0	0	1	0	0
**Subtotal**	**0**	**0**	**0**	**0**	**0**	**0**	**0**	**0**	**0**	**1**	**0**	**0**	**2**	**0**	**1**	**0**	**2**	**0**	**1**
**Klebsiella sp./bla NDM**																			
Não Detectável	0	0	0	0	0	0	0	0	0	1	0	0	2	0	0	0	0	0	0
**Subtotal**	**0**	**0**	**0**	**0**	**0**	**0**	**0**	**0**	**0**	**1**	**0**	**0**	**2**	**0**	**0**	**0**	**0**	**0**	**0**
**Klebsiella sp./bla OXA-48**																			
Não Detectável	0	0	0	0	0	0	0	0	0	0	0	0	2	0	0	0	0	0	0
**Subtotal**	**0**	**0**	**0**	**0**	**0**	**0**	**0**	**0**	**0**	**0**	**0**	**0**	**2**	**0**	**0**	**0**	**0**	**0**	**0**
**Klebsiella sp./bla SPM**																			
Não Detectável	0	0	0	0	0	0	0	0	0	0	0	0	0	0	0	0	0	0	0
**Subtotal**	**0**	**0**	**0**	**0**	**0**	**0**	**0**	**0**	**0**	**0**	**0**	**0**	**0**	**0**	**0**	**0**	**0**	**0**	**0**
**Klebsiella sp./blaVIM**																			
Não Detectável	0	0	0	0	0	0	0	0	0	0	0	0	0	0	0	0	0	0	0
**Subtotal**	**0**	**0**	**0**	**0**	**0**	**0**	**0**	**0**	**0**	**0**	**0**	**0**	**0**	**0**	**0**	**0**	**0**	**0**	**0**
**Klebsiella sp./KPC**																			
Detectável	0	0	0	0	0	0	0	0	0	1	0	0	0	1	0	0	0	0	0
**Subtotal**	**0**	**0**	**0**	**0**	**0**	**0**	**0**	**0**	**0**	**1**	**0**	**0**	**0**	**1**	**0**	**0**	**0**	**0**	**0**
**Morganella morganii/bla IMP**																			
Não Detectável	0	0	0	0	0	0	0	0	0	0	0	0	0	0	0	0	0	0	0
**Subtotal**	**0**	**0**	**0**	**0**	**0**	**0**	**0**	**0**	**0**	**0**	**0**	**0**	**0**	**0**	**0**	**0**	**0**	**0**	**0**
**Morganella morganii/bla KPC**																			

Microrganismo /Gene Pesquisado	Jan/2018	Fev/2018	Mar/2018	Abr/2018	Maio/2018	Jun/2018	Jul/2018	Ago/2018	Set/2018	Out/2018	Nov/2018	Dez/2018	Jan/2019	Fev/2019	Mar/2019	Abr/2019	Maio/2019	Jun/2019	Jul/2019
Detectável	0	0	0	0	0	0	0	0	0	0	0	0	0	0	0	0	0	0	0
Não Detectável	0	0	0	0	0	0	0	0	0	0	0	0	0	0	0	0	0	0	0
**Subtotal**	**0**	**0**	**0**	**0**	**0**	**0**	**0**	**0**	**0**	**0**	**0**	**0**	**0**	**0**	**0**	**0**	**0**	**0**	**0**
**Morganella morganii/bla NDM**																			
Não Detectável	0	0	0	0	0	0	0	0	0	0	0	0	0	0	0	0	0	0	0
**Subtotal**	**0**	**0**	**0**	**0**	**0**	**0**	**0**	**0**	**0**	**0**	**0**	**0**	**0**	**0**	**0**	**0**	**0**	**0**	**0**
**Morganella morganii/bla OXA-23**																			
Não Detectável	0	0	0	0	0	0	0	0	0	0	0	0	0	0	0	0	0	0	0
**Subtotal**	**0**	**0**	**0**	**0**	**0**	**0**	**0**	**0**	**0**	**0**	**0**	**0**	**0**	**0**	**0**	**0**	**0**	**0**	**0**
**Morganella morganii/bla OXA-48**																			
Detectável	0	0	0	0	0	0	0	0	0	0	0	0	0	0	0	0	0	0	0
Não Detectável	0	0	0	0	0	0	0	0	0	0	0	0	0	0	0	0	0	0	0
**Subtotal**	**0**	**0**	**0**	**0**	**0**	**0**	**0**	**0**	**0**	**0**	**0**	**0**	**0**	**0**	**0**	**0**	**0**	**0**	**0**
**Morganella morganii/bla OXA-51**																			
Não Detectável	0	0	0	0	0	0	0	0	0	0	0	0	0	0	0	0	0	0	0
**Subtotal**	**0**	**0**	**0**	**0**	**0**	**0**	**0**	**0**	**0**	**0**	**0**	**0**	**0**	**0**	**0**	**0**	**0**	**0**	**0**
**Morganella morganii/bla OXA-58**																			
Não Detectável	0	0	0	0	0	0	0	0	0	0	0	0	0	0	0	0	0	0	0
**Subtotal**	**0**	**0**	**0**	**0**	**0**	**0**	**0**	**0**	**0**	**0**	**0**	**0**	**0**	**0**	**0**	**0**	**0**	**0**	**0**
**Morganella morganii/bla SPM**																			
Inconclusivo	0	0	0	0	0	0	0	0	0	0	0	0	0	0	0	0	0	0	0
Não Detectável	0	0	0	0	0	0	0	0	0	0	0	0	0	0	0	0	0	0	0
**Subtotal**	**0**	**0**	**0**	**0**	**0**	**0**	**0**	**0**	**0**	**0**	**0**	**0**	**0**	**0**	**0**	**0**	**0**	**0**	**0**
**Morganella morganii/blaVIM**																			
Não Detectável	0	0	0	0	0	0	0	0	0	0	0	0	0	0	0	0	0	0	0
**Subtotal**	**0**	**0**	**0**	**0**	**0**	**0**	**0**	**0**	**0**	**0**	**0**	**0**	**0**	**0**	**0**	**0**	**0**	**0**	**0**
**Morganella morganii/KPC**																			
Detectável	0	0	0	0	0	0	0	0	0	1	0	0	0	0	0	0	0	0	0
Não Detectável	0	0	0	0	0	0	0	0	0	0	0	0	0	0	0	0	0	0	0
**Subtotal**	**0**	**0**	**0**	**0**	**0**	**0**	**0**	**0**	**0**	**1**	**0**	**0**	**0**	**0**	**0**	**0**	**0**	**0**	**0**
**Morganella morganii/mcr-1**																			
Não Detectável	0	0	0	0	0	0	0	0	0	0	0	0	0	0	0	0	0	0	0
**Subtotal**	**0**	**0**	**0**	**0**	**0**	**0**	**0**	**0**	**0**	**0**	**0**	**0**	**0**	**0**	**0**	**0**	**0**	**0**	**0**
**Morganella morganii/SPM**																			
Não Detectável	0	0	0	0	0	0	0	0	0	0	0	0	0	0	0	0	0	0	0
**Subtotal**	**0**	**0**	**0**	**0**	**0**	**0**	**0**	**0**	**0**	**0**	**0**	**0**	**0**	**0**	**0**	**0**	**0**	**0**	**0**
**Pantoea agglomerans/bla KPC**																			
Não Detectável	0	0	0	0	0	0	0	0	0	0	0	0	0	0	0	0	0	0	0
**Subtotal**	**0**	**0**	**0**	**0**	**0**	**0**	**0**	**0**	**0**	**0**	**0**	**0**	**0**	**0**	**0**	**0**	**0**	**0**	**0**
**Pantoea agglomerans/bla NDM**																			
Detectável	0	0	0	0	0	0	0	0	0	0	0	0	0	0	0	0	0	0	0
**Subtotal**	**0**	**0**	**0**	**0**	**0**	**0**	**0**	**0**	**0**	**0**	**0**	**0**	**0**	**0**	**0**	**0**	**0**	**0**	**0**
**Pantoea agglomerans/bla OXA-48**																			
Não Detectável	0	0	0	0	0	0	0	0	0	0	0	0	0	0	0	0	0	0	0
**Subtotal**	**0**	**0**	**0**	**0**	**0**	**0**	**0**	**0**	**0**	**0**	**0**	**0**	**0**	**0**	**0**	**0**	**0**	**0**	**0**
**Pantoea agglomerans/mcr-1**																			
Não Detectável	0	0	0	0	0	0	0	0	0	0	0	0	0	0	0	0	0	0	0
**Subtotal**	**0**	**0**	**0**	**0**	**0**	**0**	**0**	**0**	**0**	**0**	**0**	**0**	**0**	**0**	**0**	**0**	**0**	**0**	**0**
**Proteus mirabilis/bla IMP**																			
Não Detectável	0	0	0	0	0	1	0	0	0	0	0	1	0	0	0	0	0	0	1
**Subtotal**	**0**	**0**	**0**	**0**	**0**	**1**	**0**	**0**	**0**	**0**	**0**	**1**	**0**	**0**	**0**	**0**	**0**	**0**	**1**
**Proteus mirabilis/bla KPC**																			
Inconclusivo	0	0	0	0	0	0	0	0	0	0	0	0	0	0	0	0	0	0	0
Não Detectável	0	0	0	0	0	1	0	0	0	0	0	1	0	0	0	0	0	0	1

Microrganismo /Gene Pesquisado	Jan/2018	Fev/2018	Mar/2018	Abr/2018	Maio/2018	Jun/2018	Jul/2018	Ago/2018	Set/2018	Out/2018	Nov/2018	Dez/2018	Jan/2019	Fev/2019	Mar/2019	Abr/2019	Maio/2019	Jun/2019	Jul/2019
**Subtotal**	**0**	**0**	**0**	**0**	**0**	**1**	**0**	**0**	**0**	**0**	**0**	**1**	**0**	**0**	**0**	**0**	**0**	**0**	**1**
**Proteus mirabilis/bla NDM**																			
Não Detectável	0	0	0	0	0	0	0	0	1	0	0	1	0	0	1	0	0	0	1
**Subtotal**	**0**	**0**	**0**	**0**	**0**	**0**	**0**	**0**	**1**	**0**	**0**	**1**	**0**	**0**	**1**	**0**	**0**	**0**	**1**
**Proteus mirabilis/bla OXA-23**																			
Não Detectável	0	0	0	0	0	0	0	0	0	0	0	0	0	0	0	0	0	0	0
**Subtotal**	**0**	**0**	**0**	**0**	**0**	**0**	**0**	**0**	**0**	**0**	**0**	**0**	**0**	**0**	**0**	**0**	**0**	**0**	**0**
**Proteus mirabilis/bla OXA-48**																			
Não Detectável	0	0	0	0	0	0	0	0	0	0	0	0	0	0	0	0	0	0	1
**Subtotal**	**0**	**0**	**0**	**0**	**0**	**0**	**0**	**0**	**0**	**0**	**0**	**0**	**0**	**0**	**0**	**0**	**0**	**0**	**1**
**Proteus mirabilis/bla OXA-51**																			
Não Detectável	0	0	0	0	0	0	0	0	0	0	0	0	0	0	0	0	0	0	0
**Subtotal**	**0**	**0**	**0**	**0**	**0**	**0**	**0**	**0**	**0**	**0**	**0**	**0**	**0**	**0**	**0**	**0**	**0**	**0**	**0**
**Proteus mirabilis/bla OXA-58**																			
Não Detectável	0	0	0	0	0	0	0	0	0	0	0	0	0	0	0	0	0	0	0
**Subtotal**	**0**	**0**	**0**	**0**	**0**	**0**	**0**	**0**	**0**	**0**	**0**	**0**	**0**	**0**	**0**	**0**	**0**	**0**	**0**
**Proteus mirabilis/bla SPM**																			
Não Detectável	0	0	0	0	0	0	0	0	0	0	0	0	0	0	0	0	0	0	0
**Subtotal**	**0**	**0**	**0**	**0**	**0**	**0**	**0**	**0**	**0**	**0**	**0**	**0**	**0**	**0**	**0**	**0**	**0**	**0**	**0**
**Proteus mirabilis/blaVIM**																			
Não Detectável	0	0	0	0	0	0	0	0	0	0	0	0	0	0	0	0	0	0	1
**Subtotal**	**0**	**0**	**0**	**0**	**0**	**0**	**0**	**0**	**0**	**0**	**0**	**0**	**0**	**0**	**0**	**0**	**0**	**0**	**1**
**Proteus mirabilis/mcr-1**																			
Não Detectável	0	0	0	0	0	0	0	0	0	0	0	0	0	0	0	0	0	0	0
**Subtotal**	**0**	**0**	**0**	**0**	**0**	**0**	**0**	**0**	**0**	**0**	**0**	**0**	**0**	**0**	**0**	**0**	**0**	**0**	**0**
**Proteus mirabilis/NDM**																			
Não Detectável	0	0	0	0	0	1	0	0	0	0	0	0	0	0	0	0	0	0	0
**Subtotal**	**0**	**0**	**0**	**0**	**0**	**1**	**0**	**0**	**0**	**0**	**0**	**0**	**0**	**0**	**0**	**0**	**0**	**0**	**0**
**Proteus penneri/bla NDM**																			
Não Detectável	0	0	0	0	0	0	0	0	0	0	0	0	0	0	1	0	0	0	0
**Subtotal**	**0**	**0**	**0**	**0**	**0**	**0**	**0**	**0**	**0**	**0**	**0**	**0**	**0**	**0**	**1**	**0**	**0**	**0**	**0**
**Proteus sp./bla IMP**																			
Não Detectável	0	0	0	0	0	0	0	0	0	0	0	0	0	0	0	0	0	0	0
**Subtotal**	**0**	**0**	**0**	**0**	**0**	**0**	**0**	**0**	**0**	**0**	**0**	**0**	**0**	**0**	**0**	**0**	**0**	**0**	**0**
**Proteus sp./bla KPC**																			
Não Detectável	0	0	0	0	0	0	0	0	0	0	0	0	0	0	0	0	0	0	0
**Subtotal**	**0**	**0**	**0**	**0**	**0**	**0**	**0**	**0**	**0**	**0**	**0**	**0**	**0**	**0**	**0**	**0**	**0**	**0**	**0**
**Proteus sp./bla NDM**																			
Não Detectável	0	0	0	0	0	0	0	0	1	0	0	0	0	0	0	0	0	0	0
**Subtotal**	**0**	**0**	**0**	**0**	**0**	**0**	**0**	**0**	**1**	**0**	**0**	**0**	**0**	**0**	**0**	**0**	**0**	**0**	**0**
**Proteus sp./bla OXA-48**																			
Não Detectável	0	0	0	0	0	0	0	0	0	0	0	0	0	0	0	0	0	0	0
**Subtotal**	**0**	**0**	**0**	**0**	**0**	**0**	**0**	**0**	**0**	**0**	**0**	**0**	**0**	**0**	**0**	**0**	**0**	**0**	**0**
**Proteus sp./bla SPM**																			
Não Detectável	0	0	0	0	0	0	0	0	0	0	0	0	0	0	0	0	0	0	0
**Subtotal**	**0**	**0**	**0**	**0**	**0**	**0**	**0**	**0**	**0**	**0**	**0**	**0**	**0**	**0**	**0**	**0**	**0**	**0**	**0**
**Proteus sp./blaVIM**																			
Não Detectável	0	0	0	0	0	0	0	0	0	0	0	0	0	0	0	0	0	0	0
**Subtotal**	**0**	**0**	**0**	**0**	**0**	**0**	**0**	**0**	**0**	**0**	**0**	**0**	**0**	**0**	**0**	**0**	**0**	**0**	**0**
**Proteus vulgaris/bla KPC**																			
Não Detectável	0	0	0	0	0	0	0	0	0	0	0	0	0	0	0	0	0	0	0
**Subtotal**	**0**	**0**	**0**	**0**	**0**	**0**	**0**	**0**	**0**	**0**	**0**	**0**	**0**	**0**	**0**	**0**	**0**	**0**	**0**
**Proteus vulgaris/bla NDM**																			
Não Detectável	0	0	0	0	0	0	0	0	0	0	0	0	0	0	0	0	0	0	0

Microrganismo /Gene Pesquisado	Jan/2018	Fev/2018	Mar/2018	Abr/2018	Maio/2018	Jun/2018	Jul/2018	Ago/2018	Set/2018	Out/2018	Nov/2018	Dez/2018	Jan/2019	Fev/2019	Mar/2019	Abr/2019	Maio/2019	Jun/2019	Jul/2019
**Subtotal**	**0**	**0**	**0**	**0**	**0**	**0**	**0**	**0**	**0**	**0**	**0**	**0**	**0**	**0**	**0**	**0**	**0**	**0**	**0**
**Proteus vulgaris/bla OXA-48**																			
Não Detectável	0	0	0	0	0	0	0	0	0	0	0	0	0	0	0	0	0	0	0
**Subtotal**	**0**	**0**	**0**	**0**	**0**	**0**	**0**	**0**	**0**	**0**	**0**	**0**	**0**	**0**	**0**	**0**	**0**	**0**	**0**
**Proteus vulgaris/bla SPM**																			
Não Detectável	0	0	0	0	0	0	0	0	0	0	0	0	0	0	0	0	0	0	0
**Subtotal**	**0**	**0**	**0**	**0**	**0**	**0**	**0**	**0**	**0**	**0**	**0**	**0**	**0**	**0**	**0**	**0**	**0**	**0**	**0**
**Proteus vulgaris/blaVIM**																			
Não Detectável	0	0	0	0	0	0	0	0	0	0	0	0	0	0	0	0	0	0	0
**Subtotal**	**0**	**0**	**0**	**0**	**0**	**0**	**0**	**0**	**0**	**0**	**0**	**0**	**0**	**0**	**0**	**0**	**0**	**0**	**0**
**Proteus vulgaris/mcr-1**																			
Não Detectável	0	0	0	0	0	0	0	0	0	0	0	0	0	0	0	0	0	0	0
**Subtotal**	**0**	**0**	**0**	**0**	**0**	**0**	**0**	**0**	**0**	**0**	**0**	**0**	**0**	**0**	**0**	**0**	**0**	**0**	**0**
**Providencia rettgeri/bla IMP**																			
Não Detectável	0	0	0	0	0	0	0	0	0	0	0	0	0	0	0	0	0	0	0
**Subtotal**	**0**	**0**	**0**	**0**	**0**	**0**	**0**	**0**	**0**	**0**	**0**	**0**	**0**	**0**	**0**	**0**	**0**	**0**	**0**
**Providencia rettgeri/bla KPC**																			
Não Detectável	0	0	0	0	0	0	0	0	0	0	0	0	0	0	0	0	0	0	0
**Subtotal**	**0**	**0**	**0**	**0**	**0**	**0**	**0**	**0**	**0**	**0**	**0**	**0**	**0**	**0**	**0**	**0**	**0**	**0**	**0**
**Providencia rettgeri/bla NDM**																			
Não Detectável	0	0	0	0	0	0	0	0	0	0	0	0	0	0	0	0	0	0	0
**Subtotal**	**0**	**0**	**0**	**0**	**0**	**0**	**0**	**0**	**0**	**0**	**0**	**0**	**0**	**0**	**0**	**0**	**0**	**0**	**0**
**Providencia rettgeri/bla OXA-23**																			
Não Detectável	0	0	0	0	0	0	0	0	0	0	0	0	0	0	0	0	0	0	0
**Subtotal**	**0**	**0**	**0**	**0**	**0**	**0**	**0**	**0**	**0**	**0**	**0**	**0**	**0**	**0**	**0**	**0**	**0**	**0**	**0**
**Providencia rettgeri/bla OXA-48**																			
Detectável	0	0	0	0	0	0	0	0	0	0	0	0	0	0	0	0	0	0	0
Não Detectável	0	0	0	0	0	0	0	0	0	0	0	0	0	0	0	0	0	0	0
**Subtotal**	**0**	**0**	**0**	**0**	**0**	**0**	**0**	**0**	**0**	**0**	**0**	**0**	**0**	**0**	**0**	**0**	**0**	**0**	**0**
**Providencia rettgeri/bla OXA-51**																			
Não Detectável	0	0	0	0	0	0	0	0	0	0	0	0	0	0	0	0	0	0	0
**Subtotal**	**0**	**0**	**0**	**0**	**0**	**0**	**0**	**0**	**0**	**0**	**0**	**0**	**0**	**0**	**0**	**0**	**0**	**0**	**0**
**Providencia rettgeri/bla OXA-58**																			
Não Detectável	0	0	0	0	0	0	0	0	0	0	0	0	0	0	0	0	0	0	0
**Subtotal**	**0**	**0**	**0**	**0**	**0**	**0**	**0**	**0**	**0**	**0**	**0**	**0**	**0**	**0**	**0**	**0**	**0**	**0**	**0**
**Providencia rettgeri/bla SPM**																			
Não Detectável	0	0	0	0	0	0	0	0	0	0	0	0	0	0	0	0	0	0	0
**Subtotal**	**0**	**0**	**0**	**0**	**0**	**0**	**0**	**0**	**0**	**0**	**0**	**0**	**0**	**0**	**0**	**0**	**0**	**0**	**0**
**Providencia rettgeri/blaVIM**																			
Não Detectável	0	0	0	0	0	0	0	0	0	0	0	0	0	0	0	0	0	0	0
**Subtotal**	**0**	**0**	**0**	**0**	**0**	**0**	**0**	**0**	**0**	**0**	**0**	**0**	**0**	**0**	**0**	**0**	**0**	**0**	**0**
**Providencia rustigianii/bla IMP**																			
Não Detectável	0	0	0	0	0	0	0	0	0	0	0	0	0	0	0	0	0	0	0
**Subtotal**	**0**	**0**	**0**	**0**	**0**	**0**	**0**	**0**	**0**	**0**	**0**	**0**	**0**	**0**	**0**	**0**	**0**	**0**	**0**
**Providencia rustigianii/bla KPC**																			
Não Detectável	0	0	0	0	0	0	0	0	0	0	0	0	0	0	0	0	0	0	0
**Subtotal**	**0**	**0**	**0**	**0**	**0**	**0**	**0**	**0**	**0**	**0**	**0**	**0**	**0**	**0**	**0**	**0**	**0**	**0**	**0**
**Providencia rustigianii/bla NDM**																			
Não Detectável	0	0	0	0	0	0	0	0	0	0	0	0	0	0	0	0	0	0	0
**Subtotal**	**0**	**0**	**0**	**0**	**0**	**0**	**0**	**0**	**0**	**0**	**0**	**0**	**0**	**0**	**0**	**0**	**0**	**0**	**0**
**Providencia rustigianii/bla OXA-23**																			
Não Detectável	0	0	0	0	0	0	0	0	0	0	0	0	0	0	0	0	0	0	0
**Subtotal**	**0**	**0**	**0**	**0**	**0**	**0**	**0**	**0**	**0**	**0**	**0**	**0**	**0**	**0**	**0**	**0**	**0**	**0**	**0**
**Providencia rustigianii/bla OXA-48**																			

Microrganismo /Gene Pesquisado	Jan/2018	Fev/2018	Mar/2018	Abr/2018	Maio/2018	Jun/2018	Jul/2018	Ago/2018	Set/2018	Out/2018	Nov/2018	Dez/2018	Jan/2019	Fev/2019	Mar/2019	Abr/2019	Maio/2019	Jun/2019	Jul/2019
Não Detectável	0	0	0	0	0	0	0	0	0	0	0	0	0	0	0	0	0	0	0
**Subtotal**	**0**	**0**	**0**	**0**	**0**	**0**	**0**	**0**	**0**	**0**	**0**	**0**	**0**	**0**	**0**	**0**	**0**	**0**	**0**
**Providencia rustigianii/bla OXA-51**																			
Não Detectável	0	0	0	0	0	0	0	0	0	0	0	0	0	0	0	0	0	0	0
**Subtotal**	**0**	**0**	**0**	**0**	**0**	**0**	**0**	**0**	**0**	**0**	**0**	**0**	**0**	**0**	**0**	**0**	**0**	**0**	**0**
**Providencia rustigianii/bla OXA-58**																			
Não Detectável	0	0	0	0	0	0	0	0	0	0	0	0	0	0	0	0	0	0	0
**Subtotal**	**0**	**0**	**0**	**0**	**0**	**0**	**0**	**0**	**0**	**0**	**0**	**0**	**0**	**0**	**0**	**0**	**0**	**0**	**0**
**Providencia rustigianii/bla SPM**																			
Não Detectável	0	0	0	0	0	0	0	0	0	0	0	0	0	0	0	0	0	0	0
**Subtotal**	**0**	**0**	**0**	**0**	**0**	**0**	**0**	**0**	**0**	**0**	**0**	**0**	**0**	**0**	**0**	**0**	**0**	**0**	**0**
**Providencia rustigianii/blaVIM**																			
Não Detectável	0	0	0	0	0	0	0	0	0	0	0	0	0	0	0	0	0	0	0
**Subtotal**	**0**	**0**	**0**	**0**	**0**	**0**	**0**	**0**	**0**	**0**	**0**	**0**	**0**	**0**	**0**	**0**	**0**	**0**	**0**
**Providencia sp./bla KPC**																			
Não Detectável	0	0	0	0	0	0	0	0	0	0	0	0	0	1	0	0	0	0	0
**Subtotal**	**0**	**0**	**0**	**0**	**0**	**0**	**0**	**0**	**0**	**0**	**0**	**0**	**0**	**1**	**0**	**0**	**0**	**0**	**0**
**Providencia stuartii/bla IMP**																			
Não Detectável	0	0	0	0	0	0	0	0	0	0	0	0	0	0	0	0	0	0	0
**Subtotal**	**0**	**0**	**0**	**0**	**0**	**0**	**0**	**0**	**0**	**0**	**0**	**0**	**0**	**0**	**0**	**0**	**0**	**0**	**0**
**Providencia stuartii/bla KPC**																			
Detectável	0	0	0	0	0	0	0	0	0	0	0	0	0	0	0	0	0	0	0
Não Detectável	0	0	0	0	0	0	0	0	0	0	0	0	0	0	0	0	0	0	0
**Subtotal**	**0**	**0**	**0**	**0**	**0**	**0**	**0**	**0**	**0**	**0**	**0**	**0**	**0**	**0**	**0**	**0**	**0**	**0**	**0**
**Providencia stuartii/bla NDM**																			
Detectável	0	0	0	0	0	0	0	0	0	0	0	0	0	0	0	0	0	0	0
Não Detectável	0	0	0	0	0	0	0	0	0	0	0	0	0	0	0	0	0	0	0
**Subtotal**	**0**	**0**	**0**	**0**	**0**	**0**	**0**	**0**	**0**	**0**	**0**	**0**	**0**	**0**	**0**	**0**	**0**	**0**	**0**
**Providencia stuartii/bla OXA-23**																			
Não Detectável	0	0	0	0	0	0	0	0	0	0	0	0	0	0	0	0	0	0	0
**Subtotal**	**0**	**0**	**0**	**0**	**0**	**0**	**0**	**0**	**0**	**0**	**0**	**0**	**0**	**0**	**0**	**0**	**0**	**0**	**0**
**Providencia stuartii/bla OXA-48**																			
Não Detectável	0	0	0	0	0	0	0	0	0	0	0	0	0	0	0	0	0	0	0
**Subtotal**	**0**	**0**	**0**	**0**	**0**	**0**	**0**	**0**	**0**	**0**	**0**	**0**	**0**	**0**	**0**	**0**	**0**	**0**	**0**
**Providencia stuartii/bla OXA-51**																			
Não Detectável	0	0	0	0	0	0	0	0	0	0	0	0	0	0	0	0	0	0	0
**Subtotal**	**0**	**0**	**0**	**0**	**0**	**0**	**0**	**0**	**0**	**0**	**0**	**0**	**0**	**0**	**0**	**0**	**0**	**0**	**0**
**Providencia stuartii/bla OXA-58**																			
Não Detectável	0	0	0	0	0	0	0	0	0	0	0	0	0	0	0	0	0	0	0
**Subtotal**	**0**	**0**	**0**	**0**	**0**	**0**	**0**	**0**	**0**	**0**	**0**	**0**	**0**	**0**	**0**	**0**	**0**	**0**	**0**
**Providencia stuartii/bla SPM**																			
Não Detectável	0	0	0	0	0	0	0	0	0	0	0	0	0	0	0	0	0	0	0
**Subtotal**	**0**	**0**	**0**	**0**	**0**	**0**	**0**	**0**	**0**	**0**	**0**	**0**	**0**	**0**	**0**	**0**	**0**	**0**	**0**
**Providencia stuartii/blaVIM**																			
Não Detectável	0	0	0	0	0	0	0	0	0	0	0	0	0	0	0	0	0	0	0
**Subtotal**	**0**	**0**	**0**	**0**	**0**	**0**	**0**	**0**	**0**	**0**	**0**	**0**	**0**	**0**	**0**	**0**	**0**	**0**	**0**
**Providencia stuartii/mcr-1**																			
Não Detectável	0	0	0	0	0	0	0	0	0	0	0	0	0	0	0	0	0	0	0
**Subtotal**	**0**	**0**	**0**	**0**	**0**	**0**	**0**	**0**	**0**	**0**	**0**	**0**	**0**	**0**	**0**	**0**	**0**	**0**	**0**
**Providencia stuartii/NDM**																			
Não Detectável	0	0	0	0	0	0	0	0	0	0	0	0	0	0	0	0	0	0	0
**Subtotal**	**0**	**0**	**0**	**0**	**0**	**0**	**0**	**0**	**0**	**0**	**0**	**0**	**0**	**0**	**0**	**0**	**0**	**0**	**0**
**Pseudomonas aeruginosa/bla IMP**																			
Não Detectável	0	0	0	0	0	0	0	0	0	0	0	0	1	0	0	0	0	0	0

Microrganismo /Gene Pesquisado	Jan/2018	Fev/2018	Mar/2018	Abr/2018	Maio/2018	Jun/2018	Jul/2018	Ago/2018	Set/2018	Out/2018	Nov/2018	Dez/2018	Jan/2019	Fev/2019	Mar/2019	Abr/2019	Maio/2019	Jun/2019	Jul/2019
**Subtotal**	**0**	**0**	**0**	**0**	**0**	**0**	**0**	**0**	**0**	**0**	**0**	**0**	**1**	**0**	**0**	**0**	**0**	**0**	**0**
**Pseudomonas aeruginosa/bla KPC**																			
Detectável	0	0	0	0	0	0	0	0	0	0	0	0	0	0	0	0	0	0	0
Não Detectável	0	0	0	0	0	0	0	0	0	0	0	1	0	0	0	1	0	0	0
**Subtotal**	**0**	**0**	**0**	**0**	**0**	**0**	**0**	**0**	**0**	**0**	**0**	**1**	**0**	**0**	**0**	**1**	**0**	**0**	**0**
**Pseudomonas aeruginosa/bla NDM**																			
Não Detectável	0	0	0	0	0	0	0	0	0	1	0	0	2	0	0	0	0	0	0
**Subtotal**	**0**	**0**	**0**	**0**	**0**	**0**	**0**	**0**	**0**	**1**	**0**	**0**	**2**	**0**	**0**	**0**	**0**	**0**	**0**
**Pseudomonas aeruginosa/bla OXA-143**																			
Não Detectável	0	0	0	0	0	0	0	0	0	0	0	0	0	0	0	0	0	0	0
**Subtotal**	**0**	**0**	**0**	**0**	**0**	**0**	**0**	**0**	**0**	**0**	**0**	**0**	**0**	**0**	**0**	**0**	**0**	**0**	**0**
**Pseudomonas aeruginosa/bla OXA-23**																			
Detectável	0	0	0	0	0	0	0	0	0	0	0	0	0	0	0	0	0	0	0
Não Detectável	0	0	0	0	0	0	0	0	0	0	0	1	1	0	0	0	0	0	0
**Subtotal**	**0**	**0**	**0**	**0**	**0**	**0**	**0**	**0**	**0**	**0**	**0**	**1**	**1**	**0**	**0**	**0**	**0**	**0**	**0**
**Pseudomonas aeruginosa/bla OXA-48**																			
Detectável	0	0	0	0	0	0	0	0	0	0	0	0	0	0	0	0	0	0	0
Não Detectável	0	0	0	0	0	0	0	0	0	0	0	1	2	0	0	1	0	0	0
**Subtotal**	**0**	**0**	**0**	**0**	**0**	**0**	**0**	**0**	**0**	**0**	**0**	**1**	**2**	**0**	**0**	**1**	**0**	**0**	**0**
**Pseudomonas aeruginosa/bla OXA-51**																			
Detectável	0	0	0	0	0	0	0	0	0	0	0	0	0	0	0	0	0	0	0
Não Detectável	0	0	0	0	0	0	0	0	0	0	0	1	0	0	0	0	0	0	0
**Subtotal**	**0**	**0**	**0**	**0**	**0**	**0**	**0**	**0**	**0**	**0**	**0**	**1**	**0**	**0**	**0**	**0**	**0**	**0**	**0**
**Pseudomonas aeruginosa/bla OXA-58**																			
Não Detectável	0	0	0	0	0	0	0	0	0	0	0	0	0	0	0	0	0	0	0
**Subtotal**	**0**	**0**	**0**	**0**	**0**	**0**	**0**	**0**	**0**	**0**	**0**	**0**	**0**	**0**	**0**	**0**	**0**	**0**	**0**
**Pseudomonas aeruginosa/bla SPM**																			
Detectável	0	0	0	0	0	0	0	6	1	0	0	1	1	0	1	1	0	0	0
Inconclusivo	0	0	0	0	0	0	0	0	0	0	0	0	0	0	0	0	0	0	0
Não Detectável	0	0	0	0	0	0	0	3	7	4	0	4	1	0	6	3	5	1	3
**Subtotal**	**0**	**0**	**0**	**0**	**0**	**0**	**0**	**9**	**8**	**4**	**0**	**5**	**2**	**0**	**7**	**4**	**5**	**1**	**3**
**Pseudomonas aeruginosa/blaVIM**																			
Detectável	0	0	0	0	0	0	0	0	0	0	0	1	0	0	0	0	0	0	0
Não Detectável	0	0	0	0	0	0	0	7	8	5	0	0	1	0	6	3	5	0	3
**Subtotal**	**0**	**0**	**0**	**0**	**0**	**0**	**0**	**7**	**8**	**5**	**0**	**1**	**1**	**0**	**6**	**3**	**5**	**0**	**3**
**Pseudomonas aeruginosa/IMP**																			
Não Detectável	0	0	0	0	0	0	0	0	0	0	0	0	0	0	0	0	0	0	0
**Subtotal**	**0**	**0**	**0**	**0**	**0**	**0**	**0**	**0**	**0**	**0**	**0**	**0**	**0**	**0**	**0**	**0**	**0**	**0**	**0**
**Pseudomonas aeruginosa/KPC**																			
Não Detectável	0	0	0	0	0	0	0	0	0	0	0	0	0	0	0	0	0	0	0
**Subtotal**	**0**	**0**	**0**	**0**	**0**	**0**	**0**	**0**	**0**	**0**	**0**	**0**	**0**	**0**	**0**	**0**	**0**	**0**	**0**
**Pseudomonas aeruginosa/SPM**																			
Detectável	0	0	0	0	0	0	0	5	1	0	0	0	0	0	0	0	0	0	0
Não Detectável	0	0	0	0	0	1	0	2	0	0	0	0	0	0	0	0	0	0	0
**Subtotal**	**0**	**0**	**0**	**0**	**0**	**1**	**0**	**7**	**1**	**0**	**0**	**0**	**0**	**0**	**0**	**0**	**0**	**0**	**0**
**Pseudomonas aeruginosa/VIM**																			
Não Detectável	0	0	0	0	0	0	0	6	1	0	0	0	0	0	0	0	0	0	0
**Subtotal**	**0**	**0**	**0**	**0**	**0**	**0**	**0**	**6**	**1**	**0**	**0**	**0**	**0**	**0**	**0**	**0**	**0**	**0**	**0**
**Pseudomonas fluorescens/bla IMP**																			
Não Detectável	0	0	0	0	0	0	0	0	0	0	0	0	0	0	0	0	0	0	0
**Subtotal**	**0**	**0**	**0**	**0**	**0**	**0**	**0**	**0**	**0**	**0**	**0**	**0**	**0**	**0**	**0**	**0**	**0**	**0**	**0**
**Pseudomonas fluorescens/bla KPC**																			
Não Detectável	0	0	0	0	0	0	0	0	0	0	0	0	0	0	0	0	0	0	0
**Subtotal**	**0**	**0**	**0**	**0**	**0**	**0**	**0**	**0**	**0**	**0**	**0**	**0**	**0**	**0**	**0**	**0**	**0**	**0**	**0**

Microrganismo /Gene Pesquisado	Jan/2018	Fev/2018	Mar/2018	Abr/2018	Maio/2018	Jun/2018	Jul/2018	Ago/2018	Set/2018	Out/2018	Nov/2018	Dez/2018	Jan/2019	Fev/2019	Mar/2019	Abr/2019	Maio/2019	Jun/2019	Jul/2019
**Pseudomonas fluorescens/bla SPM**																			
Não Detectável	0	0	0	0	0	0	0	0	0	0	0	0	0	0	0	0	0	0	0
**Subtotal**	**0**	**0**	**0**	**0**	**0**	**0**	**0**	**0**	**0**	**0**	**0**	**0**	**0**	**0**	**0**	**0**	**0**	**0**	**0**
**Pseudomonas putida/bla IMP**																			
Não Detectável	0	0	0	0	0	0	0	0	0	0	0	0	0	0	0	0	0	0	0
**Subtotal**	**0**	**0**	**0**	**0**	**0**	**0**	**0**	**0**	**0**	**0**	**0**	**0**	**0**	**0**	**0**	**0**	**0**	**0**	**0**
**Pseudomonas putida/bla KPC**																			
Não Detectável	0	0	0	0	0	0	0	0	0	0	0	0	0	0	0	0	0	0	0
**Subtotal**	**0**	**0**	**0**	**0**	**0**	**0**	**0**	**0**	**0**	**0**	**0**	**0**	**0**	**0**	**0**	**0**	**0**	**0**	**0**
**Pseudomonas putida/bla NDM**																			
Não Detectável	0	0	0	0	0	0	0	0	0	0	0	0	0	0	0	0	0	0	0
**Subtotal**	**0**	**0**	**0**	**0**	**0**	**0**	**0**	**0**	**0**	**0**	**0**	**0**	**0**	**0**	**0**	**0**	**0**	**0**	**0**
**Pseudomonas putida/bla OXA-48**																			
Não Detectável	0	0	0	0	0	0	0	0	0	0	0	0	0	0	0	0	0	0	0
**Subtotal**	**0**	**0**	**0**	**0**	**0**	**0**	**0**	**0**	**0**	**0**	**0**	**0**	**0**	**0**	**0**	**0**	**0**	**0**	**0**
**Pseudomonas putida/bla SPM**																			
Não Detectável	0	0	0	0	0	0	0	0	0	0	0	0	0	0	0	0	0	0	0
**Subtotal**	**0**	**0**	**0**	**0**	**0**	**0**	**0**	**0**	**0**	**0**	**0**	**0**	**0**	**0**	**0**	**0**	**0**	**0**	**0**
**Pseudomonas putida/blaVIM**																			
Não Detectável	0	0	0	0	0	0	0	0	0	0	0	0	0	0	0	0	0	0	0
**Subtotal**	**0**	**0**	**0**	**0**	**0**	**0**	**0**	**0**	**0**	**0**	**0**	**0**	**0**	**0**	**0**	**0**	**0**	**0**	**0**
**Pseudomonas sp./bla KPC**																			
Não Detectável	0	0	0	0	0	0	0	0	0	0	0	0	0	0	0	0	0	0	0
**Subtotal**	**0**	**0**	**0**	**0**	**0**	**0**	**0**	**0**	**0**	**0**	**0**	**0**	**0**	**0**	**0**	**0**	**0**	**0**	**0**
**Pseudomonas sp./bla NDM**																			
Não Detectável	0	0	0	0	0	0	0	0	0	0	0	0	0	0	0	0	0	0	0
**Subtotal**	**0**	**0**	**0**	**0**	**0**	**0**	**0**	**0**	**0**	**0**	**0**	**0**	**0**	**0**	**0**	**0**	**0**	**0**	**0**
**Pseudomonas sp./bla OXA-48**																			
Não Detectável	0	0	0	0	0	0	0	0	0	0	0	0	0	0	0	0	0	0	0
**Subtotal**	**0**	**0**	**0**	**0**	**0**	**0**	**0**	**0**	**0**	**0**	**0**	**0**	**0**	**0**	**0**	**0**	**0**	**0**	**0**
**Pseudomonas sp./bla SPM**																			
Não Detectável	0	0	0	0	0	0	0	0	0	0	0	0	0	0	0	0	0	4	1
**Subtotal**	**0**	**0**	**0**	**0**	**0**	**0**	**0**	**0**	**0**	**0**	**0**	**0**	**0**	**0**	**0**	**0**	**0**	**4**	**1**
**Pseudomonas sp./blaVIM**																			
Não Detectável	0	0	0	0	0	0	0	0	0	0	0	0	0	0	0	0	0	0	1
**Subtotal**	**0**	**0**	**0**	**0**	**0**	**0**	**0**	**0**	**0**	**0**	**0**	**0**	**0**	**0**	**0**	**0**	**0**	**0**	**1**
**Serratia ficaria/bla IMP**																			
Não Detectável	0	0	0	0	0	0	0	0	0	0	0	0	0	0	0	0	0	0	0
**Subtotal**	**0**	**0**	**0**	**0**	**0**	**0**	**0**	**0**	**0**	**0**	**0**	**0**	**0**	**0**	**0**	**0**	**0**	**0**	**0**
**Serratia ficaria/bla KPC**																			
Não Detectável	0	0	0	0	0	0	0	0	0	0	0	0	0	0	0	0	0	0	0
**Subtotal**	**0**	**0**	**0**	**0**	**0**	**0**	**0**	**0**	**0**	**0**	**0**	**0**	**0**	**0**	**0**	**0**	**0**	**0**	**0**
**Serratia ficaria/bla NDM**																			
Não Detectável	0	0	0	0	0	0	0	0	0	0	0	0	0	0	0	0	0	0	0
**Subtotal**	**0**	**0**	**0**	**0**	**0**	**0**	**0**	**0**	**0**	**0**	**0**	**0**	**0**	**0**	**0**	**0**	**0**	**0**	**0**
**Serratia ficaria/bla OXA-48**																			
Não Detectável	0	0	0	0	0	0	0	0	0	0	0	0	0	0	0	0	0	0	0
**Subtotal**	**0**	**0**	**0**	**0**	**0**	**0**	**0**	**0**	**0**	**0**	**0**	**0**	**0**	**0**	**0**	**0**	**0**	**0**	**0**
**Serratia ficaria/bla SPM**																			
Não Detectável	0	0	0	0	0	0	0	0	0	0	0	0	0	0	0	0	0	0	0
**Subtotal**	**0**	**0**	**0**	**0**	**0**	**0**	**0**	**0**	**0**	**0**	**0**	**0**	**0**	**0**	**0**	**0**	**0**	**0**	**0**
**Serratia ficaria/blaVIM**																			
Não Detectável	0	0	0	0	0	0	0	0	0	0	0	0	0	0	0	0	0	0	0
**Subtotal**	**0**	**0**	**0**	**0**	**0**	**0**	**0**	**0**	**0**	**0**	**0**	**0**	**0**	**0**	**0**	**0**	**0**	**0**	**0**

Microrganismo /Gene Pesquisado	Jan/2018	Fev/2018	Mar/2018	Abr/2018	Maio/2018	Jun/2018	Jul/2018	Ago/2018	Set/2018	Out/2018	Nov/2018	Dez/2018	Jan/2019	Fev/2019	Mar/2019	Abr/2019	Maio/2019	Jun/2019	Jul/2019
**Serratia liquefaciens/bla KPC**																			
Não Detectável	0	0	0	0	0	0	0	0	0	0	0	0	0	0	0	0	0	0	0
**Subtotal**	**0**	**0**	**0**	**0**	**0**	**0**	**0**	**0**	**0**	**0**	**0**	**0**	**0**	**0**	**0**	**0**	**0**	**0**	**0**
**Serratia marcescens/bla IMP**																			
Não Detectável	0	0	0	0	0	0	0	0	0	0	0	1	0	0	0	0	0	0	0
**Subtotal**	**0**	**0**	**0**	**0**	**0**	**0**	**0**	**0**	**0**	**0**	**0**	**1**	**0**	**0**	**0**	**0**	**0**	**0**	**0**
**Serratia marcescens/bla KPC**																			
Detectável	0	0	0	0	2	0	0	0	1	0	0	1	0	0	1	0	1	1	0
Não Detectável	0	0	0	0	0	0	0	0	0	1	0	0	0	0	0	0	0	0	0
**Subtotal**	**0**	**0**	**0**	**0**	**2**	**0**	**0**	**0**	**1**	**1**	**0**	**1**	**0**	**0**	**1**	**0**	**1**	**1**	**0**
**Serratia marcescens/bla NDM**																			
Não Detectável	0	0	0	0	0	0	0	3	2	1	0	1	0	1	4	0	3	2	1
**Subtotal**	**0**	**0**	**0**	**0**	**0**	**0**	**0**	**3**	**2**	**1**	**0**	**1**	**0**	**1**	**4**	**0**	**3**	**2**	**1**
**Serratia marcescens/bla OXA-23**																			
Não Detectável	0	0	0	0	0	0	0	0	0	0	0	0	0	0	0	0	0	0	0
**Subtotal**	**0**	**0**	**0**	**0**	**0**	**0**	**0**	**0**	**0**	**0**	**0**	**0**	**0**	**0**	**0**	**0**	**0**	**0**	**0**
**Serratia marcescens/bla OXA-48**																			
Não Detectável	0	0	0	0	0	0	0	0	0	0	0	0	0	0	0	0	0	0	0
**Subtotal**	**0**	**0**	**0**	**0**	**0**	**0**	**0**	**0**	**0**	**0**	**0**	**0**	**0**	**0**	**0**	**0**	**0**	**0**	**0**
**Serratia marcescens/bla OXA-51**																			
Não Detectável	0	0	0	0	0	0	0	0	0	0	0	0	0	0	0	0	0	0	0
**Subtotal**	**0**	**0**	**0**	**0**	**0**	**0**	**0**	**0**	**0**	**0**	**0**	**0**	**0**	**0**	**0**	**0**	**0**	**0**	**0**
**Serratia marcescens/bla OXA-58**																			
Não Detectável	0	0	0	0	0	0	0	0	0	0	0	0	0	0	0	0	0	0	0
**Subtotal**	**0**	**0**	**0**	**0**	**0**	**0**	**0**	**0**	**0**	**0**	**0**	**0**	**0**	**0**	**0**	**0**	**0**	**0**	**0**
**Serratia marcescens/bla SPM**																			
Não Detectável	0	0	0	0	0	0	0	0	0	0	0	0	0	0	0	0	0	0	0
**Subtotal**	**0**	**0**	**0**	**0**	**0**	**0**	**0**	**0**	**0**	**0**	**0**	**0**	**0**	**0**	**0**	**0**	**0**	**0**	**0**
**Serratia marcescens/blaVIM**																			
Não Detectável	0	0	0	0	0	0	0	0	0	0	0	0	0	0	0	0	0	0	0
**Subtotal**	**0**	**0**	**0**	**0**	**0**	**0**	**0**	**0**	**0**	**0**	**0**	**0**	**0**	**0**	**0**	**0**	**0**	**0**	**0**
**Serratia marcescens/mcr-1**																			
Não Detectável	0	0	0	0	0	0	0	0	0	0	0	0	0	0	0	0	0	0	0
**Subtotal**	**0**	**0**	**0**	**0**	**0**	**0**	**0**	**0**	**0**	**0**	**0**	**0**	**0**	**0**	**0**	**0**	**0**	**0**	**0**
**Serratia plymuthica/bla KPC**																			
Não Detectável	0	0	0	0	0	0	0	0	0	0	0	0	0	0	0	0	0	0	0
**Subtotal**	**0**	**0**	**0**	**0**	**0**	**0**	**0**	**0**	**0**	**0**	**0**	**0**	**0**	**0**	**0**	**0**	**0**	**0**	**0**
**Serratia plymuthica/bla NDM**																			
Inconclusivo	0	0	0	0	0	0	0	0	0	0	0	0	0	0	0	0	0	0	0
**Subtotal**	**0**	**0**	**0**	**0**	**0**	**0**	**0**	**0**	**0**	**0**	**0**	**0**	**0**	**0**	**0**	**0**	**0**	**0**	**0**
**Serratia rubidaea/bla KPC**																			
Detectável	0	0	0	0	0	0	0	0	0	1	0	0	0	0	0	0	0	0	0
**Subtotal**	**0**	**0**	**0**	**0**	**0**	**0**	**0**	**0**	**0**	**1**	**0**	**0**	**0**	**0**	**0**	**0**	**0**	**0**	**0**
**Serratia sp./bla KPC**																			
Detectável	0	0	0	0	0	0	0	0	1	0	0	0	0	0	0	0	0	0	0
Não Detectável	0	0	0	0	0	0	0	0	1	2	0	0	0	0	0	0	0	0	0
**Subtotal**	**0**	**0**	**0**	**0**	**0**	**0**	**0**	**0**	**2**	**2**	**0**	**0**	**0**	**0**	**0**	**0**	**0**	**0**	**0**
**Serratia sp./bla NDM**																			
Não Detectável	0	0	0	0	0	0	0	0	2	2	0	0	0	0	0	0	0	0	0
**Subtotal**	**0**	**0**	**0**	**0**	**0**	**0**	**0**	**0**	**2**	**2**	**0**	**0**	**0**	**0**	**0**	**0**	**0**	**0**	**0**
**Staphylococcus aureus/bla KPC**																			
Não Detectável	0	0	0	0	0	0	0	0	0	0	0	0	0	0	0	0	0	0	0
**Subtotal**	**0**	**0**	**0**	**0**	**0**	**0**	**0**	**0**	**0**	**0**	**0**	**0**	**0**	**0**	**0**	**0**	**0**	**0**	**0**
**Staphylococcus aureus/bla NDM**																			

Microrganismo /Gene Pesquisado	Jan/2018	Fev/2018	Mar/2018	Abr/2018	Maio/2018	Jun/2018	Jul/2018	Ago/2018	Set/2018	Out/2018	Nov/2018	Dez/2018	Jan/2019	Fev/2019	Mar/2019	Abr/2019	Maio/2019	Jun/2019	Jul/2019
Não Detectável	0	0	0	0	0	0	0	0	0	0	0	0	0	0	0	0	0	0	0
**Subtotal**	**0**	**0**	**0**	**0**	**0**	**0**	**0**	**0**	**0**	**0**	**0**	**0**	**0**	**0**	**0**	**0**	**0**	**0**	**0**
**Staphylococcus aureus/mec a**																			
Detectável	0	0	0	0	0	0	0	0	0	0	0	0	3	0	0	0	0	0	0
Não Detectável	0	0	0	0	0	0	0	0	0	0	0	0	0	0	0	0	0	1	0
**Subtotal**	**0**	**0**	**0**	**0**	**0**	**0**	**0**	**0**	**0**	**0**	**0**	**0**	**3**	**0**	**0**	**0**	**0**	**1**	**0**
**Staphylococcus aureus/outros**																			
Detectável	0	0	0	0	0	0	0	0	0	0	0	0	0	0	0	0	0	0	0
Não Detectável	0	0	0	0	0	0	0	0	0	0	0	0	0	0	0	0	0	1	0
**Subtotal**	**0**	**0**	**0**	**0**	**0**	**0**	**0**	**0**	**0**	**0**	**0**	**0**	**0**	**0**	**0**	**0**	**0**	**1**	**0**
**Staphylococcus epidermidis/mec a**																			
Detectável	0	0	0	0	0	0	0	0	0	0	0	0	0	0	0	0	0	0	0
**Subtotal**	**0**	**0**	**0**	**0**	**0**	**0**	**0**	**0**	**0**	**0**	**0**	**0**	**0**	**0**	**0**	**0**	**0**	**0**	**0**
**Staphylococcus lugdunensis/mec a**																			
Detectável	0	0	0	0	0	0	0	0	0	0	0	0	0	0	0	0	0	0	0
**Subtotal**	**0**	**0**	**0**	**0**	**0**	**0**	**0**	**0**	**0**	**0**	**0**	**0**	**0**	**0**	**0**	**0**	**0**	**0**	**0**
**Stenotrophomonas maltophilia/bla KPC**																			
Não Detectável	0	0	0	0	0	0	0	0	0	0	0	0	0	0	0	0	0	0	0
**Subtotal**	**0**	**0**	**0**	**0**	**0**	**0**	**0**	**0**	**0**	**0**	**0**	**0**	**0**	**0**	**0**	**0**	**0**	**0**	**0**
**Stenotrophomonas maltophilia/bla OXA-48**																			
Não Detectável	0	0	0	0	0	0	0	0	0	0	0	0	0	0	0	0	0	0	0
**Subtotal**	**0**	**0**	**0**	**0**	**0**	**0**	**0**	**0**	**0**	**0**	**0**	**0**	**0**	**0**	**0**	**0**	**0**	**0**	**0**
**Stenotrophomonas maltophilia/bla OXA-51**																			
Detectável	0	0	0	0	0	0	0	0	0	0	0	0	0	0	0	0	0	0	0
**Subtotal**	**0**	**0**	**0**	**0**	**0**	**0**	**0**	**0**	**0**	**0**	**0**	**0**	**0**	**0**	**0**	**0**	**0**	**0**	**0**
**Stenotrophomonas maltophilia/bla SPM**																			
Detectável	0	0	0	0	0	0	0	0	0	0	0	0	0	0	0	0	0	0	0
Não Detectável	0	0	0	0	0	0	0	0	0	0	0	0	0	0	0	0	0	0	0
**Subtotal**	**0**	**0**	**0**	**0**	**0**	**0**	**0**	**0**	**0**	**0**	**0**	**0**	**0**	**0**	**0**	**0**	**0**	**0**	**0**
	0	0	0	1	0	0	0	1	0	0	0	4	2	1	0	0	1	1	0
**Subtotal**	**0**	**0**	**0**	**1**	**0**	**0**	**0**	**1**	**0**	**0**	**0**	**4**	**2**	**1**	**0**	**0**	**1**	**1**	**0**
