# Supplementary material for: Biodiversity of carbapenem-resistant bacteria in clinical samples from the Southwest Amazon region (Rondônia/Brazil)
Source: Sci Rep. 2024 Apr 23;14:9383. doi: 10.1038/s41598-024-59733-w (PMC11039742; doi:10.1038/s41598-024-59733-w)
Supplement: Supplementary file 7 — Supplementary Information 7. [file 41598_2024_59733_MOESM7_ESM.pdf]

Relatório Pesquisa de Genes de Resistência

Data Início: 01/01/2019

Data Fim: 31/12/2019

Qtd. de Exame/Metodologia: 367

Qtd. de Microrganismo/Gene Pesq.: 68

Todos os Laboratórios

Microrganismo /Gene Pesquisado	Jan/2019	Fev/2019	Mar/2019	Abr/2019	Maio/2019	Jun/2019	Jul/2019	Ago/2019	Set/2019	Out/2019	Nov/2019	Dez/2019	Total
**Acinetobacter baumannii/bla KPC**													
Não Detectável	0	0	0	0	0	0	0	0	0	3	0	0	3
**Subtotal**	**0**	**0**	**0**	**0**	**0**	**0**	**0**	**0**	**0**	**3**	**0**	**0**	**3**
**Acinetobacter baumannii/bla OXA-143**													
Não Detectável	0	0	0	0	0	3	0	0	0	0	0	0	3
**Subtotal**	**0**	**0**	**0**	**0**	**0**	**3**	**0**	**0**	**0**	**0**	**0**	**0**	**6**
**Acinetobacter baumannii/bla OXA-23**													
Detectável	14	8	16	6	12	5	11	4	5	22	4	2	109
Não Detectável	0	0	4	1	2	3	8	11	0	0	0	0	29
**Subtotal**	**14**	**8**	**20**	**7**	**14**	**8**	**19**	**15**	**5**	**22**	**4**	**2**	**144**
**Acinetobacter baumannii/bla OXA-48**													
Não Detectável	0	0	0	0	1	0	1	0	0	0	0	0	2
**Subtotal**	**0**	**0**	**0**	**0**	**1**	**0**	**1**	**0**	**0**	**0**	**0**	**0**	**146**
**Acinetobacter baumannii/bla OXA-51**													
Detectável	14	8	15	6	14	7	18	0	2	27	14	2	127
Não Detectável	0	0	3	1	2	1	1	11	0	0	0	0	19
**Subtotal**	**14**	**8**	**18**	**7**	**16**	**8**	**19**	**11**	**2**	**27**	**14**	**2**	**292**
**Acinetobacter baumannii/bla OXA-58**													
Detectável	0	0	0	0	0	0	0	4	1	6	10	0	21
Não Detectável	0	0	0	0	0	2	0	1	0	0	0	0	3
**Subtotal**	**0**	**0**	**0**	**0**	**0**	**2**	**0**	**5**	**1**	**6**	**10**	**0**	**316**
**Acinetobacter sp./bla OXA-23**													
Detectável	0	0	0	0	6	3	0	0	0	2	0	0	11
**Subtotal**	**0**	**0**	**0**	**0**	**6**	**3**	**0**	**0**	**0**	**2**	**0**	**0**	**327**
**Acinetobacter sp./bla OXA-51**													
Detectável	0	0	0	0	4	3	0	0	0	2	0	0	9

Relatório Pesquisa de Genes de Resistência

Microrganismo /Gene Pesquisado	Jan/2019	Fev/2019	Mar/2019	Abr/2019	Maio/2019	Jun/2019	Jul/2019	Ago/2019	Set/2019	Out/2019	Nov/2019	Dez/2019	Total
**Subtotal**	**0**	**0**	**0**	**0**	**4**	**3**	**0**	**0**	**0**	**2**	**0**	**0**	**336**
**Acinetobacter sp./bla OXA-58**													
Detectável	0	0	0	0	2	0	0	0	0	0	0	0	2
**Subtotal**	**0**	**0**	**0**	**0**	**2**	**0**	**0**	**0**	**0**	**0**	**0**	**0**	**338**
**Enterobacter aerogenes/bla KPC**													
Não Detectável	0	0	0	0	0	0	0	0	0	1	0	0	1
**Subtotal**	**0**	**0**	**0**	**0**	**0**	**0**	**0**	**0**	**0**	**1**	**0**	**0**	**339**
**Enterobacter aerogenes/NDM**													
Não Detectável	0	0	0	0	0	0	0	0	0	1	0	0	1
**Subtotal**	**0**	**0**	**0**	**0**	**0**	**0**	**0**	**0**	**0**	**1**	**0**	**0**	**340**
**Enterobacter cloacae/bla KPC**													
Detectável	0	0	0	1	1	0	0	0	0	0	0	0	2
Não Detectável	0	0	0	0	0	0	1	0	0	2	0	0	3
**Subtotal**	**0**	**0**	**0**	**1**	**1**	**0**	**1**	**0**	**0**	**2**	**0**	**0**	**345**
**Enterobacter cloacae/bla NDM**													
Não Detectável	0	0	0	0	0	0	0	0	0	2	0	0	2
**Subtotal**	**0**	**0**	**0**	**0**	**0**	**0**	**0**	**0**	**0**	**2**	**0**	**0**	**347**
**Enterobacter cloacae/bla OXA-48**													
Não Detectável	0	0	0	0	0	0	0	0	0	0	1	0	1
**Subtotal**	**0**	**0**	**0**	**0**	**0**	**0**	**0**	**0**	**0**	**0**	**1**	**0**	**348**
**Enterobacter cloacae/bla SPM**													
Não Detectável	0	0	0	0	0	0	0	0	0	1	0	0	1
**Subtotal**	**0**	**0**	**0**	**0**	**0**	**0**	**0**	**0**	**0**	**1**	**0**	**0**	**349**
**Enterobacter cloacae/blaVIM**													
Não Detectável	0	0	0	0	0	0	0	0	0	0	1	0	1
**Subtotal**	**0**	**0**	**0**	**0**	**0**	**0**	**0**	**0**	**0**	**0**	**1**	**0**	**350**
**Enterobacter sp./bla KPC**													
Não Detectável	0	0	0	0	0	1	0	0	0	0	0	0	1
**Subtotal**	**0**	**0**	**0**	**0**	**0**	**1**	**0**	**0**	**0**	**0**	**0**	**0**	**351**
**Escherichia coli/bla KPC**													
Detectável	1	0	2	0	2	0	0	4	1	2	0	0	12
Não Detectável	0	1	0	0	0	0	1	1	1	3	1	0	8
**Subtotal**	**1**	**1**	**2**	**0**	**2**	**0**	**1**	**5**	**2**	**5**	**1**	**0**	**371**
**Escherichia coli/bla NDM**													
Não Detectável	0	1	0	0	0	0	0	0	0	0	1	0	2
**Subtotal**	**0**	**1**	**0**	**0**	**0**	**0**	**0**	**0**	**0**	**0**	**1**	**0**	**373**

Relatório Pesquisa de Genes de Resistência

Microrganismo /Gene Pesquisado	Jan/2019	Fev/2019	Mar/2019	Abr/2019	Maio/2019	Jun/2019	Jul/2019	Ago/2019	Set/2019	Out/2019	Nov/2019	Dez/2019	Total
**Escherichia coli cepas verotoxigênicas como O103/outros**													
Detectável	0	0	0	0	0	0	0	0	0	0	1	0	1
**Subtotal**	**0**	**0**	**0**	**0**	**0**	**0**	**0**	**0**	**0**	**0**	**1**	**0**	**374**
**Escherichia coli enteroinvasora/outros**													
Detectável	0	0	0	0	0	0	0	0	0	0	1	0	1
**Subtotal**	**0**	**0**	**0**	**0**	**0**	**0**	**0**	**0**	**0**	**0**	**1**	**0**	**375**
**Escherichia coli enterotoxigênica/bla KPC**													
Não Detectável	0	0	0	0	0	0	1	0	0	0	0	0	1
**Subtotal**	**0**	**0**	**0**	**0**	**0**	**0**	**1**	**0**	**0**	**0**	**0**	**0**	**376**
**Escherichia coli enterotoxigênica/outros**													
Detectável	0	0	0	0	0	0	1	0	0	0	0	0	1
**Subtotal**	**0**	**0**	**0**	**0**	**0**	**0**	**1**	**0**	**0**	**0**	**0**	**0**	**377**
**Escherichia coli/KPC**													
Detectável	0	1	0	0	0	0	0	0	0	0	0	0	1
Não Detectável	0	0	0	0	0	0	0	0	0	1	0	0	1
**Subtotal**	**0**	**1**	**0**	**0**	**0**	**0**	**0**	**0**	**0**	**1**	**0**	**0**	**379**
**Klebsiella aerogenes/bla KPC**													
Não Detectável	0	0	0	0	0	0	0	2	0	0	0	0	2
**Subtotal**	**0**	**0**	**0**	**0**	**0**	**0**	**0**	**2**	**0**	**0**	**0**	**0**	**381**
**Klebsiella oxytoca/bla KPC**													
Não Detectável	0	1	0	0	0	0	0	1	0	0	0	0	2
**Subtotal**	**0**	**1**	**0**	**0**	**0**	**0**	**0**	**1**	**0**	**0**	**0**	**0**	**383**
**Klebsiella oxytoca/bla OXA-23**													
Não Detectável	0	0	1	0	0	0	0	0	0	0	0	0	1
**Subtotal**	**0**	**0**	**1**	**0**	**0**	**0**	**0**	**0**	**0**	**0**	**0**	**0**	**384**
**Klebsiella oxytoca/bla OXA-51**													
Não Detectável	0	0	1	0	0	0	0	0	0	0	0	0	1
**Subtotal**	**0**	**0**	**1**	**0**	**0**	**0**	**0**	**0**	**0**	**0**	**0**	**0**	**385**
**Klebsiella pneumoniae/bla IMP**													
Não Detectável	2	0	0	0	0	1	1	0	0	0	0	0	4
**Subtotal**	**2**	**0**	**0**	**0**	**0**	**1**	**1**	**0**	**0**	**0**	**0**	**0**	**389**
**Klebsiella pneumoniae/bla KPC**													
Detectável	11	1	18	3	1	1	8	1	1	3	5	1	54
Não Detectável	2	2	2	3	4	1	1	1	3	6	2	0	27
**Subtotal**	**13**	**3**	**20**	**6**	**5**	**2**	**9**	**2**	**4**	**9**	**7**	**1**	**470**
**Klebsiella pneumoniae/bla NDM**													

Relatório Pesquisa de Genes de Resistência

Microrganismo /Gene Pesquisado	Jan/2019	Fev/2019	Mar/2019	Abr/2019	Maio/2019	Jun/2019	Jul/2019	Ago/2019	Set/2019	Out/2019	Nov/2019	Dez/2019	Total
Detectável	0	0	0	1	0	0	0	0	0	0	0	0	1
Não Detectável	11	0	0	1	0	1	2	0	1	0	0	0	16
**Subtotal**	**11**	**0**	**0**	**2**	**0**	**1**	**2**	**0**	**1**	**0**	**0**	**0**	**487**
**Klebsiella pneumoniae/bla OXA-48**													
Não Detectável	4	0	0	4	0	0	1	0	0	0	0	0	9
**Subtotal**	**4**	**0**	**0**	**4**	**0**	**0**	**1**	**0**	**0**	**0**	**0**	**0**	**496**
**Klebsiella pneumoniae/bla SPM**													
Não Detectável	2	0	0	0	0	0	0	0	0	0	0	0	2
**Subtotal**	**2**	**0**	**0**	**0**	**0**	**0**	**0**	**0**	**0**	**0**	**0**	**0**	**498**
**Klebsiella pneumoniae/blaVIM**													
Não Detectável	3	0	0	0	0	0	0	0	0	0	0	0	3
**Subtotal**	**3**	**0**	**0**	**0**	**0**	**0**	**0**	**0**	**0**	**0**	**0**	**0**	**501**
**Klebsiella pneumoniae/KPC**													
Detectável	0	1	0	0	0	0	0	0	0	0	0	0	1
**Subtotal**	**0**	**1**	**0**	**0**	**0**	**0**	**0**	**0**	**0**	**0**	**0**	**0**	**502**
**Klebsiella pneumoniae/NDM**													
Não Detectável	0	0	1	0	0	0	0	0	0	0	0	0	1
**Subtotal**	**0**	**0**	**1**	**0**	**0**	**0**	**0**	**0**	**0**	**0**	**0**	**0**	**503**
**Klebsiella pneumoniae subsp. pneumoniae/bla KPC**													
Detectável	0	0	0	0	0	1	0	0	0	0	0	0	1
Não Detectável	0	0	3	0	0	1	0	0	0	0	0	0	4
**Subtotal**	**0**	**0**	**3**	**0**	**0**	**2**	**0**	**0**	**0**	**0**	**0**	**0**	**508**
**Klebsiella sp./bla KPC**													
Detectável	2	0	1	0	1	0	1	0	0	0	0	0	5
Não Detectável	0	0	0	0	1	0	0	1	0	0	0	0	2
**Subtotal**	**2**	**0**	**1**	**0**	**2**	**0**	**1**	**1**	**0**	**0**	**0**	**0**	**515**
**Klebsiella sp./bla NDM**													
Não Detectável	2	0	0	0	0	0	0	0	0	0	0	0	2
**Subtotal**	**2**	**0**	**0**	**0**	**0**	**0**	**0**	**0**	**0**	**0**	**0**	**0**	**517**
**Klebsiella sp./bla OXA-48**													
Não Detectável	2	0	0	0	0	0	0	0	0	0	0	0	2
**Subtotal**	**2**	**0**	**0**	**0**	**0**	**0**	**0**	**0**	**0**	**0**	**0**	**0**	**519**
**Klebsiella sp./KPC**													
Detectável	0	1	0	0	0	0	0	0	0	0	0	0	1
**Subtotal**	**0**	**1**	**0**	**0**	**0**	**0**	**0**	**0**	**0**	**0**	**0**	**0**	**520**
**Proteus mirabilis/bla IMP**													

Relatório Pesquisa de Genes de Resistência

Microrganismo /Gene Pesquisado	Jan/2019	Fev/2019	Mar/2019	Abr/2019	Maio/2019	Jun/2019	Jul/2019	Ago/2019	Set/2019	Out/2019	Nov/2019	Dez/2019	Total
Não Detectável	0	0	0	0	0	0	1	0	0	0	0	0	1
**Subtotal**	**0**	**0**	**0**	**0**	**0**	**0**	**1**	**0**	**0**	**0**	**0**	**0**	**521**
**Proteus mirabilis/bla KPC**													
Não Detectável	0	0	0	0	0	0	1	0	0	0	0	0	1
**Subtotal**	**0**	**0**	**0**	**0**	**0**	**0**	**1**	**0**	**0**	**0**	**0**	**0**	**522**
**Proteus mirabilis/bla NDM**													
Não Detectável	0	0	1	0	0	0	1	0	0	0	0	0	2
**Subtotal**	**0**	**0**	**1**	**0**	**0**	**0**	**1**	**0**	**0**	**0**	**0**	**0**	**524**
**Proteus mirabilis/bla OXA-48**													
Não Detectável	0	0	0	0	0	0	1	0	0	0	0	0	1
**Subtotal**	**0**	**0**	**0**	**0**	**0**	**0**	**1**	**0**	**0**	**0**	**0**	**0**	**525**
**Proteus mirabilis/blaVIM**													
Não Detectável	0	0	0	0	0	0	1	0	0	0	0	0	1
**Subtotal**	**0**	**0**	**0**	**0**	**0**	**0**	**1**	**0**	**0**	**0**	**0**	**0**	**526**
**Proteus penneri/bla NDM**													
Não Detectável	0	0	1	0	0	0	0	0	0	0	0	0	1
**Subtotal**	**0**	**0**	**1**	**0**	**0**	**0**	**0**	**0**	**0**	**0**	**0**	**0**	**527**
**Providencia sp./bla KPC**													
Não Detectável	0	1	0	0	0	0	0	0	0	0	0	0	1
**Subtotal**	**0**	**1**	**0**	**0**	**0**	**0**	**0**	**0**	**0**	**0**	**0**	**0**	**528**
**Providencia stuartii/bla KPC**													
Não Detectável	0	0	0	0	0	0	0	0	0	1	0	0	1
**Subtotal**	**0**	**0**	**0**	**0**	**0**	**0**	**0**	**0**	**0**	**1**	**0**	**0**	**529**
**Providencia stuartii/NDM**													
Não Detectável	0	0	0	0	0	0	0	0	0	1	0	0	1
**Subtotal**	**0**	**0**	**0**	**0**	**0**	**0**	**0**	**0**	**0**	**1**	**0**	**0**	**530**
**Pseudomonas aeruginosa/bla IMP**													
Não Detectável	1	0	0	0	0	0	0	0	0	0	0	0	1
**Subtotal**	**1**	**0**	**0**	**0**	**0**	**0**	**0**	**0**	**0**	**0**	**0**	**0**	**531**
**Pseudomonas aeruginosa/bla KPC**													
Não Detectável	0	0	0	1	0	0	0	3	1	4	1	0	10
**Subtotal**	**0**	**0**	**0**	**1**	**0**	**0**	**0**	**3**	**1**	**4**	**1**	**0**	**541**
**Pseudomonas aeruginosa/bla NDM**													
Não Detectável	2	0	0	0	0	0	0	0	0	0	0	0	2
**Subtotal**	**2**	**0**	**0**	**0**	**0**	**0**	**0**	**0**	**0**	**0**	**0**	**0**	**543**
**Pseudomonas aeruginosa/bla OXA-23**													

Relatório Pesquisa de Genes de Resistência

Microrganismo /Gene Pesquisado	Jan/2019	Fev/2019	Mar/2019	Abr/2019	Maio/2019	Jun/2019	Jul/2019	Ago/2019	Set/2019	Out/2019	Nov/2019	Dez/2019	Total
Não Detectável	1	0	0	0	0	0	0	0	0	0	0	0	1
**Subtotal**	**1**	**0**	**0**	**0**	**0**	**0**	**0**	**0**	**0**	**0**	**0**	**0**	**544**
**Pseudomonas aeruginosa/bla OXA-48**													
Detectável	0	0	0	0	0	0	0	0	0	0	1	0	1
Não Detectável	2	0	0	1	0	0	0	0	0	0	5	0	8
**Subtotal**	**2**	**0**	**0**	**1**	**0**	**0**	**0**	**0**	**0**	**0**	**6**	**0**	**553**
**Pseudomonas aeruginosa/bla SPM**													
Detectável	1	0	1	1	0	0	0	0	0	0	1	0	4
Não Detectável	1	0	6	3	5	1	3	3	1	2	8	0	33
**Subtotal**	**2**	**0**	**7**	**4**	**5**	**1**	**3**	**3**	**1**	**2**	**9**	**0**	**590**
**Pseudomonas aeruginosa/blaVIM**													
Não Detectável	1	0	6	3	5	0	3	0	0	0	1	0	19
**Subtotal**	**1**	**0**	**6**	**3**	**5**	**0**	**3**	**0**	**0**	**0**	**1**	**0**	**609**
**Pseudomonas aeruginosa/SPM**													
Não Detectável	0	0	0	0	0	0	0	0	0	2	0	0	2
**Subtotal**	**0**	**0**	**0**	**0**	**0**	**0**	**0**	**0**	**0**	**2**	**0**	**0**	**611**
**Pseudomonas sp./bla SPM**													
Não Detectável	0	0	0	0	0	4	1	0	0	0	0	0	5
**Subtotal**	**0**	**0**	**0**	**0**	**0**	**4**	**1**	**0**	**0**	**0**	**0**	**0**	**616**
**Pseudomonas sp./blaVIM**													
Não Detectável	0	0	0	0	0	0	1	0	0	0	0	0	1
**Subtotal**	**0**	**0**	**0**	**0**	**0**	**0**	**1**	**0**	**0**	**0**	**0**	**0**	**617**
**Serratia liquefaciens/bla KPC**													
Não Detectável	0	0	0	0	0	0	0	1	0	0	0	0	1
**Subtotal**	**0**	**0**	**0**	**0**	**0**	**0**	**0**	**1**	**0**	**0**	**0**	**0**	**618**
**Serratia marcescens/bla IMP**													
Não Detectável	0	0	0	0	0	0	0	0	0	0	1	0	1
**Subtotal**	**0**	**0**	**0**	**0**	**0**	**0**	**0**	**0**	**0**	**0**	**1**	**0**	**619**
**Serratia marcescens/bla KPC**													
Detectável	0	0	1	0	1	1	0	0	0	0	0	0	3
Não Detectável	0	0	0	0	0	0	0	3	1	2	1	0	7
**Subtotal**	**0**	**0**	**1**	**0**	**1**	**1**	**0**	**3**	**1**	**2**	**1**	**0**	**629**
**Serratia marcescens/bla NDM**													
Não Detectável	0	1	4	0	3	2	1	0	1	2	1	0	15
**Subtotal**	**0**	**1**	**4**	**0**	**3**	**2**	**1**	**0**	**1**	**2**	**1**	**0**	**644**
**Staphylococcus aureus/mec a**													

Relatório Pesquisa de Genes de Resistência

Microrganismo /Gene Pesquisado	Jan/2019	Fev/2019	Mar/2019	Abr/2019	Maio/2019	Jun/2019	Jul/2019	Ago/2019	Set/2019	Out/2019	Nov/2019	Dez/2019	Total
Detectável	3	0	0	0	0	0	0	0	0	2	0	0	5
Não Detectável	0	0	0	0	0	1	0	0	0	1	0	0	2
**Subtotal**	**3**	**0**	**0**	**0**	**0**	**1**	**0**	**0**	**0**	**3**	**0**	**0**	**651**
**Staphylococcus aureus/outros**													
Não Detectável	0	0	0	0	0	1	0	0	0	0	0	0	1
**Subtotal**	**0**	**0**	**0**	**0**	**0**	**1**	**0**	**0**	**0**	**0**	**0**	**0**	**652**
**Staphylococcus epidermidis/mec a**													
Detectável	0	0	0	0	0	0	0	0	0	2	0	0	2
**Subtotal**	**0**	**0**	**0**	**0**	**0**	**0**	**0**	**0**	**0**	**2**	**0**	**0**	**654**
	2	1	0	0	1	1	0	0	0	0	0	0	5
**Subtotal**	**2**	**1**	**0**	**0**	**1**	**1**	**0**	**0**	**0**	**0**	**0**	**0**	**659**
